# Enhancing anti-EGFRvIII CAR T cell therapy against glioblastoma with a paracrine SIRPγ-derived CD47 blocker

**DOI:** 10.1038/s41467-024-54129-w

**Published:** 2024-11-09

**Authors:** Tomás A. Martins, Deniz Kaymak, Nazanin Tatari, Fiona Gerster, Sabrina Hogan, Marie-Françoise Ritz, Valerio Sabatino, Ronja Wieboldt, Ewelina M. Bartoszek, Marta McDaid, Alexandra Gerber, Alicia Buck, Aisha Beshirova, Anja Heider, Tala Shekarian, Hayget Mohamed, Manina M. Etter, Philip Schmassmann, Ines Abel, Jean-Louis Boulay, Yasuyuki Saito, Luigi Mariani, Raphael Guzman, Berend Snijder, Tobias Weiss, Heinz Läubli, Gregor Hutter

**Affiliations:** 1 Brain Tumor Immunotherapy and Biology, Department of Biomedicine, University of Basel, Basel, Switzerland; 2 Cancer Immunotherapy, Department of Biomedicine, University of Basel, Basel, Switzerland; 3 Microscopy Core Facility, Department of Biomedicine, University of Basel, Basel, Switzerland; 4 Institute of Molecular Systems Biology, ETH Zurich, Zurich, Switzerland; 5 Department of Neurology, Clinical Neuroscience Center, University Hospital Zurich, Zurich, Switzerland; 6 Department of Neurology, University of Zurich, Zurich, Switzerland; 7 Experimental Immunology, Department of Biomedicine, University of Basel, Basel, Switzerland; 8 Swiss Institute of Allergy and Asthma Research, University of Zurich, Davos Wolfgang, Switzerland; 9 Department of Neurosurgery, University Hospital Basel, Basel, Switzerland; 10 Division of Molecular and Cellular Signaling, Department of Biochemistry and Molecular Biology, Kobe University Graduate School of Medicine, Kobe, Japan; 11 Department of Surgery, University Hospital Basel, Basel, Switzerland; 12 Department of Oncology, University Hospital Basel, Basel, Switzerland

**Keywords:** Cancer immunotherapy, CNS cancer, Cancer microenvironment

## Abstract

A significant challenge for chimeric antigen receptor (CAR) T cell therapy against glioblastoma (GBM) is its immunosuppressive microenvironment, which is densely populated by protumoral glioma-associated microglia and macrophages (GAMs). Myeloid immune checkpoint therapy targeting the CD47-signal regulatory protein alpha (SIRPα) axis induces GAM phagocytic function, but CD47 blockade monotherapy is associated with toxicity and low bioavailability in solid tumors. In this work, we engineer a CAR T cell against epidermal growth factor receptor variant III (EGFRvIII), constitutively secreting a signal regulatory protein gamma-related protein (SGRP) with high affinity to CD47. Anti-EGFRvIII-SGRP CAR T cells eradicate orthotopic EGFRvIII-mosaic GBM in vivo, promoting GAM-mediated tumor cell phagocytosis. In a subcutaneous CD19^+^ lymphoma mouse model, anti-CD19-SGRP CAR T cell therapy is superior to conventional anti-CD19 CAR T. Thus, combination of CAR and SGRP eliminates bystander tumor cells in a manner that could overcome main mechanisms of CAR T cell therapy resistance, including immune suppression and antigen escape.

## Abstract (web summary)

EGFRvIII-targeted CAR T cells have been proposed as a therapeutic option for patients with glioblastoma (GBM), however, clinical responses remain suboptimal. Here the authors engineer anti-EGFRvIII CAR T cells to secrete an optimized SIRPγ-derived CD47 blocker, showing that combining CAR T cell effector functions with enhanced macrophage-mediated tumor cell phagocytosis improves anti-tumor efficacy in preclinical models.

## Introduction

Glioblastoma (GBM) is the most aggressive, malignant primary brain tumor in adults. Surgical resection, chemo- and radiotherapy regimens are not curative, invariably leading to recurrent disease. The median survival time for patients with GBM is 15 months, underscoring an urgent need for a significant breakthrough in effective medical treatment^1,2^.

Chimeric antigen receptor (CAR) T cell-based immunotherapies have shown efficacy in treating hematological malignancies. However, developing effective CAR T cell therapies against solid tumors remains challenging^3–5^. Epidermal growth factor receptor (EGFR) variant III (EGFRvIII) is a tumor-specific antigen expressed in ~40% of GBM cases^6^. Although strictly expressed on tumor cells, EGFRvIII mutations arise with concomitant EGFR amplification during clonal evolution events, resulting in EGFRvIII-mosaic tumors^6,7^. A clinical trial of anti-EGFRvIII CAR T cells against recurrent GBM (NCT01454596) showed safety and transient efficacy but failed to produce long-lasting therapeutic responses due to adaptive resistance and antigen escape^8^. Conversely, targeting GBM-associated antigens is controversial due to the risk of on-target/off-tumor toxicity^9^. Recently, a modified version of this anti-EGFRvIII CAR that secretes a T cell-engaging EGFR blocker showed impressive responses and a tolerable toxicity profile after brain intraventricular application in a small cohort of patients with recurrent GBM^10^.

Immune checkpoint therapy has shown promising results against solid tumors. However, the highly immunosuppressive immune tumor microenvironment (iTME) of GBM severely limits the efficacy of immune checkpoint blockade^11^. Thus, understanding the complex context-dependent interactions of GBM with the surrounding iTME is crucial for effectively targeting these tumors using immunotherapeutic approaches^12^. The dominant cell populations in the GBM immune compartment are protumoral brain-resident microglia (MG) and peripheral monocyte-derived macrophages (MDMs), collectively termed glioma-associated microglia and macrophages (GAMs)^13–15^. GAM phagocytic activity is partially regulated through the CD47-signal regulatory protein alpha (SIRPα) axis^16^ and tumor cells co-opt CD47 overexpression to evade targeting by GAMs^15,17^. CD47 blockade rescues GAM phagocytic function in GBM-bearing mice, producing robust antitumoral responses in vivo^18,19^. However, clinical studies of systemic anti-CD47 monotherapy report low bioavailability within solid tumors and moderate levels of toxicity^20–22^.

In this work, we address the main challenges of myeloid immunosuppression and clonal heterogeneity that limit the efficacy of anti-cancer immunotherapies against GBM^23^. To synergistically target heterogeneous GBM and the immunosuppressive iTME, we propose the combination of intratumoral (i.t.) CAR T cell therapy and GAM modulation with a T cell-secreted signal regulatory protein gamma (SIRPγ)-related protein (SGRP) for targeting antigen-expressing and bystander tumor cells, yielding a near-complete clearance of EGFRvIII-mosaic GBM in mouse models. The therapeutic strategy shown here could improve CAR T cell therapies against GBM and other solid tumors.

## Results

### CAR T cells that secrete a SIRPγ-derived blocker of the CD47-SIRPα axis

**Fig. 1:**
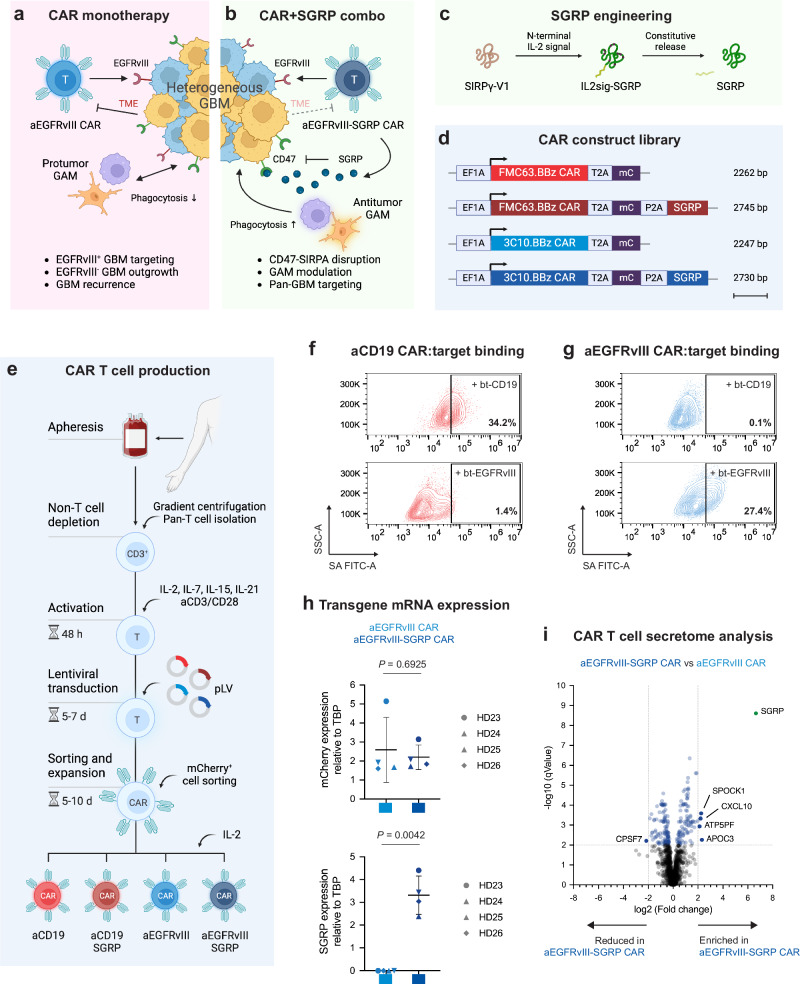
Anti-EGFRvIII-SGRP CAR T cells constitutively secrete SGRP, enabling CD47-SIRPα axis disruption. **a** Mechanism of action of conventional aEGFRvIII CAR T cell monotherapy in GBM. **b** Proposed aEGFRvIII-SGRP CAR T cell combination therapy whereby SGRP-mediated CD47 blockade induces phagocytic modulation of GAMs in the context of EGFRvIII-heterogenous GBM and its immunosuppressive iTME. **c** Outline of the SGRP engineering strategy, including specific AA substitutions to the endogenous human SIRPγ-V1 sequence and addition of an N-terminal IL-2 signal sequence (IL2sig) leading to constitutive SGRP secretion. **d** Polycistronic lentiviral constructs encoding mCherry (mC)-labeled aCD19 CAR or aEGFRvIII CAR under the control of *EF1A* promoter ± SGRP secretion. **e** Workflow of CAR T cell production applied throughout the study. **a**–**e** Created in BioRender. Hutter, G. (2022) BioRender.com/u48r093. Representative plots of CAR:target protein binding by aCD19 CAR T cells (**f**) or aEGFRvIII CAR T cells (**g**) to CAR-bound biotinylated (bt)-CD19 (top plots) or bt-EGFRvIII (bottom plots); *n* = 2 healthy donors (HDs) assessed per CAR. **h** TATA-box binding protein (TBP)-normalized expression of mCherry and SGRP detected by real-time quantitative PCR (RT-qPCR) in aEGFRvIII CAR or aEGFRvIII-SGRP CAR T cells, showing mCherry expression in CARs transduced with either construct and SGRP expression specifically in aEGFRvIII-SGRP CARs; *n* = 4 HDs. Data are presented as scatter plots with mean values ± SD. Statistical differences were assessed by two-sided unpaired *t* tests with Welch’s correction. **i** Differentially secreted proteins in aEGFRvIII-SGRP CAR- vs aEGFRvIII CAR-conditioned media, highlighting the presence of SGRP exclusively in aEGFRvIII-SGRP CAR; *n* = 2 HDs. Mean SGRP expression: $-\log 10{{{\rm{qValue}}}}/\log 2{{{\rm{foldchange}}}}=8.60/6.65$. Source data are provided as a Source Data file. Source data for (**i**) are provided as Supplementary Data 1.

Antigen escape is a significant mechanism of anti-EGFRvIII CAR T cell therapy resistance^8^ (Fig. 1a). We propose a fourth-generation CAR design, whereby anti-EGFRvIII CAR T cells constitutively release SGRP, a protein with high affinity to CD47 (Fig. 1b). The reported dissociation constant (*K*_D_) of SIRPα binding to human CD47 is 279 nM^24^. A SIRPα analog, CV1, recently used in CAR constructs for peripheral tumors, has a *K*_D_ of 11.1 pM^25^. In comparison, SGRP used in the present study has a reported *K*_D_ of 92 pM^26^, far outcompeting endogenous human phagocyte-expressed SIRPα and SIRPα analogs in the literature.

We equipped anti-EGFRvIII-BBz CAR T cells (3C10.BBz^27^, termed aEGFRvIII CAR) with a secretable SGRP, resulting in the generation of aEGFRvIII-SGRP CAR (Fig. 1c). Synthetic SGRP shares a strong identity with the endogenous SIRPγ binding domain (SIRPγ-V1) amino acid (AA) sequence (Supplementary Fig. 1a and Supplementary Table 1) and binds to human CD47 in a similar manner as human and murine SIRPα (Supplementary Fig. 1b). Specifically, we generated *EF1A*-driven polycistronic constructs encoding overall identical CAR structures targeting either EGFRvIII or control antigen CD19 (FMC63.BBz^28^, termed aCD19 CAR), a mCherry (mC) fluorescent reporter, and a secretable SGRP. The resulting polyproteins were generated by flanking T2A or P2A self-cleaving peptide sequences (Fig. 1d and Supplementary Table 2) and were incorporated into lentiviral expression vectors (Supplementary Fig. 1c).

Activated T cells were stably transduced with lentiviral supernatants, expanded, and sorted for mCherry-positivity (Fig. 1e and Supplementary Fig. 1d, e). As intended, aCD19 and aEGFRvIII CARs showed specific binding affinity to their respective target but not to mismatched target proteins (Fig. 1f, g) or wild-type (wt) EGFR protein (Supplementary Fig. 1f).

We confirmed the presence of similar amounts of mCherry mRNA in aEGFRvIII CAR and aEGFRvIII-SGRP CAR, and SGRP mRNA specifically in aEGFRvIII-SGRP CAR T cells from four T cell donors (Fig. 1h). In conditioned media from CAR T cell cultures of two donors, SGRP was the most differentially enriched protein in the secretome of aEGFRvIII-SGRP CAR compared to aEGFRvIII CAR, detected by liquid chromatography–mass spectrometry (LC-MS; Fig. 1i and Supplementary Fig. 1g). The complete list of detected proteins is provided as Supplementary Data 1. The identified peptide sequences included SGRP-specific AA modifications, confirming specific SGRP detection rather than contamination with peptides of highly conserved SIRP-family proteins (Supplementary Fig. 1h).

### SGRP-mediated CD47 blockade does not impair CAR T cell function

To identify GBM cell line targets for in vitro CAR T cell testing, we profiled the surface expression of our targets of interest on four GBM cell lines (U251, U87, U251vIII with transgenic (tg) overexpression of EGFRvIII (vector sequence in Supplementary Table 3), BS153 with endogenous EGFRvIII expression), one Burkitt’s lymphoma cell line (Raji) and one normal neural stem cell (NSC) line (NSC197). Throughout the study, we used aCD19 CAR and aCD19-SGRP CAR as controls in GBM experiments and Raji as CD19^+^ target cells (Supplementary Fig. 2a, left plot). We confirmed the surface expression of EGFRvIII on U251vIII and BS153 (99.3% and 28.9%, respectively), while U251 and U87 showed substantially lower expression levels (16.4% and 23.5%, respectively) (Supplementary Fig. 2a, center plot). Before any in vitro and in vivo experiments, U251vIII and BS153 were sorted for their EGFRvIII^+^ cell population, while U251 and U87 were sorted for their EGFRvIII^-^ cell fraction (Supplementary Fig. 2b). All cell lines were validated for EGFRvIII expression throughout the study. Furthermore, we also confirmed CD47 overexpression in all tumor cell lines (U251vIII: 56.5%, U251: 51.4%, U87: 53.5%, BS153: 80.6% and Raji: 58.4%), compared to NSCs used as a normal brain cell control (Supplementary Fig. 2a, right plot).

**Fig. 2:**
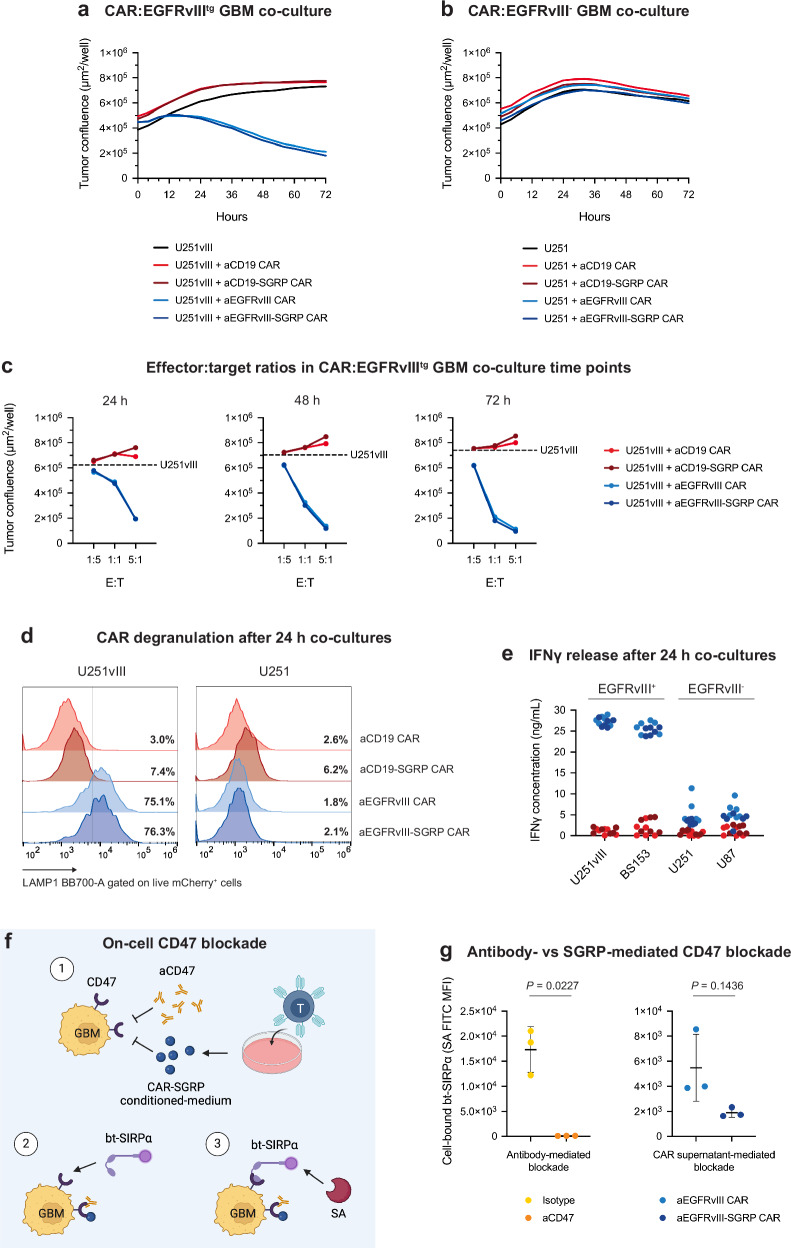
Anti-EGFRvIII-SGRP CAR T cells have comparable in vitro efficacy and on-target activation to conventional anti-EGFRvIII CAR T cells. **a** Assessment of CAR T cell on-target killing capacity by co-culture time-lapse of nEGFP^+^ U251vIII with mCherry^+^ target-specific (aEGFRvIII CAR ± SGRP) or nonspecific (aCD19 CAR ± SGRP) at a 1:1 E:T ratio for 72 h. **b** Assessment of CAR T cell off-target killing by co-culture time-lapse of nEGFP^+^ U251 with mCherry^+^ CAR T cells at a 1:1 E:T ratio for 72 h. **c** Dose-dependent CAR T cell killing capacity in a co-culture with U251vIII at defined time points. Dashed lines represent the mean confluence in control wells with only U251vIII cells. **a**–**c** Curves and dots represent the mean of duplicate measurements. **d** Representative histograms of CAR T cell degranulation in 24 h co-cultures with U251vIII, BS153, U251, or U87 (gated on live mCherry^+^ singlets); Conditions were performed in duplicates with *n* = 2 HDs. **e** IFNγ release detected by ELISA in supernatants of CAR T cells co-cultured with EGFRvIII^+^ or EGFRvIII^-^ GBM cell lines. Conditions were performed in triplicates with *n* = 2 HDs. **f** Schematic illustration of the experimental setup of an SGRP/aCD47 blocking assay on CD47^+^ BS153 cells. Created in BioRender. Hutter, G. (2024) BioRender.com/i60q967. **g** Scatter plots of the MFI of individual wells. Data are presented as mean values ± SD. Conditions were performed in triplicates. Statistical differences were assessed by two-sided unpaired t tests with Welch’s correction. Source data are provided as a Source Data file.

We then established co-cultures of mCherry^+^ conventional CAR T or SGRP-secreting CAR T cells with GBM cells expressing a nuclear-restricted EGFP (nEGFP) as a fluorescence viability reporter. A decline of the nEGFP signal in the co-cultures indicated a reduction in tumor viability. Time-lapse imaging revealed the specific cytolytic effect of aEGFRvIII and aEGFRvIII-SGRP CAR against U251vIII cells (Fig. 2a). aEGFRvIII CAR also showed similar efficacy against BS153 cells (Supplementary Fig. 2c). By contrast, no measurable cytotoxicity was observed against U251, an EGFRvIII-negative-sorted GBM cell line (Fig. 2b). At various time points and effector-target (E:T) ratios, only target-specific (aEGFRvIII ± SGRP) CAR T cells displayed a dose-dependent effect against EGFRvIII^tg^ GBM cells (Fig. 2c). Moreover, the CAR T cell cytolytic capacity remained unaffected by SGRP secretion in co-cultures with U251vIII (Fig. 2c and Supplementary Movies 1–4) or U251 (Supplementary Fig. 2d and Supplementary Movies 5–8), as shown by the analogous response to aEGFRvIII and aEGFRvIII-SGRP CAR.

Flow cytometry (FC) analysis of CAR T cells in 24 h co-cultures showed that target-specific CAR T cells expressed higher levels of LAMP1 in response to EGFRvIII^+^ GBM cell lines (Fig. 2d). We also detected significantly increased IFNγ in co-cultures of target-specific CAR T cells with EGFRvIII^+^ but not EGFRvIII^-^ GBM cell lines (Fig. 2e). Notably, our CAR T cell production protocol yielded almost exclusively CD4^+^ T cells (Supplementary Fig. 2e).

To determine the CD47-blocking capacity of T cell-secreted SGRP compared to anti-human CD47 antibody (aCD47) in vitro, we performed an on-cell blocking assay (Fig. 2f; antibody dilutions in Supplementary Table 4). aCD47 treatment induced a potent blockade of the CD47-SIRPα interaction (Fig. 2g, left dot plot), whereas treatment with aEGFRvIII-SGRP CAR-conditioned medium produced only a slight impairment (Fig. 2g, right dot plot). Since CD47 is ubiquitously expressed^16^ and SGRP has a very high affinity to CD47, we hypothesize that SGRP may be partially captured in autocrine or paracrine loops by CAR T cells, reducing the amount of unbound SGRP collected from cell culture supernatants. Importantly, CD47 expression is required for CAR T and peripheral T cell survival, as shown by studies using CD47 mutant T cells^29,30^. Despite constitutive secretion of SGRP, CD47 surface expression remained unchanged on aEGFRvIII-SGRP CAR T cells (Supplementary Fig. 2f).

In an attempt to quantify the effects of CAR targeting and SGRP-mediated macrophage modulation in vitro, we performed a 3 h-phagocytosis assay combining a mosaic GBM model consisting of EGFRvIII^-^ U87, EGFRvIII^+^ U251vIII cells with donor-matched MDMs and CAR T cells in a 1:1:1:1 ratio (Supplementary Fig. 2g). In this short-term phagocytosis assay, we assessed: (1) MDM polarization, by human CD163, CD206, HLA-DR, and CD86; (2) phagocytic marker expression, by human CD209; (3) antigen presentation, by human SIGLEC-1, and (4) tumor cell phagocytosis. However, in this in vitro system, we found no significant differences between conditions involving conventional CARs and SGRP-secreting CARs.

Nevertheless, in vitro models overlook crucial cell interactions in the context of the GBM TME, namely the impact of other phagocytes, e.g., MG and dendritic cells (DCs), on in vivo tumor clearance. Thus, understanding the effect of T cell-secreted SGRP requires a GAM-infiltrated in vivo GBM model.

### Anti-EGFRvIII-SGRP CAR improves the survival of GBM xenografts

Many anti-EGFRvIII CAR preclinical studies show effective cytotoxicity but fail to model the heterogeneity of human GBM^31,32^. Although preclinical aEGFRvIII CAR monotherapy has demonstrated efficacy, this strategy translates poorly to the clinical setting^8^. We first tested the efficacy of aEGFRvIII CAR and aCD47 monotherapies in an orthotopic EGFRvIII^+^ GBM xenograft model (Supplementary Fig. 3a–c). Morbidity scoring was performed as indicated in Supplementary Table 5. Overall survival analysis (Supplementary Fig. 3d) showed that local aCD47 monotherapy failed to improve survival (median survival: 34.5 d) compared to the isotype antibody control group (median survival: 30.5 d). Conversely, aEGFRvIII CAR treatment (median survival: 65.5 d) led to 40% survival 90 d after tumor implantation, starkly contrasting with aCD19 CAR control (median survival: 29 d). Bioluminescence assessment confirmed lower tumor burden in aEGFRvIII CAR-treated mice, assessed by the mean bioluminescence counts within a defined region of interest (ROI) applied throughout the study (Supplementary Fig. 3e, f).

**Fig. 3:**
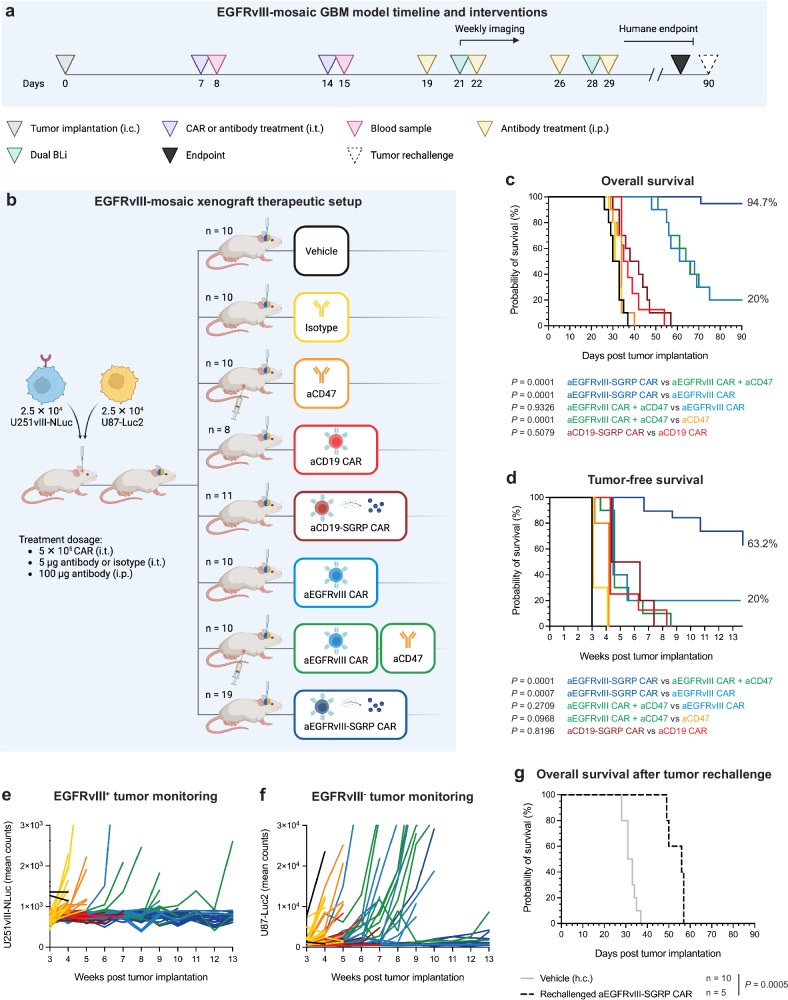
I.t. anti-EGFRvIII-SGRP CAR T cell therapy leads to survival benefit and long-term tumor control in an EGFRvIII-heterogenous GBM xenograft model. **a** Experimental treatment and monitoring schedule. Animals were treated twice – at 7 and 14 days – after intracerebral (i.c.) tumor implantation using the same stereotactic coordinates and routinely monitored for clinical signs, weekly dual bioluminescence imaging (BLi), and morbidity/survival assessment. Plasma for cytokine analysis was collected on day 15–24 h after the second i.t. treatment. Systemic aCD47 therapy was administered in the relevant treatment groups on days 19, 22, 26, and 29. Animals were euthanized upon reaching the humane endpoint. Upon reaching 90 days of tumor-free survival, aEGFRvIII-SGRP CAR-treated animals (*n* = 5) were tumor-rechallenged in the ipsilateral hemisphere using the same stereotactic coordinates. **b** Experimental setup of orthotopic xenograft experiments in NSG mice encompassing co-implanted EGFRvIII^+^ U251vIII and EGFRvIII^-^ U87 GBM cell lines mimicking tumor heterogeneity and therapeutic/control cohorts including local CAR T cell or antibody monotherapies or combinations (aCD19 CAR *n* = 8; aCD19-SGRP CAR *n* = 11; aEGFRvIII-SGRP CAR *n* = 19; others *n* = 10). CAR T cell dose: 5 × 10^5^ cells delivered i.t.; Antibody dose: 5 µg delivered i.t. or 100 µg delivered i.p. **a**, **b** Created in BioRender. Hutter, G. (2024) BioRender.com/e30g370. **c** Kaplan–Meier plot of overall survival (in days). **d** Kaplan–Meier plot of tumor-free survival (in weeks), combining survival assessment with BLi monitoring scores. **c**, **d** Two-sided log-rank tests were used to compare treatment/control groups. **e**, **f** Tumor progression in EGFRvIII-mosaic xenografts was monitored for each mouse using differential BLi with either FFz or D-luciferin substrates (in weeks). **e** U251vIII NLuc-reporter BLi curves. **f** U87 Luc2-reporter BLi curves. **c**–**f** The data were pooled from three independent experiments. **g** Kaplan–Meier plot of overall survival of aEGFRvIII-SGRP CAR-cured, tumor-rechallenged animals (in days). A two-sided log-rank test compared the rechallenge group (*n* = 5) to the historic vehicle control group (vehicle (h.c.); *n* = 10). Source data are provided as a Source Data file.

To assess the efficacy of CAR + SGRP combination therapy against GBM, we established a preclinical EGFRvIII-mosaic GBM mouse model with orthotopic tumor implantation and i.t. therapy administration (Fig. 3a, b). First, we sorted U251vIII and U87 cell lines for their EGFRvIII^+^ and EGFRvIII^-^ cell fractions, respectively, and co-injected them in a 1:1 ratio. Animals received two identical i.t. doses (5 × 10^5^ CAR T cells or 5 µg of antibody) on days 7 and 14 post tumor implantation. For better equipoise between antibody-based CD47 blockade (aCD47) and constitutive CAR-mediated SGRP release, animals receiving aCD47 as monotherapy or in combination with aEGFRvIII CARs (aEGFRvIII CAR + aCD47) received four additional doses (100 µg of antibody) delivered intraperitoneally (i.p.) within the two weeks following the i.t. treatment regimen. To monitor U251vIII and U87 cell populations separately in vivo, we labeled U251vIII with NanoLuciferase (NLuc) and U87 with Luciferase2 (Luc2) bioluminescence reporters (Supplementary Table 3).

Overall survival analysis (Fig. 3c) showed that aCD47 monotherapy failed to improve survival (median survival: 34 d) compared to an isotype antibody control group (median survival: 32 d). Notably, aggressive tumor models in the NSG context are difficult to treat using even higher doses of aCD47 antibodies^18^. aCD19-SGRP CAR therapy (median survival: 40 d) was not significantly better than aCD19 CAR treatment (median survival: 36 d). aEGFRvIII CAR monotherapy eliminated GBM in 20% of treated animals (Fig. 3c, d). However, the combination of aEGFRvIII CAR + aCD47 failed to improve survival compared to aEGFRvIII CAR monotherapy (20%). In contrast, aEGFRvIII-SGRP CAR was extraordinarily potent in this challenging GBM model. A near-complete therapeutic response was observed, with 94.7% overall and 63.2% tumor-free survival (Fig. 3c, d).

Differential bioluminescence monitoring of U251vIII-NLuc (Fig. 3e) and U87-Luc2 (Fig. 3f) confirmed that a majority of EGFRvIII^-^ tumors were eradicated after aEGFRvIII-SGRP CAR treatment. Individual bioluminescence plots of EGFRvIII^+^ and EGFRvIII^-^ tumor burden in aEGFRvIII CAR, aEGFRvIII CAR + aCD47 and aEGFRvIII-SGRP CAR treatment groups are shown in Supplementary Fig. 4a. A direct BLi comparison and quantification on week 7 post-tumor implantation shows significant differences in EGFRvIII^+^ and EGFRvIII^-^ tumor burden in aEGFRvIII-SGRP CAR compared to aEGFRvIII CAR and aEGFRvIII CAR + aCD47 treatment groups (Supplementary Fig. 4b, c).

To further elucidate the mechanism of aEGFRvIII-SGRP CAR-mediated tumor clearance, we performed an in vivo experiment with orthotopic EGFRvIII^-^ (U87) or EGFRvIII-mosaic (U87 + U251vIII) tumor implantation and treatment with aEGFRvIII-SGRP or aCD19-SGRP CAR (Supplementary Fig. 4d). Three days after the treatment, we collected the tumor-implanted brain hemisphere to characterize condition-specific immune cell abundance (cluster definition is shown in Supplementary Fig. 4e). Treatment with aEGFRvIII-SGRP CAR resulted in higher T cell frequencies in the tumor-implanted brain hemisphere compared to aCD19-SGRP CAR, regardless of tumor type (Supplementary Fig. 4f). Notably, aEGFRvIII-SGRP CAR treatment led to a higher influx of neutrophils, MDMs, and monocyte-derived DCs (moDCs)/conventional type 1 DCs (cDC1s) and an increase in CD25^hi^CD4^+^ CAR T cells exclusively in EGFRvIII-mosaic tumors (Supplementary Fig. 4f). This suggests that the benefit of SGRP-mediated CD47 blockade combined with CAR-mediated tumor killing relies on CAR target priming, leading to CAR T cell activation and persistence and enhanced myeloid cell infiltration to the brain.

Tumor rechallenge of aEGFRvIII-SGRP CAR-cured mice in the GBM cohort of Fig. 3c, d resulted in prolonged survival compared to the historic vehicle control group (Fig. 3g). Interestingly, tumor monitoring after rechallenge revealed preferential control of the EGFRvIII^+^ fraction (Supplementary Fig. 5a). Residual aEGFRvIII-SGRP CAR T cells in tumor-free brains on day 90 post tumor implantation were few and mostly found at the brain periphery (Supplementary Fig. 5b, c), suggesting a potential role for host innate immune cell priming, or lasting TME alterations from aEGFRvIII-SGRP CAR treatment, resulting in reduced tumor engraftment.

These results demonstrate that constitutive CAR-secreted SGRP is a superior combination partner to EGFRvIII-specific CAR T cells compared to antibodies. Notably, anti-CD47 clone B6.H12 was used in our experiments because of the limited availability of the superior clone Hu5F9-G4^19^. Owing to the ubiquitous expression pattern of CD47^16^, the presence of competing host “anti-CD47-consumer” cells likely contributes to the significant difference in efficacy observed for CAR + aCD47 and CAR + SGRP. During antigen-positive tumor cell lysis, aEGFRvIII-SGRP CAR T cells come into close contact with antigen-negative bystander tumor cells. We hypothesize that the extended secretion of SGRP in these tumor niches during tumor cytolysis is a crucial factor for the efficacy of aEGFRvIII-SGRP CAR.

### Anti-EGFRvIII-SGRP CAR specifically induces CCL3 in the periphery

To elucidate potential pathways of innate immune activation upon locoregional CAR T cell GBM treatments, we conducted a human immuno-oncology-targeted proteomic analysis of plasma samples collected 24 h after the second treatment (day 15 post-tumor implantation). We analyzed 92 proteins on two complementary datasets encompassing 54 plasma samples from individual mice (Supplementary Fig. 5d). Baseline protein expression was determined using plasma from sex- and age-matched healthy control mice. Source data is provided as Supplementary Data 2.

**Fig. 4:**
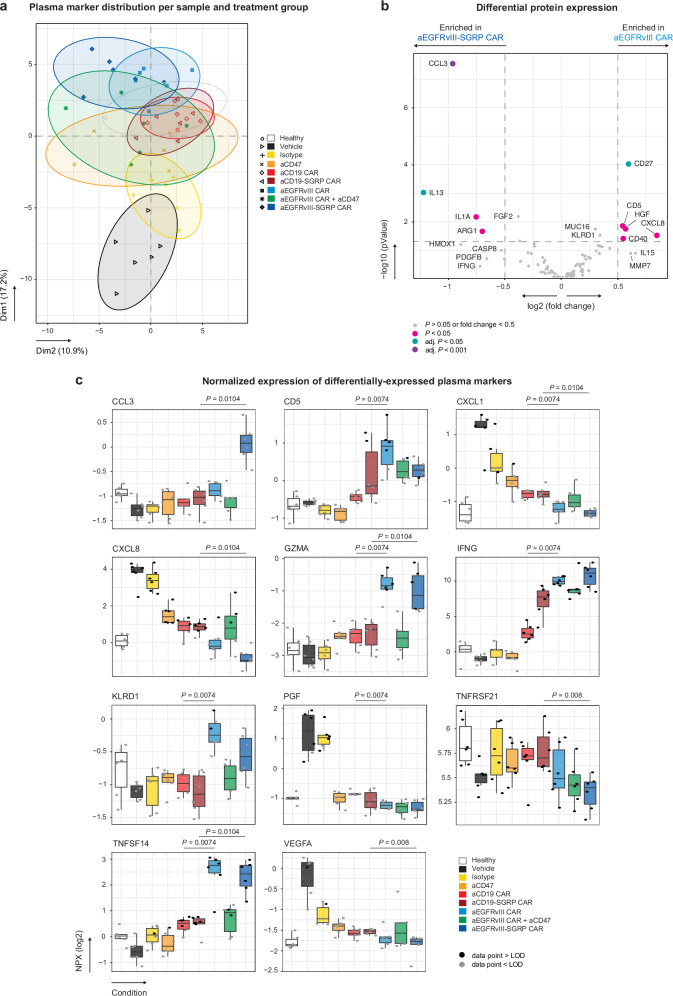
Local anti-EGFRvIII-SGRP CAR T cell therapy induces a potent peripheral inflammatory mediator response. **a** Non-metric multidimensional scaling (NMDS) plot of 92 examined soluble proteins in mouse plasma determined by proximity extension assay. Each data point represents one animal (*n* = 6 per condition). The ellipses represent the 95% confidence interval of each condition. **b** Differential expression analysis of aEGFRvIII-SGRP CAR vs aEGFRvIII CAR treatment groups. A two-sided test was used based on the limma-trend method with empirical Bayes moderation applied for variance estimation. Significant differences in protein expression are represented by differently colored data points: adj. *P* < 0.001, purple; adj. *P* < 0.05, teal; *P* < 0.05, pink; *P* > 0.05 or fold change <0.5, light grey. **c** Box plots with individual data points showing the normalized protein expression (NPX) of significant innate immune surrogate markers in plasma. Each data point represents one animal (*n* = 6 per condition). Data points above the assay’s limit of detection (>LOD) are illustrated in black, and those below (<LOD) are displayed in grey. The boxes’ central line represents the median, with the 75th percentile at the upper bound, the 25th percentile at the lower bound, and the whiskers representing all samples lying within 1.5 times the interquartile range. Statistics were calculated for the comparisons of interest using two-sided Mann–Whitney-U tests with Benjamini–Hochberg correction; Only significant statistics are shown. The experiment was repeated with two independent datasets with 16 overlapping biological replicates between the datasets. Source data are provided as Supplementary Data 2.

We first visualized the distribution of all assessed markers, which revealed that aEGFRvIII CAR- and aEGFRvIII-SGRP CAR-treated plasma segregated the furthest from vehicle controls (Fig. 4a). To examine the peripheral immune response associated with the superior aEGFRvIII-SGRP CAR treatment, we performed differential expression analysis against aEGFRvIII CAR monotherapy (Fig. 4b), which showed a significant enrichment of CCL3 in aEGFRvIII-SGRP CAR-treated plasma. To identify additional immune markers associated with aEGFRvIII CAR ± SGRP, we screened for differences between SGRP-secreting CARs (aEGFRvIII-SGRP CAR vs aCD19-SGRP CAR) or non-SGRP-secreting CARs (aEGFRvIII CAR vs aCD19 CAR) across all 92 markers (Fig. 4c). Significant differences were observed for CCL3, CXCL1, CXCL8, GZMA, TNFRSF21, TNFSF14 and VEGFA expression between SGRP-secreting CAR T cell treatments, and CD5, CXCL1, GZMA, IFNG, KLRD1, PGF and TNFSF14 expression between non-SGRP-secreting CAR T cell monotherapies.

The myeloid chemoattractant CCL3 emerged as a significantly upregulated protein in the plasma of mice treated with aEGFRvIII-SGRP CAR, potentially shaping peripheral responses to local antitumor therapy, promoting antigen presentation in tumor-draining lymph nodes or inducing T cell proliferation and differentiation^33^. To investigate whether CCL3 plays a crucial role in aEGFRvIII-SGRP CAR efficacy, we administered a CCL3-neutralizing antibody i.p. with concomitant i.t. CAR T cell therapy (Supplementary Fig. 5e). Control groups included an isotype antibody and PBS-injected animals. Despite CCL3 blockade, we observed no reduction in the efficacy of aEGFRvIII-SGRP CAR T cells compared to control groups (Supplementary Fig. 5f). The slight decrease in survival across all treatment/control groups compared to aEGFRvIII-SGRP CAR treatment in Fig. 3c could be attributed to overall lower activity of the T cell batch or the significant increase in frequency and duration of animal interventions.

### Anti-EGFRvIII-SGRP CAR T elicits EGFRvIII-mosaic GBM rejection

**Fig. 5:**
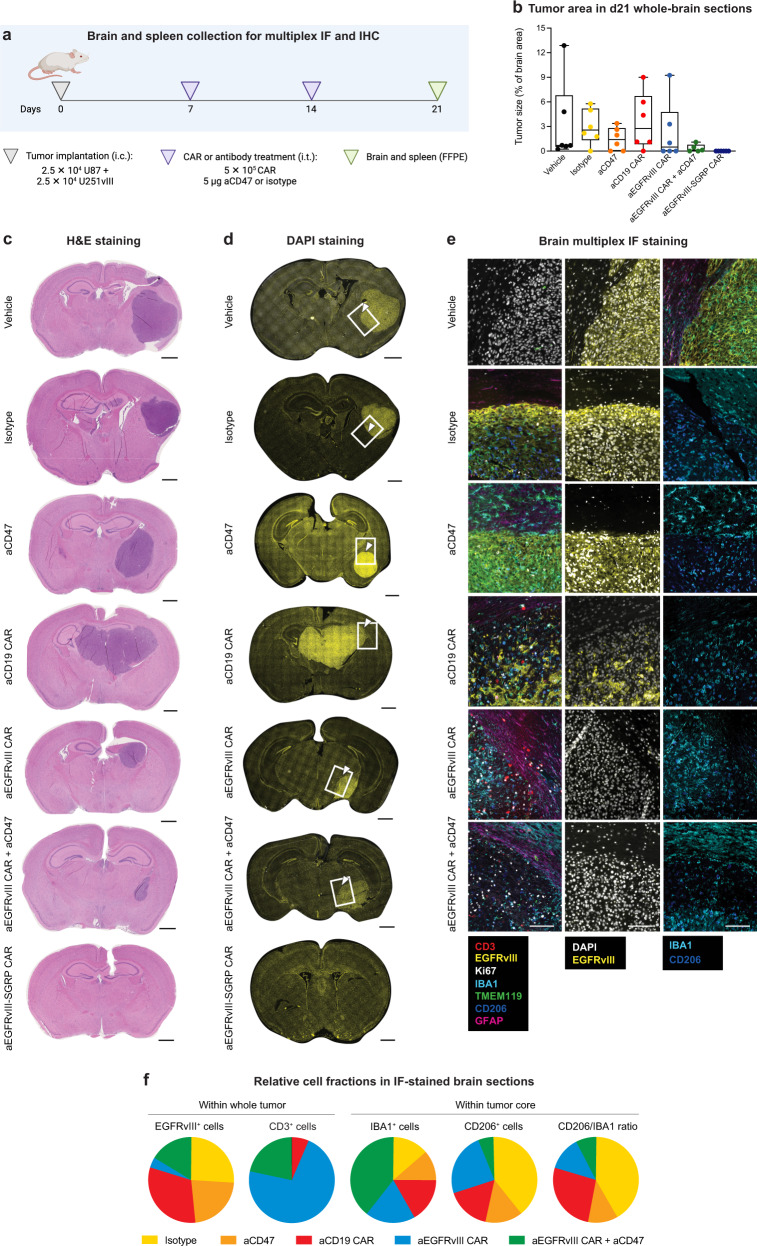
EGFRvIII-specific CAR T cells persist within the tumor, but only anti-EGFRvIII-SGRP CAR T cells eradicate EGFRvIII-mosaic GBM in histological brain sections. **a** Brain and spleen collection at an intermediate post-therapeutic time point for multiplex immunofluorescence (IF). Created in BioRender. Hutter, G. (2024) BioRender.com/t61q547. **b** Histomorphological brain tumor size assessment on day 21 post-tumor implantation. The boxes’ central line represents the median, with the 75th percentile at the upper bound, the 25th percentile at the lower bound, and the whiskers representing all samples from min to max values. All comparisons were non-significant using a two-sided one-way ANOVA with Dunnett’s multiple comparisons. **c** H&E-stained sections of representative tumor-burdened brains on day 21 post-tumor implantation and seven days after the second treatment. Coronal sections of cerebrum from different treatment groups; Scale bars: 1000 µm. **d** DAPI-stained (yellow) stitched assemblies of coronal brain sections used for subsequent IF multiplexing with inserts and arrowheads highlighting regions magnified in (**e**); Scale bars: 1000 µm. **b**–**d** The data were pooled from two independent experiments (aEGFRvIII CAR + aCD47 *n* = 5; others *n* = 6), and stainings were performed individually for each histological slide and staining cycle. **e** Representative micrographs of multiplexed IF assessments (*n* = 3 per condition); *Left column:* Overlays of 7 TME/tumor and proliferation markers; *Center column*: Overlays of DAPI and EGFRvIII staining; *Right column:* Overlays of CD206 and IBA1 staining; Scale bars: 50 µm. The data were pooled from two independent experiments, and stainings were performed individually for each histological slide and staining cycle. **f** Pie charts displaying relative comparisons of the percentage of marker-positive cells per all cells within the whole tumor or in the tumor core across experimental conditions (aCD19 CAR *n* = 1; aCD47 *n* = 3; others *n* = 2). No tumors were detected in any of the aEGFRvIII-SGRP CAR-treated brains analyzed. Source data are provided as a Source Data file and Supplementary Data 3.

To characterize the response of GBM xenografts to aEGFRvIII-SGRP CAR therapy, we collected spleen and brain tissue at an intermediate time point (7 days after the second i.t. treatment, day 21 post tumor implantation) for a histological workup (Fig. 5a). Tumor size assessment calculated from hematoxylin and eosin (H&E)-stained brain histological sections confirmed the persistence of intracerebral tumor in all but aEGFRvIII-SGRP CAR-treated animals, where we found only tumor remnants (Fig. 5b, c), precluding us from a detailed multidimensional analysis of the iTME in this condition. However, using anti-human CD3 immunohistochemistry (IHC) and applying an earlier time point (day 13 post tumor implantation) for the collection of aEGFRvIII-SGRP CAR-treated brains (Supplementary Fig. 6a), we confirmed the persistence of CAR T cells in tumor core and tumor rim in all CAR conditions (Supplementary Fig. 6b–d).

Multiplexed IF analysis focused on GAMs (murine IBA1, TMEM119, and CD206), astrocytes (murine GFAP), CAR T cells (human CD3), and tumor surface/proliferation markers (human EGFRvIII, and Ki67) within and surrounding tumor regions (Fig. 5d, e). The quantification of post-therapy EGFRvIII expression confirmed the selective elimination of EGFRvIII^+^ tumor cells after EGFRvIII-specific CAR treatment, accompanied by persisting EGFRvIII^-^ tumor cells, except after aEGFRvIII-SGRP CAR treatment, where tumors were eradicated (Fig. 5f). CD3^+^ cells were found in all CAR treatment/control paradigms, albeit in higher relative numbers in aEGFRvIII CAR-treated brains (Fig. 5f). Furthermore, the analysis of GAM markers revealed an overall increase in IBA1^+^ myeloid cell influx in aEGFRvIII CAR + aCD47-treated tumors (Fig. 5f) and a reduction of CD206^+^ cells in aCD47-treated tumors, consistent with a previous study^34^ (Fig. 5f). Alternatively, we showed that anti-mouse CD68 IHC of aEGFRvIII-SGRP CAR-treated brains on day 13 post-tumor implantation (compared to day 21 for all other conditions; Supplementary Fig. 6a) had a more pronounced density of GAMs on sites of tumor scarring (Supplementary Fig. 6e–g). Source data for histological analyses is provided in Supplementary Data 3. These results suggest that constitutively secreted, paracrine-delivered SGRP into the TME is superior to antibody-based CD47 blockade for persistent GAM modulation, potentiating antitumor responses in vivo.

Despite its efficacy, aEGFRvIII-SGRP CAR showed no signs of systemic toxicity, as evidenced by the absence of splenomegaly after two rounds of therapy (Supplementary Fig. 7a) and the average weight fluctuation throughout the experiment (Supplementary Fig. 7b). We observed a sharp drop in the weight of a few animals after the first aEGFRvIII-SGRP CAR treatment, suggestive of acute inflammatory events that resolve on their own after the second treatment (Supplementary Fig. 7b, right plot). Moreover, we examined CAR hematotoxicity parameters^35^: plasma IL6 and CRP, erythrocyte (RBC), platelet (PLT), and neutrophil (NEUT) counts, but found no indication of increased inflammation, anemia, thrombocytopenia or neutropenia (Supplementary Fig. 7c–e). The increase in neutrophil counts in the vehicle group is likely linked to a higher tumor burden in these animals from day 13 on (Supplementary Fig. 7e, bottom plot). Locally, we did not detect differences in brain myelination after therapy, an additional indication of safety (Supplementary Fig. 7f, g).

### SGRP improves GAM-mediated tumor phagocytosis

**Fig. 6:**
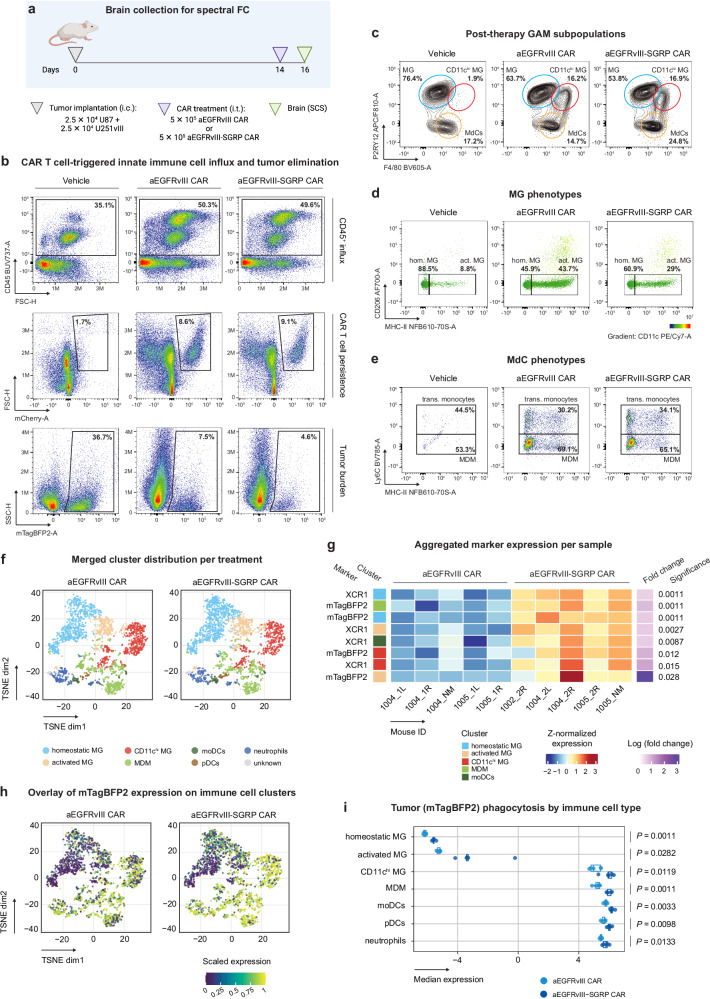
Anti-EGFRvIII-SGRP CAR treatment leads to increased myeloid cell-mediated GBM phagocytosis. **a** Brain tumor collection at an intermediate post-therapeutic time point for spectral FC (*n* = 5 per condition). Created in BioRender. Hutter, G. (2024) BioRender.com/f07x159. **b** Representative pseudocolor plots derived from individual mouse brains per condition displaying differential amounts of CD45^+^ cells (top row), mCherry^+^ CAR T cells (center row), and mTagBFP2^+^ tumor cells (bottom row). **c** Representative contour plots derived from individual mouse brains per condition with the relative amounts of GAM subpopulations. **d** MG-gated cells from (**c**) were subclassified into homeostatic MG or activated MG based on MHC-II expression. The color bar insert represents the MFI of the CD11c expression overlay. **e** MdC-gated cells from (**c**) were subclassified into transitory monocytes or MDMs based on Ly6C expression. **f** TSNE plots depicting 8 cell populations after merging and manual annotation of 22 populations generated by unbiased clustering of immune cell lineage marker expression. **g** Heatmap of aggregated marker expression per sample depicting significant changes between aEGFRvIII CAR and aEGFRvIII-SGRP CAR treatments. Significance cutoffs: false discovery rate = 0.05, log (fold change) = 0.5. **h** Overlay of mTagBFP2 expression on the TSNEs from (**f**), per therapeutic condition**. i** Scatter box plot of median mTagBFP2 tumor reporter expression in immune cell populations. Box plot displays pseudobulk expression levels of mTagBFP2 across conditions and populations. Statistical significance based on differential analysis is annotated with adjusted *P* values calculated using the diffcyt package with the diffcyt-DS-limma method. The boxes show the min and max values, excluding outliers. The boxes’ central line represents the median, with the 75th percentile at the upper bound, the 25th percentile at the lower bound, and the whiskers representing all samples lying within 1.5 times the interquartile range. Each dot represents one animal (*n* = 5 injected brain hemispheres per condition).

To further elucidate the effect of CAR + SGRP in the GBM TME, we performed a myeloid-targeted spectral FC analysis on tumor-bearing brain hemispheres collected 36 h after a single i.t. infusion of aEGFRvIII or aEGFRvIII-SGRP CAR T cells (Fig. 6a). The panel included human CD4, CD8a and EGFRvIII, and murine AXL, CD11b, CD11c, CD45, CD86, CD206, F4/80, Ly6C, Ly6G, MERTK, MHC-II, P2RY12, Siglec-H, TNF and XCR1 (Supplementary Table 4). Compared to vehicle controls, both CAR conditions triggered a rapid influx of CD45^+^ cells (Fig. 6b, top row pseudocolor plots). We confirmed the presence of mCherry^+^ CAR T cells in CAR treatment groups (Fig. 6b, center row pseudocolor plots) and found that both CAR treatments strongly reduced mTagBFP2^+^ tumor burden at this early time point (Fig. 6b, bottom row pseudocolor plots).

We then elaborated on the differences between GAM subsets. To differentiate MG from monocyte-derived cells (MdCs), we used P2RY12 in addition to CD45^int^CD11b^hi^ surface expression, which is highly specific for MG in homeostasis^36^ and the tumor context^37^. Notably, P2RY12 is downregulated during lipopolysaccharide (LPS)-induced MG activation^38^ and neurodegeneration in disease-associated microglia (DAM)^39,40^. Using conventional FC data analysis (Supplementary Fig. 8a), we identified a GAM population within which we distinguished three subpopulations (Fig. 6c): ‘MG’, ‘CD11c^hi^ MG’, and ‘MdCs’. Marker expression profiles of GAM subpopulations are shown in Supplementary Fig. 8b. Additionally, we subdivided MG into homeostatic MG (‘hom. MG’; F4/80^int^MHC-II^-^) or activated MG (‘act. MG’; F4/80^int^MHC-II^+^) (Fig. 6d). Moreover, we resolved the MdC population into Ly6C^+^ transitory monocytes (‘trans. monocytes’) or terminally differentiated, Ly6C^-^ ‘MDMs’ (Fig. 6e). We observed a significant CAR T cell-dependent increase in transitory monocytes and terminally differentiated MDMs, consistent with a recent report of monocyte dependence on IFNγ for monocyte-to-phagocyte transition^41^.

Multiple studies describe a CD11c^+^ MG subpopulation in healthy^42^ and diseased mouse brains^43,44^. Interestingly, we found that CD11c^hi^ MG displayed a surface signature similar to DAMs (Fig. 6d) and were functionally prophagocytic, as shown by the mTagBFP2-positivity of this population in CAR-treated brains (Supplementary Fig. 8b). These cells were also positive for CD206 (Supplementary Fig. 8b), a marker classically used to define M2-polarized MG and MDMs but recently associated with prophagocytic cells^45^. Furthermore, both CAR treatments induced the influx of plasmacytoid DCs (pDCs; CD45^+^CD11b^-^Ly6C^+^Siglec-H^+^) (Supplementary Fig. 8c).

To investigate potential differences in innate immune cells, we performed a FlowSOM multiparametric analysis (Supplementary Fig. 9a) that identified 22 metaclusters of differential lineage marker expression (Supplementary Fig. 9b). Subsequently, these clusters were merged into eight distinct cell populations (Supplementary Fig. 9b, c). While immune population frequencies did not differ between the CAR treatments (Fig. 6f and Supplementary Fig. 9d), differential abundance analysis showed significantly increased tumor cell phagocytosis (mTagBFP2) in MDM and MG in aEGFRvIII-SGRP CAR-treated brains (Fig. 6g–i). Furthermore, XCR1, a specific classical DC marker involved in antigen presentation^46^, was also increased in MG and MDMs in this condition (Fig. 6g). In contrast, we did not observe SGRP-mediated CAR T cell (mCherry) phagocytosis (Supplementary Fig. 9e). These data show that CAR T cells elicited the appearance of a DAM-like, CD11c^hi^ MG population that could be partially reprogrammed, along with other GAMs into a prophagocytic state by SGRP-secreting CAR T cells.

### Anti-EGFRvIII CARs outperform non-targeted CARs against patient GBM

**Fig. 7:**
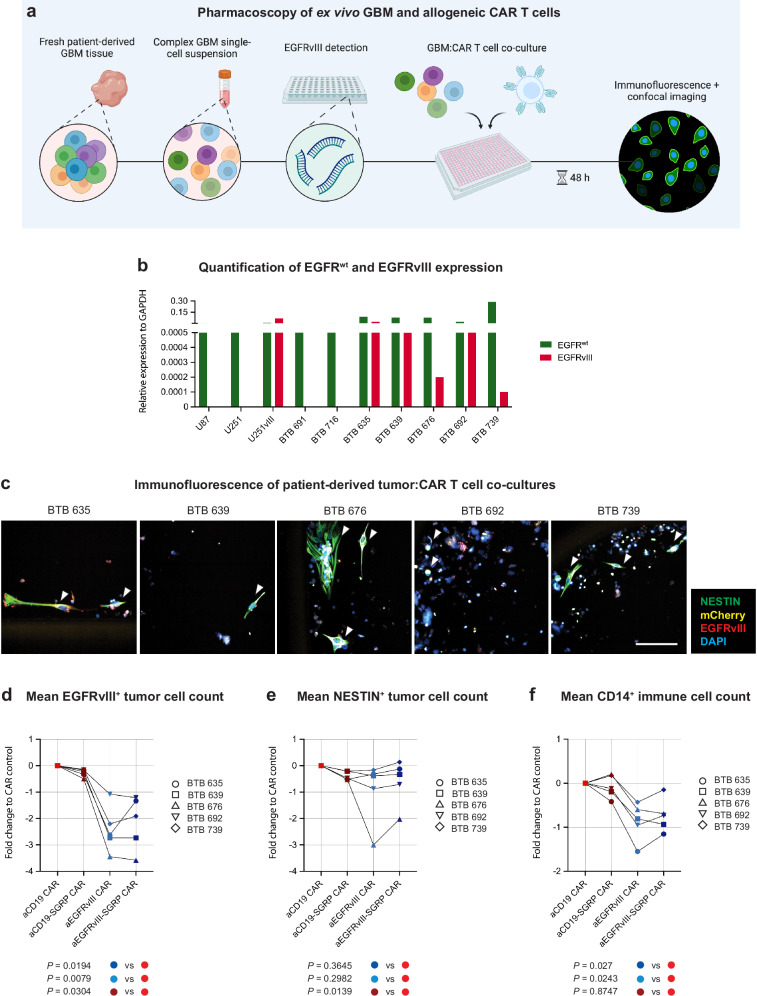
Pharmacoscopy analysis of GBM:CAR co-cultures reveals low functionality of GBM-associated immune cells in long-term stored patient-derived samples. **a** Overview of experimental setup. Frozen SCSs from patient-derived GBM (tumor center) were thawed and plated into 384 well plates at equal numbers. Beforehand, EGFRvIII status was determined on RNA extracts of matching samples. SCSs were co-cultured with aEGFRvIII, aEGFRvIII-SGRP, aCD19, or aCD19-SGRP CAR T cells for 48 h, fixed, stained, and imaged via confocal microscopy. Created in BioRender. Hutter, G. (2024) BioRender.com/p80p582. **b** Glyceraldehyde-3-phosphate dehydrogenase (GAPDH)-normalized expression of EGFR^wt^ and EGFRvIII detected by RT-qPCR in indicated patient-derived SCSs, showing EGFRvIII positivity in BTB 635, BTB 639, BTB 676, BTB 692 and BTB 739, and no EGFRvIII detection in BTB 691 and BTB 716. GBM cell lines were used as controls for EGFRvIII expression: EGFRvIII^-^ U87 and U251; EGFRvIII^+^ U251vIII. All GBM cell lines and patient-derived samples were positive for EGFR^wt^. **c** Exemplary IF readouts of co-cultures of CAR T cells with SCSs from EGFRvIII^+^ patient-derived GBM (*n* = 5) with white arrowheads showing viable NESTIN-stained tumor cells (green); Scale: 100 µm. Multiple micrographs were recorded from five replicate wells for each sample. **d** Mean EGFRvIII^+^ tumor cell count displayed as fold change relative to the aCD19 CAR control. **e** Mean NESTIN^+^ tumor cell count displayed as fold change relative to the aCD19 CAR control. **f** Mean CD14^+^ tumor cell count displayed as fold change relative to the aCD19 CAR control. **d**–**f** Statistics were calculated using a two-sided one-way ANOVA with Dunnett’s multiple comparisons. The experiment was performed once due to the scarcity of human material, particularly EGFRvIII^+^ GBM samples. Source data are provided as a Source Data file and Supplementary Data 4.

To test the efficacy of aEGFRvIII-SGRP CAR in a translational setting, we co-cultured CAR T cells with patient-derived GBM single-cell suspensions (SCSs) and performed a high-content imaging analysis in 5 patients screened for EGFRvIII-positivity (Fig. 7a, b; patient data in Supplementary Table 6). We did not match CAR T and patient-derived SCSs for logistical reasons, raising the potential of alloimmunological phenomena. The readout included human CD14 (myeloid cells), EGFRvIII (antigen-positive tumor cells), and NESTIN (pan-GBM cell marker) IF stainings (Fig. 7c). As expected, EGFRvIII-specific CARs significantly reduced the number of EGFRvIII^+^ cells compared to CD19-specific control CARs but we did not observe a clear benefit of aEGFRvIII-SGRP CAR (Fig. 7d). Moreover, aCD19-SGRP CARs induced moderate antigen-independent killing of both EGFRvIII^+^ and NESTIN^+^ tumor cells, presumably due to a potential SGRP-mediated effect (Fig. 7e). CD14^+^ myeloid cell numbers were reduced in aEGFRvIII CAR:GBM co-cultures, possibly due to a short-term bystander effect of CAR-mediated cytokine release (Fig. 7f). We hypothesize that using long-term-stored SCSs led to low viability and functional deficits in the myeloid cell compartment, which may explain the lack of significantly different outcomes of aEGFRvIII and aEGFRvIII-SGRP CAR therapy.

### Anti-CD19-SGRP CAR is the superior treatment in lymphoma xenografts

**Fig. 8:**
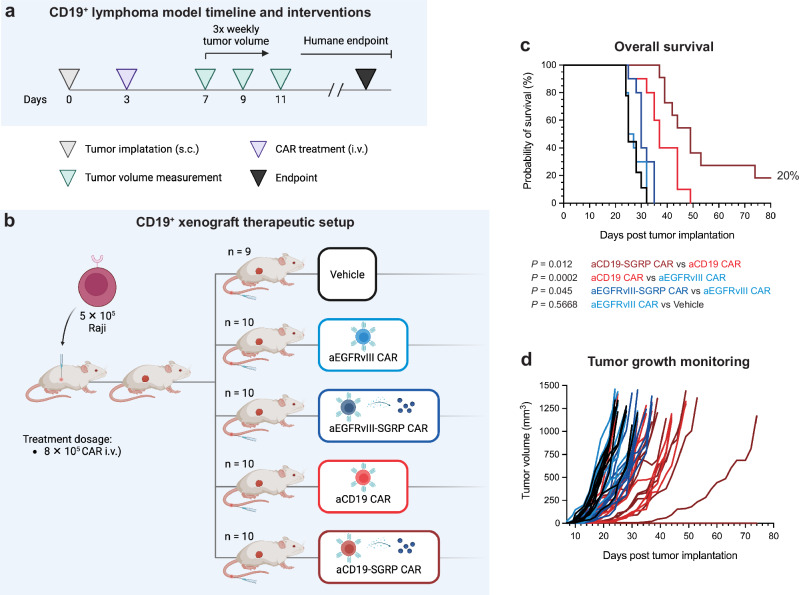
Systemic anti-CD19-SGRP CAR T cell therapy delays tumor growth and improves survival in a CD19^+^ lymphoma xenograft model. **a** Experimental setup and timeline of interventions of peripheral CD19^+^ lymphoma model. Three days after tumor implantation in the right flank, mice were treated with a single i.v. infusion of CAR T cells, followed by 3 times weekly tumor volume assessment and clinical scoring. **b** Overview of experimental groups/therapeutic conditions and treatment dosages (vehicle *n* = 9; others *n* = 10). CAR T cell dose: 8 × 10^5^ cells delivered i.v. **a**, **b** Created in BioRender. Hutter, G. (2024) BioRender.com/f99s127. **c** Kaplan–Meier plot of overall survival (in days). Two-sided log-rank tests were used to compare treatment/control groups. **d** Tumor volume measurements of individual mice (in days). **c**, **d** The data were pooled from two independent experiments. Source data are provided as a Source Data file.

To demonstrate the potential benefit of iTME-targeted secretion of SGRP in another tumor model, we assessed aCD19-SGRP CAR efficacy in CD19^+^ lymphoma xenografts. We considered a systemic approach mirroring CAR T cell treatments currently used against CD19^+^ malignancies. Raji cells were injected in the right flank of NSG mice, followed by systemic CAR T cell infusion on day 3 post-tumor implantation (Fig. 8a). The therapeutic setup consisted of a single injection of 8 × 10^5^ target-specific CAR T cells (aCD19 CAR or aCD19-SGRP CAR) or -unspecific CAR T cells (aEGFRvIII CAR or aEGFRvIII-SGRP CAR) as controls (Fig. 8b). All aCD19 CAR-treated animals had a significant survival benefit compared to either vehicle or target-unspecific CAR controls (Fig. 8c). Strikingly, aCD19-SGRP CAR treatment resulted in the most prolonged survival benefit (20%) with one cured animal (Fig. 8c, d). Thus, the contribution of SGRP-mediated innate immune modulation is relevant in solid cancers other than GBM, demonstrating a broad application in vastly different solid tumor types resistant to conventional CAR T cell therapy.

## Discussion

**Fig. 9:**
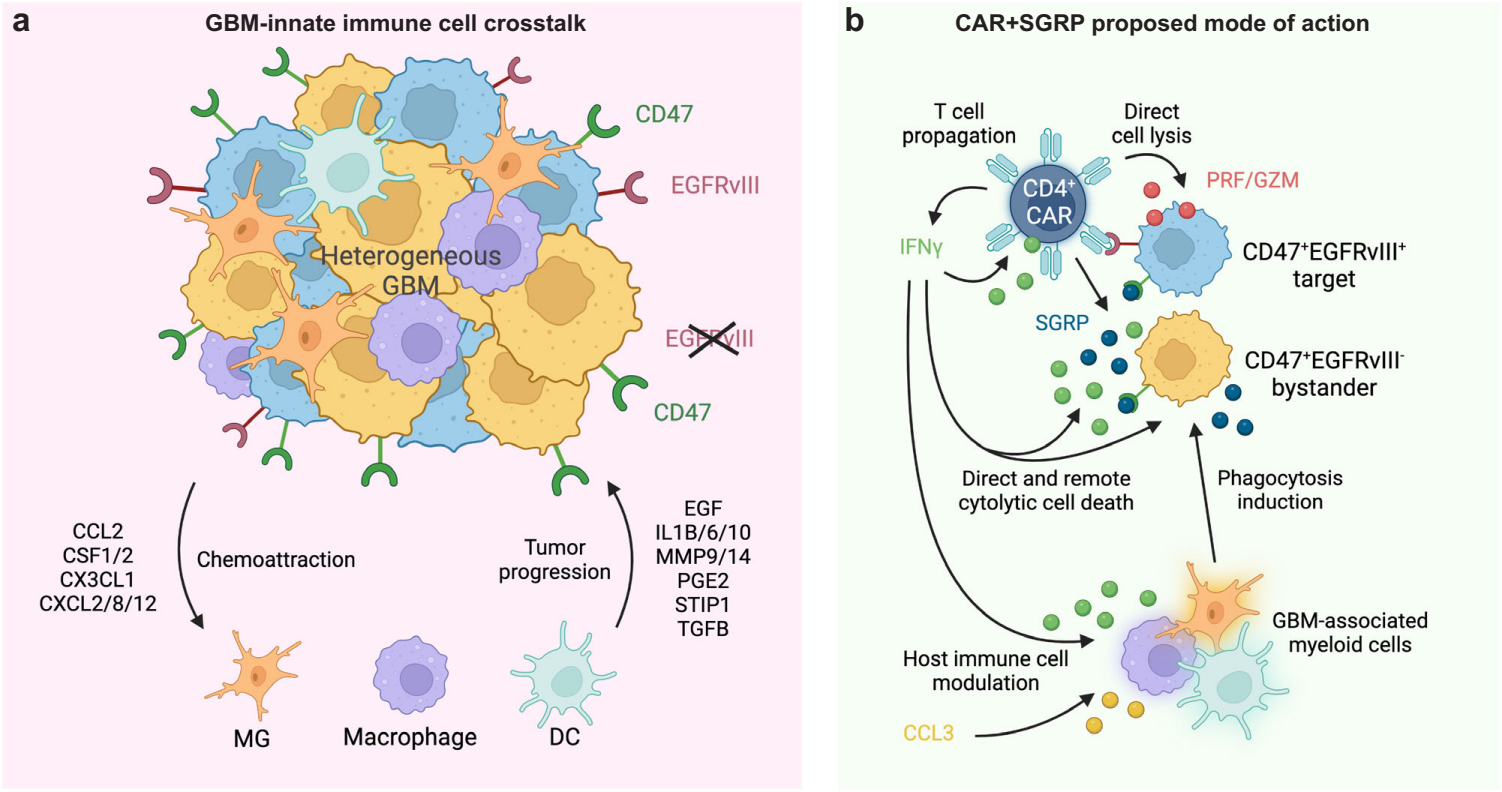
Bypassing tumor immune suppression and antigen escape with CAR T cells that secrete a paracrine myeloid cell modulator. **a** The EGFRvIII expression pattern in GBM is heterogeneous, the leading cause of antigen escape and failure of conventional EGFRvIII-targeted CAR T cell therapy (Fig. 1a). CD47 is a phagocytosis immune checkpoint typically overexpressed by GBM cells to evade recognition and targeting by phagocytes. The GBM immune response is hampered by infiltrating innate immune cells recruited by the tumor, aiding its progression by releasing trophic factors, cytokines, matrix peptidases, and inflammatory molecules. CCL2: C-C motif chemokine ligand 2; CSF1: colony stimulating factor 1; CSF2: colony stimulating factor 2; CX3CL1: C-X3-C motif chemokine ligand 1; CXCL2: C-X-C motif chemokine ligand 2; CXCL8: C-X-C motif chemokine ligand 8; CXCL12: C-X-C motif chemokine ligand 12; EGF: epidermal growth factor; IL1B: interleukin 1 beta; IL6: interleukin 6; IL10: interleukin 10; MMP9: matrix metallopeptidase 9; MMP14: matrix metallopeptidase 14; PGE2: prostaglandin E2; STIP1: stress induced phosphoprotein 1; TGFB: transforming growth factor beta. **b** Schematic overview of the proposed mode of action of SGRP-secreting CAR T cells with the primary mechanisms identified in our study. CAR T cell products predominantly consisting of CD4^+^ T cells eliminate target-positive tumor cells directly via perforin/granzyme and IFNγ. High local IFNγ and CCL3 levels induce host immune cell activation and chemotaxis. Bystander tumor cells are preferentially targeted by IFNγ-dependent remote cytolytic cell death or marked for phagocytosis by SGRP secreted by nearby target-engaged CAR T cells. PRF: perforin; GZM: granzyme. **a**, **b** Created in BioRender. Hutter, G. (2024) BioRender.com/l24e295.

Myeloid suppression and tumor heterogeneity are critical factors for CAR T cell therapy failure in GBM^8,27,47^ (Fig. 9a). Here, we show the superior efficacy of SGRP-secreting CAR T cells in treating EGFRvIII-mosaic GBM and CD19^+^ lymphoma, offering an approach to target solid tumors heavily infiltrated by immunosuppressive innate immune cells. Recent preclinical studies combining CAR T cells and SIRPα-based CD47 blockade show promise in hematological cancers^25,48,49^. However, this approach in solid tumors like GBM remains understudied despite GBM’s iTME being predominantly composed of actionable GAMs.

EGFRvIII, one of the few known tumor-specific antigens in GBM, remains an attractive CAR T cell target due to its clinically demonstrated safety^8,27^. Recent studies suggest that up to 70% of patients with GBM express EGFRvIII^50^. Yet, antigen escape is a known resistance mechanism to EGFRvIII-targeted therapies^8^.

We confirmed SGRP presence in aEGFRvIII-SGRP CAR T cell lysates and culture media. However, functional in vitro assays were technically challenging to adapt to CAR-mediated secretion models. We hypothesize that SGRP effects may not be observed in standard phagocytosis assays due to the low CAR T cell density in this experimental setup, leading to reduced SGRP availability compared to high-dose CD47 blockade by antibodies. Furthermore, MDM differentiation into a naïve M0-like phenotype in vitro may also reduce their phagocytic potential compared to MDMs differentiated in a GBM setting.

Our EGFRvIII-mosaic GBM model, while valuable for mechanistic insights, does not fully capture the clonal dynamics, tumor evolution, or complex cell interactions characteristic of GBM^51^. This reductionistic approach allowed us to assess therapeutic responses in a controlled environment but should be considered when translating these findings to more clinically relevant settings. In our GBM mouse models, CAR T cells were deployed intratumorally since locoregional application is superior to systemic infusion in brain tumors in terms of efficacy, avoiding preconditioning lymphodepletion and reducing systemic side effects^52^. However, CAR T cells with constitutive SGRP secretion, given its high affinity to CD47, might risk off-tumor toxicity. While absent in our GBM model, potential SGRP-mediated neuro- and hematologic toxicity^21^ should be carefully monitored in future studies. Nonetheless, combinatorial CAR/immunomodulatory strategies, such as EGFRvIII-specific CAR T cells with local IL-12^53^ or with concomitant bispecific T cell engagers (BiTEs)^54^ did not report systemic toxicity in vivo.

Interestingly, our study’s T cell production preferentially yielded CD4^+^ CAR T cells, for which diverse cytotoxic mechanisms have been described. Although these cells directly engage target-positive tumor cells via perforin/granzyme, a recent study showed that their cytotoxic effect is primarily IFNγ-dependent^55^. In addition to direct antitumor activity, CD4^+^ CAR T cells may induce IFNγ-dependent remote cytolytic tumor cell death and promote host immune cell activation^55,56^, further complementing phagocytosis-potentiating therapeutic approaches like the one presented here (Fig. 9b). Indeed, we found high levels of IFNγ secretion by both aEGFRvIII and aEGFRvIII-SGRP CARs in vitro and in vivo but only SGRP-armored CARs were able to cope with a high tumor load of target-negative cells in vivo. Other studies have shown that CD4^+^ CAR T cells display superior proliferation and antitumor function compared to CD8^+^ cells in GBM, leukemia, and pleural tumor models^57–59^. Moreover, preclinical and clinical reports have identified long-term persisting CD4^+^ CAR T cells following anti-CD19 CAR T cell therapy^59,60^.

We also found significantly increased CCL3 levels in the plasma of aEGFRvIII-SGRP CAR-treated animals, indicating a beneficial antitumor response^61,62^. However, blockade of CCL3 during aEGFRvIII-SGRP CAR treatment did not reduce therapeutic efficacy, suggesting that CCL3 is not an essential therapeutic mediator in this experimental context. This outcome may be attributed to the upregulation of compensatory molecules, such as CCL4 and CCL5 or others, known to act redundantly with CCL3 and maintain immune cell recruitment^61^, thereby sustaining this therapeutic effect independent of CCL3. Multiple mechanisms likely contribute to the improved efficacy of SGRP-secreting CAR T cells, and potential cooperation between SGRP, IFNγ, and CCL3-like chemoattractants might be crucial to eliminate bystander antigen-negative tumor cells (Fig. 9b). Further work will be needed to evaluate the relative contributions of the multiple mechanisms described here.

SGRP-secreting target-unspecific CAR T cells in GBM and lymphoma xenografts produced minor therapeutic effects, suggesting that tumor antigen-mediated persistence of effector cells in the TME is crucial for the efficacy observed in target-specific conditions. Direct local application of SGRP (e.g., via osmotic pumps) could provide additional insights into its effects on the TME.

This work highlights the importance of sustained, local paracrine CD47 blockade to relieve GAM-mediated immunosuppression in GBM, which is unachievable through systemic or local episodic application of CD47-blocking antibodies. This is an example of effective targeting of heterogeneous GBM by adoptive T cells with concurrent local paracrine GAM modulation in a single therapeutic modality. Potentiating CAR T cells with a secretable SGRP to target other tumor antigens might be a valuable tool for improving CAR T cell therapy in solid tumors characterized by high antigen heterogeneity and immunosuppressive myeloid compartments.

## Methods

### Ethics and study design

Peripheral blood leukocytes from volunteer human HDs were obtained from the Blood Donation Center of the University Hospital Basel, Switzerland, after written informed consent was obtained from all participants before blood collection. Donors were anonymous, and no data on sex or age was collected. Freshly resected primary tumor tissue samples from patients with a pathology-confirmed GBM diagnosis were obtained from the Neurosurgical Clinic of the University Hospital Basel, Switzerland, following the Swiss Human Research Act and Institutional Ethics Commission (EKNZ 02019-02358). All patients with GBM were treatment-naïve at the time of tissue collection. The study followed the ethical principles of the Declaration of Helsinki, regulatory requirements, and the Code of Good Clinical Practice. All patients gave written informed consent for tumor biopsy collection and signed a declaration enabling the use of their biopsy specimens in scientific research (Req-2019-00553) with all identifying information removed. Study participants were not compensated. The study included seven patients with GBM, six males and one female, with an average age of 67.7 years. Participant sex was reported as assigned in legal documents. The clinical data of patients who participated in this study is summarized in Supplementary Table 6.

This research complies with all relevant ethical research regulations using animals from the Veterinary Office of the Health Department of Canton Basel-Stadt. Animal handling, surveillance, and experimentation were performed according to the Swiss Federal Veterinary Office (SFVO) guidelines and the Cantonal Veterinary Office (CVO) of Basel-Stadt, Switzerland. GBM model experiments were executed under licenses #2929_31795 and #3176_35274, and lymphoma model experiments under license #3036_34231. Animals were maintained at the local animal facility in pathogen-free, ventilated HEPA-filtered cages under stable housing conditions of 45–65% humidity, a temperature of 21–25 °C, and a gradual light cycle from 7 am to 5 pm. Animals were provided standard food and water without restrictions. NOD.Cg-Prkdc^scid^ Il2rg/SzJ (NSG) mice with identifier RRID:IMSR_JAX:005557 were obtained from in-house breedings or externally (Janvier Labs, France) under protocols approved by the SFVO and CVO of Basel-Stadt. Co-housed animals were assigned to treatment or control groups using a randomized approach whenever possible. The study protocol placed no specific restriction on maximal tumor size in orthotopic GBM models as long as animals were euthanized upon reaching the humane endpoint, which included a significant reduction of locomotion, significant weight loss, and mild-to-severe neurologic symptoms, according to approved scoring criteria detailed in Supplementary Table 5. In subcutaneous (s.c.) lymphoma models, the study protocol restricted maximal tumor volume to 1500 mm^3^. This limit was not breached for any animal in the study. Sex was not explicitly considered in our study design for human or animal experiments due to GBM affecting people of the male and female sex.

### SGRP engineering

To generate SGRP, nineAA substitutions were made to the endogenous human SIRPγ binding domain (hSIRPγ-V1) AA sequence, as previously described by Ring and colleagues^26^ (Supplementary Fig. 1a). A human IL-2 signal peptide (IL2sig) sequence was added to the N-terminus of SGRP to allow its secretion by T cells to the extracellular space (Fig. 1c). The SGRP AA sequence was codon-optimized using GenSmart Codon Optimization (GenScript Biotech, USA) with ‘Human T-cell’ set as the expression host organism. The resulting optimized AA sequence was then reverse-translated into a nucleotide (nt) sequence. AA and nt sequences are listed in Supplementary Table 1.

### Protein interaction modeling

Protein structures of SGRP, human SIRPα binding domain (hSIRPα-V1), murine SIRPα binding domain (mSIRPα-V1), and human CD47 (hCD47) were predicted by AlphaFold^63,64^, using the source code, trained weights and inference script available under an open-source license [https://github.com/deepmind/alphafold]. AA sequences of SGRP, hSIRPα-V1, mSIRPα-V1, and hCD47 were entered and folded using the multimer model. Sequences were superimposed against a genetic database to generate multiple sequence alignment statistics. Predictions ran on ‘relax mode without GPU’ and generated 3D interaction models for SGRP and hCD47, hSIRPα-V1 and hCD47, or mSIRPα-V1 and hCD47 (Supplementary Fig. 1b). Predicted local distance difference tests (pLDDT) were calculated to evaluate local distance differences of all atoms in each model and validate stereochemical plausibility (data not shown). All AA and nt sequences are listed in Supplementary Table 1.

### CAR construct and lentiviral expression vector design

For human T cell transduction, replication-defective lentiviruses were produced using a second-generation lentiviral system with transfer plasmids encoding a 3C10.BBz^27,65^ (anti-EGFRvIII) or FMC63.BBz^28,66^ (anti-CD19) CAR, an mCherry fluorescence reporter protein, and, in some iterations, SGRP^24,26^. The CAR structure consisted of a CD8α leader, a single-chain variable fragment (scFv), CD8α hinge and transmembrane domains, a 4-1BB costimulatory domain, and a CD3ζ signaling domain. Transgene expression was driven by the *EF1A* promoter, and polyprotein sequences were cleaved by T2A or P2A peptides. All sequences were assembled with Vector Design Studio (VectorBuilder, USA). The vectors included an ampicillin resistance gene to positively select transformed *Escherichia coli*. The lentiviral plasmids were purchased as bacterial glycerol stocks from VectorBuilder (VectorBuilder, USA). CAR constructs and lentiviral vector maps are illustrated in Fig. 1d and Supplementary Fig. 1c, respectively. CAR construct and CAR domain sequences are listed in Supplementary Table 2.

### Lentivirus production

Plasmid DNA was extracted with a QIAprep Spin Miniprep kit (#27104, QIAGEN, Netherlands) from bacterial cultures grown overnight in the presence of 100 µg/mL ampicillin (#A5354, Sigma-Aldrich, USA). Lentiviral particles were generated by co-delivery of a transfer plasmid, a plasmid encoding a VSV-G envelope (pMD2.G; #12259, Addgene, USA), and an empty backbone plasmid (psPAX2; #12260, Addgene, USA) into HEK293T cells. Cells were maintained in DMEM (#11995065, Gibco, USA) supplemented with 10% inactivated fetal bovine serum (FBS; #P30-3302, PAN-Biotech, Germany), 1% pen strep (#15140-122, Gibco, USA) and 2 mM GlutaMAX-I (#35050-038, Gibco, USA). They were cultured as adherent monolayers at 37 °C in a 5% CO_2_ atmosphere and regularly subcultured when reaching ~70–80% confluence. For the transfections, 5 × 10^5^ HEK293T cells were seeded per well in a 6-well plate and rested for 24 h. Growth media were replaced with antibiotic-free growth media and DNA plasmids were complexed with polyethyleneimine (PEI; #408727, Sigma-Aldrich, USA) for 15 min and added dropwise to the cells, followed by a 48 h incubation. The viral supernatants were collected and cleared from cells and debris by centrifugation at 500 × *g* for 10 min, followed by filtration through a 0.45 µm polyethersulfone filter (#SLHPM33RS, MilliporeSigma, USA). Virus particles were precipitated with Lenti-X Concentrator (#631232, Takara Bio, Japan), suspended in phosphate buffer saline (PBS; #D8537, Sigma-Aldrich, USA), quantified with Lenti-X GoStix Plus (#631280, Takara Bio, Japan), aliquoted and stored at −80 °C.

### Healthy donor T cell and monocyte isolation

Peripheral blood mononuclear cells (PBMCs) were isolated with Ficoll Paque-PLUS (#GE17-1440-02, Cytiva, Germany) and density centrifugation. After up to 2 rounds of ACK-lysis (#A10492-01, Gibco, USA) to remove erythrocytes, PBMCs were washed with PBS. CD3^+^ T cells were magnetically separated by negative selection with a Human Pan T cell isolation kit (#130-096-535, Miltenyi Biotec, Germany) and stored long-term in Bambanker serum-free cell freezing medium (#BB01, GC Lymphotec, Japan) in liquid nitrogen (LN_2_). CD14^+^ cells used in phagocytosis assays were magnetically separated by positive selection using Human CD14 MicroBeads (#130-050-201, Miltenyi Biotec, Germany) and stored in Bambanker serum-free cell freezing medium in LN_2_.

### CAR T cell production

CAR T cells were produced from HD PBMCs, depleted for non-T cells, and frozen in batches. CAR T cells were freshly made from frozen HD T cell batches for every experiment. Upon thawing, T cells were washed with PBS and rested in X-VIVO 15 (#BE02-060F, Lonza, Switzerland) at a density of 1 × 10^6^ cells per mL at 37 °C in a 5% CO_2_ atmosphere. After 24 h, the T cells were activated in X-VIVO 15 containing 150 μ/mL of human IL-2 (#Ro 23-6019, Roche, Switzerland), 10 ng/mL of recombinant IL-7 (#200-07, PeproTech, USA), 10 ng/mL of recombinant IL-15 (#200-15, PeproTech, USA), 20 ng/mL of recombinant IL-21 (#200-21, PeproTech, USA) and Dynabeads human T-activator CD3/CD28 (aCD3/CD28; #11131D, Gibco, USA) in a 1:1 cell-bead ratio^67^. After 48 h, aCD3/CD28 beads were magnetically removed, and T cells were resuspended in X-VIVO 15 with 5 µg/mL polybrene (#TR-1003-G, Sigma-Aldrich, USA) at a density of 3 × 10^6^ cells per mL. Lentiviral suspensions were added to the T cells at different multiplicity of infection (MOI) ratios and spinfected at 1200 × *g*, at 30 °C for 90 min. After spinfection, the T cells were washed and maintained at a density of 1 × 10^6^ cells per mL in X-VIVO 15 containing 500 μ/mL IL-2 for 5–7 days. After this post-transduction cell expansion, T cells were sorted for mCherry expression using a BD FACSMelody Cell Sorter (BD Biosciences, USA). After sorting, the CAR T cell cultures were expanded in X-VIVO 15 containing 500 μ/mL IL-2 and kept at a density of 1 × 10^6^ cells per mL by adjusting the cell density every 2–3 days based on automated cell counting with a LUNA-FL Dual Fluorescence Cell Counter (Logos Biosystems, South Korea) for 5–10 days until used in downstream assays. A schematic of CAR T cell production from HD T cells is illustrated in Fig. 1e.

### CAR-biotinylated target protein binding assay

CAR T cell viability and count were assessed by Trypan blue exclusion. Cells were washed with PBS and seeded into a 96-well plate at a density of 2 × 10^5^ live cells per well. Cells were immediately stained with a Zombie NIR Viability kit (#423106, BioLegend, USA) diluted 1:5000 in PBS for 20 min in the dark at RT. After viability staining, the cells were washed with autoMACS Running Buffer (#130-091-221, Miltenyi Biotec, Germany) and then resuspended in 100 µL per well of 10 µg/mL dilutions of biotinylated CD19, EGFR, or EGFRvIII proteins (#CD9-H82E9, #EGR-H82E3, and #EGR-H82E0, ACROBiosystems, USA) for 1 h in the dark at 4 °C. After CAR-target exposure, the cells were washed with autoMACS Running Buffer and stained with 100 µL per well of FITC Streptavidin (SA; #405202, BioLegend, USA) diluted 1:50 in autoMACS Running Buffer for 1 h in the dark at 4 °C. Afterward, the cells were washed three times with autoMACS Running Buffer and resuspended in 100 µL of autoMACS Running Buffer. Samples were acquired with a CytoFLEX Flow Cytometer (Beckman Coulter, USA), and data were analyzed using FlowJo v10 Software (BD Biosciences, USA).

### Real-time quantitative PCR

The total RNA contents of ~1 × 10^6^ cells from human CAR T cells were extracted with TRIzol Reagent (#15596026, Invitrogen, USA) and an AllPrep DNA/RNA/Protein Mini kit (#80004, QIAGEN, USA). cDNA was synthesized with an iScript cDNA Synthesis kit (#1708891, Bio-Rad, USA) using 500 ng of input RNA in a 20 µL reaction. The reaction included priming for 5 min at 25 °C, reverse transcription for 20 min at 46 °C, and inactivation for 1 min at 95 °C. RT-qPCR reactions were performed with a SsoFast EvaGreen supermix (#172-5201, Bio-Rad, USA) using 2 µL of input cDNA in a 20 µL reaction. The total RNA contents of ~2 × 10^6^ cells from culture-dissociated tumor cells or human GBM single-cell suspensions were extracted with TRIzol Reagent and a Direct-zol RNA Miniprep Plus kit (#R2070, Zymo Research, USA). cDNA was synthesized with a SuperScript VILO cDNA Synthesis kit (#11754-050, ThermoFisher Scientific, USA) using 20 ng of input RNA in a 20 µL reaction. The reaction included priming for 10 min at 25 °C, reverse transcription for 60 min at 42 °C, and inactivation for 5 min at 85 °C. RT-qPCR reactions were performed with a SsoFast EvaGreen supermix using 2 µL of input cDNA in a 20 µL reaction. RT-qPCR primers were used at a final concentration of 1 µM and are listed in Supplementary Table 7. Thermal cycling was performed using a CFX96 Touch Real-Time PCR Detection System (Bio-Rad). It included an initial step of enzyme activation for 30 s at 95 °C, followed by 40 cycles of denaturation for 5 s at 95 °C and annealing/extension for 5 s at 60 °C. Relative mCherry, SGRP, EGFR, and EGFRvIII expression were calculated with the ΔΔCt method, using TBP or GAPDH Ct to normalize signal expression.

### Mass spectrometry

#### Supernatant isolation

Expanded CAR T cell cultures from two donors were rested in X VIVO medium without additional supplements for 24 h. The following day, cell viability and count were assessed by Trypan blue exclusion, after which cells were washed in PBS, resuspended in RPMI at a density of 1 × 10^6^ live cells per mL, and cultures were incubated for another 24 h at 37 °C. Afterward, cultures were centrifuged at 300 × *g* for 5 min, and supernatants were collected, passed through 0.22 µm filters, and stored at −20 °C. aEGFRvIII-SGRP CAR culture supernatants were compared to control aEGFRvIII CAR culture supernatants. A total of 12 samples were analyzed: 2 biological replicates (CAR T cells from HD6 and HD7) and 3 technical replicates.

#### Protein digestion

Supernatant samples were TCA-precipitated following the procedure: one volume of TCA was added to every 4 volumes of sample, mixed by vortexing, and incubated for 10 min at 4 °C followed by precipitate collection by centrifugation for 5 min at 23,000 × *g*. The supernatant was discarded, and the pellet was washed two times with acetone precooled to −20 °C. The washed pellets were incubated in the open tube for 2 min at RT to allow residual acetone to evaporate. The pellets were resuspended in 2 M guanidine hydrochloride (GUA), 10 mM TCEP, and 100 mM Ammonium Bicarbonate (AmBIC; pH = 8.5) by sonication. The samples were incubated for 10 min at 95 °C, let to cool down to RT, followed by the addition of chloroacetamide in a final concentration of 15 mM. After an incubation of 30 min at 37 °C, the samples were diluted with 100 mM AmBIC to achieve a final concentration of 0.5 M GUA. Sequencing-grade modified trypsin (1/50, w/w; #V5280, Promega, USA) was added, and the proteins were digested for 12 h at 37 °C with gentle agitation. Digests were acidified (pH < 3) using TFA and desalted using C18 spin columns (#74-4101, Harvard Apparatus, USA) according to the manufacturer’s instructions. Peptides were dried under vacuum and stored at −20 °C.

#### Peptide mixture loading

A total of 1 µg of peptides were subjected to LC-MS/MS analysis using a Q Exactive Plus Mass Spectrometer (ThermoFisher Scientific, USA) fitted with an EASY-nLC 1000 (ThermoFisher Scientific, USA) and a custom-made column heater set to 60 °C. Peptides were resolved using RP-HPLC columns (75 μm × 30 cm) packed in-house with C18 resin (ReproSil-Pur C18–AQ, 1.9 μm resin; Dr. Maisch, Germany) at a flow rate of 0.2 μL per min. The following gradient was used for peptide separation: from 5% B to 10% B over 5 min to 35% B over 40 min to 50% B over 15 min to 95% B over 2 min followed by 18 min at 95% B. Buffer A was 0.1% formic acid in water and buffer B was 80% acetonitrile, 0.1% formic acid in water.

#### Acquisition

The mass spectrometer was operated in DDA mode with a total cycle time of ~1 s. For MS1, 3e6 ions were accumulated in the Orbitrap over a maximum time of 100 ms and scanned at a resolution of 70,000 FWHM at 200 *m*/*z*. Each MS1 scan was followed by high-collision-dissociation (HCD) of the 10 most abundant precursor ions with dynamic exclusion set to 45 s. MS2 scans were acquired at a target setting of 1e5 ions, a maximum accumulation time of 100 ms, and a resolution of 35,000 FWHM at 200 *m*/*z*. Singly charged ions, ions with charge state ≥6 and ions with unassigned charge state were excluded from triggering MS2 events. The normalized collision energy was set to 27%; the mass isolation window was set to 1.4 *m*/*z*, and one microscope was acquired for each spectrum.

#### Data preprocessing and analysis

Raw data were imported into Progenesis QI v2.0 Software (Nonlinear Dynamics, UK), which extracted peptide precursor ion intensities across all samples with the default parameters. The generated mgf file was searched using MASCOT against a human database (consisting of 41094 forward and reverse protein sequences downloaded from Uniprot in April 2020), a manually entered recombinant SGRP AA sequence as well as 392 commonly observed contaminants using the following search criteria: full tryptic specificity was required (cleavage after lysine or arginine residues, unless followed by proline); 3 missed cleavages were allowed; carbamidomethylation (C) was set as fixed modification; oxidation (M) and acetyl (Protein N-term) were applied as variable modifications; mass tolerance of 10 ppm (precursor) and 0.6 Da (fragments). The database search results were filtered using the ion score to set the false discovery rate to 1% on the peptide and protein level, respectively, based on the number of reverse protein sequence hits in the dataset. Quantitative analysis results from label-free quantification were processed using the SafeQuant R package v.2.3.2 [https://github.com/eahrne/SafeQuant/]^68^ to obtain peptide relative abundances. This analysis included global data normalization by equalizing the total peak/reporter areas across all LC-MS runs, data imputation using the knn algorithm, summation of peak areas per protein and LC-MS/MS run, followed by calculation of peptide abundance ratios. Only isoform-specific peptide ion signals were considered for quantification. To meet additional assumptions (normality and homoscedasticity) underlying the use of linear regression models and *t*-tests, MS-intensity signals were transformed from the linear to the log scale. The summarized peptide expression values were used to test for differentially abundant peptides between conditions. Here, empirical Bayes-moderated *t*-tests were applied, as implemented in the R/Bioconductor limma package [http://bioconductor.org/packages/release/bioc/html/limma.html]. The resulting per protein and condition comparison *p*-values were adjusted for multiple testing using the Benjamini–Hochberg method. Differential expression analysis compared aEGFRvIII-SGRP CAR and aEGFRvIII CAR samples from 2 HDs. A complete list of detected proteins and their relative abundance is provided as Supplementary Data 1.

#### Cell lines and cell culture

BS153, U87, U87vIII, U251, and U251vIII are human glioma cell lines. BS153 cells were maintained in DMEM (#10938025, Gibco, USA) supplemented with 10% inactivated FBS, 1% pen strep, 2 mM GlutaMAX-I and 1 mM sodium pyruvate (#S8636, Sigma-Aldrich, USA). U87, U87vIII, U251, and U251vIII cells were maintained in MEM (#M4655, Sigma-Aldrich, USA) supplemented with 10% inactivated FBS, 1% pen strep, 1X MEM NEAA (#11140-035, Gibco, USA), 2 mM GlutaMAX-I and 1 mM sodium pyruvate. Raji is a lymphoma cell line cultured in DMEM supplemented with 10% inactivated FBS, 1X MEM NEAA, and 1% pen strep. NSC197 is a NSC line cultured in Human NeuroCult NS-A Basal Medium (#05750, STEMCELL Technologies, Canada) supplemented with 50 mL Human NeuroCult Proliferation supplement (#05753, STEMCELL Technologies, Canada), 20 ng/mL Recombinant Human EGF Protein, CF (#236-EG, R&D Systems, USA), 10 ng/mL Recombinant Human FGF-basic (#100-18B, Peprotech, USA), 0.002% heparin (w/v; #07980, STEMCELL Technologies, Canada), and 1X antibiotic-antimycotic (#A5955, Sigma-Aldrich, USA). All cell lines were maintained at 37 °C in a 5% CO_2_ atmosphere and regularly subcultured when reaching ~70–80% confluence. Cell cultures were routinely tested for mycoplasma contamination using a MycoAlert PLUS Mycoplasma Detection kit (#LT07-710, Lonza, Switzerland). Raji cells were cultured in suspension, whereas all others were cultured as adherent monolayers. All cell lines were obtained from collaborators.

#### GBM cell line lentiviral transduction

Parental EGFRvIII-negative U87 and U251 cell lines were transduced with a pmp71 lentiviral vector encoding a full-length EGFRvIII to generate stable EGFRvIII-expressing U87vIII and U251vIII cell lines, respectively. For in vitro cytotoxicity assays, BS153, U251, and U251vIII cells were transduced with an Incucyte NucLight Green lentivirus (#4624, Sartorius, Germany) to express a nuclear-restricted EGFP (nEGFP) fluorescence viability reporter protein. For in vivo tumor monitoring by BLi and fluorescence labeling of tumor cells in downstream assays, U87 cells were transduced with a Luc2-mTagBFP2 lentivirus, and U251vIII cells were transduced with an iRFP713-NLuc lentivirus. EGFRvIII-overexpression and fluorescence/bioluminescence vector sequences are listed in Supplementary Table 3. For lentiviral transduction, tumor cells were seeded at 1 × 10^5^ cells per well of a 24-well plate and rested for 24 h. Growth media were replaced with antibiotic-free growth media containing 8 µg/mL polybrene. Lentiviral suspensions were added to the cells at different MOIs and incubated for 6 h at 37 °C. Afterward, transduction media were replaced with fresh growth media, and the cells were expanded for 1–2 weeks. Cells expressing the relevant surface receptor or fluorescence protein were sorted using a BD FACSMelody or a BD FACSAria SORP Cell Sorter (BD Biosciences, USA). Luc2 and NLuc expression was confirmed by exposing cells seeded in the wells of a flat-bottom white 96-well plate to 1 volume of 15 mg/mL D-luciferin (#LUCNA-1G, Goldbio, USA) or 1 volume of 0.5 mg/mL Nano-Glo In Vivo Substrate, fluorofurimazine (FFz; #CS320501, Promega, USA), respectively. Cells were imaged after a 10-min incubation protected from the light and imaged with a Fusion FX System (Vilber, France).

#### Cell surface marker expression analysis

The expression of cell surface markers was determined by FC. Briefly, SCSs were counted by Trypan blue exclusion and seeded in 96-well plates at a density of 2 × 10^5^ cells per well. Cells were washed with PBS and resuspended in 100 µL of antibody staining solution. Depending on the experiment, a viability staining step was performed either before antibody staining, using Zombie NIR Viability kit (#423105, BioLegend, USA) or after, using BD Pharmingen DAPI Solution (#564907, BD Biosciences, USA) or DRAQ7 (#424001, BioLegend, USA). Antibodies, dyes, and their respective dilutions are listed in Supplementary Table 4.

#### CAR T cell cytotoxicity assay

Killing assays were performed using an Incucyte S3 Live-Cell Analysis System (Sartorius, Germany). nEGFP-labeled target cells were seeded in flat-bottom, clear, 96-well plates at a density of 1 × 10^4^ cells per well and incubated for 24 h to allow cell monolayer formation. mCherry-labeled CAR T cells were added at various effector-target (E:T) ratios, and co-cultures were followed for 72 h. Brightfield and fluorescence images were recorded every 4 h with a 10X objective. Target cell viability kinetics were analyzed via time-lapse videos generated with Incucyte v2020B Software (Sartorius, Germany) and quantified using GraphPad Prism v10 Software (GraphPad, USA). Target cells incubated in medium alone were used to determine the baseline viability kinetics of each target cell line. Depending on the experiment, specific target cell lysis was calculated for each 4 h timepoint as green object area or count. All conditions were performed as duplicates or triplicates. Representative time-lapse videos of all assessed GBM:CAR T cell co-culture conditions are provided as Supplementary Movies 1–8.

#### CAR T cell degranulation assay

T cell degranulation in co-cultures with GBM cells was assessed by FC. Briefly, tumor cells were washed with PBS, dissociated, and counted by Trypan blue exclusion. GBM cells were seeded in a flat-bottom 96-well plate at a density of 1 × 10^4^ cells per well in 100 µL of growth medium and incubated for 24 h to form a cell monolayer. Afterward, media in the wells was discarded, replaced by 100 µL of CAR T cell or mock-transduced T cell suspensions in GBM growth medium in 1:1 E:T, and incubated for 24 h. At 24 h of co-culture, suspension cells were gently mixed in the supernatant and collected into a round-bottom 96-well plate. Cells were washed with PBS and stained with a BB700-conjugated anti-LAMP1 (clone H4A3; #566558, BD Biosciences, USA) for 20 min in the dark at 4 °C. After surface staining, the cells were washed 3 times with autoMACS Running Buffer and resuspended in 100 µL per well of 0.5X DAPI diluted in autoMACS Running Buffer. Antibodies, dyes, and their respective dilutions are listed in Supplementary Table 4. Samples were acquired with a CytoFLEX Flow Cytometer, and data were analyzed with FlowJo v10 Software. Positively stained cells were differentiated from the background using unstained controls. All conditions were performed as triplicates.

#### GBM-CAR T cell co-culture supernatant ELISA

Flat-bottom F96 MAXISORP NUNC-IMMUNO plates (#439454, Thermo Scientific, USA) were coated with 50 µL per well of Purified anti-human IFNγ Antibody (clone MD-1; #507502, BioLegend, USA) diluted 1:200 in 1X PBS (#5460-0023, BioConcept, Switzerland) and incubated overnight at 4 °C. The following day, wells were washed three times with PBS-T buffer (PBS with 0.05% Tween 20) and blocked for unspecific binding with 100 µL per well of 1% SureBlock solution (#SB232010, LubioScience, Switzerland) for 1 h at RT. After the blocking, plates were washed three times with PBS-T, then 50 µL of 24 h co-culture supernatants or serial dilutions of Recombinant Human IFNγ standard (#300-02, Peprotech, USA) were added to the wells and incubated for 2 h at RT. Wells were washed three times before adding 50 µL per well of Biotin anti-human IFN-γ Antibody (clone 4S.B3; #502504, BioLegend, USA) diluted 1:400 in 1% SureBlock solution and incubated 1 h at RT. Wells were washed thrice before adding 50 µL per well of HRP Streptavidin (#405210, BioLegend, USA) diluted 1:2000 in 1% SureBlock solution and incubated 1 h at RT. Wells were washed three times, and 100 µL per well of SIGMAFAST OPD tablet (#P9187, Sigma-Aldrich, USA) solution was added. The chromogenic reactions were stopped by adding 50 µL per well of 10% sulfuric acid (H_2_SO_4_), and absorbance values were measured by a Synergy H1 Hybrid microplate reader (BioTek, USA). Absolute IFNγ concentrations in test samples were interpolated from a standard curve and represented using GraphPad Prism v10 Software.

#### On-cell CD47 blocking assay

BS153 viability and count were assessed by Trypan blue exclusion, after which cells were seeded in flat-bottom 96-well plates at a density of 3 × 10^5^ cells per well. Cells were then treated for 30 min at 4 °C with 50 µL per well of 10 µg/mL of InVivoMAb anti-human CD47 (clone B6.H12; #BE0019-1, Bio X Cell, USA) or InVivoMAb mouse IgG1 isotype control (clone MOPC-21; #BE0083, Bio X Cell, USA) or conditioned-media from 24 h-rested, antigen-naïve aEGFRvIII or aEGFRvIII-SGRP CAR T cells seeded at a density of 1 × 10^6^ cells per mL of unsupplemented RPMI. Pre-treated tumor cells were washed with PBS and incubated for 30 min at 4 °C with biotinylated SIRPα (bt-SIRPα; #CDA-H82F2, ACROBiosystems, USA), which bound to the unblocked CD47 on BS153 cells. Finally, APC Streptavidin (SA; #405207, BioLegend, USA) staining was performed, followed by three washes with autoMACS Running Buffer and resuspension in 100 µL per well of 0.5X DAPI in autoMACS Running Buffer. Antibody treatments, SA stains, dyes, and their final concentrations/dilutions are listed in Supplementary Table 4. Samples were acquired in a CytoFLEX Flow Cytometer, and data were analyzed with FlowJo v10 Software.

#### CD14^+^ monocyte differentiation and culture

Primary monocytes were thawed, washed in PBS and plated at 0.8 × 10^6^ cells per mL in RPMI 1640 with 1X GlutaMAX (#61870-036, Gibco, USA), 10% FBS (#P30-3302, PAN-Biotech, Germany), 5% human serum (#H4522, Sigma-Aldrich, USA) and 25 ng/mL M-CSF (#300-25, PeproTech, USA), and incubated at 37 °C in a 5% CO_2_ atmosphere. After 48 h, the medium was replaced with human serum-free growth media and refreshed every 2–3 days for a maximum of 14 days until phagocytosis assays.

#### Phagocytosis assay

MDMs were detached from culture dishes using cell scrapers after a 15-min incubation at 37 °C in TrypLE Express (#12604-021, Gibco, USA). Cells were washed in PBS, counted, seeded at 5 × 10^4^ cells per well in a 96-well flat-bottom plate (#353072, Corning, USA), and incubated for 48 h to allow attachment. CAR T cells were counted and diluted to 1 × 10^6^ cells per mL in X VIVO 15. Tumor cells were washed in PBS, dissociated, and counted. All cell counts were performed by Trypan Blue exclusion. U251vIII cells were diluted to 1 × 10^6^ cells per mL in IMDM. U87 cells were stained with 62.5 × 10 nM CellTracker Green (#C2925, Thermo Scientific, USA) diluted in IMDM (#12440-053, Gibco, USA) for 30 min at 37 °C at a density of 1 × 10^6^ cells per mL. After two washes with PBS, stained cells were counted and adjusted to 1 × 10^6^ cells per mL in IMDM. U87 and U251vIII cells were then mixed in a 1:1 ratio. The tumor cell mixture (1 × 10^5^ cells) and CAR T cells (5 × 10^4^ cells) were added to each well containing MDMs. The contents were resuspended in IMDM and incubated at 37 °C for 3 h. After incubation, cells were detached using TrypLE, transferred to a 96-well U-bottom plate, and washed twice in PBS. Viability staining was carried out by incubating cells with a Zombie UV Viability kit (#423107, BioLegend, USA) for 20 min at 4 °C in the dark. Fc-block was performed by incubating cells in a dilution of Human TruStain FcX (#101320, BioLegend, USA) for 10 min at 4 °C. Antibody mastermixes (full-stains and FMOs) were prepared freshly in autoMACS Running Buffer. Cells were stained with surface marker antibody mastermixes for 25 min at 4 °C in the dark and washed twice in autoMACS Running Buffer. Cells were fixed by incubation for 20 min at RT using a Cyto-Fast Fix/Perm Buffer set (#426803, BioLegend, USA). The spectral FC staining panel with antibodies, their dyes, and respective dilutions is summarized in Supplementary Table 4. After antibody staining, samples were washed twice with autoMACS Running Buffer, resuspended in 200 µL MACS, and acquired on a Cytek Aurora 5-Laser Spectral Analyzer (Cytek Biosciences, USA), using standard, daily quality-controlled, Cytek-Assay-Settings. All conditions were performed with donor-matched CAR T cells and MDMs from 4 HDs.

#### GBM mouse models and survival assessment

All experiments involving GBM models were performed on NSG mice of the male sex, aged 7–12 weeks at the time of tumor implantation. To assess the efficacy of anti-EGFRvIII CAR T cell and anti-CD47 antibody monotherapies, mice were injected i.c. with 5 × 10^4^ U251vIII-NLuc cells. To test our proposed combination therapy, mice were injected i.c. with a total of 5 × 10^4^ GBM cells consisting of 2.5 × 10^4^ U87-Luc2 and 2.5 × 10^4^ U251-NLuc, resulting in EGFRvIII-mosaic tumors. In both models, the animals were anesthetized in an induction chamber with 2.0 ± 0.5% isoflurane in an O_2_ atmosphere immediately before the tumor injections. Anesthesia on the stereotactic frame (Neurostar, Germany) was maintained at 2.0 ± 0.5% isoflurane delivered through a nose/mouth adaptor. General analgesia (Buprenorphine; Bupaq-P, Streuli Tiergesundheit, Switzerland) was given s.c. immediately before surgery at 0.05 mg per kg, and local analgesia was applied s.c. under the scalp. Eye gel (Lacrinorm, Bausch+Lomb Swiss AG, Switzerland) was applied to prevent drying of the eyes during surgery. The scalp was briefly swabbed with povidone-iodide solution, and a midline incision was made. A burr hole was manually drilled 2 mm lateral from the cranial midline and 1 mm posterior of the bregma suture (Supplementary Fig. 3a). A digitally controlled injection was performed with the Stereodrive v1 Software (Neurostar, Germany) using a 10 µL syringe (#80300, Hamilton, USA). The syringe was lowered into the burr hole to a depth of 3 mm below the surface of the dura and retracted by 0.5 mm to form a small reservoir. Four µL of SCS were injected at 1 µL per min. The needle was left in place for at least 1 min and carefully retracted by 0.5 mm every 30 s. After injection, the incision was sutured (#8661H, Ethicon, USA). The following treatments and controls were administered i.t. in a volume of 4 µL on days 7 and 14: vehicle (PBS), antibody isotype (InVivoMAb mouse IgG1 isotype control, clone MOPC-21; 5 µg), aCD47 (InVivoMAb anti-human CD47, clone B6.H12; 5 µg), aCD19 CAR (5 × 10^5^ cells), aCD19-SGRP CAR (5 × 10^5^ cells), aEGFRvIII CAR (5 × 10^5^ cells), aEGFRvIII CAR + aCD47 (5 × 10^5^ cells and 5 µg, respectively) or aEGFRvIII-SGRP CAR (5 × 10^5^ cells). In EGFRvIII-mosaic survival experiments, aCD47 and aEGFRvIII CAR + aCD47 treatment groups received additional doses of anti-CD47 (100 µg) administered i.p. in a volume of 100 µL on days 19, 22, 26, and 29. In the EGFRvIII-mosaic, CCL3 blockade survival experiment, CAR T cells were applied as described above, and 50 ng of antibody per mouse (aCCL3 or isotype) were administered i.p. in a volume of 100 µL on days 8, 10, 13, 15, 17, 20, 22, 24, 27, 29, 31, 34, 36, 38, and 41. Antibody treatments and their respective final concentrations by application are listed in Supplementary Table 4. For survival experiments, tumor cell implantation was set as day 0, and the survival time was set as the day of euthanasia. Mice were monitored for clinical signs until day 90, upon which all remaining survivors were either euthanized or assigned to a tumor rechallenge experiment. Animals assigned to a rechallenge experiment were injected with the same mix of tumor cells using the same procedure described above. Tumor-rechallenged animals received no treatment, and a historic vehicle group was used as a control. Kaplan–Meier survival comparison was performed using a log-rank (Mantel–Cox) test with GraphPad Prism v10 Software.

#### In vivo GBM bioluminescence imaging

GBM engraftment and growth were monitored by BLi. Mice implanted i.c. with EGFRvIII-mosaic tumors were subjected to dual BLi with specific substrates to monitor the growth of the EGFRvIII^+^ and EGFRvIII^-^ tumor fractions. The animals’ clinical scores and luminescence images were taken weekly, after i.p. injection of 150 mg kg^−1^ of D-luciferin or intravenous (i.v.) injection of 0.325 µmol of fluorofurimazine per mouse. Subsequently, mice were anesthetized by isoflurane inhalation and imaged on a Newton 7.0 instrument (Vilber, France). Bioluminescence counts were measured using NEWTON v7 Software (Vilber, France) with a defined ROI (Supplementary Fig. 3f) overlaid on the head surface area of individual mice. Bioluminescence curves were generated using GraphPad Prism v10 Software. Tumor-free survival curves were calculated by combining survival and bioluminescence measurements. Animals reaching a bioluminescence score 20% above the healthy control threshold for either NLuc or Luc2 were considered not tumor-free.

#### Mouse tissue collection and processing

For spectral FC analyses, mice were euthanized by CO_2_ suffocation, and brain regions were immediately harvested into ice-cold HBSS. Depending on the experiment, the tumor-injected hemisphere, contralateral hemisphere, or brain meninges were carefully dissected and manually minced using razor blades and enzymatically dissociated at 37 °C for 30 min with 1 mg/mL collagenase type IV (#LS004188, Worthington Biochemical Corporation, USA) and 250 μ/mL DNase 1 (#10104159001, Roche, Switzerland) in a buffer containing HBSS with Ca^2+^/Mg^2+^ (#14205-050, Gibco, USA), 1% MEM NEAA, 1 mM sodium pyruvate, 44 mM sodium bicarbonate (#25080-060, Gibco, USA), 25 mM HEPES (#H0887, Gibco, USA), 1% GlutaMAX-I and 1% antibiotic-antimycotic (#15240062, Gibco, USA). Extraction of the meninges was performed as described earlier^69^. The resulting cell suspensions were filtered through a 70 μm strainer and centrifuged in a density gradient using debris removal solution (#130-109-398, Miltenyi Biotec, Germany) according to the manufacturer’s protocol to remove myelin and cell debris. Erythrocytes were removed using ACK lysing solution (#A1049201, ThermoFisher Scientific, USA), and cell suspensions were washed with PBS and kept on ice until spectral flow staining. For IHC, quantification of brain tumor area, IF brain analysis, and spleen size measurement, mice were anesthetized i.p. with a mix of 80 mg per kg of ketamine (Ketanarkon, Streuli Tiergesundheit, Switzerland) and 16 mg per kg of xylazine (Rompun, Elanco, USA), and transcardially perfused with ice-cold PBS. Brains and spleens were dissected and immediately fixed in formalin at 4 °C for 72 h before paraffin embedding.

### Spectral flow cytometry of mouse tissues

#### Staining and acquisition

After the ACK-lysis step described in the “Mouse tissue collection and processing” section of the Supplementary Methods, freshly dissociated cells were washed with PBS and resuspended in 400 µL of PBS. A 200 µL fraction of one sample per treatment group was used as an unstained control to detect and correct for condition-specific autofluorescence. In all cases, the input volume for full-stained samples was kept at 200 µL to ensure equivalent staining conditions across all samples. The remaining 200 µL of SCS volume was mixed and used to generate fluorescence minus one (FMO) stainings, allowing for condition-specific FMO gating. All centrifugation and incubation steps were performed at 300 × *g* at 4 °C, protected from light, unless otherwise stated. Viability staining was performed by incubating cells with a Zombie Aqua or NIR Viability kit for 20 min. Subsequently, Fc-block was performed by incubating cells in a dilution of human TruStain FcX (#422302, BioLegend, USA) and mouse TruStain FcX (#101320, BioLegend, USA) for 10 min. Antibody mastermixes (full-stains and FMOs) were freshly prepared on the day of staining in Brilliant Stain Buffer (#00-4409-75, ThermoFisher Scientific, USA). Cell surface markers were stained with surface antibody mastermixes for 25 min and then washed twice in autoMACS Running Buffer. To allow the subsequent staining of intracellular antigens, a fixation/permeabilization step was performed by incubating the cells for 20 min at RT using a Cyto-Fast Fix/Perm Buffer set (#426803, BioLegend, USA). Intracellular antibody staining was then performed for 25 min at RT. The spectral FC staining panels employed in each experiment are provided in Supplementary Table 4. Finally, the samples were washed twice, resuspended in a final volume of 200 µL of PBS, and acquired on a Cytek Aurora 5-Laser Spectral Analyzer (Cytek Biosciences, USA) using standard daily quality-controlled Cytek-Assay-Settings.

#### Data preprocessing

Spectral unmixing was performed using SpectroFlo v3.1.0 Software. UltraComp eBeads Plus Compensation Beads (#01-3333-42, ThermoFisher Scientific, USA) were used as single reference controls (SRC). SRC for mCherry and mTagBFP2 were generated with mock-transduced and the respective transduced CAR T cells (mCherry) and U87 tumor cells (mTagBFP2) from cell cultures. Due to the fluorescent nature of our unstained single cell suspensions (mCherry^+^ CAR T cells and mTagBFP2^+^ tumor cells) and to avoid contamination of the autofluorescence signature of all immune populations, fcs files of ‘unstained’ conditions were first cleaned by the exclusion of V3^hi^ mTagBFP2 tumor cells and YG3^hi^ mCherry CAR T cells. The resulting fcs files were reimported to detect populations with highly divergent autofluorescence signatures by NxN plots. When detected, such populations were extracted similarly and separately added as a fluorescence tag to avoid assigning incorrect marker expression caused by heterogenous autofluorescence between immune cell populations. Conventional analysis and generation of input fcs files (gates: debris removal, single cells, live cells, and CD45^+^ cells) for downstream analysis with R was performed using FlowJo v10 Software and is detailed in the “FlowSOM analysis” section below.

#### FlowSOM analysis

Data were manually pre-gated to remove the debris and select for CD45^+^, live, single cells using FlowJo v10 Software. The analysis was subsequently performed in R v4.3.1. Data was transformed by asinh transformation using variance stabilizing cofactors for each channel (*estParamFlowVS* and *transFlowVS* functions from the FlowVS package), except for the mTagBFP2 channel where the cofactor was manually set to 3. Preprocessing QC (using the *PeacoQC* function from the PeacoQC package) was performed to remove outliers and unstable events (IT_limit was set at 0.55 and MAD at 6). Clustering was performed using FlowSOM and ConsensusClusterPlus using the wrapper function *cluster* from the CATALYS package (xdim = 10, ydim = 10, maxK = 15, seed = 1234). The resulting clusters were manually annotated. Differential testing was performed using the diffcyt package v1.3.0 [https://github.com/lmweber/diffcyt/tree/devel?tab=readme-ov-file]^70^. The diffcyt-DA-edgeR function was used for differential abundance and the diffcyt-DS-limma function for differential state.

#### Conventional flow cytometry data analysis

FlowJo v10 Software was used for data analysis. Gates were set using FMO controls. Either the percentage of cell population of interest or median fluorescence intensity (MFI) was reported.

### Mouse brain immunohistochemistry

#### Stainings

Formalin-fixed brains were embedded in paraffin, and 5 µm sections were made using a microtome. Slides were stained according to the standard H&E protocol using the automated Gemini AS Slide Stainer (ThermoFisher Scientific, USA) and covered with Permount Mounting Medium (#SP15‐100, ThermoFisher Scientific, USA). For CD3 and CD68 IHC stainings, formalin-fixed paraffin-embedded (FFPE) brain sections were stained with primary antibody in a Ventana DISCOVERY ULTRA Research Staining System (Ventana Medical Systems Inc., USA), using Histofine Simple Stain MAX PO anti-rabbit (UIP anti-rabbit; #414142F, Nicherei Biosciences Inc., Japan) as a detection reagent. Antibodies, the detection reagent, and their final dilutions are listed in Supplementary Table 4. Slides were counterstained with Hematoxylin II (#790-2208, Ventana Medical Systems Inc., USA) and post-counterstained with Bluing Reagent (#760-2037, Ventana Medical Systems Inc., USA). For myelin staining, brain sections were deparaffinized and rehydrated before incubation in Luxol solution (#1B 389, Medite, Switzerland) at 60 °C for 2 h. After cooling, sections were washed with 96% ethanol and flowing tap water for 5 min, and then distilled water. Differentiation was performed with 0.1% lithium carbonate solution (#62470, Sigma-Aldrich, USA) for 10 s. After washing the sections in distilled water, nuclei were stained using 1% cresyl violet (#1.05235, Merck, Germany) in 96% ethanol for 5 s until the background became colorless. Sections were then dehydrated in 100% ethanol, Xylol (#253-VI53TE, Biosystems, Switzerland), and embedded (#41-4012-00, HistoLab, Sweden).

#### Acquisition

Slides were acquired using a Nanozoomer S60 digital slide scanner (Hamamatsu, Japan) with a 40X objective.

#### Histological brain tumor size measurement

To calculate tumor size, a pixel classifier was trained to detect tumors in H&E-stained mouse brain histological sections in QuPath v0.4.3^71^. The Random Trees Classifier was used with the following parameters: Pixel size: 3.53 µm; Channels: red, green, and blue; Features: Gaussian and Laplacian of Gaussian; Scales: 4.0 and 8.0; no normalization. Classification of tumor areas was based on example annotations to distinguish brain tissue, tumor, and background (ignore*). Results were visually verified. The cumulative tumor area per sample was quantified using GraphPad Prism v10 Software. Source data are provided as Supplementary Data 3.

#### CD3 and CD68 quantification

Cells were segmented based on Hematoxylin staining with the StarDist2D plugin^72^ within QuPath v0.4.3^71^ using the following parameters: Probability threshold: 0.2; Pixel size: 0.2 µm; Cell expansion: 2.0 µm; using the pre-trained model ‘he_heavy_augment.pb’. Cells were classified for CD3- and CD68-positivity based on mean UIP staining in the nucleus with a threshold of 0.12 and 0.2, respectively. All images were prepared using OMERO.web app [www.openmicroscopy.org/omero/figure/]. The tumor core was delineated based on nuclear stain density, and the tumor rim was defined as a 100 μm-wide area surrounding the tumor core. Relative cell positivity per brain section was calculated as a percentage of stained vs non-stained cells in areas defined as tumor core or tumor rim and quantified using GraphPad Prism v10 Software. Source data are provided in Supplementary Data 3.

#### Olink proteomics of mouse plasma

Peripheral blood samples were collected ~24 h after each treatment dose (days 8 and 15 after tumor implantation). Unanesthetized mice were briefly put under a heat lamp and placed in a cylindrical restrainer. A small puncture was made on the tail to allow the dripping of ~100 µL of blood into lithium heparin-coated Microvette 100 capillary blood collection tubes (#20.1282.100, Sarstedt, Germany). Blood samples were centrifuged at 1500 × *g* for 15 min at RT, and the top layer of plasma was transferred into sterile 0.5 µL Eppendorf tubes. Samples were immediately frozen at −80 °C until analysis. Fifty-four samples were analyzed by proximity extension assay technology using a standard Olink Target 96 Immuno-Oncology panel (Olink Holding, Sweden). Samples were measured neat and processed according to the manufacturer’s instructions. Randomization of the samples was performed using random.org. Samples were analyzed across two plates, and 16 samples from the first dataset were included in the second dataset for reference sample normalization. Samples with good detection that passed QC and provided a wide range of the input data of the first dataset were selected using the ‘olink_bridgeselector’ function from the ‘Olink Analyze’ R package as bridging samples for the second dataset. Analysis of the bridging procedure consisted of calculating the median of the paired normalized protein expression levels (NPX) differences per protein between the overlapping samples to determine the adjustment factors to be applied between the two datasets. For the overlapping samples, the mean value was kept. A cyclic loess normalization was applied. Since data below the limit of detection may be non-linear, the differential expression for the contrasts of interest was assessed by a Mann–Whitney U test with Benjamini–Hochberg correction. Source data is available as Supplementary Data 2.

### Mouse brain immunofluorescence multiplexing

#### Staining

The protocol was adapted from Gut and colleagues^73^. FFPE brains were sectioned in 5 µm-thick sections using a microtome. Slides were deparaffinized three times for 5 min with ROTI Histol (#6640, Carl Roth, Germany) and 30 s with 100% EtOH, 95% EtOH, 70% EtOH, 50% EtOH and dH_2_O washing steps. For antigen retrieval, slides were exposed to pre-heated 1X Citrate Buffer (#C9999, Sigma-Aldrich, USA), followed by a washing step with PBS for 10 min. Tissue sections were permeabilized in PBS with 0.2% Tween-20 (#P5927, Sigma-Aldrich, USA) twice for 15 min. After that, the slides were washed with PBS twice for 10 min, followed by blocking for 1 h in a dark, humid chamber with 10% normal donkey serum (#017-000-121, Jackson ImmunoResearch, USA) diluted in PBS. Afterward, the slides were stained in different cycles overnight at 4 °C with the following antibodies: anti-CD3 (clone CD3-12; #MCA1477, Bio-Rad, USA), anti-CD206 (clone E6T5J; #24595S, Cell Signaling Technology, USA), anti-EGFRvIII (clone RM419; #MA5-36216, ThermoFisher Scientific, USA), anti-GFAP (clone D1F4Q; #12389S, Cell Signaling Technology, USA), anti-IBA1 (polyclonal; #NB100-1028, Novus Biologicals, USA), anti-Ki67 (clone SolA15; #14-5698-82, ThermoFisher Scientific, USA) and anti-TMEM119 (clone 28-3; #ab209064, Abcam, UK). After each primary antibody staining cycle, the slides were washed three times for 5 min with PBS and stained with the respective secondary fluorescent antibodies and DAPI for 1 h in a dark, humid chamber at RT. Antibodies, dyes, and their final dilutions are listed in Supplementary Table 4. The slides were washed three times for 5 min with PBS and mounted with imaging buffer at pH 7.4 (700 mM N-Acetyl-Cysteine (NAC; #160280250, ThermoFisher Scientific, USA) in ddH_2_O + 20% HEPES solution (#H0887, Sigma-Aldrich, USA)). For antibody elution after each imaging cycle, the slides were treated three times for 15 min with elution buffer containing 0.5 M L-Glycine (#3790.2, Carl Roth, Germany), 1.2 M Urea (#U5378, Sigma-Aldrich, USA), 3 M Guanidium chloride (#G3272, Sigma-Aldrich, USA), and freshly added 70 mM TCEP-HCl (#C4706, Sigma-Aldrich, USA), followed by a ddH_2_O wash and restaining.

#### Acquisition

Images were acquired using an ECLIPSE Ni upright microscope (Nikon, Japan) equipped with 395, 470, 561, and 640 nm lasers with a Prior PL-200 robotic slide loader equipped with Microscan MiniHawk (Omron Microscan Systems Inc., Japan) and the Photometrics Prior 95B camera (Teledyne Photometrics, USA). The acquisition was done using JOBS automation in NIS-Elements v5.11.00 Software. 4X Plan Apo NA 0.2 (Nikon, Japan) objective was used to make a slide overview, and ‘general analysis3’ with ‘Otsu’ threshold was used to detect the tissue. The focus surface was created by performing software autofocus every 2 points based on the DAPI signal with Plan Apo λ 20X NA 0.8, and the tissue was scanned with the same objective. Slides were scanned after each staining cycle, with blank (unstained) cycles used to evaluate autofluorescence. The exposure time of each channel was constant for all cycles, and corresponding blank channels (GFP, Cy3, and Cy5) were subtracted from the signal cycle.

#### Image preprocessing

Fiji v2.9.0^74^ was used for image preprocessing. Single tiles were stitched using Grid/Collection Stitching^75^. Stitched tissues were registered based on the DAPI channel in consecutive cycles using the MultiStackReg plugin^76^, and the same correction was propagated on the remaining channels. Images were saved as pyramidal files using the Kheops plugin^77^, available under an open-source license [https://github.com/BIOP/ijp-kheops/releases].

#### Cell segmentation and quantification

Cells were segmented based on DAPI staining with the StarDist2D plugin^72^ within QuPath v0.4.3^71^ using the following parameters: Probability threshold: 0.5; Pixel size: 0.5 µm; Cell expansion: 2.0 µm; using a pre-trained model ‘dsb2018_heavy_augment.pb’. Object classification in QuPath defined cell positivity for CD3, CD206, EGFRvIII, and IBA1. All images were prepared using OMERO.web app [http://www.openmicroscopy.org/omero/figure/]. Cell population ratios per treatment condition were quantified using GraphPad Prism v10 Software. Source data are provided as Supplementary Data 3.

### Post-therapy toxicity monitoring

#### Spleen size measurement

The coronal oblique length of FFPE spleen cross-sections was measured and plotted as the ratio of spleen length to mouse bodyweight at the spleen collection time point. Source data are provided as Supplementary Data 3.

#### Mouse weight monitoring

Animals were weighed weekly, starting from the tumor-implantation day until endpoint.

#### Plasma IL6 and CRP

Plasma IL6 was assessed from the Olink proteomics dataset described in the “Olink proteomics of mouse plasma” section. Mouse CRP levels in plasma were investigated with a Mouse C-Reactive Protein ELISA kit (#41-CRPMS-E01, ALPCO Diagnostics, USA), following the manufacturer’s instructions. The chromogenic reactions were stopped by adding 50 µL per well of 10% sulfuric acid (H_2_SO_4_), and absorbance values were measured by a Synergy H1 Hybrid microplate reader (BioTek, USA). Absolute CRP concentrations in test samples were interpolated from a standard curve and represented using GraphPad Prism v10 Software.

#### Automated hematology analysis

Longitudinal analysis of hematological parameters was performed after a single CAR treatment using 20 µL of mouse blood collected from the tail vein into 1.5 mL Eppendorf tubes prefilled with 120 µL of 1 mM EDTA diluted in PBS. Blood samples from multiple time points (1, 3, 6, 13, and, 20 days after treatment) were immediately acquired on a XN-1000 benchtop hemocytometer (Sysmex, Japan). Values were normalized and scaled to allow multiple time point comparisons.

#### Myelin quantification

Brain sample collection and processing and the myelin staining procedure were described above in the “Mouse brain immunohistochemistry” section. Images were color-deconvolved^78^ to separate luxol and cresyl violet staining using QuPath v0.5.0^71^. The Random Trees Classifier was trained to detect brain tissue and myelin area with the following parameters: Pixel size: 7.07 µm; Channels: luxol and cresyl violet; Features: Gaussian, structure_tensor_eigenvalue_max, and hessian_determinant”; Scales: 1.0 and 4.0; no normalization. The positive area of myelin (luxol staining) was quantified within brain tissue as an area above the threshold. Results were visually verified. The relative myelin area per sample was plotted using GraphPad Prism v10 Software. Source data are provided in Supplementary Data 3.

#### Human GBM tissue processing

GBM tissue samples were transported on ice to the laboratory for dissociation into single-cell suspensions within 2–3 h after surgical resection. Human brain tissue was mechanically minced using razor blades and enzymatically dissociated as described in the “Mouse tissue collection and processing” section, with the difference that debris and myelin were removed by a 0.9 M sucrose (#84100, Sigma-Aldrich, USA) density gradient centrifugation. After ACK-lysis, the single-cell suspensions were washed with PBS, resuspended in Bambanker at an approximate density of 2 × 10^6^ live cells per mL, and stored long-term in LN_2_.

#### Pharmacoscopy

Frozen patient SCSs were thawed and resuspended in (DMEM #11995065, Gibco, USA) supplemented with 10% FBS, 25 mM HEPES, and 1% pen strep. They were seeded at 8 × 10^3^ cells per well in 50 µL per well into clear-bottom, tissue-culture treated CellCarrier-384 Ultra Microplates (#50-209-8071, Perkin Elmer, USA). mCherry-labeled CAR T cells were added and co-cultured with the patient single cell suspensions for 48 h at 37 °C, 5% CO_2_. Every CAR T cell co-incubation condition was tested with five technical replicate wells and six technical replicate wells for the PBS control. After the co-incubation period, the cells were fixed with 4% PFA, blocked with PBS containing 5% FBS, 0.1% Triton-X, and 4 µg/mL DAPI (#422801, BioLegend, USA) for 1 h at RT and stained with two staining panels: (1) anti-CD3 (clone UCHT1; #300415, BioLegend, USA) and anti-CD14 (clone HCD14; #325612, BioLegend, USA); and (2) anti-NESTIN (clone 10C2; #656802, BioLegend, USA) and anti-EGFRvIII (clone RM419; #MA5-36216, ThermoFisher Scientific, USA) overnight at 4 °C. The wells were washed, and the cells stained with primary antibodies were incubated with the secondary fluorescent antibodies for 1 h at RT in the dark. The staining panels are summarized in Supplementary Table 4. The plates were imaged with an Opera Phenix automated spinning-disk confocal microscope at 20X magnification (Perkin Elmer, USA). Single cells were segmented based on nuclear DAPI staining with CellProfiler v2.2.0 Software. Downstream image analysis was performed with MATLAB vR2021b. Marker-positive cell counts for each condition were identified based on each channel’s linear threshold, averaged across each well, and compared between CAR T cell co-incubation conditions. Cell counts per staining per replicate of patient-derived tumor co-cultures with CAR T cells are provided as Supplementary Data 4.

#### Lymphoma mouse models and survival assessment

All experiments involving lymphoma models were performed on NSG mice of the female sex, aged 8–12 weeks, were anesthetized with isoflurane and injected with 5 × 10^5^ CD19-expressing Raji cells s.c. in the right flank. Raji cells were suspended in BD Matrigel Basement Membrane Matrix High Concentration (#354248, BD Biosciences, USA) diluted 1:1 in phenol red-free DMEM (#31053028, Gibco, USA) without additives in a total volume of 100 µL. Three days after tumor inoculation, each mouse received 8 × 10^5^ CAR T cells suspended in 200 µL of PBS or PBS alone, i.v. via the tail vein. Tumor-bearing mice injected with unspecific CAR T cells or PBS were used as controls. For survival experiments, tumor cell implantation was set as day 0, and the survival time was set as the day of euthanasia. Tumor size was measured three times per week using a caliper. Animals were sacrificed before reaching a tumor volume of 1500 mm^3^ or when reaching an exclusion criterion (ulceration, severe weight loss, severe infection, or bite wounds). Tumor volume was calculated using the formula: ${{{\rm{Tumor\; volume}}}}({{{\rm{mm}}}}3)=({{{\rm{d}}}}2\times {{{\rm{D}}}})/2$, with D and d being the longest and shortest tumor parameter in mm, respectively.

#### SGRP detection by mass-spectrometry alternatives

CAR T cells were plated 3 days before the experiment, incubated with complete growth medium and allowed to expand until reaching the cell density of 1 × 10^6^ cells/mL. On the day of the experiment, the cells were centrifuged at 300 × *g* for 5 min and the supernatants were carefully removed and used in downstream assays. Experiments involving ELISA assays were performed using a CD47:SIRP alpha Biotinylated Inhibitor Screening ELISA Assay Pair (#EP-102, ACROBiosystems, USA) or Human SIRP alpha DuoSet ELISA (#DY4546-05, R&D Systems, USA). The assays to generate the standard curves and supernatant analysis were performed following the manufacturer’s instructions. For Western Blot assays involving cell lysates, the cell pellets were resuspended and lysed using RIPA cell lysis buffer supplemented with protease inhibitor cocktail and phosphatase inhibitors on ice for 30 min. The cells were centrifuged at full speed for 15 min at 4 °C. Both cell lysates and supernatants were supplemented with Laemmli buffer 4X (#1610747, Bio-Rad, USA) and loaded on SDS-PAGE gels (4–15% Mini-PROTEAN TGX Precast Protein Gels, 15-well, 15 µL; #4561086, Bio-Rad, USA). The proteins were then transferred onto nitrocellulose membranes (TransBlot Turbo Mini 0.2 µm Nitrocellulose Transfer Packs; #1704158, Bio-Rad, USA). The membranes were blocked for 1 h with 5% non-fat dry milk (#M7409, Sigma-Aldrich, USA) in TBS buffer supplemented with 0.05% Tween-20 (TBST) at RT for 1 h and then probed with the primary antibody (HRP-conjugated 6*His, His-Tag Monoclonal antibody; #HRP-66005, Proteintech, USA) diluted 1:1000 in blocking buffer and incubated overnight at 4 °C. After five times washing with TBST, the membranes were exposed using ECL substrate (SuperSignal West Pico PLUS Chemiluminescent Substrate; #34578, ThermoFisher Scientific, USA) and imaged with a Fusion-FX (Vilber, France). For mCherry imaging, the blots were imaged in the same instrument with a f595 Y3 filter.

### Statistics and reproducibility

Data analysis and visualization were performed using Microsoft Excel v16.14.1 Software (Microsoft, USA) and GraphPad Prism v10 Software. Graphs represent either group mean values ±SD (for in vitro experiments), or ±SEM (for in vivo experiments), or individual values. For statistical tests, *P* < 0.05 was considered statistically significant. Significance is shown directly within Figures or Supplementary Figs. with *P* or *adjusted P* values. All experiments were repeated once with similar results unless otherwise stated in Figure or Supplementary Fig. legends.

### Reporting summary

Further information on research design is available in the Nature Portfolio Reporting Summary linked to this article.

## Supplementary information

Supplementary Information

Description of Additional Supplementary Files

Supplementary Data 1

Supplementary Data 2

Supplementary Data 3

Supplementary Data 4

Supplementary Movie 1

Supplementary Movie 2

Supplementary Movie 3

Supplementary Movie 4

Supplementary Movie 5

Supplementary Movie 6

Supplementary Movie 7

Supplementary Movie 8

Reporting Summary

Transparent Peer Review file

## Source data

Source Data

## Data Availability

The protein mass spectrometry data generated in this study have been deposited in the MassIVE database under accession code PXD054059 [https://massive.ucsd.edu/ProteoSAFe/dataset.jsp?task=a1b0183b582548eda092f2487ba065fb]. The remaining data are available within the Article, Supplementary Information, or Source Data file. Source data are provided with this paper.

## Code availability

All code used for data analysis is referenced in the relevant Methods subsections. Original R scripts used in the study are available in a GitHub repository under 10.5281/zenodo.13819903 [https://github.com/BTITLab/SGRP_CART]^79^.

## References

[1] Cloughesy, T. F., Cavenee, W. K. & Mischel, P. S. Glioblastoma: from molecular pathology to targeted treatment. *Annu. Rev. Pathol.* **9**, 1–25 (2014). 23937436 10.1146/annurev-pathol-011110-130324

[2] Ostrom, Q. T. et al. CBTRUS Statistical report: primary brain and other central nervous system tumors diagnosed in the United States in 2012-2016. *Neuro. Oncol.* **21**, v1–v100 (2019). 31675094 10.1093/neuonc/noz150 PMC6823730

[3] Newick, K., O’Brien, S., Moon, E. & Albelda, S. M. CAR T cell therapy for solid tumors. *Annu. Rev. Med.* **68**, 139–152 (2017). 27860544 10.1146/annurev-med-062315-120245

[4] June, C. H. & Sadelain, M. Chimeric antigen receptor therapy. *N. Engl. J. Med.* **379**, 64–73 (2018). 29972754 10.1056/NEJMra1706169 PMC7433347

[5] Hou, A. J., Chen, L. C. & Chen, Y. Y. Navigating CAR-T cells through the solid-tumour microenvironment. *Nat. Rev. Drug Discov.* **20**, 531–550 (2021). 33972771 10.1038/s41573-021-00189-2

[6] Felsberg, J. et al. Epidermal growth factor receptor variant III (EGFRvIII) positivity in EGFR-amplified glioblastomas: prognostic role and comparison between primary and recurrent tumors. *Clin. Cancer Res.* **23**, 6846–6855 (2017). 28855349 10.1158/1078-0432.CCR-17-0890

[7] An, Z., Aksoy, O., Zheng, T., Fan, Q. W. & Weiss, W. A. Epidermal growth factor receptor and EGFRvIII in glioblastoma: signaling pathways and targeted therapies. *Oncogene* **37**, 1561–1575 (2018). 29321659 10.1038/s41388-017-0045-7 PMC5860944

[8] O’Rourke, D. M. et al. A single dose of peripherally infused EGFRvIII-directed CAR T cells mediates antigen loss and induces adaptive resistance in patients with recurrent glioblastoma. *Sci. Transl. Med.* **9**, eaaa0984 (2017). 10.1126/scitranslmed.aaa0984 PMC5762203 28724573

[9] Luksik, A. S., Yazigi, E., Shah, P. & Jackson, C. M. CAR T. Cell therapy in glioblastoma: overcoming challenges related to antigen expression. *Cancers* **15**, 1414 (2023). 10.3390/cancers15051414 PMC10000604 36900205

[10] Choi, B. D. et al. Intraventricular CARv3-TEAM-E T cells in recurrent glioblastoma. *N. Engl. J. Med.* **390**, 1290–1298 (2024). 38477966 10.1056/NEJMoa2314390 PMC11162836

[11] Arrieta, V. A. et al. Immune checkpoint blockade in glioblastoma: from tumor heterogeneity to personalized treatment. *J. Clin. Invest.* **133**, e163447 (2023). 10.1172/JCI163447 PMC9843050 36647828

[12] Yeo, A. T. et al. Single-cell RNA sequencing reveals evolution of immune landscape during glioblastoma progression. *Nat. Immunol.* **23**, 971–984 (2022). 35624211 10.1038/s41590-022-01215-0 PMC9174057

[13] Lakshmanachetty, S. & Mitra, S. S. Mapping the tumor-infiltrating immune cells during glioblastoma progression. *Nat. Immunol.* **23**, 826–828 (2022). 35624212 10.1038/s41590-022-01223-0

[14] Wolf, S. A., Boddeke, H. W. & Kettenmann, H. Microglia in physiology and disease. *Annu. Rev. Physiol.* **79**, 619–643 (2017). 27959620 10.1146/annurev-physiol-022516-034406

[15] Martins, T. A. et al. Microglia-centered combinatorial strategies against glioblastoma. *Front. Immunol.* **11**, 571951 (2020). 33117364 10.3389/fimmu.2020.571951 PMC7552736

[16] Barclay, A. N. & Van den Berg, T. K. The interaction between signal regulatory protein alpha (SIRPalpha) and CD47: structure, function, and therapeutic target. *Annu. Rev. Immunol.* **32**, 25–50 (2014). 24215318 10.1146/annurev-immunol-032713-120142

[17] Willingham, S. B. et al. The CD47-signal regulatory protein alpha (SIRPa) interaction is a therapeutic target for human solid tumors. *Proc. Natl Acad. Sci. USA* **109**, 6662–6667 (2012). 22451913 10.1073/pnas.1121623109 PMC3340046

[18] Hutter, G. et al. Microglia are effector cells of CD47-SIRPalpha antiphagocytic axis disruption against glioblastoma. *Proc. Natl Acad. Sci. USA* **116**, 997–1006 (2019). 30602457 10.1073/pnas.1721434116 PMC6338872

[19] Gholamin, S. et al. Disrupting the CD47-SIRPalpha anti-phagocytic axis by a humanized anti-CD47 antibody is an efficacious treatment for malignant pediatric brain tumors. *Sci. Transl. Med.* **9**, eaaf2968 (2017). 10.1126/scitranslmed.aaf2968 28298418

[20] Sikic, B. I. et al. First-in-human, first-in-class phase I trial of the anti-CD47 antibody Hu5F9-G4 in patients with advanced cancers. *J. Clin. Oncol.* **37**, 946–953 (2019). 30811285 10.1200/JCO.18.02018 PMC7186585

[21] Advani, R. et al. CD47 blockade by Hu5F9-G4 and rituximab in Non-Hodgkin’s lymphoma. *N. Engl. J. Med.* **379**, 1711–1721 (2018). 30380386 10.1056/NEJMoa1807315 PMC8058634

[22] Dizman, N. & Buchbinder, E. I. Cancer therapy targeting CD47/SIRPalpha. *Cancers* **13**, 6229 (2021). 10.3390/cancers13246229 PMC8699673 34944850

[23] Habashy, K. J., Mansour, R., Moussalem, C., Sawaya, R. & Massaad, M. J. Challenges in glioblastoma immunotherapy: mechanisms of resistance and therapeutic approaches to overcome them. *Br. J. Cancer* **127**, 976–987 (2022). 35662275 10.1038/s41416-022-01864-w PMC9470562

[24] Weiskopf, K. et al. Engineered SIRPalpha variants as immunotherapeutic adjuvants to anticancer antibodies. *Science* **341**, 88–91 (2013). 23722425 10.1126/science.1238856 PMC3810306

[25] Dacek, M. M. et al. Potentiating antibody-dependent killing of cancers with CAR T cells secreting CD47-SIRPalpha checkpoint blocker. *Blood* **141**, 2003–2015 (2023). 36696633 10.1182/blood.2022016101 PMC10163312

[26] Ring, A. M., Maute, R. L., Kruse, A. C., Manglik, A. & Lin, K. S., inventors; Ab Initio Biotherapeutics Inc, assignee. Sirp polypeptide compositions and methods of use. US patent US20160340397A1 (2016).

[27] Johnson, L. A. et al. Rational development and characterization of humanized anti-EGFR variant III chimeric antigen receptor T cells for glioblastoma. *Sci. Transl. Med.* **7**, 275ra222 (2015). 10.1126/scitranslmed.aaa4963 PMC4467166 25696001

[28] Milone, M. C. et al. Chimeric receptors containing CD137 signal transduction domains mediate enhanced survival of T cells and increased antileukemic efficacy in vivo. *Mol. Ther.* **17**, 1453–1464 (2009). 19384291 10.1038/mt.2009.83 PMC2805264

[29] Beckett, A. N. et al. CD47 expression is critical for CAR T-cell survival in vivo. *J. Immunother. Cancer* **11**, e005857 (2023). 10.1136/jitc-2022-005857 PMC10016274 36918226

[30] Komori, S. et al. CD47 promotes peripheral T cell survival by preventing dendritic cell-mediated T cell necroptosis. *Proc. Natl Acad. Sci. USA* **120**, e2304943120 (2023). 37549290 10.1073/pnas.2304943120 PMC10440595

[31] Miao, H. et al. EGFRvIII-specific chimeric antigen receptor T cells migrate to and kill tumor deposits infiltrating the brain parenchyma in an invasive xenograft model of glioblastoma. *PLoS ONE* **9**, e94281 (2014). 24722266 10.1371/journal.pone.0094281 PMC3983153

[32] Abbott, R. C. et al. Novel high-affinity EGFRvIII-specific chimeric antigen receptor T cells effectively eliminate human glioblastoma. *Clin. Transl. Immunol.* **10**, e1283 (2021). 10.1002/cti2.1283 PMC8106904 33976881

[33] Allen, F. et al. CCL3 enhances antitumor immune priming in the lymph node via IFNgamma with dependency on natural killer cells. *Front. Immunol.* **8**, 1390 (2017). 29109732 10.3389/fimmu.2017.01390 PMC5660298

[34] Zhang, M. et al. Anti-CD47 treatment stimulates phagocytosis of glioblastoma by M1 and M2 polarized macrophages and promotes M1 polarized macrophages in vivo. *PLoS ONE* **11**, e0153550 (2016). 27092773 10.1371/journal.pone.0153550 PMC4836698

[35] Rejeski, K. et al. CAR-HEMATOTOX: a model for CAR T-cell-related hematologic toxicity in relapsed/refractory large B-cell lymphoma. *Blood* **138**, 2499–2513 (2021). 34166502 10.1182/blood.2020010543 PMC8893508

[36] McKinsey, G. L. et al. A new genetic strategy for targeting microglia in development and disease. *eLife* **9**, e54590 (2020). 10.7554/eLife.54590 PMC7375817 32573436

[37] Friebel, E. et al. Single-cell mapping of human brain cancer reveals tumor-specific instruction of tissue-invading leukocytes. *Cell* **181**, 1626–1642.e1620 (2020). 32470397 10.1016/j.cell.2020.04.055

[38] Haynes, S. E. et al. The P2Y12 receptor regulates microglial activation by extracellular nucleotides. *Nat. Neurosci.* **9**, 1512–1519 (2006). 17115040 10.1038/nn1805

[39] Keren-Shaul, H. et al. A unique microglia type associated with restricting development of Alzheimer’s disease. *Cell* **169**, 1276–1290.e1217 (2017). 28602351 10.1016/j.cell.2017.05.018

[40] Silvin, A., Qian, J. & Ginhoux, F. Brain macrophage development, diversity and dysregulation in health and disease. *Cell Mol. Immunol.* **20**, 1277–1289 (2023). 37365324 10.1038/s41423-023-01053-6 PMC10616292

[41] Amorim, A. et al. IFNgamma and GM-CSF control complementary differentiation programs in the monocyte-to-phagocyte transition during neuroinflammation. *Nat. Immunol.* **23**, 217–228 (2022). 35102344 10.1038/s41590-021-01117-7

[42] Hammond, T. R., Robinton, D. & Stevens, B. Microglia and the brain: complementary partners in development and disease. *Annu. Rev. Cell Dev. Biol.* **34**, 523–544 (2018). 30089221 10.1146/annurev-cellbio-100616-060509

[43] Hagemeyer, N. et al. Microglia contribute to normal myelinogenesis and to oligodendrocyte progenitor maintenance during adulthood. *Acta Neuropathol.* **134**, 441–458 (2017). 28685323 10.1007/s00401-017-1747-1 PMC5951721

[44] Li, Q. et al. Developmental heterogeneity of microglia and brain myeloid cells revealed by deep single-cell RNA sequencing. *Neuron* **101**, 207–223.e210 (2019). 30606613 10.1016/j.neuron.2018.12.006 PMC6336504

[45] Schulz, D., Severin, Y., Zanotelli, V. R. T. & Bodenmiller, B. In-depth characterization of monocyte-derived macrophages using a mass cytometry-based phagocytosis assay. *Sci. Rep.* **9**, 1925 (2019). 30760760 10.1038/s41598-018-38127-9 PMC6374473

[46] Fossum, E. et al. Targeting antigens to different receptors on conventional type 1 dendritic cells impacts the immune response. *J. Immunol.* **205**, 661–673 (2020). 32591401 10.4049/jimmunol.1901119

[47] Alghamri, M. S. et al. Targeting neuroinflammation in brain cancer: uncovering mechanisms, pharmacological targets, and neuropharmaceutical developments. *Front. Pharm.* **12**, 680021 (2021). 10.3389/fphar.2021.680021 PMC8167057 34084145

[48] Chen, H. et al. Delivery of CD47 blocker SIRPalpha-Fc by CAR-T cells enhances antitumor efficacy. *J. Immunother. Cancer* **10**, e003737 (2022). 10.1136/jitc-2021-003737 PMC8811602 35110357

[49] Lu, Q. et al. Delivery of CD47-SIRPalpha checkpoint blocker by BCMA-directed UCAR-T cells enhances antitumor efficacy in multiple myeloma. *Cancer Lett.* **585**, 216660 (2024). 38266806 10.1016/j.canlet.2024.216660

[50] Batool, S. M. et al. Highly sensitive EGFRvIII detection in circulating extracellular vesicle RNA of glioma patients. *Clin. Cancer Res.* **28**, 4070–4082 (2022). 35849415 10.1158/1078-0432.CCR-22-0444 PMC9475243

[51] Sharma, P., Aaroe, A., Liang, J. & Puduvalli, V. K. Tumor microenvironment in glioblastoma: current and emerging concepts. *Neurooncol. Adv.* **5**, vdad009 (2023). 36968288 10.1093/noajnl/vdad009 PMC10034917

[52] Theruvath, J. et al. Locoregionally administered B7-H3-targeted CAR T cells for treatment of atypical teratoid/rhabdoid tumors. *Nat. Med.* **26**, 712–719 (2020). 32341579 10.1038/s41591-020-0821-8 PMC7992505

[53] Agliardi, G. et al. Intratumoral IL-12 delivery empowers CAR-T cell immunotherapy in a pre-clinical model of glioblastoma. *Nat. Commun.* **12**, 444 (2021). 33469002 10.1038/s41467-020-20599-x PMC7815781

[54] Choi, B. D. et al. CAR-T cells secreting BiTEs circumvent antigen escape without detectable toxicity. *Nat. Biotechnol.* **37**, 1049–1058 (2019). 31332324 10.1038/s41587-019-0192-1

[55] Boulch, M. et al. Tumor-intrinsic sensitivity to the pro-apoptotic effects of IFN-gamma is a major determinant of CD4(+) CAR T-cell antitumor activity. *Nat. Cancer* **4**, 968–983 (2023). 37248395 10.1038/s43018-023-00570-7 PMC10368531

[56] Kruse, B. et al. CD4(+) T cell-induced inflammatory cell death controls immune-evasive tumours. *Nature* **618**, 1033–1040 (2023). 37316667 10.1038/s41586-023-06199-x PMC10307640

[57] Wang, D. et al. Glioblastoma-targeted CD4+ CAR T cells mediate superior antitumor activity. *JCI Insight* **3**, e99048 (2018). 10.1172/jci.insight.99048 PMC6012522 29769444

[58] Adusumilli, P. S. et al. Regional delivery of mesothelin-targeted CAR T cell therapy generates potent and long-lasting CD4-dependent tumor immunity. *Sci. Transl. Med.* **6**, 261ra151 (2014). 25378643 10.1126/scitranslmed.3010162 PMC4373413

[59] Bove, C. et al. CD4 CAR-T cells targeting CD19 play a key role in exacerbating cytokine release syndrome, while maintaining long-term responses. *J. Immunother. Cancer* **11**, e005878 (2023). 10.1136/jitc-2022-005878 PMC9809278 36593069

[60] Melenhorst, J. J. et al. Decade-long leukaemia remissions with persistence of CD4(+) CAR T cells. *Nature* **602**, 503–509 (2022). 35110735 10.1038/s41586-021-04390-6 PMC9166916

[61] Schaller, T. H., Batich, K. A., Suryadevara, C. M., Desai, R. & Sampson, J. H. Chemokines as adjuvants for immunotherapy: implications for immune activation with CCL3. *Expert Rev. Clin. Immunol.* **13**, 1049–1060 (2017). 28965431 10.1080/1744666X.2017.1384313 PMC6020048

[62] Davatelis, G. et al. Macrophage inflammatory protein-1: a prostaglandin-independent endogenous pyrogen. *Science* **243**, 1066–1068 (1989). 2646711 10.1126/science.2646711

[63] Tunyasuvunakool, K. et al. Highly accurate protein structure prediction for the human proteome. *Nature* **596**, 590–596 (2021). 34293799 10.1038/s41586-021-03828-1 PMC8387240

[64] Jumper, J. et al. Highly accurate protein structure prediction with AlphaFold. *Nature* **596**, 583–589 (2021). 34265844 10.1038/s41586-021-03819-2 PMC8371605

[65] Nakayashiki, N. et al. Production of a single-chain variable fragment antibody recognizing type III mutant epidermal growth factor receptor. *Jpn. J. Cancer Res.* **91**, 1035–1043 (2000). 11050475 10.1111/j.1349-7006.2000.tb00882.x PMC5926257

[66] Nicholson, I. C. et al. Construction and characterisation of a functional CD19 specific single chain Fv fragment for immunotherapy of B lineage leukaemia and lymphoma. *Mol. Immunol.* **34**, 1157–1165 (1997). 9566763 10.1016/s0161-5890(97)00144-2

[67] Du, L. et al. IL-21 optimizes the CAR-T cell preparation through improving lentivirus mediated transfection efficiency of T cells and enhancing CAR-T cell cytotoxic activities. *Front. Mol. Biosci.* **8**, 675179 (2021). 34179083 10.3389/fmolb.2021.675179 PMC8220804

[68] Ahrne, E. et al. Evaluation and improvement of quantification accuracy in isobaric mass tag-based protein quantification experiments. *J. Proteome Res.* **15**, 2537–2547 (2016). 27345528 10.1021/acs.jproteome.6b00066

[69] Roussel-Queval, A., Rebejac, J., Eme-Scolan, E., Paroutaud, L. A. & Rua, R. Flow cytometry and immunohistochemistry of the mouse dural meninges for immunological and virological assessments. *STAR Protoc.* **4**, 102119 (2023). 36853673 10.1016/j.xpro.2023.102119 PMC9958090

[70] Weber, L. M., Nowicka, M., Soneson, C. & Robinson, M. D. diffcyt: differential discovery in high-dimensional cytometry via high-resolution clustering. *Commun. Biol.* **2**, 183 (2019). 31098416 10.1038/s42003-019-0415-5 PMC6517415

[71] Bankhead, P. et al. QuPath: open source software for digital pathology image analysis. *Sci. Rep.* **7**, 16878 (2017). 29203879 10.1038/s41598-017-17204-5 PMC5715110

[72] Schmidt, U. et al. Cell detection with star-convex polygons. In *Proc. Medical Image Computing and Computer Assisted Intervention – MICCAI 2018*, 265–273 (Springer International Publishing, 2018).

[73] Gut, G., Herrmann, M. D. & Pelkmans, L. Multiplexed protein maps link subcellular organization to cellular states. *Science* **361**, eaar7042 (2018). 10.1126/science.aar7042 30072512

[74] Schindelin, J. et al. Fiji: an open-source platform for biological-image analysis. *Nat. Methods* **9**, 676–682 (2012). 22743772 10.1038/nmeth.2019 PMC3855844

[75] Preibisch, S., Saalfeld, S. & Tomancak, P. Globally optimal stitching of tiled 3D microscopic image acquisitions. *Bioinformatics* **25**, 1463–1465 (2009). 19346324 10.1093/bioinformatics/btp184 PMC2682522

[76] Thevenaz, P., Ruttimann, U. E. & Unser, M. A pyramid approach to subpixel registration based on intensity. *IEEE Trans. Image Process* **7**, 27–41 (1998). 18267377 10.1109/83.650848

[77] Guiet, R., Burri, O., Chiaruttini, N., Seitz, A. & Eglinger, J. Kheops. 0.1.8 ed. GitHub; 2021.

[78] Ruifrok, A. C. & Johnston, D. A. Quantification of histochemical staining by color deconvolution. *Anal. Quant. Cytol. Histol.* **23**, 291–299 (2001). 11531144

[79] Hogan, S., Kaymak, D., Bartoszek, E. M. & Martins, T. A. Enhancing anti-EGFRvIII CAR T cell therapy against glioblastoma with a paracrine SIRPγ-derived CD47 blocker. 1.0 ed. GitHub; 2024. 10.1038/s41467-024-54129-w PMC11550474 39521782

