# Supplementary material for: Enhancing anti-EGFRvIII CAR T cell therapy against glioblastoma with a paracrine SIRPγ-derived CD47 blocker
Source: Nat Commun. 2024 Nov 9;15:9718. doi: 10.1038/s41467-024-54129-w (PMC11550474; doi:10.1038/s41467-024-54129-w)
Supplement: Supplementary file 1 — Supplementary Information [file 41467_2024_54129_MOESM1_ESM.pdf]

Supplementary Information for

Enhancing anti-EGFRvIII CAR T cell therapy against glioblastoma with a paracrine SIRPγ-derived CD47 blocker

Corresponding authors:
Tomás A. Martins, tomas.martins@unibas.ch
Gregor Hutter, gregor.hutter@usb.ch

The file includes:
Supplementary Figures 1 to 12
Supplementary Tables 1 to 7

Supplementary Figures

Supplementary Figure 1

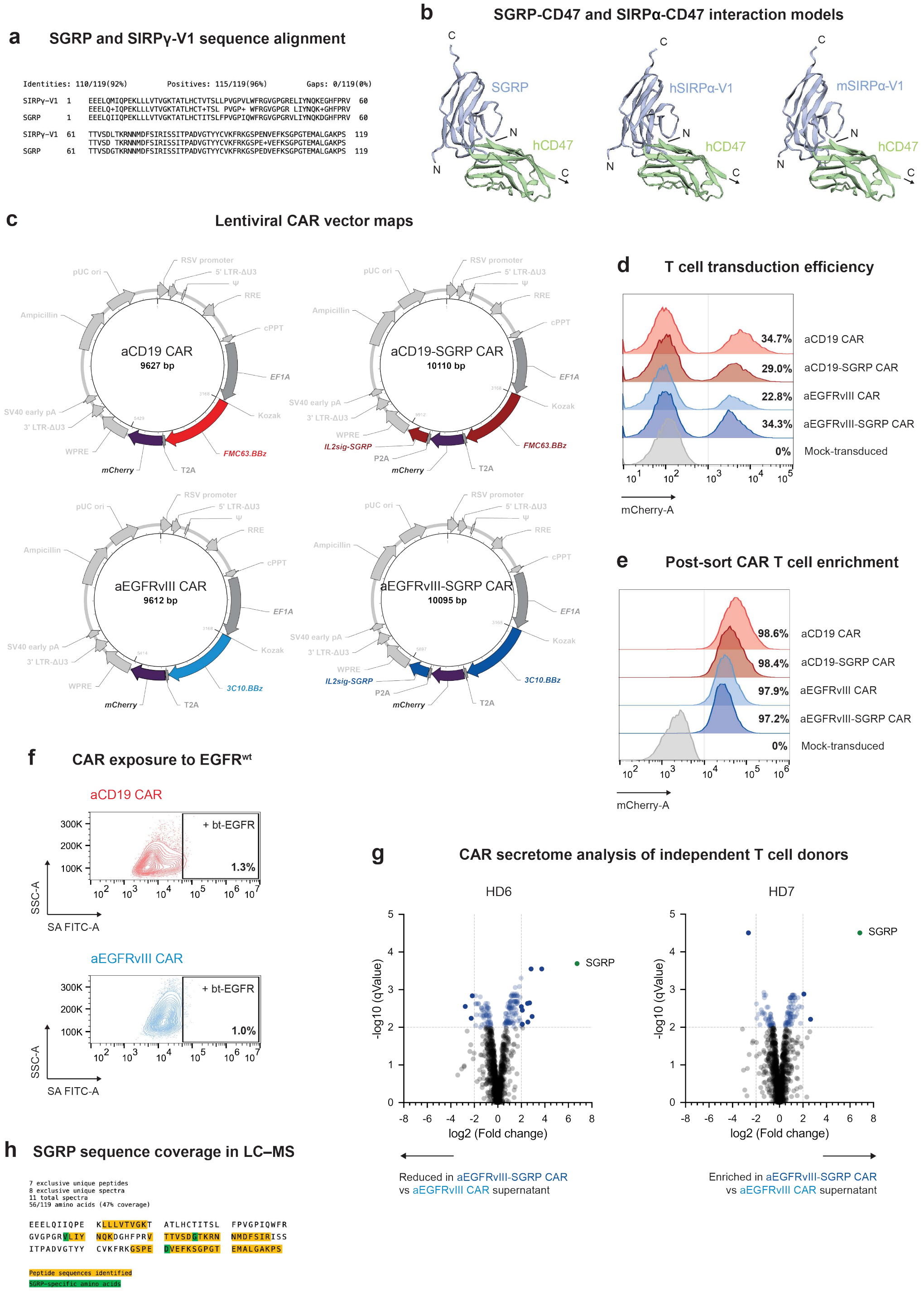

Supplementary Figure 1. Overview of CAR constructs, sequence alignments and LC–MS supernatant analysis. a, SGRP and homologous SIRPγ sequence alignment encompassing the whole length of SGRP (119 AA). **b,** AlphaFold-generated *in silico* modeling displaying predicted protein-protein interactions of SGRP, hSIRPα-V1 and mSIRPα-V1 with hCD47. Amino acid sequence information regarding hCD47, IL2sig, hSIRPα-V1, hSIRPγ-V1, IL2sig-SGRP, mSIRPα-V1, and SGRP are listed in Supplementary Table 1. **c,** Overview of lentiviral vector maps used in the study. Nucleotide sequences for aCD19 (FMC63.BBz), aEGFRvIII (3C10.BBz), aCD19-SGRP and aEGFRvIII-SGRP constructs are listed in Supplementary Table 2. **d,** Representative T cell lentiviral transduction efficiency for the different CAR lentiviral vectors used (n = 3 HDs), based on the percentage of $mCherry^+$ T cells assessed by FC 4 days after transduction (gated on live single cells). Mock-transduced T cells served as controls. The experiment was repeated more than 3 times. **e,** Representative post-sort enrichment of CAR T cells after sorting for $mCherry^+$ cells (n = 3 HDs). These were subsequently expanded and used for downstream experiments. The experiment was repeated more than 3 times. **f,** aCD19 or aEGFRvIII CAR T cells were exposed to wt-EGFR using biotinylated (bt) EGFR recombinant protein. No binding of CARs to bt-EGFR by FC analysis of streptavidin (SA)-FITC could be detected (n = 2 HDs). **g,** Volcano plots of healthy-donor (HD)-specific differential supernatant secretome analysis of aEGFRvIII-SGRP CAR vs aEGFRvIII CAR T cells from HD6 and HD7. Log2 (fold change) indicates the mean expression level for each protein. Each dot represents one protein. The -log10 (qValue) represents the adjusted significance level for each protein. SGRP (green dot) is highly enriched in aEGFRvIII-SGRP CAR T cell-conditioned media from both HDs. The raw expression data is available as Supplementary Data 1. **h,** Representation of SGRP sequence coverage in LC–MS. SGRP was identified by 7 exclusive unique peptides and 8 exclusive unique spectra covering 47% of the 119 AA sequence. Source data are provided as a Source Data file.

Supplementary Figure 2

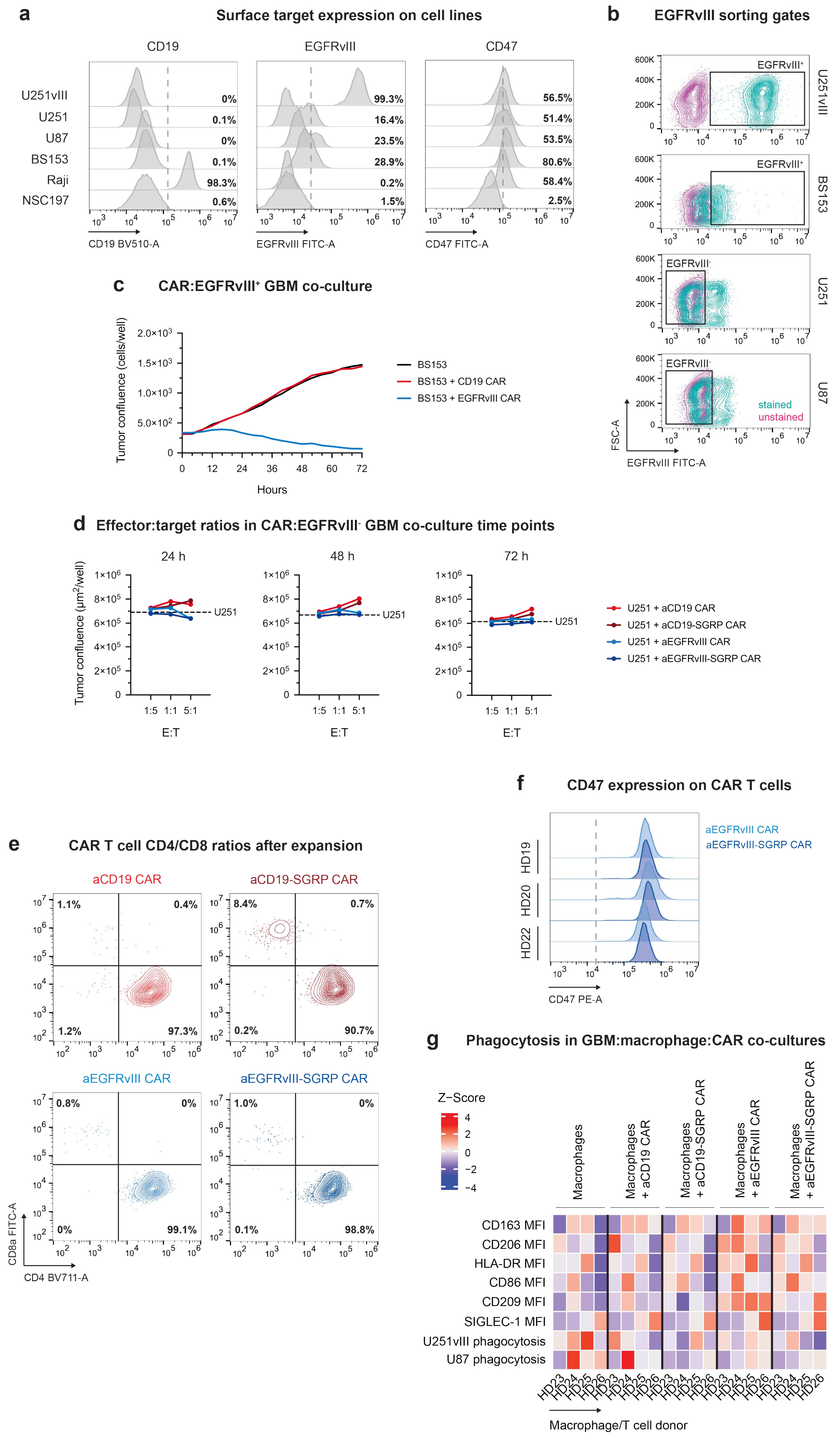

Supplementary Figure 2. *In vitro* characterization of tumor cell lines, CAR T cell efficacy, and CD4/CD8 phenotyping. a, FC representation of surface target expression of tumor cell lines U251vIII, U251, U87, BS153, Raji, and neural stem cell line NSC197 serving as a negative control. **b,** FC gates showing the EGFRvIII-stained (teal) populations sorted for subsequent *in vitro* and *in vivo* experiments encompassing $EGFRvIII^+$ U251vIII or BS153 and $EGFRvIII^-$ U251 or U87 cells. **c,** 72 h time-lapse co-culture experiment of endogenously $EGFRvIII^+$ BS153 cells with aCD19 or aEGFRvIII CARs, respectively. Tumor confluence (cells/well) is plotted over time. Curves represent the mean of duplicate measurements. **d,** Tumor confluence (green nuclei per well) in co-cultures of $EGFRvIII^-$ U251 with either aCD19, aCD19-SGRP, aEGFRvIII, or aEGFRvIII-SGRP CAR T cells in 3 different effector-target ratios at 24, 48 and 72 h timepoints. Dots represent the mean of duplicate measurements. Dashed lines represent the mean confluence in control wells with only U251 cells. **e,** FC assessment of CD4/CD8 T cell phenotypes of aCD19, aCD19-SGRP, aEGFRvIII, and aEGFRvIII-SGRP CAR T cells used in this study (gated on live singlets) at the time of experimental use. One representative healthy donor CAR T cell batch is shown (n = 3 HDs) . **f,** FC representation of surface CD47 expression of aEGFRvIII CAR and aEGFRvIII-SGRP CAR T cells from 3 HDs. Unstained T cells served as a negative control. **g,** FC assessment of phagocytosis and macrophage polarization/effector function in co-cultures of EGFRvIII-mosaic tumor cells with donor-matched macrophages and CAR T cells from 4 HDs. Heatmap showing the MFI of markers and the fractions of phagocytosed U87 and U251vIII cells. Gated on $CD11b^+$ cells. All differences between conventional CARs and SGRP-secreting CARs were not statistically significant using a two-sided one-way ANOVA with Tukey's multiple comparisons. Source data are provided as a Source Data file.

Supplementary Figure 3

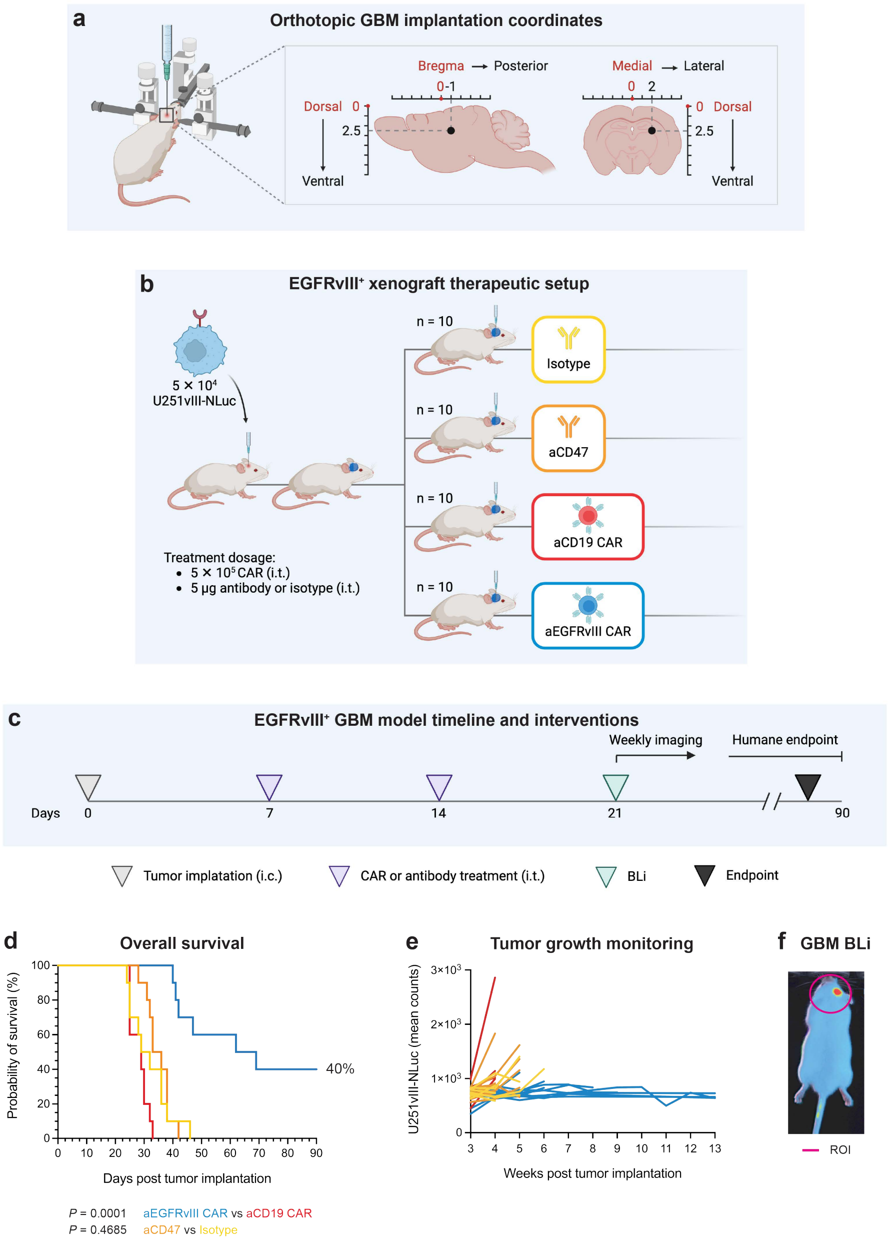

Supplementary Figure 3. Setup and results of orthotopic EGFRvIII-homogeneous GBM *in vivo* experiments. a, Coordinates (in mm) of orthotopic GBM implantation in adult NSG mice. **b,** Overview of the experimental setup of the EGFRvIII$^+$ xenograft GBM tumor model and subsequent monotherapeutic CAR or antibody treatments (n = 10 per group). **c,** Animals were treated with either i.t. CAR T cells or antibodies 7 and 14 days after orthotopic tumor implantation with U251vIII-NLuc tumor cells, followed by BLi and scoring until the humane endpoint was reached. **a-c,** Created in BioRender. Hutter, G. (2024) BioRender.com/e50z019. **d,** Kaplan–Meier plot of overall survival (in days). Two-sided log-rank tests were used to compare the indicated treatment/control groups. **e,** Tumor progression in EGFRvIII$^+$ xenografts was monitored using BLi time course imaging with FFz substrate (in weeks). **d,e,** The data were pooled from two independent experiments. **f,** Head ROI shape defined for all BLi measurements throughout the study. Source data are provided as a Source Data file.

Supplementary Figure 4

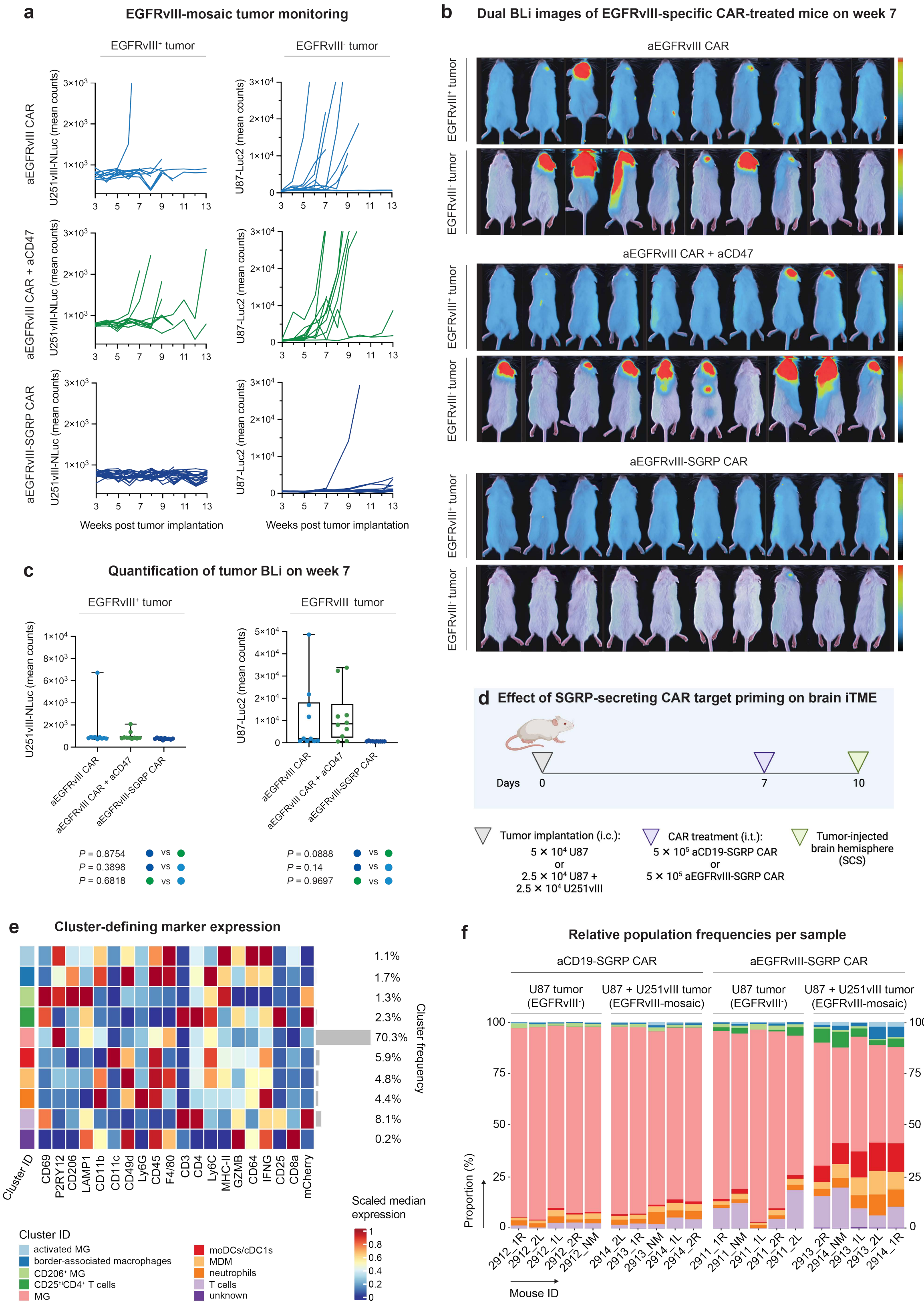

Supplementary Figure 4. Overview of EGFRvIII-mosaic GBM *in vivo* bioluminescence readouts and effect of on-/off-target SGRP-secreting CARs on brain iTME modulation. a, Cumulative differential monitoring in weeks by BLi for both grafted mosaic EGFRvIII^{+} and EGFRvIII^{-} brain tumors per experimental condition. U251vIII^{+} tumor growth was measured by luminescence elicited by FFz, whereas growth of U87vIII^{-} tumors was detected by D-luciferin luminescence. Curves end whenever the humane endpoint was reached. **b,** Overlay images of dual BLi studies of aEGFRvIII CAR-, aEGFRvIII CAR + aCD47- and aEGFRvIII-SGRP CAR-treated animals on week 7, highlighting the suppression of EGFRvIII^{-} tumors in aEGFRvIII-SGRP CAR-treated animals, for which representative images are shown. Scale of EGFRvIII^{+} (NLuc) tumors: 500-7000 counts; Scale of EGFRvIII^{-} (Luc2) tumors: 500-15000 counts. **a,b,** The data were pooled from three independent experiments (aEGFRvIII-SGRP CAR n = 19; others n = 10). **c,** Quantification of BLi signal intensities (mean photon counts) as a surrogate for tumor burden for EGFRvIII^{+} and EGFRvIII^{-} tumors from (**b**) at 7 weeks after tumor implantation. Each dot represents one animal (n = 10 per condition). The data were pooled from three independent experiments. The boxes' central line represents the median, with the 75th percentile at the upper bound, the 25th percentile at the lower bound, and the whiskers representing all samples from min to max values. Statistics were performed using two-sided one-way ANOVA with Tukey's multiple comparisons. **d,** Outline of *in vivo* experiment comparing the effect of aCD19-SGRP CAR and aEGFRvIII-SGRP CAR on the GBM iTME in the context of EGFRvIII^{-} or EGFRvIII-mosaic intracerebral tumors. Created in BioRender. Hutter, G. (2024) BioRender.com/i16d744. **e,** Heatmap of scaled median cluster-defining cell lineage marker expression across all animals (n = 5 per condition) on 10 immune cell populations indicated on the left y-axis. Cluster frequency is indicated on the right y-axis. **f,** Frequency of immune cell clusters in the tumor-injected brain hemispheres of U87 or U87+U251vIII tumor-engrafted animals treated with aCD19-SGRP CAR or aEGFRvIII-SGRP CAR (n = 5 per condition). The population color code is identical to the 'cluster ID' from (**e**). Source data are provided as a Source Data file.

Supplementary Figure 5

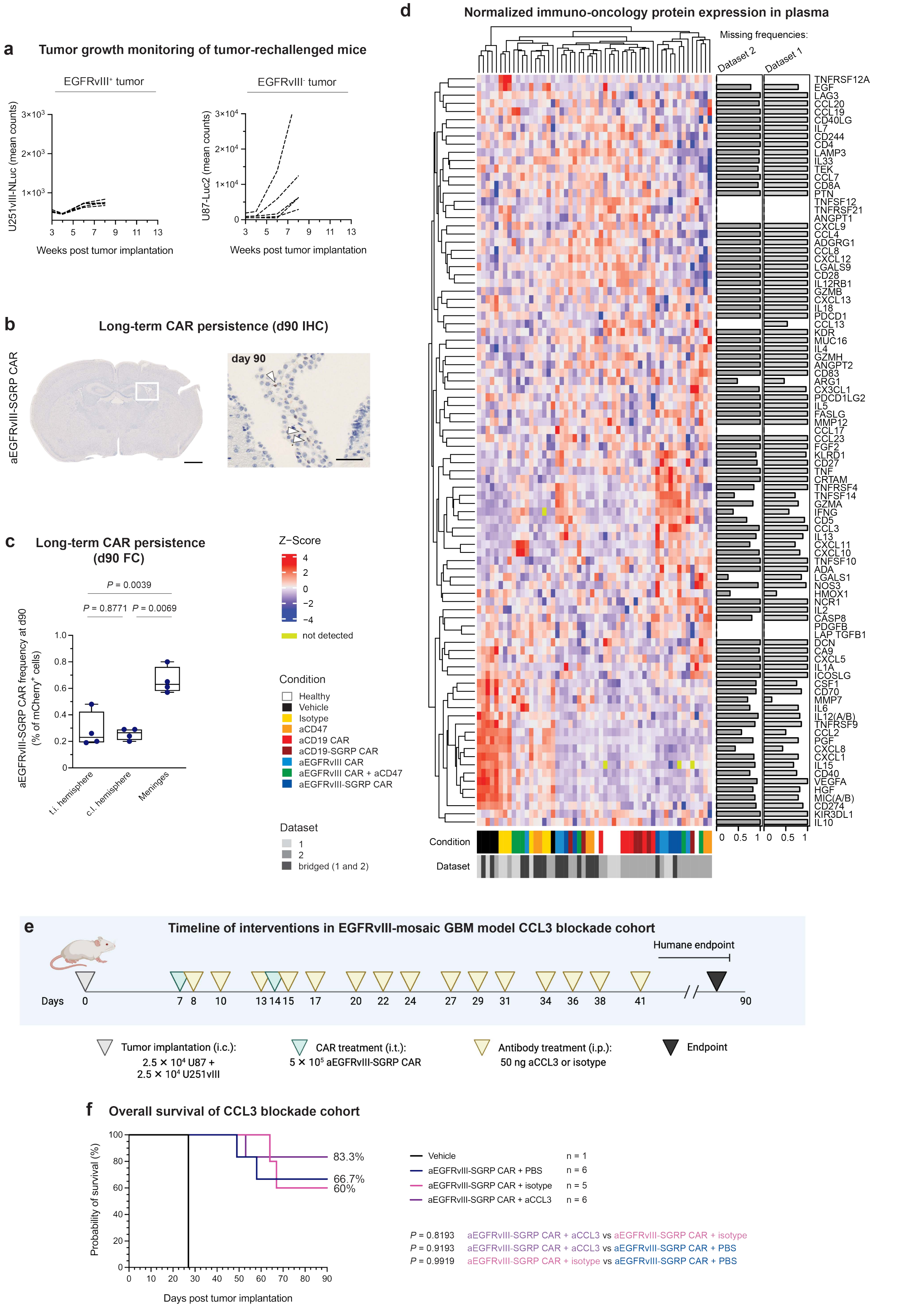

Supplementary Figure 5. Assessment of long-term CAR T cell persistence in the brain, immune-targeted post-therapeutic plasma proteomics, and *in vivo* CCL3 blockade. a, Dual BLi monitoring (in weeks) of EGFRvIII-mosaic tumor-rechallenged mice (n = 5). **b,** IHC micrograph of a brain section from an aEGFRvIII-SGRP CAR-treated mouse tumor-free until day 90 post-tumor implantation (n = 3), showing DAPI-stained cell nuclei (blue) and human CD3-stained CAR T cells (brown); *Left:* Overview of brain section; Scale bar: 1 mm; *Right:* Magnified region defined by the white insert; White arrowheads indicate CAR T cells; Scale bar: 50 µm. **c,** Frequency of mCherry-labeled aEGFRvIII-SGRP CAR T cells gated on single live cells (Zombie Aqua^{-}) in the brains of tumor-free animals on day 90 post-tumor implantation. Three brain regions were processed separately: tumor-implanted (t.i.) hemisphere, contralateral (c.l.) hemisphere, and meninges. Each dot represents one animal (n = 4). The boxes' central line represents the median, with the 75th percentile at the upper bound, the 25th percentile at the lower bound, and the whiskers representing all samples from min to max values. Statistics were performed using a two-sided one-way ANOVA with Tukey's multiple comparisons. **d,** Clustered heatmap of immuno-oncology proteins in plasma on day 15 post-tumor implantation (n = 6 per condition). Datasets were bridged and normalized (grey scale). The y-axis depicts individual protein expression; each cell represents the Z-score of the row's measurements, clustered by Euclidean distance. The bar plot on the right shows the proportion of protein measurements below the limit of detection (LOD) for each dataset. The data were pooled from two independent experiments with 16 overlapping biological replicates. **e,** CCL3 blockade experimental schedule (vehicle n = 1; aEGFRvIII-SGRP CAR + isotype n = 5; others n = 6). Animals were treated i.t. on days 7 and 14 after i.c. implantation of EGFRvIII-mosaic tumors. Tumor and CAR T cells were injected using the same stereotactic coordinates. Systemic aCCL3 therapy was administered 3x weekly for 5 weeks starting on day 8. CAR T cell dose: 5×10^5 cells delivered i.t.; Antibody dose: 50 ng delivered i.p. Created in BioRender. Hutter, G. (2024) BioRender.com/y18i859. **f,** Kaplan–Meier plot of overall survival (in days). Two-sided log-rank tests compared treatment/control groups. Source data are provided as a Source Data file.

Supplementary Figure 6

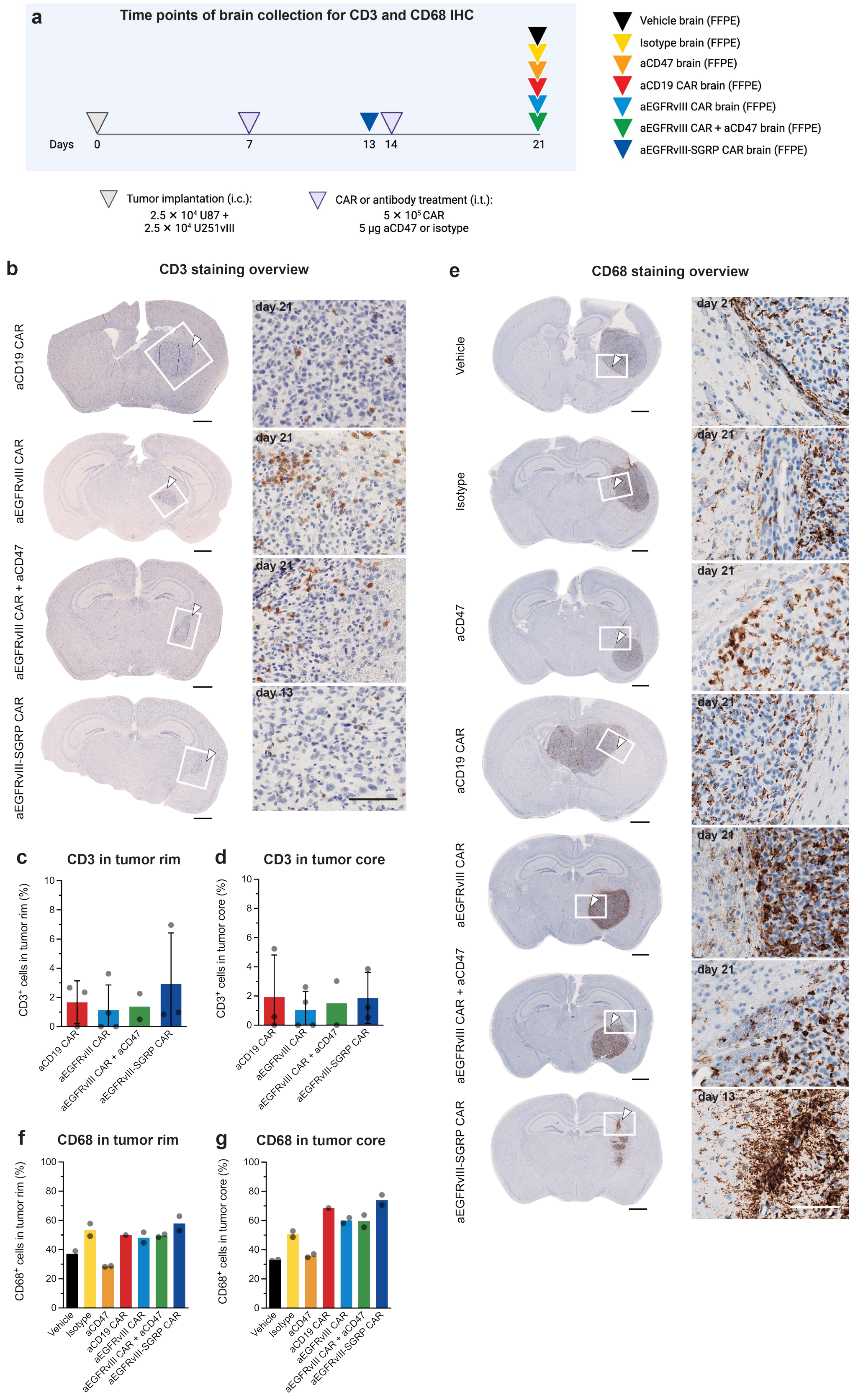

Supplementary Figure 6. Human CD3 and murine CD68 immunohistochemistry of post-therapy intermediate time point brains. a, Post-therapeutic, intermediate brain collection time points for conventional immunohistochemistry per experimental condition. Created in BioRender. Hutter, G. (2024) BioRender.com/c36v073. **b,** Representative IHC micrographs of mouse brain sections per therapeutic condition at the indicated time points showing DAPI-stained cell nuclei (blue) and human CD3-stained grafted CAR T cells (brown) within the tumor core, tumor rim, and adjacent brain tissue; *Left column:* Overview of tumor-burdened brain sections per condition; Scale bars: 1 mm; *Right column:* Close-up of the regions defined by the inserts on a representative tumor-burdened brain per condition; White arrowheads indicate the magnified tumor-brain interface regions on the overview of each condition; Scale bar: 100 µm. **c,** Quantification of $CD3^+$ cells in the tumor rim. **d,** Quantification of $CD3^+$ cells in the tumor core. **c,d,** Each dot represents one animal (aEGFRvIII CAR n = 4; aEGFRvIII CAR + aCD47 n = 2; others n = 3). Data are presented as mean values (bar tops) ± SD. **e,** Representative IHC micrographs of mouse brain sections per therapeutic condition at the indicated time points showing DAPI-stained cell nuclei (blue) and mouse CD68-stained tumor-infiltrating GAMs (brown) within the tumor core, tumor rim, and adjacent brain tissue; *Left column:* Overview of tumor-burdened brain sections per condition; Scale bars: 1 mm; *Right column:* Close-up of the regions defined by the inserts on a representative tumor-burdened brain per condition; White arrowheads indicate the magnified tumor-brain interface regions on the overview of each condition; Scale bar: 100 µm. **b,e,** The data were pooled from two independent experiments, and stainings were performed individually for each histological slide (n = 3 per condition). **f,** Quantification of $CD68^+$ cells in the tumor rim. **g,** Quantification of $CD68^+$ cells in the tumor core. **f,g,** Each dot represents one animal (aCD19 CAR n= 1; others n = 2). Data are presented as mean values (bar tops). Source data are provided as a Source Data file.

Supplementary Figure 7

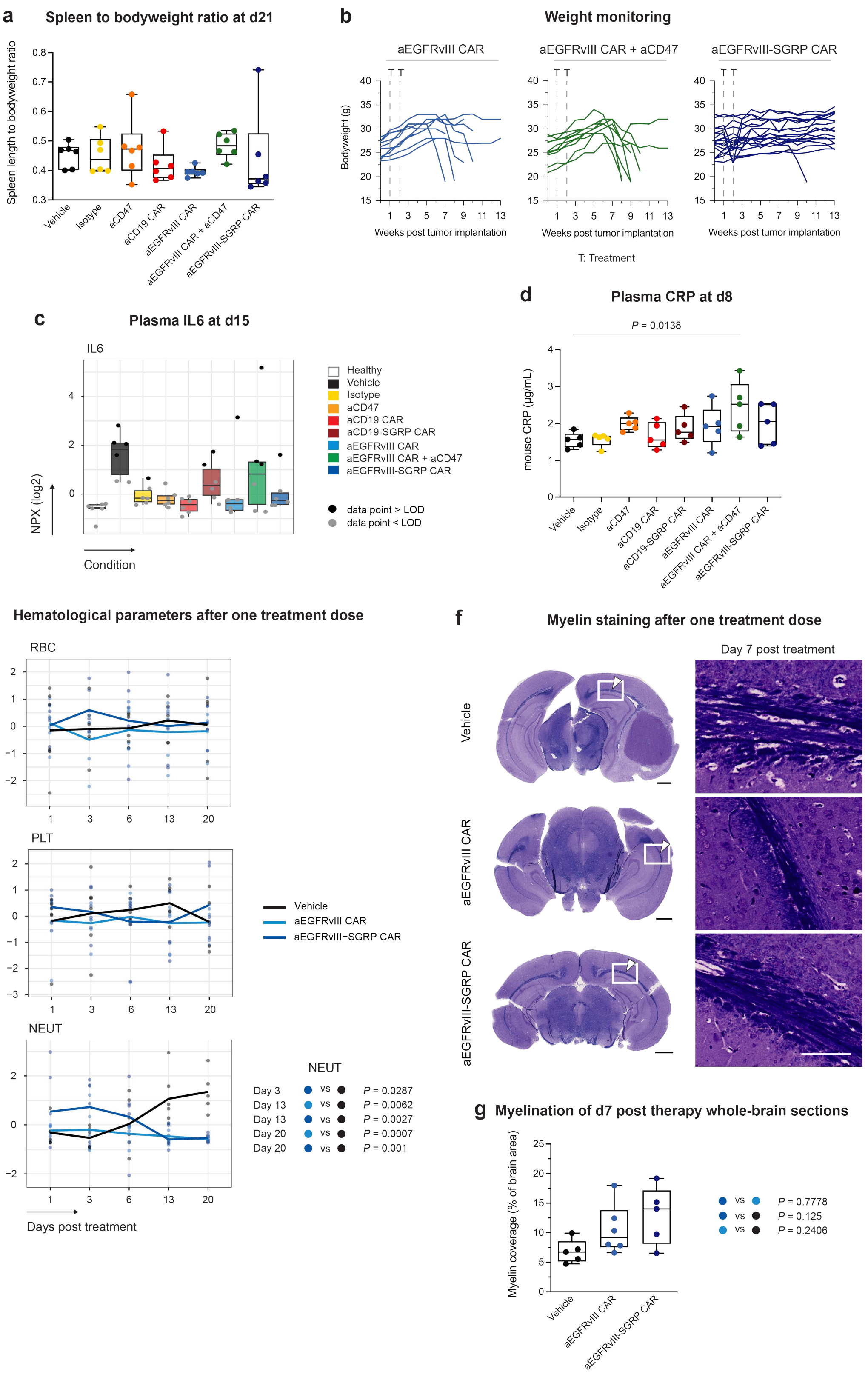

Supplementary Figure 7. Treatment toxicity assessment in post-therapy intermediate time point mouse tissues. a, Spleen-to-bodyweight ratio was assessed on day 21 post-tumor implantation after treatments on days 7 and 14 (n = 6 per condition). No significant differences between treatment groups and vehicle were found. **b,** Weekly weight monitoring of animals in the EGFRvIII-mosaic GBM survival experiment for aEGFRvIII CAR, aEGFRvIII CAR + aCD47, and aEGFRvIII-SGRP CAR groups (aEGFRvIII-SGRP CAR n = 19; others n = 10). Dashed lines indicate treatments in weeks 1 and 2 post-tumor implantation. **c,** Box plots display normalized protein expression (NPX) of IL6 in plasma on day 15 post-tumor implantation. Each data point represents one animal (n = 6 per condition). The definition of boxes and the statistical test used are identical to Fig. 4c; All comparisons were non-significant. **d,** Mouse CRP in plasma on day 8 post-tumor implantation (n = 5 per condition). Only the aEGFRvIII CAR + aCD47 group differed significantly from vehicle. **e,** Monitoring of erythrocyte (RBC), platelet (PLT), and neutrophil (NEUT) counts up to 20 days post-single treatment (n = 6 per condition), normalized to Z-scores. Two-sided mixed-effects modeling (lme4 in R) assessed interactions between day and condition, with sample_ID as a random effect. Post-hoc comparisons (emmeans in R) evaluated time point differences without multiple comparison adjustments. Significant NEUT differences were noted on days 3, 13, and 20. **f,** Representative luxol-stained micrographs of mouse brain sections on day 7 post-treatment (aEGFRvIII CAR n = 6; others n = 5), showing myelin density; *Left column:* Overview of representative brain sections per condition; Scale bars: 1 mm; *Right column:* Magnified regions of the corpus callosum defined by the white arrowheads; Scale bar: 100 µm. **g,** Quantification of myelin-covered brain area on day 7 post-treatment (aEGFRvIII CAR n = 6; others n = 5). No significant differences were observed. **a-g,** Tumors were implanted i.c. (2.5 x 10^4 U87 and 2.5 x 10^4 U251vIII cells); Treatments included 5 x 10^5 CAR T cells and/or 5 µg antibody injected i.t. The data were pooled from two independent experiments, and stainings were performed individually for each histological slide. **a,d,g,** The definition of boxes and statistical tests used are identical to Fig. 5b. Source data are provided as a Source Data file.

Supplementary Figure 8

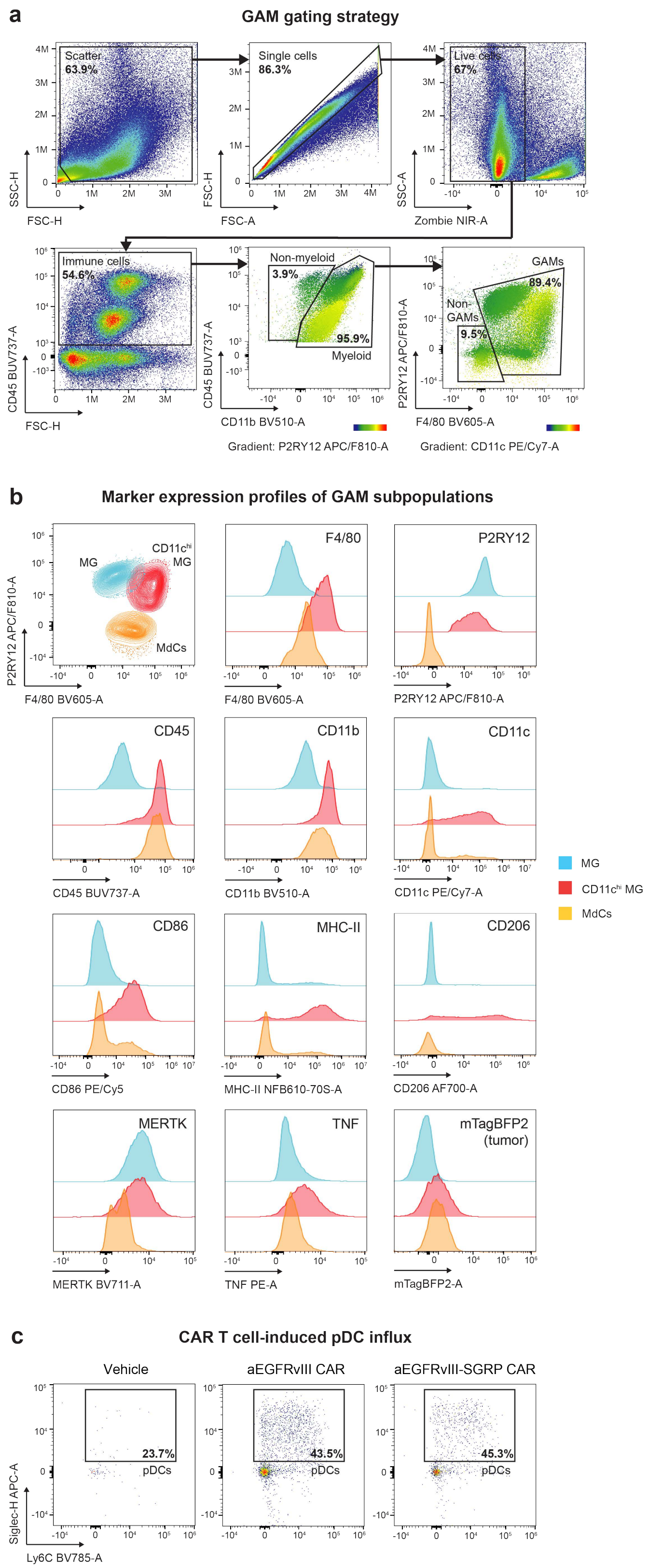

Supplementary Figure 8. Gating strategy and conventional analysis of myeloid cell-targeted spectral flow cytometry. a, Representative pseudocolor plots of the gating strategy applied to isolate single, live (Zombie NIR$^-$), immune (CD45$^+$) cells for the analysis of Fig. 6b-e. Immune cells were further subgated into myeloid (CD11b$^+$) or non-myeloid (CD11b$^-$) populations; The color bar insert represents the MFI of P2RY12 expression overlay on the plot; Myeloid cells were further subgated into GAMs or non-GAMs, as defined by P2RY12 and F4/80 expression; The color bar insert represents the MFI of CD11c expression overlay on the plot. **b,** Histograms of surface and intracellular marker expression in GAM subpopulations defined as MG (blue), CD11c^{hi} MG (red), and MdCs (light orange) on a representative CAR-treated brain. **c,** Representative pseudocolor plots per condition of pDC populations defined by Siglec-H positivity in the non-myeloid gate from (**a**).

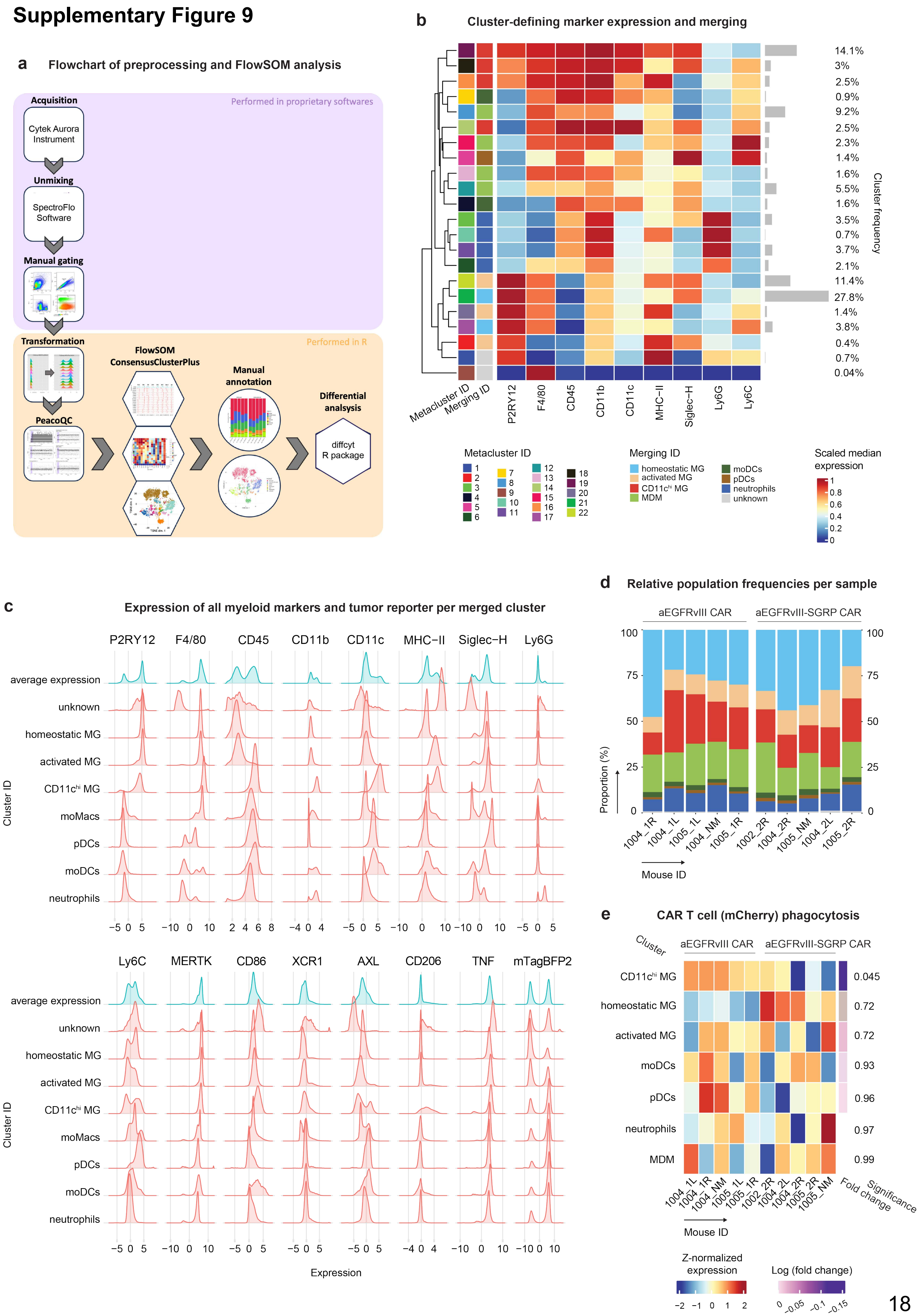

Supplementary Figure 9
a
Flowchart of preprocessing and FlowSOM analysis
Acquisition
Performed in proprietary softwares
Cytek Aurora Instrument
Unmixing
SpectroFlo Software
Manual gating
Transformation
Performed in R
FlowSOM ConsensusClusterPlus
Manual annotation
Differential analysis
PeacoQC
diffcyt R package
b
Cluster-defining marker expression and merging
Metacluster ID
Merging ID
P2RY12
F4/80
CD45
CD11b
CD11c
MHC−II
Siglec−H
Ly6G
Ly6C
Cluster frequency
14.1%
3%
2.5%
0.9%
9.2%
2.5%
2.3%
1.4%
1.6%
5.5%
1.6%
3.5%
0.7%
3.7%
2.1%
11.4%
27.8%
1.4%
3.8%
0.4%
0.7%
0.04%
Merging ID
homeostatic MG
activated MG
CD11c^hi MG
MDM
moDCs
pDCs
neutrophils
unknown
Scaled median expression
c
Expression of all myeloid markers and tumor reporter per merged cluster
P2RY12
F4/80
CD45
CD11b
CD11c
MHC−II
Siglec−H
Ly6G
average expression
unknown
homeostatic MG
activated MG
CD11c^hi MG
moMacs
pDCs
moDCs
neutrophils
Cluster ID
Ly6C
MERTK
CD86
XCR1
AXL
CD206
TNF
mTagBFP2
Expression
d
Relative population frequencies per sample
aEGFRvIII CAR
aEGFRvIII-SGRP CAR
Proportion (%)
1004_1R
1004_1L
1005_1L
1004_NM
1005_1R
1002_2R
1004_2R
1005_NM
1004_2L
1005_2R
Mouse ID
e
CAR T cell (mCherry) phagocytosis
Cluster
CD11c^hi MG
homeostatic MG
activated MG
moDCs
pDCs
neutrophils
MDM
0.045
0.72
0.72
0.93
0.96
0.97
0.99
1004_1L
1004_1R
1004_NM
1005_1L
1005_1R
1002_2R
1004_2L
1004_2R
1005_2R
1005_NM
Fold change
Significance
Mouse ID
Z-normalized expression
Log (fold change)

Supplementary Figure 9. FlowSOM cluster-defining marker expression and relative population frequencies for differential analysis. a, Flowchart of the data acquisition, preprocessing, and FlowSOM analysis. **b,** Heatmap of scaled median cluster-defining cell lineage marker expression on 22 metaclusters merged into 8 immune cell populations indicated on the left y-axis. Cluster frequency is indicated on the right y-axis. **c,** Histograms of marker expression per merged cluster. **d,** Frequency of clusters in aEGFRvIII CAR- and aEGFRvIII-SGRP CAR-treated animals (n = 5 per condition). The population color code is identical to the 'merging ID' from (**b**). **e,** Heatmap of mCherry expression across innate immune cell populations per sample as a surrogate for grafted CAR T cell phagocytosis (n = 5 per condition). Significance cutoffs: false discovery rate = 0.05, log (fold change) = 0.5.

Supplementary Figure 10

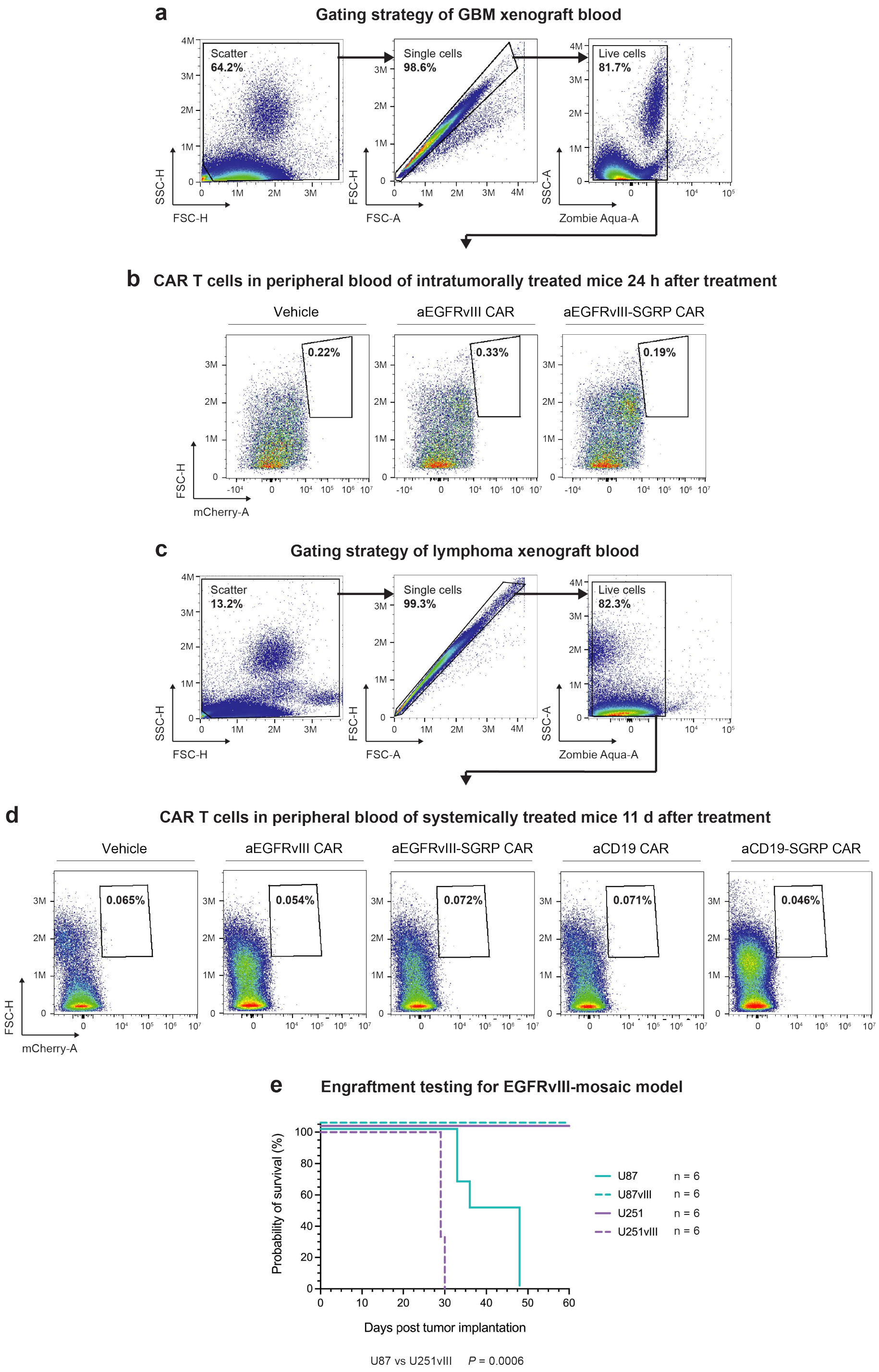

Supplementary Figure 10. CAR T cell detection in peripheral blood and engraftment testing for GBM cell lines. a, Representative pseudocolor plots of the gating strategy applied to isolate single, live (Zombie Aqua^{-}) cells within erythrocyte-depleted peripheral blood from EGFRvIII-mosaic GBM-engrafted mice. **b,** Representative pseudocolor plots derived from individual mouse blood samples per condition collected 24 h after i.t. CAR therapy, displaying the frequency of mCherry^{+} CAR T cells (n = 6 per condition). **c,** Representative pseudocolor plots of the gating strategy applied to isolate single, live (Zombie Aqua^{-}) cells within erythrocyte-rich peripheral blood from CD19^{+} lymphoma-engrafted mice. **d,** Representative pseudocolor plots derived from individual mouse blood samples per condition collected 11 d after i.v. CAR therapy, displaying the frequency of mCherry^{+} CAR T cells (n = 6 per condition). **e,** Kaplan–Meier plot of overall survival (in days). Tumor cells were implanted intracranially (2.5 x 10^{4} per animal; n = 6 per cell line). A two-sided log-rank test compared U87 and U251vIII groups: p = 0.0006. Source data are provided as a Source Data file.

Supplementary Figure 11

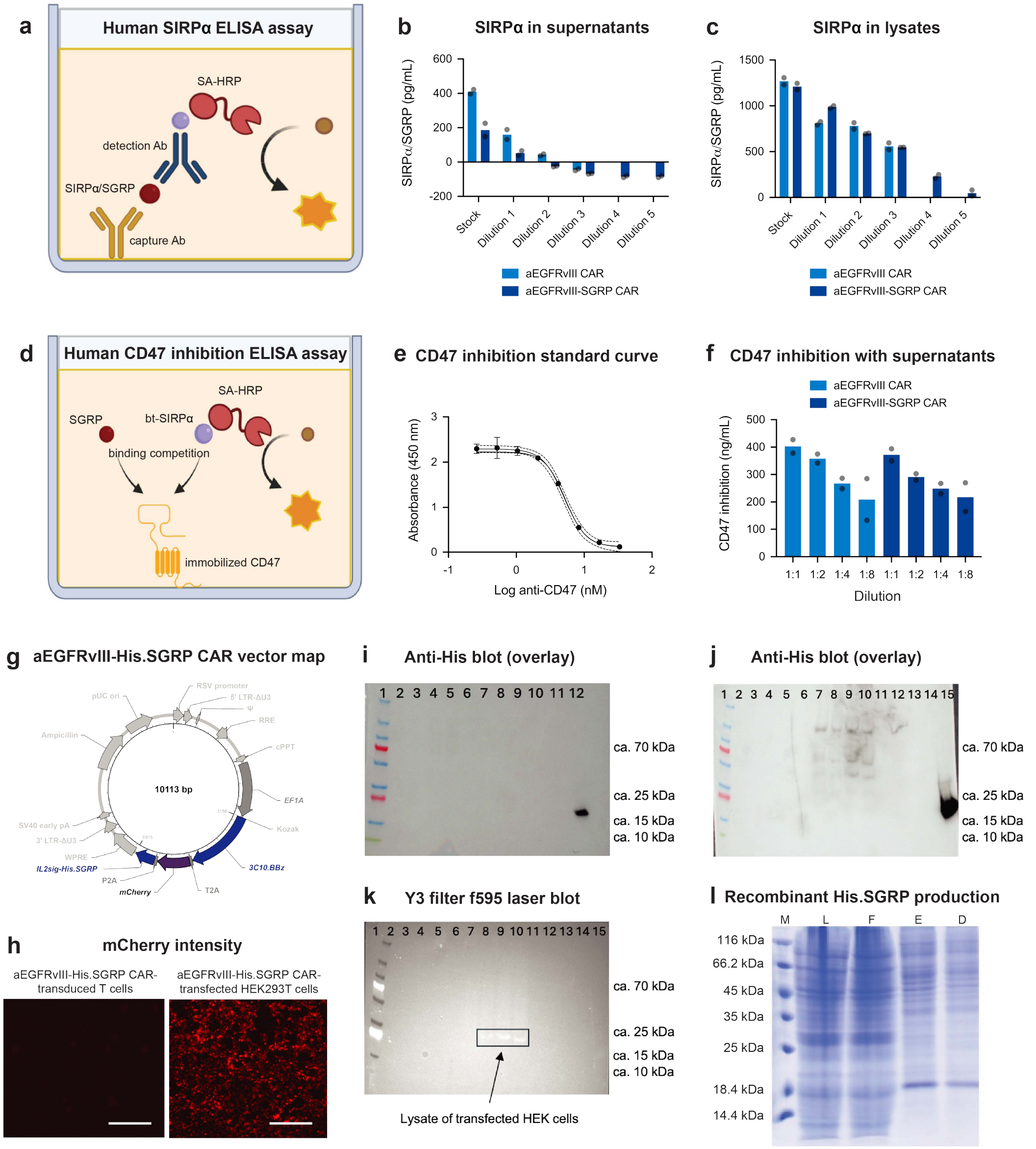

Supplementary Figure 11. Attempts of SGRP detection via LC–MS-alternative methods. a, Schematic representation of a SIRPα ELISA assay. **b,c,** Analysis of supernatants (**b**) and lysates (**c**) of CAR T cells secreting SGRP (aEGFRvIII-SGRP CAR) and negative control (aEGFRvIII CAR) at different dilution factors. **d,** Schematic representation of the CD47 inhibition ELISA assay. **a,d,** Created in BioRender. Sabatino, V. (2023) BioRender.com/v17d531. **e,** Standard curve with varying concentration of anti-CD47 antibody and fixed concentration of biotinylated (bt)-SIRPα. **f,** analysis of CAR T cell culture supernatants of aEGFRvIII-SGRP CAR and aEGFRvIII CAR at different dilution factors. **b,c,f,** Conditions were performed in technical duplicates. Each dot represents one measurement. Data are presented as mean values (bar tops). **g,** Lentiviral vector map of aEGFRvIII-SGRP CAR with His-tagged SGRP (aEGFRvIII-His.SGRP CAR). Nucleotide sequences are listed in Supplementary Table 2. **h,** Representative fluorescence microscopy images (RFP channel) of mCherry^{+}-sorted T cells 22 days after transduction or HEK293T cells 48 h after transfection (n = 3); Scale bars: 500 µm. **i,** Anti-His blot of transduced T cell lysates and supernatants (n = 2 per condition); SGRP expected molecular weight: ca. 15 kDa; Lanes: (1) Ladder; (2) aEGFRvIII-SGRP CAR supernatant; (3) aEGFRvIII-His.SGRP CAR supernatant; (4) aEGFRvIII-SGRP CAR lysate; (5) aEGFRvIII-His.SGRP CAR lysate; (6) His-purified aEGFRvIII-SGRP CAR eluate #1; (7) His-purified aEGFRvIII-His.SGRP CAR eluate #1; (8) His-purified aEGFRvIII-SGRP CAR eluate #2; (9) His-purified aEGFRvIII-His.SGRP CAR eluate #2; (10) His-purified aEGFRvIII-SGRP CAR eluate #3; (11) His-purified aEGFRvIII-His.SGRP CAR eluate #3; (12) dnPD-L1 6xHis positive control. **j,k,** Anti-His and f595 Y3 filter blots (n = 2 per condition); SGRP expected molecular weight: ca. 15 kDa; Lanes: (1) Ladder; (2-6) His-purified supernatants - Control; His.SGRP #1; His.SGRP #2; His.SGRP #3; His.SGRP #4; (7-10) Cell lysates - SGRP control; His.SGRP #1; His.SGRP #2; His.SGRP #3; (11-14) Non-purified supernatants - SGRP control; His.SGRP #1; His.SGRP #2; His.SGRP #3; (15) aPD-L1 His-tag positive control. **l,** Recombinant SGRP production and recovery using an up-scaled mammalian (HEK293T) expression system (n = 1 per condition); SGRP expected molecular weight: ca. 15 kDa; Lanes: (M) marker; (L) loaded raw supernatant; (F) flow-through sample; (E) eluted sample; (D) desalted sample. Source data are provided as a Source Data file.

Supplementary Figure 12

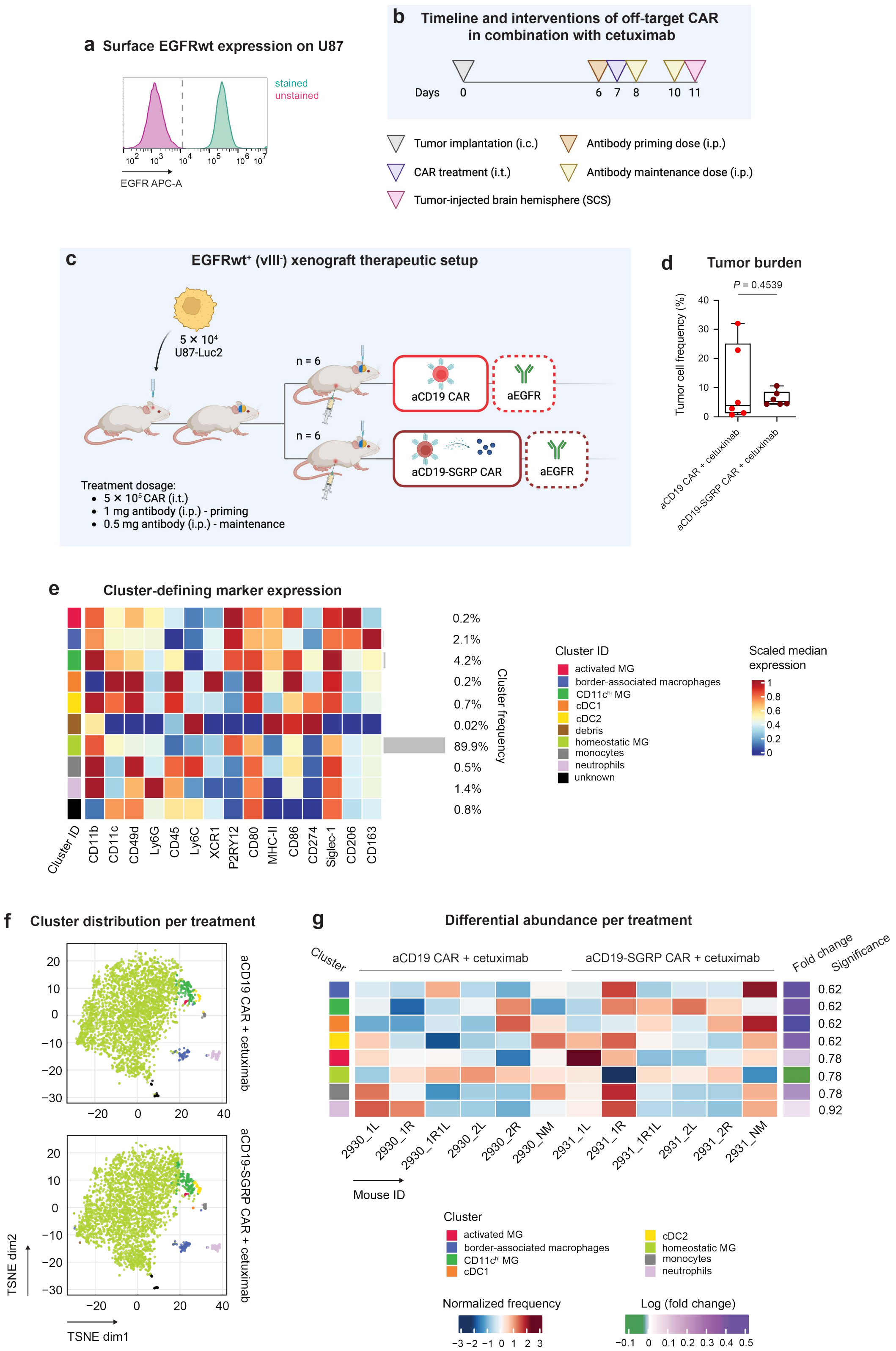

Supplementary Figure 12. Effect of SGRP and opsonizing antibody cetuximab combination on GAM-mediated tumor targeting *in vivo*. a, FC representation of EGFRwt surface expression on U87 tumor cells. **b,** Experimental treatment and monitoring schedule. Animals were treated once with CAR T cells – at 7 days – after intracranial (i.c.) tumor implantation using the same stereotactic coordinates and monitored for clinical signs and bioluminescence imaging (BLi) every 2 days. Systemic aEGFR opsonizing antibody doses were administered on days 6, 8, and 10. Animals were euthanized on day 11 post-tumor implantation. **c,** Experimental setup of orthotopic xenograft experiments in NSG mice implanted with $EGFRvIII^{-}$ $EGFRwt^{+}$ U87 GBM cells and therapeutic cohorts, including local CAR T cell combination with cetuximab (n = 6 per group). CAR T cell dose: 5 x 10^{5} cells delivered i.t.; Antibody dose (priming): 1 mg delivered i.p.; Antibody dose (maintenance): 0.5 mg delivered i.p. **b,c,** Created in BioRender. Hutter, G. (2024) BioRender.com/o80z455. **d,** Frequency of mTagBFP2-labeled U87 tumor cells in the brains of animals treated with aCD19 CAR + cetuximab or aCD19-SGRP CAR + cetuximab combinations; Each dot represents one animal (n = 6 per condition). The boxes' central line represents the median, with the 75th percentile at the upper bound, the 25th percentile at the lower bound, and the whiskers representing all samples from min to max values. A two-sided unpaired t test with Welch's correction was used to compare the two conditions. **e,** Heatmap of scaled median cluster-defining cell lineage marker expression on 10 immune cell populations indicated on the left y-axis. Cluster frequency is indicated on the right y-axis. **f,** TSNE plots (n = 6 per condition) depicting 10 populations manually annotated based on the heatmap from (**e**). The population color code is identical to the 'cluster ID' from (**e**). **g,** Heatmap of the differential abundance of immune cell populations per sample depicting no significant changes between aCD19 CAR + cetuximab and aCD19-SGRP CAR + cetuximab treatments (n = 6 per condition). Significance cutoffs: false discovery rate = 0.05, log (fold change) = 0.5. Source data are provided as a Source Data file.

Supplementary Tables

Supplementary Table 1. Amino acid (AA) and nucleotide (nt) sequences of SGRP and related proteins.

Protein/peptide	Uniprot Ref	AA sequence
Human CD47	Q08722	QLLFNKTKSVEFTFCNDTVVIPCFVTNMEAQNTTEVYVKWKFKGRDI YTFDGALNKSTVPTDFSSAKIEVSQLLKGDASLKMDKSDAVSHTGN YTCEVTELTREGETIIELKYRVVSWFSPNENILIVIFPIFAILLFWGQFG IKTLKYRSGGMDEKTIALLVAGLVITVIVIVGAILFVPGEYSLKNATGLG LIVTSTGILILLHYYVFSTAIGLTSFVIAILVIQVIAYILAVVGLSLCIAACIP MHGPLLISGLSILALAQLLGLVYMKFVASNQKTIQPPRKAVEEPLNAF KESKGMMNDE
Human IL2sig	P60568	MYRMQLLSCIALSLALVTNS
Human SIRPα-V1	P78324	EELQVIQPDKSVLVAAGETATLRCTATSLIPVGPIQWFRGAGPGRELI YNQKEGHFPRVTTVSDLTKRNNMDFSIRIGNITPADAGTYYCVKFRK GSPDDVEFKSG
Human SIRPγ-V1	Q9P1W8	EELQMIQPEKLLLVTVGKTATLHCTVTSLLPVGPVLWFRGVGPGREL IYNQKEGHFPRVTTVSDLTKRNNMDFSIRISSITPADVGTYYCVKFRK GSPENVEFKSG
IL2sig-SGRP	NA	MYRMQLLSCIALSLALVTNSEEELQIIQPEKLLLVTVGKTATLHCTITS LFPVGPIQWFRGVGPGRVLIYNQKDGHFPRVTTVSDGTKRNNMDF SIRISSITPADVGTYYCVKFRKGSPEDVEFKSGPGTEMALGAKPS
Murine SIRPα-V1	P97797	KELKVTQPEKSVSVAAGDSTVLNCTLTSLLPVGPIRWYRGVGPSRL LIYSFAGEYVPRIRNVSDTTKRNNMDFSIRISNVTPADAGIYYCVKFQ KGSSEPDTEIQS
SGRP	NA	EEELQIIQPEKLLLVTVGKTATLHCTITSLFPVGPIQWFRGVGPGRVLI YNQKDGHFPRVTTVSDGTKRNNMDFSIRISSITPADVGTYYCVKFRK GSPEDVEFKSGPGTEMALGAKPS

Protein/peptide	NCBI Ref Seq	nt sequence
Human CD47	NM_001777.4	CAGCTACTATTTAATAAAACAAAATCTGTAGAATTCACGTTTTGTA ATGACACTGTCGTCATTCCATGCTTTGTTACTAATATGGAGGCAC AAAACACTACTGAAGTATACGTAAAGTGGAAATTTAAAGGAAGAG ATATTTACACCTTTGATGGAGCTCTAAACAAGTCCACTGTCCCCA CTGACTTTAGTAGTGCAAAAATTGAAGTCTCACAATTACTAAAAG GAGATGCCTCTTTGAAGATGGATAAGAGTGATGCTGTCTCACAC ACAGGAAACTACACTTGTGAAGTAACAGAATTAACCAGAGAAGGT GAAACGATCATCGAGCTAAAATATCGTGTTGTTTCATGGTTTTCT CCAAATGAAAATATTCTTATTGTTATTTTCCCAATTTTTGCTATACT CCTGTTCTGGGGACAGTTTGGTATTAAAACACTTAAATATAGATC CGGTGGTATGGATGAGAAAACAATTGCTTTACTTGTTGCTGGACT AGTGATCACTGTCATTGTCATTGTTGGAGCCATTCTTTTCGTCCC AGGTGAATATTCATTAAAGAATGCTACTGGCCTTGGTTTAATTGT GACTTCTACAGGGATATTAATATTACTTCACTACTATGTGTTTAGT ACAGCGATTGGATTAACCTCCTTCGTCATTGCCATATTGGTTATT CAGGTGATAGCCTATATCCTCGCTGTGGTTGGACTGAGTCTCTG TATTGCGGCGTGTATACCAATGCATGGCCCTCTTCTGATTTCAGG TTTGAGTATCTTAGCTCTAGCACAATTACTTGGACTAGTTTATATG AAATTTGTGGCTTCCAATCAGAAGACTATACAACCTCCTAGGAAA GCTGTAGAGGAACCCCTTAATGCATTCAAAGAATCAAAAGGAATG ATGAATGATGAA
Human IL2sig	NM_000586.4	ATGTACAGGATGCAACTCCTGTCTTGCATTGCACTAAGTCTTGCA CTTGTCACGAATTCG
Human SIRPα-V1	NM_001040022.1	GAGGAGGAGCTGCAGGTGATTCAGCCTGACAAGTCCGTGTTGG TTGCAGCTGGAGAGACAGCCACTCTGCGCTGCACTGCGACCTCT CTGATCCCTGTGGGGCCCATCCAGTGGTTCAGAGGAGCTGGAC CAGGCCGGGAATTAATCTACAATCAAAAAGAAGGCCACTTCCCC

		CGGGTAACAACTGTTTCAGACCTCACAAAGAGAAACAACATGGA<br>CTTTTCCATCCGCATCGGTAACATCACCCCAGCAGATGCCGGCA<br>CCTACTACTGTGTGAAGTTCCGGAAAGGGAGCCCCGATGACGTG<br>GAGTTTAAGTCTGGA
Human SIRPγ-V1	NM_018556.4	GAGGAGGAGCTACAGATGATTCAGCCTGAGAAGCTCCTGTTGGT<br>CACAGTTGGAAAGACAGCCACTCTGCACTGCACTGTGACCTCCC<br>TGCTTCCCGTGGGACCCGTCCTGTGGTTCAGAGGAGTTGGACCA<br>GGCCGGGAATTAATCTACAATCAAAAAGAAGGCCACTTCCCCAG<br>GGTAACAACAGTTTCAGACCTCACAAAGAGAAACAACATGGACTT<br>TTCCATCCGCATCAGTAGCATCACCCCAGCAGATGTCGGCACAT<br>ACTACTGTGTGAAGTTTCGAAAAGGGAGCCCTGAGAACGTGGAG<br>TTTAAGTCTGGA
IL2sig-SGRP	NA	TACAGGATGCAACTCCTGTCTTGCATTGCACTAAGTCTTGCACTT<br>GTCACGAATTCGGAGGAGGAGCTGCAGATCATCCAGCCAGAGA<br>AGCTGCTGCTGGTGACCGTGGGCAAGACGGCCACTCTTCATTGT<br>ACCATCACCTCTTTGTTCCCCGTGGGTCCCATCCAGTGGTTCCG<br>CGGGGTCGGACCCGGGCGGGTGCTCATCTACAACCAGAAGGAC<br>GGCCACTTTCCTAGGGTCACCACAGTAAGCGACGGCACCAAGC<br>GCAACAATATGGATTTCAGCATTCGCATCTCTTCCATTACTCCGG<br>CGGACGTGGGCACCTATTACTGCGTTAAATTTCGTAAGGGATCC<br>CCGGAAGACGTGGAGTTCAAATCCGGCCCTGGTACGGAGATGG<br>CTCTGGGCGCCAAGCCCTCG
Murine SIRPα-V1	NM_007547.5	AAGGAACTGAAGGTGACTCAGCCTGAGAAATCAGTGTCTGTTGC<br>TGCTGGGGATTCGACCGTTCTGAACTGCACTTTGACCTCCTTGTT<br>GCCGGTGGGACCCATTAGGTGGTACAGAGGAGTAGGGCCAAGC<br>CGGCTGTTGATCTACAGTTTCGCAGGAGAATACGTTCCTCGAATT<br>AGAAATGTTTCAGATACTACTAAGAGAAACAATATGGACTTTTCC<br>ATCCGTATCAGTAATGTCACCCCAGCAGATGCTGGCATCTACTAC<br>TGTGTGAAGTTCCAGAAAGGATCATCAGAGCCTGACACAGAAAT<br>ACAATCT
SGRP	NA	ATGGAGGAGGAGCTGCAGATCATCCAGCCAGAGAAGCTGCTGC<br>TGGTGACCGTGGGCAAGACGGCCACTCTTCATTGTACCATCACC<br>TCTTTGTTCCCCGTGGGTCCCATCCAGTGGTTCCGCGGGGTCGG<br>ACCCGGGCGGGTGCTCATCTACAACCAGAAGGACGGCCACTTT<br>CCTAGGGTCACCACAGTAAGCGACGGCACCAAGCGCAACAATAT<br>GGATTTCAGCATTCGCATCTCTTCCATTACTCCGGCGGACGTGG<br>GCACCTATTACTGCGTTAAATTTCGTAAGGGATCCCCGGAAGAC<br>GTGGAGTTCAAATCCGGCCCTGGTACGGAGATGGCTCTGGGCG<br>CCAAGCCCTCG

Supplementary Table 2. Nucleotide (nt) sequences of overall CAR constructs and individual 3C10.BBz and FMC63.BBz CAR domains with respective SGRP linkage.

CAR construct	Design	bp	nt sequence
aCD19 CAR	FMC63.BBz	2262	ATGGCCTTACCAGTGACCGCCTTGCTCCTGCCGCTGGCCTTG<br>CTGCTCCACGCCGCCAGGCCGGGATCCGACATCCAGATGAC<br>ACAGACTACATCCTCCCTGTCTGCCTCTCTGGGAGACAGAGT<br>CACCATCAGTTGCAGGGCAAGTCAGGACATTAGTAAATATTTA<br>AATTGGTATCAGCAGAAACCAGATGGAACTGTTAAACTCCTG<br>ATCTACCATACATCAAGATTACACTCAGGAGTCCCATCAAGGT<br>TCAGTGGCAGTGGGTCTGGAACAGATTATTCTCTCACCATTA<br>GCAACCTGGAGCAAGAAGATATTGCCACTTACTTTTGCCAAC<br>AGGGTAATACGCTTCCGTACACGTTCGGAGGGGGGACTAAG<br>TTGGAAATAACAGGCTCCACCTCTGGATCCGGCAAGCCCGG<br>ATCTGGCGAGGGATCCACCAAGGGCGAGGTGAAACTGCAGG<br>AGTCAGGACCTGGCCTGGTGGCGCCCTCACAGAGCCTGTCC<br>GTCACATGCACTGTCTCAGGGGTCTCATTACCCGACTATGGT<br>GTAAGCTGGATTCGCCAGCCTCCACGAAAGGGTCTGGAGTG<br>GCTGGGAGTAATATGGGGTAGTGAAACCACATACTATAATTC<br>AGCTCTCAAATCCAGACTGACCATCATCAAGGACAACTCCAA<br>GAGCCAAGTTTTCTTAAAAATGAACAGTCTGCAAACTGATGAC<br>ACAGCCATTTACTACTGTGCCAAACATTATTACTACGGTGGTA<br>GCTATGCTATGGACTACTGGGGTCAAGGAACCTCAGTCACCG<br>TCTCCTCAGCGGCCGCAGCTAGCACCACGACGCCAGCGCCG<br>CGACCACCAACACCGGCGCCCACCATCGCGTCGCAGCCCCT<br>GTCCCTGCGCCCAGAGGCGTGCCGGCCAGCGGCGGGGGGC<br>GCAGTGCACACGAGGGGGCTGGACTTCGCCTGTGATATCTA<br>CATCTGGGCGCCCTTGGCCGGGACTTGTGGGGTCCTTCTCC<br>TGTCACTGGTTATCACCCTTTACTGCAAACGGGGCAGAAAGA<br>AACTCCTGTATATATTCAAACAACCATTTATGAGACCAGTACA<br>AACTACTCAAGAGGAAGATGGCTGTAGCTGCCGATTTCCAGA<br>AGAAGAAGAAGGAGGATGTGAACTGAGAGTGAAGTTCAGCA<br>GGAGCGCAGACGCCCCCGCGTACAAGCAGGGCCAGAACCA<br>GCTCTATAACGAGCTCAATCTAGGACGAAGAGAGGAGTACGA<br>TGTTTTGGACAAGAGACGTGGCCGGGACCCTGAGATGGGGG<br>GAAAGCCGAGAAGGAAGAACCCTCAGGAAGGCCTGTACAAT<br>GAACTGCAGAAAGATAAGATGGCGGAGGCCTACAGTGAGATT<br>GGGATGAAAGGCGAGCGCCGGAGGGGCAAGGGGCACGATG<br>GCCTTTACCAGGGTCTCAGTACAGCCACCAAGGACACCTACG<br>ACGCCCTTCACATGCAGGCCCTGCCCCCTCGCGGAAGCGGA<br>GAGGGCAGGGGAAGTCTTCTAACATGCGGGGACGTGGAGGA<br>AAATCCCGGCCCCATGGTGAGCAAGGGCGAGGAGGATAACA<br>TGGCCATCATCAAGGAGTTCATGCGCTTCAAGGTGCACATGG<br>AGGGCTCCGTGAACGGCCACGAGTTCGAGATCGAGGGCGAG<br>GGCGAGGGCCGCCCCTACGAGGGCACCCAGACCGCCAAGC<br>TGAAGGTGACCAAGGGTGGCCCCCTGCCCTTCGCCTGGGAC<br>ATCCTGTCCCCTCAGTTCATGTACGGCTCCAAGGCCTACGTG<br>AAGCACCCCGCCGACATCCCCGACTACTTGAAGCTGTCCTTC<br>CCCGAGGGCTTCAAGTGGGAGCGCGTGATGAACTTCGAGGA<br>CGGCGGCGTGGTGACCGTGACCCAGGACTCCTCCCTGCAGG<br>ACGGCGAGTTCATCTACAAGGTGAAGCTGCGCGGCACCAAC<br>TTCCCCTCCGACGGCCCCGTAATGCAGAAGAAGACCATGGG<br>CTGGGAGGCCTCCTCCGAGCGGATGTACCCCGAGGACGGC<br>GCCCTGAAGGGCGAGATCAAGCAGAGGCTGAAGCTGAAGGA<br>CGGCGGCCACTACGACGCTGAGGTCAAGACCACCTACAAGG<br>CCAAGAAGCCCGTGCAGCTGCCCGGCGCCTACAACGTCAAC<br>ATCAAGTTGGACATCACCTCCCACAACGAGGACTACACCATC<br>GTGGAACAGTACGAACGCGCCGAGGGCCGCCACTCCACCG<br>GCGGCATGGACGAGCTGTACAAGTAA
aCD19-SGRP CAR	FMC63.BBz	2745	ATGGCCTTACCAGTGACCGCCTTGCTCCTGCCGCTGGCCTTG<br>CTGCTCCACGCCGCCAGGCCGGGATCCGACATCCAGATGAC

			ACAGACTACATCCTCCCTGTCTGCCTCTCTGGGAGACAGAGT<br>CACCATCAGTTGCAGGGCAAGTCAGGACATTAGTAAATATTTA<br>AATTGGTATCAGCAGAAACCAGATGGAACTGTTAAACTCCTG<br>ATCTACCATACATCAAGATTACACTCAGGAGTCCCATCAAGGT<br>TCAGTGGCAGTGGGTCTGGAACAGATTATTCTCTCACCATTA<br>GCAACCTGGAGCAAGAAGATATTGCCACTTACTTTTGCCAAC<br>AGGGTAATACGCTTCCGTACACGTTCGGAGGGGGGACTAAG<br>TTGGAAATAACAGGCTCCACCTCTGGATCCGGCAAGCCCGG<br>ATCTGGCGAGGGATCCACCAAGGGCGAGGTGAAACTGCAGG<br>AGTCAGGACCTGGCCTGGTGGCGCCCTCACAGAGCCTGTCC<br>GTCACATGCACTGTCTCAGGGGTCTCATTACCCGACTATGGT<br>GTAAGCTGGATTCGCCAGCCTCCACGAAAGGGTCTGGAGTG<br>GCTGGGAGTAATATGGGGTAGTGAAACCACATACTATAATTC<br>AGCTCTCAAATCCAGACTGACCATCATCAAGGACAACTCCAA<br>GAGCCAAGTTTTCTTAAAAATGAACAGTCTGCAAACTGATGAC<br>ACAGCCATTTACTACTGTGCCAAACATTATTACTACGGTGGTA<br>GCTATGCTATGGACTACTGGGGTCAAGGAACCTCAGTCACCG<br>TCTCCTCAGCGGCCGCAGCTAGCACCACGACGCCAGCGCCG<br>CGACCACCAACACCGGCGCCCACCATCGCGTCGCAGCCCCT<br>GTCCCTGCGCCCAGAGGCGTGCCGGCCAGCGGCGGGGGGC<br>GCAGTGCACACGAGGGGGCTGGACTTCGCCTGTGATATCTA<br>CATCTGGGCGCCCTTGGCCGGGACTTGTGGGGTCCTTCTCC<br>TGTCACTGGTTATCACCCTTTACTGCAAACGGGGCAGAAAGA<br>AACTCCTGTATATATTCAAACAACCATTTATGAGACCAGTACA<br>AACTACTCAAGAGGAAGATGGCTGTAGCTGCCGATTTCCAGA<br>AGAAGAAGAAGGAGGATGTGAACTGAGAGTGAAGTTCAGCA<br>GGAGCGCAGACGCCCCCGCGTACAAGCAGGGCCAGAACCA<br>GCTCTATAACGAGCTCAATCTAGGACGAAGAGAGGAGTACGA<br>TGTTTTGGACAAGAGACGTGGCCGGGACCCTGAGATGGGGG<br>GAAAGCCGAGAAGGAAGAACCCTCAGGAAGGCCTGTACAAT<br>GAACTGCAGAAAGATAAGATGGCGGAGGCCTACAGTGAGATT<br>GGGATGAAAGGCGAGCGCCGGAGGGGCAAGGGGCACGATG<br>GCCTTTACCAGGGTCTCAGTACAGCCACCAAGGACACCTACG<br>ACGCCCTTCACATGCAGGCCCTGCCCCCTCGCGGAAGCGGA<br>GAGGGCAGGGGAAGTCTTCTAACATGCGGGGACGTGGAGGA<br>AAATCCCGGCCCCATGGTGAGCAAGGGCGAGGAGGATAACA<br>TGGCCATCATCAAGGAGTTCATGCGCTTCAAGGTGCACATGG<br>AGGGCTCCGTGAACGGCCACGAGTTCGAGATCGAGGGCGAG<br>GGCGAGGGCCGCCCCTACGAGGGCACCCAGACCGCCAAGC<br>TGAAGGTGACCAAGGGTGGCCCCCTGCCCTTCGCCTGGGAC<br>ATCCTGTCCCCTCAGTTCATGTACGGCTCCAAGGCCTACGTG<br>AAGCACCCCGCCGACATCCCCGACTACTTGAAGCTGTCCTTC<br>CCCGAGGGCTTCAAGTGGGAGCGCGTGATGAACTTCGAGGA<br>CGGCGGCGTGGTGACCGTGACCCAGGACTCCTCCCTGCAGG<br>ACGGCGAGTTCATCTACAAGGTGAAGCTGCGCGGCACCAAC<br>TTCCCCTCCGACGGCCCCGTAATGCAGAAGAAGACCATGGG<br>CTGGGAGGCCTCCTCCGAGCGGATGTACCCCGAGGACGGC<br>GCCCTGAAGGGCGAGATCAAGCAGAGGCTGAAGCTGAAGGA<br>CGGCGGCCACTACGACGCTGAGGTCAAGACCACCTACAAGG<br>CCAAGAAGCCCGTGCAGCTGCCCGGCGCCTACAACGTCAAC<br>ATCAAGTTGGACATCACCTCCCACAACGAGGACTACACCATC<br>GTGGAACAGTACGAACGCGCCGAGGGCCGCCACTCCACCG<br>GCGGCATGGACGAGCTGTACAAGGGAAGCGGAGCCACGAAC<br>TTCTCTCTGTTAAAGCAAGCAGGAGATGTTGAAGAAAACCCC<br>GGGCCTATGTACAGGATGCAACTCCTGTCTTGCATTGCACTA<br>AGTCTTGCACTTGTCACGAATTCGGAGGAGGAGCTGCAGATC<br>ATCCAGCCAGAGAAGCTGCTGCTGGTGACCGTGGGCAAGAC<br>GGCCACTCTTCATTGTACCATCACCTCTTTGTTCCCCGTGGG<br>TCCCATCCAGTGGTTCCGCGGGGTCGGACCCGGGCGGGTG<br>CTCATCTACAACCAGAAGGACGGCCACTTTCCTAGGGTCACC<br>ACAGTAAGCGACGGCACCAAGCGCAACAATATGGATTTCAGC<br>ATTCGCATCTCTTCCATTACTCCGGCGGACGTGGGCACCTAT

			TACTGCGTTAAATTTCGTAAGGGATCCCCGGAAGACGTGGAG TTCAAATCCGGCCCTGGTACGGAGATGGCTCTGGGCGCCAA GCCCTCGTAA
aEGFRvIII CAR	3C10.BBz	2247	ATGGCCTTACCAGTGACCGCCTTGCTCCTGCCGCTGGCCTTG CTGCTCCACGCCGCCAGGCCGGGATCCGAGATTCAGCTGCA GCAATCTGGGGCAGAACTTGTGAAGCCAGGGGCCTCAGTCA AGCTGTCCTGCACAGGTTCTGGCTTCAACATTGAAGACTACT ATATTCACTGGGTGAAGCAGAGGACTGAACAGGGCCTGGAAT GGATTGGAAGGATTGATCCTGAGAATGATGAAACTAAATATG GCCCAATATTCCAGGGCAGGGCCACTATAACAGCAGACACAT CCTCCAACACAGTCTACCTGCAACTCAGCAGCCTGACATCTG AGGACACTGCCGTCTATTACTGTGCCTTTCGCGGTGGAGTCT ACTGGGGGCCAGGAACCACTCTCACAGTCTCCTCAGGAGGT GGTGGTTCCGGTGGTGGTGGTTCCGGAGGTGGTGGTTCACA TATGGATGTTGTGATGACCCAGTCTCCACTCACTCTATCGGTT GCCATTGGACAATCAGCCTCCATCTCTTGCAAGTCAAGTCAG AGCCTCTTAGATAGTGATGGAAAGACATATTTGAATTGGTTGT TACAGAGGCCAGGCCAGTCTCCAAAGCGCCTAATCTCTCTGG TGTCTAAACTGGACTCTGGAGTCCCTGACAGGTTCACTGGCA GTGGATCAGGGACAGATTTCACACTGAGAATCAGCAGAGTGG AGGCTGAGGATTTGGGAATTTATTATTGCTGGCAAGGTACAC ATTTTCCTGGGACGTTCGGTGGAGGGACCAAGCTGGAGATAA AAGCTAGCACCACGACGCCAGCGCCGCGACCACCAACACCG GCGCCCACCATCGCGTCGCAGCCCCTGTCCCTGCGCCCAGA GGCGTGCCGGCCAGCGGCGGGGGGCGCAGTGCACACGAG GGGGCTGGACTTCGCCTGTGATATCTACATCTGGGCGCCCTT GGCCGGGACTTGTGGGGTCCTTCTCCTGTCACTGGTTATCAC CCTTTACTGCAAACGGGGCAGAAAGAAACTCCTGTATATATTC AAACAACCATTTATGAGACCAGTACAAACTACTCAAGAGGAA GATGGCTGTAGCTGCCGATTTCCAGAAGAAGAAGAAGGAGG ATGTGAACTGAGAGTGAAGTTCAGCAGGAGCGCAGACGCCC CCGCGTACAAGCAGGGCCAGAACCAGCTCTATAACGAGCTC AATCTAGGACGAAGAGAGGAGTACGATGTTTTGGACAAGAGA CGTGGCCGGGACCCTGAGATGGGGGGAAAGCCGAGAAGGA AGAACCCTCAGGAAGGCCTGTACAATGAACTGCAGAAAGATA AGATGGCGGAGGCCTACAGTGAGATTGGGATGAAAGGCGAG CGCCGGAGGGGCAAGGGGCACGATGGCCTTTACCAGGGTCT CAGTACAGCCACCAAGGACACCTACGACGCCCTTCACATGCA GGCCCTGCCCCCTCGCGGAAGCGGAGAGGGCAGGGGAAGT CTTCTAACATGCGGGGACGTGGAGGAAAATCCCGGCCCCAT GGTGAGCAAGGGCGAGGAGGATAACATGGCCATCATCAAGG AGTTCATGCGCTTCAAGGTGCACATGGAGGGCTCCGTGAAC GGCCACGAGTTCGAGATCGAGGGCGAGGGCGAGGGCCGCC CCTACGAGGGCACCCAGACCGCCAAGCTGAAGGTGACCAAG GGTGGCCCCCTGCCCTTCGCCTGGGACATCCTGTCCCCTCA GTTCATGTACGGCTCCAAGGCCTACGTGAAGCACCCCGCCG ACATCCCCGACTACTTGAAGCTGTCCTTCCCCGAGGGCTTCA AGTGGGAGCGCGTGATGAACTTCGAGGACGGCGGCGTGGT GACCGTGACCCAGGACTCCTCCCTGCAGGACGGCGAGTTCA TCTACAAGGTGAAGCTGCGCGGCACCAACTTCCCCTCCGAC GGCCCCGTAATGCAGAAGAAGACCATGGGCTGGGAGGCCTC CTCCGAGCGGATGTACCCCGAGGACGGCGCCCTGAAGGGC GAGATCAAGCAGAGGCTGAAGCTGAAGGACGGCGGCCACTA CGACGCTGAGGTCAAGACCACCTACAAGGCCAAGAAGCCCG TGCAGCTGCCCGGCGCCTACAACGTCAACATCAAGTTGGACA TCACCTCCCACAACGAGGACTACACCATCGTGGAACAGTACG AACGCGCCGAGGGCCGCCACTCCACCGGCGGCATGGACGA GCTGTACAAGTAA
aEGFRvIII-SGRP CAR	3C10.BBz	2730	ATGGCCTTACCAGTGACCGCCTTGCTCCTGCCGCTGGCCTTG CTGCTCCACGCCGCCAGGCCGGGATCCGAGATTCAGCTGCA GCAATCTGGGGCAGAACTTGTGAAGCCAGGGGCCTCAGTCA AGCTGTCCTGCACAGGTTCTGGCTTCAACATTGAAGACTACT

			ATATTCACTGGGTGAAGCAGAGGACTGAACAGGGCCTGGAAT<br>GGATTGGAAGGATTGATCCTGAGAATGATGAAACTAAATATG<br>GCCCAATATTCCAGGGCAGGGCCACTATAACAGCAGACACAT<br>CCTCCAACACAGTCTACCTGCAACTCAGCAGCCTGACATCTG<br>AGGACACTGCCGTCTATTACTGTGCCTTTCGCGGTGGAGTCT<br>ACTGGGGGCCAGGAACCACTCTCACAGTCTCCTCAGGAGGT<br>GGTGGTTCCGGTGGTGGTGGTTCCGGAGGTGGTGGTTCACA<br>TATGGATGTTGTGATGACCCAGTCTCCACTCACTCTATCGGTT<br>GCCATTGGACAATCAGCCTCCATCTCTTGCAAGTCAAGTCAG<br>AGCCTCTTAGATAGTGATGGAAAGACATATTTGAATTGGTTGT<br>TACAGAGGCCAGGCCAGTCTCCAAAGCGCCTAATCTCTCTGG<br>TGTCTAAACTGGACTCTGGAGTCCCTGACAGGTTCACTGGCA<br>GTGGATCAGGGACAGATTTCACACTGAGAATCAGCAGAGTGG<br>AGGCTGAGGATTTGGGAATTTATTATTGCTGGCAAGGTACAC<br>ATTTTCCTGGGACGTTCGGTGGAGGGACCAAGCTGGAGATAA<br>AAGCTAGCACCACGACGCCAGCGCCGCGACCACCAACACCG<br>GCGCCCACCATCGCGTCGCAGCCCCTGTCCCTGCGCCCAGA<br>GGCGTGCCGGCCAGCGGCGGGGGGCGCAGTGCACACGAG<br>GGGGCTGGACTTCGCCTGTGATATCTACATCTGGGCGCCCTT<br>GGCCGGGACTTGTGGGGTCCTTCTCCTGTCACTGGTTATCAC<br>CCTTTACTGCAAACGGGGCAGAAAGAAACTCCTGTATATATTC<br>AAACAACCATTTATGAGACCAGTACAAACTACTCAAGAGGAA<br>GATGGCTGTAGCTGCCGATTTCCAGAAGAAGAAGAAGGAGG<br>ATGTGAACTGAGAGTGAAGTTCAGCAGGAGCGCAGACGCCC<br>CCGCGTACAAGCAGGGCCAGAACCAGCTCTATAACGAGCTC<br>AATCTAGGACGAAGAGAGGAGTACGATGTTTTGGACAAGAGA<br>CGTGGCCGGGACCCTGAGATGGGGGGAAAGCCGAGAAGGA<br>AGAACCCTCAGGAAGGCCTGTACAATGAACTGCAGAAAGATA<br>AGATGGCGGAGGCCTACAGTGAGATTGGGATGAAAGGCGAG<br>CGCCGGAGGGGCAAGGGGCACGATGGCCTTTACCAGGGTCT<br>CAGTACAGCCACCAAGGACACCTACGACGCCCTTCACATGCA<br>GGCCCTGCCCCCTCGCGGAAGCGGAGAGGGCAGGGGAAGT<br>CTTCTAACATGCGGGGACGTGGAGGAAAATCCCGGCCCCAT<br>GGTGAGCAAGGGCGAGGAGGATAACATGGCCATCATCAAGG<br>AGTTCATGCGCTTCAAGGTGCACATGGAGGGCTCCGTGAAC<br>GGCCACGAGTTCGAGATCGAGGGCGAGGGCGAGGGCCGCC<br>CCTACGAGGGCACCCAGACCGCCAAGCTGAAGGTGACCAAG<br>GGTGGCCCCCTGCCCTTCGCCTGGGACATCCTGTCCCCTCA<br>GTTCATGTACGGCTCCAAGGCCTACGTGAAGCACCCCGCCG<br>ACATCCCCGACTACTTGAAGCTGTCCTTCCCCGAGGGCTTCA<br>AGTGGGAGCGCGTGATGAACTTCGAGGACGGCGGCGTGGT<br>GACCGTGACCCAGGACTCCTCCCTGCAGGACGGCGAGTTCA<br>TCTACAAGGTGAAGCTGCGCGGCACCAACTTCCCCTCCGAC<br>GGCCCCGTAATGCAGAAGAAGACCATGGGCTGGGAGGCCTC<br>CTCCGAGCGGATGTACCCCGAGGACGGCGCCCTGAAGGGC<br>GAGATCAAGCAGAGGCTGAAGCTGAAGGACGGCGGCCACTA<br>CGACGCTGAGGTCAAGACCACCTACAAGGCCAAGAAGCCCG<br>TGCAGCTGCCCGGCGCCTACAACGTCAACATCAAGTTGGACA<br>TCACCTCCCACAACGAGGACTACACCATCGTGGAACAGTACG<br>AACGCGCCGAGGGCCGCCACTCCACCGGCGGCATGGACGA<br>GCTGTACAAGGGAAGCGGAGCCACGAACTTCTCTCTGTTAAA<br>GCAAGCAGGAGATGTTGAAGAAAACCCCGGGCCTATGTACA<br>GGATGCAACTCCTGTCTTGCATTGCACTAAGTCTTGCACTTGT<br>CACGAATTCGGAGGAGGAGCTGCAGATCATCCAGCCAGAGA<br>AGCTGCTGCTGGTGACCGTGGGCAAGACGGCCACTCTTCAT<br>TGTACCATCACCTCTTTGTTCCCCGTGGGTCCCATCCAGTGG<br>TTCCGCGGGGTCGGACCCGGGCGGGTGCTCATCTACAACCA<br>GAAGGACGGCCACTTTCCTAGGGTCACCACAGTAAGCGACG<br>GCACCAAGCGCAACAATATGGATTTCAGCATTCGCATCTCTT<br>CCATTACTCCGGCGGACGTGGGCACCTATTACTGCGTTAAAT<br>TTCGTAAGGGATCCCCGGAAGACGTGGAGTTCAAATCCGGC<br>CCTGGTACGGAGATGGCTCTGGGCGCCAAGCCCTCGTAA

aEGFRvIII-SGRP-His CAR	3C10.BBz	2748	ATGGCCTTACCAGTGACCGCCTTGCTCCTGCCGCTGGCCTTG<br>CTGCTCCACGCCGCCAGGCCGGGATCCGAGATTCAGCTGCA<br>GCAATCTGGGGCAGAACTTGTGAAGCCAGGGGCCTCAGTCA<br>AGCTGTCCTGCACAGGTTCTGGCTTCAACATTGAAGACTACT<br>ATATTCACTGGGTGAAGCAGAGGACTGAACAGGGCCTGGAAT<br>GGATTGGAAGGATTGATCCTGAGAATGATGAAACTAAATATG<br>GCCCAATATTCCAGGGCAGGGCCACTATAACAGCAGACACAT<br>CCTCCAACACAGTCTACCTGCAACTCAGCAGCCTGACATCTG<br>AGGACACTGCCGTCTATTACTGTGCCTTTCGCGGTGGAGTCT<br>ACTGGGGGCCAGGAACCACTCTCACAGTCTCCTCAGGAGGT<br>GGTGGTTCCGGTGGTGGTGGTTCCGGAGGTGGTGGTTCACA<br>TATGGATGTTGTGATGACCCAGTCTCCACTCACTCTATCGGTT<br>GCCATTGGACAATCAGCCTCCATCTCTTGCAAGTCAAGTCAG<br>AGCCTCTTAGATAGTGATGGAAAGACATATTTGAATTGGTTGT<br>TACAGAGGCCAGGCCAGTCTCCAAAGCGCCTAATCTCTCTGG<br>TGTCTAAACTGGACTCTGGAGTCCCTGACAGGTTCACTGGCA<br>GTGGATCAGGGACAGATTTCACACTGAGAATCAGCAGAGTGG<br>AGGCTGAGGATTTGGGAATTTATTATTGCTGGCAAGGTACAC<br>ATTTTCCTGGGACGTTCGGTGGAGGGACCAAGCTGGAGATAA<br>AAGCTAGCACCACGACGCCAGCGCCGCGACCACCAACACCG<br>GCGCCCACCATCGCGTCGCAGCCCCTGTCCCTGCGCCCAGA<br>GGCGTGCCGGCCAGCGGCGGGGGGCGCAGTGCACACGAG<br>GGGGCTGGACTTCGCCTGTGATATCTACATCTGGGCGCCCTT<br>GGCCGGGACTTGTGGGGTCCTTCTCCTGTCACTGGTTATCAC<br>CCTTTACTGCAAACGGGGCAGAAAGAAACTCCTGTATATATTC<br>AAACAACCATTTATGAGACCAGTACAAACTACTCAAGAGGAA<br>GATGGCTGTAGCTGCCGATTTCCAGAAGAAGAAGAAGGAGG<br>ATGTGAACTGAGAGTGAAGTTCAGCAGGAGCGCAGACGCCC<br>CCGCGTACAAGCAGGGCCAGAACCAGCTCTATAACGAGCTC<br>AATCTAGGACGAAGAGAGGAGTACGATGTTTTGGACAAGAGA<br>CGTGGCCGGGACCCTGAGATGGGGGGAAAGCCGAGAAGGA<br>AGAACCCTCAGGAAGGCCTGTACAATGAACTGCAGAAAGATA<br>AGATGGCGGAGGCCTACAGTGAGATTGGGATGAAAGGCGAG<br>CGCCGGAGGGGCAAGGGGCACGATGGCCTTTACCAGGGTCT<br>CAGTACAGCCACCAAGGACACCTACGACGCCCTTCACATGCA<br>GGCCCTGCCCCCTCGCGGAAGCGGAGAGGGCAGGGGAAGT<br>CTTCTAACATGCGGGGACGTGGAGGAAAATCCCGGCCCCAT<br>GGTGAGCAAGGGCGAGGAGGATAACATGGCCATCATCAAGG<br>AGTTCATGCGCTTCAAGGTGCACATGGAGGGCTCCGTGAAC<br>GGCCACGAGTTCGAGATCGAGGGCGAGGGCGAGGGCCGCC<br>CCTACGAGGGCACCCAGACCGCCAAGCTGAAGGTGACCAAG<br>GGTGGCCCCCTGCCCTTCGCCTGGGACATCCTGTCCCCTCA<br>GTTCATGTACGGCTCCAAGGCCTACGTGAAGCACCCCGCCG<br>ACATCCCCGACTACTTGAAGCTGTCCTTCCCCGAGGGCTTCA<br>AGTGGGAGCGCGTGATGAACTTCGAGGACGGCGGCGTGGT<br>GACCGTGACCCAGGACTCCTCCCTGCAGGACGGCGAGTTCA<br>TCTACAAGGTGAAGCTGCGCGGCACCAACTTCCCCTCCGAC<br>GGCCCCGTAATGCAGAAGAAGACCATGGGCTGGGAGGCCTC<br>CTCCGAGCGGATGTACCCCGAGGACGGCGCCCTGAAGGGC<br>GAGATCAAGCAGAGGCTGAAGCTGAAGGACGGCGGCCACTA<br>CGACGCTGAGGTCAAGACCACCTACAAGGCCAAGAAGCCCG<br>TGCAGCTGCCCGGCGCCTACAACGTCAACATCAAGTTGGACA<br>TCACCTCCCACAACGAGGACTACACCATCGTGGAACAGTACG<br>AACGCGCCGAGGGCCGCCACTCCACCGGCGGCATGGACGA<br>GCTGTACAAGGGAAGCGGAGCCACGAACTTCTCTCTGTTAAA<br>GCAAGCAGGAGATGTTGAAGAAAACCCCGGGCCTATGTACA<br>GGATGCAACTCCTGTCTTGCATTGCACTAAGTCTTGCACTTGT<br>CACGAATTCGCATCACCATCACCATCACGAGGAGGAGCTGCA<br>GATCATCCAGCCAGAGAAGCTGCTGCTGGTGACCGTGGGCA<br>AGACGGCCACTCTTCATTGTACCATCACCTCTTTGTTCCCCGT<br>GGGTCCCATCCAGTGGTTCCGCGGGGTCGGACCCGGGCGG<br>GTGCTCATCTACAACCAGAAGGACGGCCACTTTCCTAGGGTC

			ACCACAGTAAGCGACGGCACCAAGCGCAACAATATGGATTTC AGCATTCGCATCTCTTCCATTACTCCGGCGGACGTGGGCACC TATTACTGCGTTAAATTTCGTAAGGGATCCCCGGAAGACGTG GAGTTCAAATCCGGCCCTGGTACGGAGATGGCTCTGGGCGC CAAGCCCTCGTAA

**FMC63.BBz CAR construct domain**	**bp**	**nt sequence**
FMC63.BBz	1488	ATGGCCTTACCAGTGACCGCCTTGCTCCTGCCGCTGGCCTTGCTGCTCCACG CCGCCAGGCCGGGATCCGACATCCAGATGACACAGACTACATCCTCCCTGT CTGCCTCTCTGGGAGACAGAGTCACCATCAGTTGCAGGGCAAGTCAGGACAT TAGTAAATATTTAAATTGGTATCAGCAGAAACCAGATGGAACTGTTAAACTCCT GATCTACCATACATCAAGATTACACTCAGGAGTCCCATCAAGGTTCAGTGGCA GTGGGTCTGGAACAGATTATTCTCTCACCATTAGCAACCTGGAGCAAGAAGA TATTGCCACTTACTTTTGCCAACAGGGTAATACGCTTCCGTACACGTTCGGAG GGGGGACTAAGTTGGAAATAACAGGCTCCACCTCTGGATCCGGCAAGCCCG GATCTGGCGAGGGATCCACCAAGGGCGAGGTGAAACTGCAGGAGTCAGGAC CTGGCCTGGTGGCGCCCTCACAGAGCCTGTCCGTCACATGCACTGTCTCAG GGGTCTCATTACCCGACTATGGTGTAAGCTGGATTCGCCAGCCTCCACGAAA GGGTCTGGAGTGGCTGGGAGTAATATGGGGTAGTGAAACCACATACTATAAT TCAGCTCTCAAATCCAGACTGACCATCATCAAGGACAACTCCAAGAGCCAAG TTTTCTTAAAAATGAACAGTCTGCAAACTGATGACACAGCCATTTACTACTGTG CCAAACATTATTACTACGGTGGTAGCTATGCTATGGACTACTGGGGTCAAGG AACCTCAGTCACCGTCTCCTCAGCGGCCGCAGCTAGCACCACGACGCCAGC GCCGCGACCACCAACACCGGCGCCCACCATCGCGTCGCAGCCCCTGTCCCT GCGCCCAGAGGCGTGCCGGCCAGCGGCGGGGGGCGCAGTGCACACGAGG GGGCTGGACTTCGCCTGTGATATCTACATCTGGGCGCCCTTGGCCGGGACT TGTGGGGTCCTTCTCCTGTCACTGGTTATCACCCTTTACTGCAAACGGGGCA GAAAGAAACTCCTGTATATATTCAAACAACCATTTATGAGACCAGTACAAACTA CTCAAGAGGAAGATGGCTGTAGCTGCCGATTTCCAGAAGAAGAAGAAGGAG GATGTGAACTGAGAGTGAAGTTCAGCAGGAGCGCAGACGCCCCCGCGTACA AGCAGGGCCAGAACCAGCTCTATAACGAGCTCAATCTAGGACGAAGAGAGG AGTACGATGTTTTGGACAAGAGACGTGGCCGGGACCCTGAGATGGGGGGAA AGCCGAGAAGGAAGAACCCTCAGGAAGGCCTGTACAATGAACTGCAGAAAG ATAAGATGGCGGAGGCCTACAGTGAGATTGGGATGAAAGGCGAGCGCCGGA GGGGCAAGGGGCACGATGGCCTTTACCAGGGTCTCAGTACAGCCACCAAGG ACACCTACGACGCCCTTCACATGCAGGCCCTGCCCCCTCGC
CD8a leader	60	GCCTTACCAGTGACCGCCTTGCTCCTGCCGCTGGCCTTGCTGCTCCACGCC GCCAGGCCG
FMC63	744	GACATCCAGATGACACAGACTACATCCTCCCTGTCTGCCTCTCTGGGAGACA GAGTCACCATCAGTTGCAGGGCAAGTCAGGACATTAGTAAATATTTAAATTGG TATCAGCAGAAACCAGATGGAACTGTTAAACTCCTGATCTACCATACATCAAG ATTACACTCAGGAGTCCCATCAAGGTTCAGTGGCAGTGGGTCTGGAACAGAT TATTCTCTCACCATTAGCAACCTGGAGCAAGAAGATATTGCCACTTACTTTTG CCAACAGGGTAATACGCTTCCGTACACGTTCGGAGGGGGGACTAAGTTGGA AATAACAGGCTCCACCTCTGGATCCGGCAAGCCCGGATCTGGCGAGGGATC CACCAAGGGCGAGGTGAAACTGCAGGAGTCAGGACCTGGCCTGGTGGCGC CCTCACAGAGCCTGTCCGTCACATGCACTGTCTCAGGGGTCTCATTACCCGA CTATGGTGTAAGCTGGATTCGCCAGCCTCCACGAAAGGGTCTGGAGTGGCT GGGAGTAATATGGGGTAGTGAAACCACATACTATAATTCAGCTCTCAAATCCA GACTGACCATCATCAAGGACAACTCCAAGAGCCAAGTTTTCTTAAAAATGAAC AGTCTGCAAACTGATGACACAGCCATTTACTACTGTGCCAAACATTATTACTA CGGTGGTAGCTATGCTATGGACTACTGGGGTCAAGGAACCTCAGTCACCGTC TCCTCAGCGGCCGCA
CD8a hinge	135	ACCACGACGCCAGCGCCGCGACCACCAACACCGGCGCCCACCATCGCGTC GCAGCCCCTGTCCCTGCGCCCAGAGGCGTGCCGGCCAGCGGCGGGGGGC GCAGTGCACACGAGGGGGCTGGACTTCGCCTGTGAT
CD8a transmembrane	72	ATCTACATCTGGGCGCCCTTGGCCGGGACTTGTGGGGTCCTTCTCCTGTCAC TGGTTATCACCCTTTACTGC

4-1BB	126	AAACGGGGCAGAAAGAAACTCCTGTATATATTCAAACAACCATTTATGAGACC AGTACAAACTACTCAAGAGGAAGATGGCTGTAGCTGCCGATTTCCAGAAGAA GAAGAAGGAGGATGTGAACTG
CD3z	336	AGAGTGAAGTTCAGCAGGAGCGCAGACGCCCCCGCGTACCAGCAGGGCCA GAACCAGCTCTATAACGAGCTCAATCTAGGACGAAGAGAGGAGTACGATGTT TTGGACAAGAGACGTGGCCGGGACCCTGAGATGGGGGGAAAGCCGAGAAG GAAGAACCCTCAGGAAGGCCTGTACAATGAACTGCAGAAAGATAAGATGGCG GAGGCCTACAGTGAGATTGGGATGAAAGGCGAGCGCCGGAGGGGCAAGGG GCACGATGGCCTTTACCAGGGTCTCAGTACAGCCACCAAGGACACCTACGAC GCCCTTCACATGCAGGCCCTGCCCCCTCGC
T2A	63	GGAAGCGGAGAGGGCAGGGGAAGTCTTCTAACATGCGGGGACGTGGAGGA AAATCCCGGCCCC
mCherry	708	ATGGTGAGCAAGGGCGAGGAGGATAACATGGCCATCATCAAGGAGTTCATG CGCTTCAAGGTGCACATGGAGGGCTCCGTGAACGGCCACGAGTTCGAGATC GAGGGCGAGGGCGAGGGCCGCCCCTACGAGGGCACCCAGACCGCCAAGCT GAAGGTGACCAAGGGTGGCCCCCTGCCCTTCGCCTGGGACATCCTGTCCCC TCAGTTCATGTACGGCTCCAAGGCCTACGTGAAGCACCCCGCCGACATCCCC GACTACTTGAAGCTGTCCTTCCCCGAGGGCTTCAAGTGGGAGCGCGTGATG AACTTCGAGGACGGCGGCGTGGTGACCGTGACCCAGGACTCCTCCCTGCAG GACGGCGAGTTCATCTACAAGGTGAAGCTGCGCGGCACCAACTTCCCCTCC GACGGCCCCGTAATGCAGAAGAAGACCATGGGCTGGGAGGCCTCCTCCGAG CGGATGTACCCCGAGGACGGCGCCCTGAAGGGCGAGATCAAGCAGAGGCT GAAGCTGAAGGACGGCGGCCACTACGACGCTGAGGTCAAGACCACCTACAA GGCCAAGAAGCCCGTGCAGCTGCCCGGCGCCTACAACGTCAACATCAAGTT GGACATCACCTCCCACAACGAGGACTACACCATCGTGGAACAGTACGAACGC GCCGAGGGCCGCCACTCCACCGGCGGCATGGACGAGCTGTACAAG
P2A	66	GGAAGCGGAGCCACGAACTTCTCTCTGTTAAAGCAAGCAGGAGATGTTGAAG AAAACCCCGGGCCT
IL2sig-SGRP	417	ATGTACAGGATGCAACTCCTGTCTTGCATTGCACTAAGTCTTGCACTTGTCAC GAATTCGGAGGAGGAGCTGCAGATCATCCAGCCAGAGAAGCTGCTGCTGGT GACCGTGGGCAAGACGGCCACTCTTCATTGTACCATCACCTCTTTGTTCCCC GTGGGTCCCATCCAGTGGTTCCGCGGGGTCGGACCCGGGCGGGTGCTCAT CTACAACCAGAAGGACGGCCACTTTCCTAGGGTCACCACAGTAAGCGACGG CACCAAGCGCAACAATATGGATTTCAGCATTCGCATCTCTTCCATTACTCCGG CGGACGTGGGCACCTATTACTGCGTTAAATTTCGTAAGGGATCCCCGGAAGA CGTGGAGTTCAAATCCGGCCCTGGTACGGAGATGGCTCTGGGCGCCAAGCC CTCG
IL2sig	57	TACAGGATGCAACTCCTGTCTTGCATTGCACTAAGTCTTGCACTTGTCACGAA TTCG
SGRP	357	GAGGAGGAGCTGCAGATCATCCAGCCAGAGAAGCTGCTGCTGGTGACCGTG GGCAAGACGGCCACTCTTCATTGTACCATCACCTCTTTGTTCCCCGTGGGTC CCATCCAGTGGTTCCGCGGGGTCGGACCCGGGCGGGTGCTCATCTACAACC AGAAGGACGGCCACTTTCCTAGGGTCACCACAGTAAGCGACGGCACCAAGC GCAACAATATGGATTTCAGCATTCGCATCTCTTCCATTACTCCGGCGGACGTG GGCACCTATTACTGCGTTAAATTTCGTAAGGGATCCCCGGAAGACGTGGAGT TCAAATCCGGCCCTGGTACGGAGATGGCTCTGGGCGCCAAGCCCTCG

**3C10.BBz CAR construct domain**	**bp**	**nt sequence**
3C10.BBz	1473	ATGGCCTTACCAGTGACCGCCTTGCTCCTGCCGCTGGCCTTGCTGCTCCACG CCGCCAGGCCGGGATCCGAGATTCAGCTGCAGCAATCTGGGGCAGAACTTG TGAAGCCAGGGGCCTCAGTCAAGCTGTCCTGCACAGGTTCTGGCTTCAACAT TGAAGACTACTATATTCACTGGGTGAAGCAGAGGACTGAACAGGGCCTGGAA TGGATTGGAAGGATTGATCCTGAGAATGATGAAACTAAATATGGCCCAATATT CCAGGGCAGGGCCACTATAACAGCAGACACATCCTCCAACACAGTCTACCTG CAACTCAGCAGCCTGACATCTGAGGACACTGCCGTCTATTACTGTGCCTTTC GCGGTGGAGTCTACTGGGGGCCAGGAACCACTCTCACAGTCTCCTCAGGAG GTGGTGGTTCCGGTGGTGGTGGTTCCGGAGGTGGTGGTTCACATATGGATG TTGTGATGACCCAGTCTCCACTCACTCTATCGGTTGCCATTGGACAATCAGCC TCCATCTCTTGCAAGTCAAGTCAGAGCCTCTTAGATAGTGATGGAAAGACATA

		TTTGAATTGGTTGTTACAGAGGCCAGGCCAGTCTCCAAAGCGCCTAATCTCT CTGGTGTCTAAACTGGACTCTGGAGTCCCTGACAGGTTCACTGGCAGTGGAT CAGGGACAGATTTCACACTGAGAATCAGCAGAGTGGAGGCTGAGGATTTGG GAATTTATTATTGCTGGCAAGGTACACATTTTCCTGGGACGTTCGGTGGAGG GACCAAGCTGGAGATAAAAGCTAGCACCACGACGCCAGCGCCGCGACCACC AACACCGGCGCCCACCATCGCGTCGCAGCCCCTGTCCCTGCGCCCAGAGGC GTGCCGGCCAGCGGCGGGGGGCGCAGTGCACACGAGGGGGCTGGACTTC GCCTGTGATATCTACATCTGGGCGCCCTTGGCCGGGACTTGTGGGGTCCTTC TCCTGTCACTGGTTATCACCCTTTACTGCAAACGGGGCAGAAAGAAACTCCT GTATATATTCAAACAACCATTTATGAGACCAGTACAAACTACTCAAGAGGAAG ATGGCTGTAGCTGCCGATTTCCAGAAGAAGAAGAAGGAGGATGTGAACTGAG AGTGAAGTTCAGCAGGAGCGCAGACGCCCCCGCGTACAAGCAGGGCCAGAA CCAGCTCTATAACGAGCTCAATCTAGGACGAAGAGAGGAGTACGATGTTTTG GACAAGAGACGTGGCCGGGACCCTGAGATGGGGGGAAAGCCGAGAAGGAA GAACCCTCAGGAAGGCCTGTACAATGAACTGCAGAAAGATAAGATGGCGGA GGCCTACAGTGAGATTGGGATGAAAGGCGAGCGCCGGAGGGGCAAGGGGC ACGATGGCCTTTACCAGGGTCTCAGTACAGCCACCAAGGACACCTACGACGC CCTTCACATGCAGGCCCTGCCCCCTCGC
CD8a leader	60	GCCTTACCAGTGACCGCCTTGCTCCTGCCGCTGGCCTTGCTGCTCCACGCC GCCAGGCCG
3C10	729	GAGATTCAGCTGCAGCAATCTGGGGCAGAACTTGTGAAGCCAGGGGCCTCA GTCAAGCTGTCCTGCACAGGTTCTGGCTTCAACATTGAAGACTACTATATTCA CTGGGTGAAGCAGAGGACTGAACAGGGCCTGGAATGGATTGGAAGGATTGA TCCTGAGAATGATGAAACTAAATATGGCCCAATATTCCAGGGCAGGGCCACT ATAACAGCAGACACATCCTCCAACACAGTCTACCTGCAACTCAGCAGCCTGA CATCTGAGGACACTGCCGTCTATTACTGTGCCTTTCGCGGTGGAGTCTACTG GGGGCCAGGAACCACTCTCACAGTCTCCTCAGGAGGTGGTGGTTCCGGTGG TGGTGGTTCCGGAGGTGGTGGTTCACATATGGATGTTGTGATGACCCAGTCT CCACTCACTCTATCGGTTGCCATTGGACAATCAGCCTCCATCTCTTGCAAGTC AAGTCAGAGCCTCTTAGATAGTGATGGAAAGACATATTTGAATTGGTTGTTAC AGAGGCCAGGCCAGTCTCCAAAGCGCCTAATCTCTCTGGTGTCTAAACTGGA CTCTGGAGTCCCTGACAGGTTCACTGGCAGTGGATCAGGGACAGATTTCACA CTGAGAATCAGCAGAGTGGAGGCTGAGGATTTGGGAATTTATTATTGCTGGC AAGGTACACATTTTCCTGGGACGTTCGGTGGAGGGACCAAGCTGGAGATAAA A
CD8a hinge	135	ACCACGACGCCAGCGCCGCGACCACCAACACCGGCGCCCACCATCGCGTC GCAGCCCCTGTCCCTGCGCCCAGAGGCGTGCCGGCCAGCGGCGGGGGGC GCAGTGCACACGAGGGGGCTGGACTTCGCCTGTGAT
CD8a transmembrane	72	ATCTACATCTGGGCGCCCTTGGCCGGGACTTGTGGGGTCCTTCTCCTGTCAC TGGTTATCACCCTTTACTGC
4-1BB	126	AAACGGGGCAGAAAGAAACTCCTGTATATATTCAAACAACCATTTATGAGACC AGTACAAACTACTCAAGAGGAAGATGGCTGTAGCTGCCGATTTCCAGAAGAA GAAGAAGGAGGATGTGAACTG
CD3z	336	AGAGTGAAGTTCAGCAGGAGCGCAGACGCCCCCGCGTACCAGCAGGGCCA GAACCAGCTCTATAACGAGCTCAATCTAGGACGAAGAGAGGAGTACGATGTT TTGGACAAGAGACGTGGCCGGGACCCTGAGATGGGGGGAAAGCCGAGAAG GAAGAACCCTCAGGAAGGCCTGTACAATGAACTGCAGAAAGATAAGATGGCG GAGGCCTACAGTGAGATTGGGATGAAAGGCGAGCGCCGGAGGGGCAAGGG GCACGATGGCCTTTACCAGGGTCTCAGTACAGCCACCAAGGACACCTACGAC GCCCTTCACATGCAGGCCCTGCCCCCTCGC
T2A	63	GGAAGCGGAGAGGGCAGGGGAAGTCTTCTAACATGCGGGGACGTGGAGGA AAATCCCGGCCCC
mCherry	708	ATGGTGAGCAAGGGCGAGGAGGATAACATGGCCATCATCAAGGAGTTCATG CGCTTCAAGGTGCACATGGAGGGCTCCGTGAACGGCCACGAGTTCGAGATC GAGGGCGAGGGCGAGGGCCGCCCCTACGAGGGCACCCAGACCGCCAAGCT GAAGGTGACCAAGGGTGGCCCCCTGCCCTTCGCCTGGGACATCCTGTCCCC TCAGTTCATGTACGGCTCCAAGGCCTACGTGAAGCACCCCGCCGACATCCCC GACTACTTGAAGCTGTCCTTCCCCGAGGGCTTCAAGTGGGAGCGCGTGATG AACTTCGAGGACGGCGGCGTGGTGACCGTGACCCAGGACTCCTCCCTGCAG GACGGCGAGTTCATCTACAAGGTGAAGCTGCGCGGCACCAACTTCCCCTCC GACGGCCCCGTAATGCAGAAGAAGACCATGGGCTGGGAGGCCTCCTCCGAG CGGATGTACCCCGAGGACGGCGCCCTGAAGGGCGAGATCAAGCAGAGGCT

		GAAGCTGAAGGACGGCGGCCACTACGACGCTGAGGTCAAGACCACCTACAA GGCCAAGAAGCCCGTGCAGCTGCCCGGCGCCTACAACGTCAACATCAAGTT GGACATCACCTCCCACAACGAGGACTACACCATCGTGGAACAGTACGAACGC GCCGAGGGCCGCCACTCCACCGGCGGCATGGACGAGCTGTACAAG
P2A	66	GGAAGCGGAGCCACGAACTTCTCTCTGTTAAAGCAAGCAGGAGATGTTGAAG AAAACCCCGGGCCT
IL2sig-His-SGRP	435	ATGTACAGGATGCAACTCCTGTCTTGCATTGCACTAAGTCTTGCACTTGTCAC GAATTCGCATCACCATCACCATCACGAGGAGGAGCTGCAGATCATCCAGCCA GAGAAGCTGCTGCTGGTGACCGTGGGCAAGACGGCCACTCTTCATTGTACC ATCACCTCTTTGTTCCCCGTGGGTCCCATCCAGTGGTTCCGCGGGGTCGGAC CCGGGCGGGTGCTCATCTACAACCAGAAGGACGGCCACTTTCCTAGGGTCA CCACAGTAAGCGACGGCACCAAGCGCAACAATATGGATTTCAGCATTCGCAT CTCTTCCATTACTCCGGCGGACGTGGGCACCTATTACTGCGTTAAATTTCGTA AGGGATCCCCGGAAGACGTGGAGTTCAAATCCGGCCCTGGTACGGAGATGG CTCTGGGCGCCAAGCCCTCG
IL2sig-SGRP	417	ATGTACAGGATGCAACTCCTGTCTTGCATTGCACTAAGTCTTGCACTTGTCAC GAATTCGGAGGAGGAGCTGCAGATCATCCAGCCAGAGAAGCTGCTGCTGGT GACCGTGGGCAAGACGGCCACTCTTCATTGTACCATCACCTCTTTGTTCCCC GTGGGTCCCATCCAGTGGTTCCGCGGGGTCGGACCCGGGCGGGTGCTCAT CTACAACCAGAAGGACGGCCACTTTCCTAGGGTCACCACAGTAAGCGACGG CACCAAGCGCAACAATATGGATTTCAGCATTCGCATCTCTTCCATTACTCCGG CGGACGTGGGCACCTATTACTGCGTTAAATTTCGTAAGGGATCCCCGGAAGA CGTGGAGTTCAAATCCGGCCCTGGTACGGAGATGGCTCTGGGCGCCAAGCC CTCG
IL2sig	57	TACAGGATGCAACTCCTGTCTTGCATTGCACTAAGTCTTGCACTTGTCACGAA TTCG
His	18	CATCACCATCACCATCAC
SGRP	357	GAGGAGGAGCTGCAGATCATCCAGCCAGAGAAGCTGCTGCTGGTGACCGTG GGCAAGACGGCCACTCTTCATTGTACCATCACCTCTTTGTTCCCCGTGGGTC CCATCCAGTGGTTCCGCGGGGTCGGACCCGGGCGGGTGCTCATCTACAACC AGAAGGACGGCCACTTTCCTAGGGTCACCACAGTAAGCGACGGCACCAAGC GCAACAATATGGATTTCAGCATTCGCATCTCTTCCATTACTCCGGCGGACGTG GGCACCTATTACTGCGTTAAATTTCGTAAGGGATCCCCGGAAGACGTGGAGT TCAAATCCGGCCCTGGTACGGAGATGGCTCTGGGCGCCAAGCCCTCG

Supplementary Table 3. Nucleotide (nt) sequences of double fluorescence/bioluminescence lentiviral reporter and gene overexpression constructs used to transduce GBM cell lines.

GBM cell line transgene constructs	Transgene	Selection cassette	nt sequence
EGFRvIII	EGFRvIII	Puromycin	ATGCGACCCTCCGGGACGGCCGGGGCAGCGCTCCTGGC<br>GCTGCTGGCTGCGCTCTGCCCGGCGAGTCGGGCTCTGG<br>AGGAAAAGAAAGGTAATTATGTGGTGACAGATCACGGCTC<br>GTGCGTCCGAGCCTGTGGGGCCGACAGCTATGAGATGGA<br>GGAAGACGGCGTCCGCAAGTGTAAGAAGTGCGAAGGGC<br>CTTGCCGCAAAGTGTGTAACGGAATAGGTATTGGTGAATT<br>TAAAGACTCACTCTCCATAAATGCTACGAATATTAAACACT<br>TCAAAAACTGCACCTCCATCAGTGGCGATCTCCACATCCT<br>GCCGGTGGCATTTAGGGGTGACTCCTTCACACATACTCCT<br>CCTCTGGATCCACAGGAACTGGATATTCTGAAAACCGTAA<br>AGGAAATCACAGGGTTTTTGCTGATTCAGGCTTGGCCTGA<br>AAACAGGACGGACCTCCATGCCTTTGAGAACCTAGAAATC<br>ATACGCGGCAGGACCAAGCAACATGGTCAGTTTTCTCTTG<br>CAGTCGTCAGCCTGAACATAACATCCTTGGGATTACGCTC<br>CCTCAAGGAGATAAGTGATGGAGATGTGATAATTTCAGGA<br>AACAAAAATTTGTGCTATGCAAATACAATAAACTGGAAAAA<br>ACTGTTTGGGACCTCCGGTCAGAAAACCAAAATTATAAGC<br>AACAGAGGTGAAAACAGCTGCAAGGCCACAGGCCAGGTC<br>TGCCATGCCTTGTGCTCCCCCGAGGGCTGCTGGGGCCC<br>GGAGCCCAGGGACTGCGTCTCTTGCCGGAATGTCAGCCG<br>AGGCAGGGAATGCGTGGACAAGTGCAACCTTCTGGAGGG<br>TGAGCCAAGGGAGTTTGTGGAGAACTCTGAGTGCATACA<br>GTGCCACCCAGAGTGCCTGCCTCAGGCCATGAACATCAC<br>CTGCACAGGACGGGGACCAGACAACTGTATCCAGTGTGC<br>CCACTACATTGACGGCCCCCACTGCGTCAAGACCTGCCC<br>GGCAGGAGTCATGGGAGAAAACAACACCCTGGTCTGGAA<br>GTACGCAGACGCCGGCCATGTGTGCCACCTGTGCCATCC<br>AAACTGCACCTACGGATGCACTGGGCCAGGTCTTGAAGG<br>CTGTCCAACGAATGGGCCTAAGATCCCGTCCATCGCCAC<br>TGGGATGGTGGGGGCCCTCCTCTTGCTGCTGGTGGTGGC<br>CCTGGGGATCGGCCTCTTCATGCGAAGGCGCCACATCGT<br>TCGGAAGCGC
iRFP713-P2A-Nluc	iRFP713, NanoLuciferase	Puromycin	ATGGCTGAAGGATCCGTCGCCAGGCAGCCTGACCTCTTG<br>ACCTGCGACGATGAGCCGATCCATATCCCCGGTGCCATC<br>CAACCGCATGGACTGCTGCTCGCCCTCGCCGCCGACATG<br>ACGATCGTTGCCGGCAGCGACAACCTTCCCGAACTCACC<br>GGACTGGCGATCGGCGCCCTGATCGGCCGCTCTGCGGC<br>CGATGTCTTCGACTCGGAGACGCACAACCGTCTGACGAT<br>CGCCTTGGCCGAGCCCGGGGCGGCCGTCGGAGCACCGA<br>TCACTGTCGGCTTCACGATGCGAAAGGACGCAGGCTTCA<br>TCGGCTCCTGGCATCGCCATGATCAGCTCATCTTCCTCGA<br>GCTCGAGCCTCCCCAGCGGGACGTCGCCGAGCCGCAGG<br>CGTTCTTCCGCCGCACCAACAGCGCCATCCGCCGCCTGC<br>AGGCCGCCGAAACCTTGGAAAGCGCCTGCGCCGCCGCG<br>GCGCAAGAGGTGCGGAAGATTACCGGCTTCGATCGGGTG<br>ATGATCTATCGCTTCGCCTCCGACTTCAGCGGCGAAGTGA<br>TCGCAGAGGATCGGTGCGCCGAGGTCGAGTCAAAACTAG<br>GCCTGCACTATCCTGCCTCAACCGTGCCGGCGCAGGCCC<br>GTCGGCTCTATACCATCAACCCGGTACGGATCATTCCCGA<br>TATCAATTATCGGCCGGTGCCGGTCACCCCAGACCTCAAT<br>CCGGTCACCGGGCGGCCGATTGATCTTAGCTTCGCCATC<br>CTGCGCAGCGTCTCGCCCGTCCATCTGGAATTCATGCGC<br>AACATAGGCATGCACGGCACGATGTCGATCTCGATTTTGC<br>GCGGCGAGCGACTGTGGGGATTGATCGTTTGCCATCACC

			GAACGCCGTACTACGTCGATCTCGATGGCCGCCAAGCCT<br>GCGAGCTAGTCGCCCAGGTTCTGGCCTGGCAGATCGGC<br>GTGATGGAAGAGGGAAGCGGAGCCACGAACTTCTCTCTG<br>TTAAAGCAAGCAGGAGATGTTGAAGAAAACCCCGGGCCT<br>ATGGTCTTCACACTCGAAGATTTCGTTGGGGACTGGCGAC<br>AGACAGCCGGCTACAACCTGGACCAAGTCCTTGAACAGG<br>GAGGTGTGTCCAGTTTGTTTCAGAATCTCGGGGTGTCCGT<br>AACTCCGATCCAAAGGATTGTCCTGAGCGGTGAAAATGG<br>GCTGAAGATCGACATCCATGTCATCATCCCGTATGAAGGT<br>CTGAGCGGCGACCAAATGGGCCAGATCGAAAAAATTTTTA<br>AGGTGGTGTACCCTGTGGATGATCATCACTTTAAGGTGAT<br>CCTGCACTATGGCACACTGGTAATCGACGGGGTTACGCC<br>GAACATGATCGACTATTTCGGACGGCCGTATGAAGGCATC<br>GCCGTGTTCGACGGCAAAAAGATCACTGTAACAGGGACC<br>CTGTGGAACGGCAACAAAATTATCGACGAGCGCCTGATC<br>AACCCCGACGGCTCCCTGCTGTTCCGAGTAACCATCAAC<br>GGAGTGACCGGCTGGCGGCTGTGCGAACGCATTCTGGC<br>G
Luc2-T2A-mTagBFP2	mTagBFP2, Luciferase2	Puromycin	ATGGAAGATGCCAAAAACATTAAGAAGGGCCCAGCGCCA<br>TTCTACCCACTCGAAGACGGGACCGCCGGCGAGCAGCTG<br>CACAAAGCCATGAAGCGCTACGCCCTGGTGCCCGGCACC<br>ATCGCCTTTACCGACGCACATATCGAGGTGGACATTACCT<br>ACGCCGAGTACTTCGAGATGAGCGTTCGGCTGGCAGAAG<br>CTATGAAGCGCTATGGGCTGAATACAAACCATCGGATCGT<br>GGTGTGCAGCGAGAATAGCTTGCAGTTCTTCATGCCCGT<br>GTTGGGTGCCCTGTTCATCGGTGTGGCTGTGGCCCCAGC<br>TAACGACATCTACAACGAGCGCGAGCTGCTGAACAGCAT<br>GGGCATCAGCCAGCCCACCGTCGTATTCGTGAGCAAGAA<br>AGGGCTGCAAAAGATCCTCAACGTGCAAAAGAAGCTACC<br>GATCATACAAAAGATCATCATCATGGATAGCAAGACCGAC<br>TACCAGGGCTTCCAAAGCATGTACACCTTCGTGACTTCCC<br>ATTTGCCACCCGGCTTCAACGAGTACGACTTCGTGCCCG<br>AGAGCTTCGACCGGGACAAAACCATCGCCCTGATCATGA<br>ACAGTAGTGGCAGTACCGGATTGCCCAAGGGCGTAGCCC<br>TACCGCACCGCACCGCTTGTGTCCGATTCAGTCATGCCC<br>GCGACCCCATCTTCGGCAACCAGATCATCCCCGACACCG<br>CTATCCTCAGCGTGGTGCCATTTCACCACGGCTTCGGCAT<br>GTTCACCACGCTGGGCTACTTGATCTGCGGCTTTCGGGT<br>CGTGCTCATGTACCGCTTCGAGGAGGAGCTATTCTTGCG<br>CAGCTTGCAAGACTATAAGATTCAATCTGCCCTGCTGGTG<br>CCCACACTATTTAGCTTCTTCGCTAAGAGCACTCTCATCG<br>ACAAGTACGACCTAAGCAACTTGCACGAGATCGCCAGCG<br>GCGGGGCGCCGCTCAGCAAGGAGGTAGGTGAGGCCGTG<br>GCCAAACGCTTCCACCTACCAGGCATCCGCCAGGGCTAC<br>GGCCTGACAGAAACAACCAGCGCCATTCTGATCACCCCC<br>GAAGGGGACGACAAGCCTGGCGCAGTAGGCAAGGTGGT<br>GCCCTTCTTCGAGGCTAAGGTGGTGGACTTGGACACCGG<br>TAAGACACTGGGTGTGAACCAGCGCGGCGAGCTGTGCGT<br>CCGTGGCCCCATGATCATGAGCGGCTACGTTAACAACCC<br>CGAGGCTACAAACGCTCTCATCGACAAGGACGGCTGGCT<br>GCACAGCGGCGACATCGCCTACTGGGACGAGGACGAGC<br>ACTTCTTCATCGTGGACCGGCTGAAGAGCCTGATCAAATA<br>CAAGGGCTACCAGGTAGCCCCAGCCGAACTGGAGAGCAT<br>CCTGCTGCAACACCCCAACATCTTCGACGCCGGGGTCGC<br>CGGCCTGCCCGACGACGATGCCGGCGAGCTGCCCGCCG<br>CAGTCGTCGTGCTGGAACACGGTAAAACCATGACCGAGA<br>AGGAGATCGTGGACTATGTGGCCAGCCAGGTTACAACCG<br>CCAAGAAGCTGCGCGGTGGTGTTGTGTTCGTGGACGAGG<br>TGCCTAAAGGACTGACCGGCAAGTTGGACGCCCGCAAGA<br>TCCGCGAGATTCTCATTAAGGCCAAGAAGGGCGGCAAGA<br>TCGCCGTGGGAAGCGGAGAGGGCAGGGGAAGTCTTCTA<br>ACATGCGGGGACGTGGAGGAAAATCCCGGCCCCATGAG

			CGAGCTGATTAAGGAGAACATGCACATGAAGCTGTACATG GAGGGCACCGTGGACAACCATCACTTCAAGTGCACATCC GAGGGCGAAGGCAAGCCCTACGAGGGCACCCAGACCAT GAGAATCAAGGTGGTCGAGGGCGGCCCTCTCCCCTTCGC CTTCGACATCCTGGCTACTAGCTTCCTCTACGGCAGCAAG ACCTTCATCAACCACACCCAGGGCATCCCCGACTTCTTCA AGCAGTCCTTCCCTGAGGGCTTCACATGGGAGAGAGTCA CCACATACGAAGACGGGGGCGTGCTGACCGCTACCCAG GACACCAGCCTCCAGGACGGCTGCCTCATCTACAACGTC AAGATCAGAGGGGTGAACTTCACATCCAACGGCCCTGTG ATGCAGAAGAAAACACTCGGCTGGGAGGCCTTCACCGAG ACGCTGTACCCCGCTGACGGCGGCCTGGAAGGCAGAAA CGACATGGCCCTGAAGCTCGTGGGCGGGAGCCATCTGAT CGCAAACGCCAAGACCACATATAGATCCAAGAAACCCGCT AAGAACCTCAAGATGCCTGGCGTCTACTATGTGGACTACA GACTGGAAAGAATCAAGGAGGCCAACAACGAGACCTACG TCGAGCAGCACGAGGTGGCAGTGGCCAGATACTGCGACC TCCCTAGCAAACTGGGGCACAAGCTTAAT

Supplementary Table 4. List of all antibodies and cell dyes used in the study.

Conventional flow cytometry antibodies and dyes							
**Marker**	**Fluorophore**	**Clone**	**Host/Isotype**	**Reactivity**	**Catalog ref**	**Company**	**Final dilution**
CD4	BV711	OK14	Mouse IgG2b, κ	Human	317440	BioLegend	1:50
CD8a	FITC	HIT8a	Mouse IgG1, κ	Human	300906	BioLegend	1:100
CD19	BV510	HIB19	Mouse IgG1, κ	Human	302241	BioLegend	1:20
CD47	FITC	CC2C6	Mouse IgG1, κ	Human	323106	BioLegend	1:100
EGFRvIII	FITC	DH8.3	Mouse IgG1	Human	NBP2-50599F	Novus Biologicals	1:200
LAMP1	BB700	H4A3	Mouse IgG1, κ	Human	566558	BD Biosciences	1:200
Streptavidin	APC	None	None	Biotin	405207	BioLegend	1:200
Streptavidin	FITC	None	None	Biotin	405202	BioLegend	1:50
**Viability dye**					**Catalog ref**	**Company**	**Final dilution**
DAPI					564907	BD Biosciences	0.5X
DRAQ7					424001	BioLegend	1:1000
Zombie NIR					423105	BioLegend	1:5000

Phagocytosis spectral flow cytometry panel									
**Tagged cell**	**Protein**	**Dye**							
CAR T	mCherry								
U87	mTagBFP2	CellTracker Green, CMFDA Dye							
U251vIII	mTagBFP2								
**Surface marker**	**Fluorophore**	**Clone**	**Host/Isotype**	**Reactivity**	**Catalog ref**	**Lot**	**Company**	**Stock concentration**	**Final dilution**
CD3	BV605	SK7	Mouse IgG1, κ	Human	344835	B376001	BioLegend	0.1 mg/mL	1:50
CD11b	BV395	M1/70	Rat DA	Human/ Mouse	563553	2245854	BD Biosciences	0.2 mg/mL	1:50
CD86	BV785	IT2.2	Mouse IgG2b, κ	Human	305441	B376334	BioLegend	0.15 mg/mL	1:50
CD163	APC	GHI/61	Mouse IgG1, κ	Human	333609	B352117	BioLegend	0.1 mg/mL	1:50
CD206	PE/F700	15-2	Mouse IgG1, κ	Human	321153	B414740	BioLegend	50 µg/mL	1:50
CD209	PE	DCS-8C1	Mouse IgG1, κ	Human	343004	B377998	BioLegend	0.1 mg/mL	1:50
HLA-DR	APC/F810	L243	Mouse IgG2a, κ	Human	307673	B370992	BioLegend	50 µg/mL	1:50

SIGLEC-1	PE/Cy7	7-239	Mouse IgG1, κ	Human	346013	B354805	BioLegend	0.1 mg/mL	1:50
**Viability dye**					**Catalog ref**		**Company**		**Final dilution**
Zombie UV					423107		BioLegend		1:500
**Fc-blocking antibody**	**Target**	**Clone**	**Host/Isotype**	**Reactivity**	**Catalog ref**	**Lot**	**Company**	**Stock concentration**	**Final dilution**
Human TruStain FcX	CD16/CD32/CD64	Unknown	Unknown	Human	422302	B417359	BioLegend	Unknown	1:50

**In vivo antibody treatments**								
**Condition**	**Target**	**Clone**	**Host/Isotype**	**Reactivity**	**Catalog ref**	**Company**	**Application**	**Final concentration/dose**
aCCL3	CCL3	Polyclonal	Goat IgG	Human	AF-270-SP	R&D Systems	Mouse intraperitoneal injection	50 ng per injection
aCD47	CD47	B6.H12	Mouse IgG1, κ	Human	BE0019-1	Bio X Cell	On-cell blocking assay	10 µg/mL
aCD47	CD47	B6.H12	Mouse IgG1, κ	Human	BE0019-1	Bio X Cell	Mouse intratumoral injection	5 µg per injection
aCD47	CD47	B6.H12	Mouse IgG1, κ	Human	BE0019-1	Bio X Cell	Mouse intraperitoneal injection	100 µg per injection
Isotype (Goat IgG)	Unknown	Polyclonal	Goat IgG	Non-reactive	AB-108-C	R&D Systems	Mouse intraperitoneal injection	50 ng per injection
Isotype (Mouse IgG1)	Unknown	MOPC-21	Mouse IgG1, κ	Non-reactive	BE0083	Bio X Cell	On-cell blocking assay	10 µg/mL
Isotype (Mouse IgG1)	Unknown	MOPC-21	Mouse IgG1, κ	Non-reactive	BE0083	Bio X Cell	Mouse intratumoral injection	5 µg per injection

**On-off target CAR spectral flow cytometry panel**									
**Tagged cell**	**Protein**								
CAR T	mCherry								
U87	mTagBFP2								
U251vIII	mTagBFP2								
**Surface marker**	**Fluorophore**	**Clone**	**Host/Isotype**	**Reactivity**	**Catalog ref**	**Lot**	**Company**	**Stock concentration**	**Final dilution**
CD3	BV605	SK7	Mouse IgG1, κ	Human	344835	B376001	BioLegend	0.1 mg/mL	1:50
CD4	BV711	OKT4	Mouse IgG2b, κ	Human	317440	B261488	BioLegend	60 µg/mL	1:50
CD8a	SB574	SK1	Mouse IgG1, κ	Human	344783	B413964	BioLegend	50 µg/mL	1:50

CD11b	BUV395	M1/70	Rat DA	Human/ Mouse	563553	3346840	BD Biosciences	0.2 mg/mL	1:1600
CD11c	BUV496	N418	Armenian Hamster IgG2	Mouse	750450	4079322	BD Biosciences	0.2 mg/mL	1:100
CD25	PerCP/ F806	M-A251	Mouse IgG1, κ	Human	356163	B416234	BioLegend	0.2 mg/mL	1:50
CD45	BUV737	30-F11	Rat LOU	Mouse	748371	2322187	BD Biosciences	0.2 mg/mL	1:4000
CD64	PE/Cy5	X54-5/7.1	Mouse IgG1, κ	Mouse	139331	B403177	BioLegend	0.2 mg/mL	1:400
CD69	APC/Cy7	FN50	Mouse IgG1, κ	Human	310913	B284261	BioLegend	0.2 mg/mL	1:75
CD206	AF700	C068C2	Rat IgG2a, κ	Mouse	141734	B384250	BioLegend	0.5 mg/mL	1:100
EGFRvIII	FITC	DH8.3	Mouse IgG1	Human	NBP2-50599F	D156917	Novus Biologicals	-	1:200
F4/80	BUV805	T45-2342	Rat WI	Mouse	749282	3200633	BD Biosciences	0.2 mg/mL	1:500
FAP	AF647	427819	Mouse IgG1	Human	FAB3715R	AFEM0321011	R&D Systems	0.2 mg/mL	1:800
CD49d	BUV563	9C10(MFR4.B)	Rat LEW	Mouse	741243	3200542	BD Biosciences	0.2 mg/mL	1:800
LAMP1	BB700	H4A3	Mouse IgG1, κ	Human	566558	9080923	BD Biosciences	-	1:200
Ly6C	BV785	HK1.4	Rat IgG2c, κ	Mouse	128041	B386418	BioLegend	0.2 mg/mL	1:12000
Ly6G	BUV661	1A8	Rat LEW	Mouse	741587	4012615	BD Biosciences	0.2 mg/mL	1:500
MHC-II	NF610-70S	M5/114.152	Rat IgG2b, κ	Mouse	M024T02B06	2925490	ThermoFisher Scientific	0.1 mg/mL	1:1600
P2RY12	APC/F810	S16007D	Rat IgG2b, κ	Mouse	848013	B39443	BioLegend	0.2 mg/mL	1:50
**Intracellular marker**	**Fluorophore**	**Clone**	**Host/Isotype**	**Reactivity**	**Catalog ref**	**Lot**	**Company**	**Stock concentration**	**Final dilution**
GZMB	PE	QA16A02	Mouse IgG1, κ	Human/ mouse	372207	B402217	BioLegend	100 µg/mL	1:50
IFNG	PE/Cy7	4S.B3	Mouse IgG1, κ	Human	502527	B374513	BioLegend	50 µg/mL	1:50
**Viability dye**					**Catalog ref**		**Company**		**Final dilution**
Zombie Aqua					423101		BioLegend		1:1000
**Fc-blocking antibody**	**Target**	**Clone**	**Host/Isotype**	**Reactivity**	**Catalog ref**	**Lot**	**Company**	**Stock concentration**	**Final dilution**
Human TruStain FcX	CD16/CD32/CD64	Unknown	Unknown	Human	422302	B369982	BioLegend	Unknown	1:50
TruStain FcX	CD16/CD32	93	Rat IgG2a, λ	Mouse	101320	B380119	BioLegend	0.5 mg/mL	1:50

**Immunohistochemistry antibodies and reagents**						
**Primary antibody**	**Clone**	**Host/Isotype**	**Reactivity**	**Catalog ref**	**Company**	**Dilution**
CD3	polyclonal	Rabbit IgG	Mouse/Rat/Human	ab5690	Abcam	1:100

CD68	E3O7V	Rabbit IgG	Mouse	97778S	Cell Signaling Technology	1:100
**Detection reagent**			**Reactivity**	**Catalog ref**	**Company**	**Dilution**
UIP anti-rabbit			Rabbit	414142F	Nicherei Biosciences Inc.	None

**Immunofluorescence antibodies and dyes**							
**Primary antibody**		**Clone**	**Host/Isotype**	**Reactivity**	**Catalog ref**	**Company**	**Final dilution**
CD3		CD3-12	Rat IgG1	Human	MCA1477	Bio-Rad	1:100
CD206		E6T5J	Rabbit IgG	Human/Mouse/Rat	24595S	Cell Signaling Technology	1:200
EGFRvIII		RM419	Rabbit IgG	Human	MA5-36216	ThermoFisher Scientific	1:1000
GFAP		D1F4Q	Rabbit IgG	Human/Mouse/Rat	12389S	Cell Signaling Technology	1:500
IBA1		Polyclonal	Goat IgG	Human/Mouse/Rat	NB100-1028	Novus Biologicals	1:500
Ki67		SolA15	Rat IgG2a, κ	Human/Mouse/Rat	14-5698-82	ThermoFisher Scientific	1:500
TMEM119		28-3	Rabbit IgG	Mouse	ab209064	Abcam	1:500
**Secondary antibody**	**Conjugate**	**Clone**	**Host/Isotype**	**Reactivity**	**Catalog ref**	**Company**	**Final dilution**
Donkey anti-Rabbit	AF647	Polyclonal	Donkey IgG	Rabbit (H+L)	711-605-152	Jackson ImmunoResearch	1:300
Donkey anti-Rat	AF555	Polyclonal	Donkey IgG	Rat (H+L)	A78945	Invitrogen	1:1000
Donkey anti-Goat	AF488	Polyclonal	Donkey IgG	Goat (H+L)	705-545-147	Jackson ImmunoResearch	1:300
**Nuclear stain**					**Catalog ref**	**Company**	**Final dilution**
DAPI					564907	BD Biosciences	1X

**GAM modulation spectral flow cytometry panel**									
**Tagged cell**	**Protein**								
CAR T	mCherry								
Tumor	mTagBFP2								
**Surface marker**	**Fluorophore**	**Clone**	**Host/Isotype**	**Reactivity**	**Catalog ref**	**Lot**	**Company**	**Stock concentration**	**Final dilution**
AXL	AF488	175128	Rat IgG2a	Mouse	FAB8541G	AEWU0422121	R&D Systems	0.2 mg/mL	1:200
CD4	BUV496	GK1.5	Rat LEW	Human/Mouse	612952	2272360	BD Biosciences	0.2 mg/mL	1:800
CD8a	BV650	53-6.7	Rat IgG2a, κ	Human/Mouse	100742	B368585	BioLegend	0.2 mg/mL	1:200
CD11b	BV510	M1/70	Rat IgG2b, κ	Human/Mouse	101245	B376886	BioLegend	80 μg/mL	1:1600

CD11c	PE/Cy7	N418	Armenian Hamster IgG	Mouse	117317	B346714	BioLegend	0.2 mg/mL	1:3200
CD45	BUV737	30-F11	Rat LOU	Mouse	748371	23222187	BD Biosciences	0.2 mg/mL	1:6000
CD86	PE/Cy5	GL-1	Rat IgG2a, κ	Mouse	105016	B240898	BioLegend	0.2 mg/mL	1:1600
CD206	AF700	C068C2	Rat IgG2a, κ	Mouse	141734	B384250	BioLegend	0.5 mg/mL	1:100
EGFRvIII	PerCP	DH8.3	Mouse IgG1	Human	NBP2-50599PCP	D139305	Novus Biologicals	0.3 mg/mL	1:200
F4/80	BV605	BM8	Rat IgG2a, κ	Mouse	123133	B386425	BioLegend	0.1 mg/mL	1:50
Ly6C	BV785	HK1.4	Rat IgG2c, κ	Mouse	128041	B371649	BioLegend	0.2 mg/mL	1:12000
Ly6G	BUV563	1A8	Rat LEW	Mouse	612921	2343097	BD Biosciences	0.2 mg/mL	1:2500
MERTK	BV711	2B10C42	Rat IgG2a, κ	Mouse	151515	B354635	BioLegend	0.2 mg/mL	1:100
MHC-II	NF610-70S	M5/114.152	Rat IgG2b, κ	Mouse	M024T02B06	2511116	ThermoFisher Scientific	0.1 mg/mL	1:1600
P2RY12	APC/F810	S16007D	Rat IgG2b, κ	Mouse	848013	B343968	BioLegend	0.2 mg/mL	1:50
Siglec-H	APC	551	Rat IgG1, κ	Mouse	129612	B286621	BioLegend	0.2 mg/mL	1:150
XCR1	BV421	ZET	Mouse IgG2b, κ	Mouse/Rat	148216	B377550	BioLegend	0.2 mg/mL	1:100
**Intracellular marker**	**Fluorophore**	**Clone**	**Host/Isotype**	**Reactivity**	**Catalog ref**	**Lot**	**Company**	**Stock concentration**	**Final dilution**
TNF	PE	MP6-XT22	Rat IgG1, κ	Mouse	506305	B336284	BioLegend	0.2 mg/mL	1:100
**Viability dye**					**Catalog ref**		**Company**		**Final dilution**
Zombie NIR					423105		BioLegend		1:1600
**Fc-blocking antibody**	**Target**	**Clone**	**Host/Isotype**	**Reactivity**	**Catalog ref**	**Lot**	**Company**	**Stock concentration**	**Final dilution**
Human TruStain FcX	CD16/CD32/CD64	Unknown	Unknown	Human	422302	B369982	BioLegend	Unknown	1:50
TruStain FcX	CD16/CD32	93	Rat IgG2a, λ	Mouse	101320	B380119	BioLegend	0.5 mg/mL	1:50

**Immunofluorescence pharmacoscopy panel**								
**Tagged cell**	**Protein**							**Co-staining panel**
CAR T	mCherry							1 and 2
**Conjugated antibody**	**Fluorophore**	**Clone**	**Host/Isotype**	**Reactivity**	**Catalog ref**	**Company**	**Final dilution**	**Co-staining panel**
CD3	AF488	UCHT1	Mouse IgG1, κ	Human	300415	BioLegend	1:300	1
CD14	AF647	HCD14	Mouse IgG1, κ	Human	325612	BioLegend	1:300	1

Primary antibody		Clone	Host/Isotype	Reactivity	Catalog ref	Company	Final dilution	Co-staining panel
EGFRvIII		RM419	Rabbit IgG	Human	MA5-36216	ThermoFisher Scientific	1:150	2
NESTIN		10C2	Mouse IgG1, κ	Human	656802	BioLegend	1:150	2
**Secondary antibody**	**Conjugate**	**Clone**	**Host/Isotype**	**Reactivity**	**Catalog ref**	**Company**	**Final dilution**	**Co-staining panel**
Goat anti-Mouse	AF488	Polyclonal	Goat IgG	Mouse (H+L)	A32723TR	Invitrogen	1:500	2
Goat anti-Rabbit	AF647	Polyclonal	Goat IgG	Rabbit (H+L)	A32733	Invitrogen	1:500	2
**Nuclear stain**					**Catalog ref**	**Company**	**Final concentration**	
DAPI					422801	BioLegend	4 µg/mL	

CAR T cell and cetuximab combination spectral flow cytometry panel									
**Tagged cell**	**Protein**								
CAR T	mCherry								
U87	mTagBFP2								
**Surface marker**	**Fluorophore**	**Clone**	**Host/Isotype**	**Reactivity**	**Catalog ref**	**Lot**	**Company**	**Stock concentration**	**Final dilution**
CD11b	BUV395	M1/70	Rat DA	Human/Mouse	563553	3346840	BD Biosciences	0.2 mg/mL	1:1000
CD11c	BUV496	N418	Armenian Hamster IgG2	Mouse	750450	4079322	BD Biosciences	0.2 mg/mL	1:200
CD45	BUV737	30-F11	Rat LOU	Mouse	748371	2322187	BD Biosciences	0.2 mg/mL	1:4000
CD80	BV605	16-10A1	Armenian Hamster IgG	Mouse	104729	B357122	BioLegend	50 µg/mL	1:200
CD86	PE/Cy5	GL-1	Rat IgG2a, κ	Mouse	105016	B376879	BioLegend	0.2 mg/mL	1:1000
CD163	APC/Cy7	S15049I	Rat IgG2a, κ	Mouse	155323	B383053	BioLegend	0.2 mg/mL	1:100
CD206	AF700	C068C2	Rat IgG2a, κ	Mouse	141734	B384250	BioLegend	0.5 mg/mL	1:100
CD274	PE/Cy7	MIH6	Rat IgG2a, λ	Mouse	153613		BioLegend	0.2 mg/mL	1:800
CD49d	BUV563	9C10(MFR4.B)	Rat LEW	Mouse	741243	3200542	BD Biosciences	0.2 mg/mL	1:800
Ly6C	BV785	HK1.4	Rat IgG2c, κ	Mouse	128041	B386418	BioLegend	0.2 mg/mL	1:10000
Ly6G	BUV661	1A8	Rat LEW	Mouse	741587	4012615	BD Biosciences	0.2 mg/mL	1:500

MHC-II	NF610-70S	M5/114.152	Rat IgG2b, κ	Mouse	M024T02B06	2925490	ThermoFisher Scientific	0.1 mg/mL	1:1000
P2RY12	APC/F810	S16007D	Rat IgG2b, κ	Mouse	848013	B39443	BioLegend	0.2 mg/mL	1:50
Siglec-1	FITC	3D6.112	Rat IgG2a, κ	Mouse	142405	B388729	BioLegend	0.5 mg/mL	1:100
XCR1	APC	ZET	Mouse IgG2b, κ	Mouse/Rat	148205	B349249	BioLegend	0.2 mg/mL	1:100
**Viability dye**					**Catalog ref**		**Company**		**Final dilution**
Zombie Aqua					423101		BioLegend		1:1000
**Fc-blocking antibody**	**Target**	**Clone**	**Host/Isotype**	**Reactivity**	**Catalog ref**	**Lot**	**Company**	**Stock concentration**	**Final dilution**
Human TruStain FcX	CD16/CD32/CD64	Unknown	Unknown	Human	422302	B369982	BioLegend	Unknown	1:50
TruStain FcX	CD16/CD32	93	Rat IgG2a, λ	Mouse	101320	B380119	BioLegend	0.5 mg/mL	1:50

Supplementary Table 5. Animal scoring sheets for longitudinal monitoring of experimental outcomes.

**Injection date:**
**Mouse strain:**
**Project/cells injected:**
**Cage:**
**Sex:**

**Mouse**	**Date**															
	Intervention															
	Weight															
	Tumor															
	Score															
	Intervention															
	Weight															
	Tumor															
	Score															
	Intervention															
	Weight															
	Tumor															
	Score															
	Intervention															
	Weight															
	Tumor															
	Score															
	Intervention															
	Weight															
	Tumor															
	Score															
	Intervention															
	Weight															
	Tumor															
	Score															

Administered **drug(s)**	Name, dose, and route
Bioluminescence **imaging**	Tumor detected (+/-)
Euthanasia **score**	0-4: Normal/mild distress, monitor 1-2x/week
	5-9: Moderate distress, monitor daily
	10-15: Severe distress, euthanasia

**Euthanasia score**																
**Type**	**Score**	**Observations**	**Date**													

		**Animal**													
Appearance	0<br>1<br>2<br>3	Normal posture and smooth fur<br>Hunched posture OR slightly ruffled fur<br>Hunched posture AND slightly ruffled fur<br>Hunched posture AND completely ruffled fur													
Weight	0<br>1<br>2<br>3	No loss or loss of less than 10% of body weight<br>Loss of 10% of body weight<br>Loss of 15% of body weight<br>Loss of 20% of body weight													
Signs	0<br>1<br>2<br>3	Normal respiratory rate and pattern<br>Slight changes, increased respiratory rate only<br>Increased rate with abdominal breathing<br>Decreased rate with abdominal breathing													
Movement	0<br>1<br>2<br>3	Moves normally around the cage<br>Moves slowly around the cage<br>Moves only when touched<br>Does not move OR self-mutilation													
Behavior	0<br>1<br>2<br>3	Normal (moves when cage is disturbed, runs from hand)<br>Attenuated or exaggerated responses (moves away briskly)<br>Moderate change (moves away slowly)<br>Reacts violently OR unresponsive													
Euthanasia	3 is scored in any one category OR at least 10 is scored across different categories														

Supplementary Table 6. Clinical information of GBM patient-derived material.

Patient code	EGFRvIII mutation	EGFRwt amplification status	IDH status	MGMT promotor methylation status	Pre-existing conditions	Steroid dosage during surgery	Additional info
BTB 635	Detected	Amplified	Wild-type	Methlyated	Chronic alcoholism, depression	None	None
BTB 639	Detected	Amplified	Wild-type	Methlyated	None	10 mg of fortecortin	Postoperative wound infection
BTB 676	Detected	Amplified	Wild-type	Methylated	Arterial hypertension, structural epilepsy, deep vein thrombosis	None	None
BTB 691	Not detected	Non amplified	Wild-type	Non methylated	Arterial hypertension	None	Early postoperative meningitis
BTB 692	Detected	Amplified	Wild-type	Methylated	Prostate carcinoma, atrial fibrillation	4 mg of fortecortin	None
BTB 716	Not detected	Non amplified	Wild-type	Non methylated	Prostate carcinoma, arterial hypertension, diabetes mellitus	4 mg of fortecortin	None
BTB 739	Detected	Amplified	Wild-type	Methlyated	Chronic obstructive pulmonary disease	None	None

Supplementary Table 7. RT-qPCR primer sequences.

Primer	Template strand (5'-3')	Primer length (bp)	Product length (bp)
mCherry Forward	GCAGAAGAAGACCATGGGCT	20	82
mCherry Reverse	CTGCTTGATCTCGCCCTTCA	20	
SGRP Forward	TCACCTCTTTGTTCCCCGTG	20	104
SGRP Reverse	GGTGACCCTAGGAAAGTGGC	20	
EGFRvIII Forward	CTGCTGGCTGCGCTCTG	17	72
EGFRvIII Reverse	GTGATCTGTCACCACATAATTACCTTTC	28	
EGFRwt Forward	TATGTCCTCATTGCCCTCAACA	22	62
EGFRwt Reverse	CTGATGATCTGCAGGTTTTCCA	22	
GAPDH Forward	GTCTCCTCTGACTTCAACAGCG	22	131
GAPDH Reverse	ACCACCCTGTTGCTGTAGCCAA	22	
**Primer**	**Kit**	**Catalog ref**	**Company**
TBP	QuantiTect Primer Assay	QT00000721	QIAGEN
