# Supplementary material for: Enhancing anti-EGFRvIII CAR T cell therapy against glioblastoma with a paracrine SIRPγ-derived CD47 blocker
Source: Nat Commun. 2024 Nov 9;15:9718. doi: 10.1038/s41467-024-54129-w (PMC11550474; doi:10.1038/s41467-024-54129-w)
Supplement: Supplementary file 2 — Description of Additional Supplementary Files [file 41467_2024_54129_MOESM2_ESM.pdf]

Description of Additional Supplementary Files

Supplementary Data 1. Protein expression results and accession IDs of LC–MS-identified proteins from aEGFRvIII CAR- and aEGFRvIII-SGRP CAR-conditioned media.

Supplementary Data 2. Raw normalized protein expression (NPX) data obtained by proximity extension assay using an Olink Target 96 immuno-oncology panel.

Supplementary Data 3. Source data for cell counts of IHC and IF imaging analyses.

Supplementary Data 4. Source data for pharmacoscopy analysis.

Supplementary Movie 1. U251vIII + aCD19 CAR co-culture time-lapse.

Supplementary Movie 2. U251vIII + aCD19-SGRP CAR co-culture time-lapse.

Supplementary Movie 3. U251vIII + aEGFRvIII CAR co-culture time-lapse.

Supplementary Movie 4. U251vIII + aEGFRvIII-SGRP CAR co-culture time-lapse.

Supplementary Movie 5. U251 + aCD19 CAR co-culture time-lapse.

Supplementary Movie 6. U251 + aCD19-SGRP CAR co-culture time-lapse.

Supplementary Movie 7. U251 + aEGFRvIII CAR co-culture time-lapse.

Supplementary Movie 8. U251 + aEGFRvIII-SGRP CAR co-culture time-lapse.
