# Supplementary material for: Enhancing anti-EGFRvIII CAR T cell therapy against glioblastoma with a paracrine SIRPγ-derived CD47 blocker
Source: Nat Commun. 2024 Nov 9;15:9718. doi: 10.1038/s41467-024-54129-w (PMC11550474; doi:10.1038/s41467-024-54129-w)
Supplement: Supplementary file 15 — Reporting Summary [file 41467_2024_54129_MOESM15_ESM.pdf]

nature portfolio

Corresponding author(s): Tomás A. Martins, Gregor Hutter

Last updated by author(s): Sep 27, 2024

Reporting Summary

Nature Portfolio wishes to improve the reproducibility of the work that we publish. This form provides structure for consistency and transparency in reporting. For further information on Nature Portfolio policies, see our Editorial Policies and the Editorial Policy Checklist.

Statistics

For all statistical analyses, confirm that the following items are present in the figure legend, table legend, main text, or Methods section.

n/a	Confirmed	
☐	☒	The exact sample size (n) for each experimental group/condition, given as a discrete number and unit of measurement
☐	☒	A statement on whether measurements were taken from distinct samples or whether the same sample was measured repeatedly
☐	☒	The statistical test(s) used AND whether they are one- or two-sided<br>*Only common tests should be described solely by name; describe more complex techniques in the Methods section.*
☐	☒	A description of all covariates tested
☐	☒	A description of any assumptions or corrections, such as tests of normality and adjustment for multiple comparisons
☐	☒	A full description of the statistical parameters including central tendency (e.g. means) or other basic estimates (e.g. regression coefficient) AND variation (e.g. standard deviation) or associated estimates of uncertainty (e.g. confidence intervals)
☐	☒	For null hypothesis testing, the test statistic (e.g. F, t, r) with confidence intervals, effect sizes, degrees of freedom and P value noted<br>*Give P values as exact values whenever suitable.*
☒	☐	For Bayesian analysis, information on the choice of priors and Markov chain Monte Carlo settings
☐	☒	For hierarchical and complex designs, identification of the appropriate level for tests and full reporting of outcomes
☒	☐	Estimates of effect sizes (e.g. Cohen's d, Pearson's r), indicating how they were calculated

Our web collection on statistics for biologists contains articles on many of the points above.

Software and code

Policy information about availability of computer code

Data collection	Animal monitoring: Microsoft Excel v16.14.1 (Microsoft, USA)<br>Bioluminescence imaging: NEWTON v7 (Vilber, France)<br>Conventional FC: Cytexpert v2.6 (Beckman Coulter, USA)<br>FC sorting: BD FACSChorus v3.0 (BD Biosciences, USA), BD FACSDiva v6.0 (BD Biosciences, USA)<br>IHC and IF imaging: NIS-Elements v5.11.00 (Nikon, Japan)<br>Live cell imaging: Incucyte v2020B (Sartorius, Germany)<br>RT-qPCR: Precision Melt Analysis v1.2 (Bio-Rad, USA)<br>Spectral FC: Spectroflo v3.3 (Cytek Biosciences, USA)

Data analysis	Animal monitoring: GraphPad Prism v10 (GraphPad, USA)<br>Bioluminescence imaging: NEWTON v7 (Vilber, France), GraphPad Prism v10 (GraphPad, USA)<br>Conventional FC: FlowJo v10 (BD Biosciences, USA), GraphPad Prism v10 (GraphPad, USA)<br>IHC and IF imaging: NIS-Elements v5.11.00 (Nikon, Japan), Fiji v2.9.0, QuPath v0.4.3 and v.0.5.0, GraphPad Prism v10 (GraphPad, USA)<br>LC–MS/MS: Progenesis QI v2.0 (Nonlinear Dynamics, UK), R v4.3.1, GraphPad Prism v10 (GraphPad, USA)<br>Live cell imaging: Incucyte v2020B (Sartorius, Germany), GraphPad Prism v10 (GraphPad, USA)<br>Pharmacoscopy: CellProfiler v2.2.0, MATLAB vR2021b<br>RT-qPCR: Precision Melt Analysis v1.2 (Bio-Rad, USA), GraphPad Prism v10 (GraphPad, USA)<br>Spectral FC: Spectroflo v3.3 (Cytek Biosciences, USA), FlowJo v10 (BD Biosciences, USA), R v4.3.1, GraphPad Prism v10 (GraphPad, USA)

For manuscripts utilizing custom algorithms or software that are central to the research but not yet described in published literature, software must be made available to editors and reviewers. We strongly encourage code deposition in a community repository (e.g. GitHub). See the Nature Portfolio guidelines for submitting code & software for further information.

Data

Policy information about availability of data

All manuscripts must include a data availability statement. This statement should provide the following information, where applicable:

- Accession codes, unique identifiers, or web links for publicly available datasets
- A description of any restrictions on data availability
- For clinical datasets or third party data, please ensure that the statement adheres to our policy

The protein mass spectrometry data generated in this study have been deposited in the MassIVE database under accession code PXD054059 [https://massive.ucsd.edu/ProteoSAFe/dataset.jsp?task=a1b0183b582548eda092f2487ba065fb]. The remaining data are available within the Article, Supplementary Information, or Source Data file. Any additional requests or clarification may be forwarded to the corresponding authors. Original R scripts used in the study are available in a GitHub repository under DOI: 10.5281/zenodo.13819903 [https://github.com/BTITLab/SGRP_CART].

Research involving human participants, their data, or biological material

Policy information about studies with human participants or human data. See also policy information about sex, gender (identity/presentation), and sexual orientation and race, ethnicity and racism.

Reporting on sex and gender	Our CAR T cells were generated from anonymous healthy donors with undisclosed sex. Patient samples were derived from a majority male cohort. The study included seven GBM patients, six males and one female. Participant sex was reported as assigned in legal documents.
Reporting on race, ethnicity, or other socially relevant groupings	Our CAR T cells were generated from anonymous healthy donors with undisclosed race, ethnicity, or other socially relevant groupings. GBM samples were derived from patients from whom we did not collect information on race, ethnicity, or other socially relevant groupings.
Population characteristics	Our CAR T cells were generated from anonymous healthy donors. Patient samples were derived from a cohort with an average age of 67.7 years (55 to 82 years (median age 64)). Other relevant population characteristics like the preexisting conditions of GBM patients are summarized in Supplementary Table 6.
Recruitment	For blood from human healthy donors, the only selection criterium was a confirmed negative result for bloodborne infections. Patient-derived tumor samples were selected based on the criterium that samples were not stored for longer than 3 years in our biobank. The final selection of tumor samples included in the pharmacoscopy experiment of Figure 7 was based on a positive EGFRvIII mutation status determined by qPCR.
Ethics oversight	Patient-derived samples were acquired following the Swiss Human Research Act and Institutional Ethics Commission (EKNZ Ethikkommission Nordwest- und Zentralschweiz 02019-02358). The study followed the ethical principles of the Declaration of Helsinki, regulatory requirements, and the Code of Good Clinical Practice. All patients gave written informed consent for tumor biopsy collection and signed a declaration enabling the use of their biopsy specimens in scientific research (Req-2019-00553).

Note that full information on the approval of the study protocol must also be provided in the manuscript.

Field-specific reporting

Please select the one below that is the best fit for your research. If you are not sure, read the appropriate sections before making your selection.

☒ Life sciences ☐ Behavioural & social sciences ☐ Ecological, evolutionary & environmental sciences

For a reference copy of the document with all sections, see nature.com/documents/nr-reporting-summary-flat.pdf

Life sciences study design

All studies must disclose on these points even when the disclosure is negative.

Sample size	No power analyses were used to predetermine sample sizes in experiments. However, sample sizes were chosen on the basis of prior

literature using similar experimental paradigms.

Data exclusions	No data was excluded.
Replication	All experiments were repeated at least once producing similar results, except the pharmacoscopy experiment of Figure 7 that was performed once due to the scarcity of human material, particularly EGFRvIII+ GBM samples. In vitro experiments were replicated using different batches of freshly produced CAR T cells from different donors. Large experiments involving animals were split into 2 or 3 smaller experiments, as specified in Figure/Supplementary Figure legends and the data from animal cohorts were pooled for analysis. Within the same in vivo experiment, comparable results were obtained across animal cohorts. Repetition experiments were performed by different researchers whenever possible, to ensure reproducibility. All attempts of replication were successful except for in vitro phagocytosis assays, where some donor-specific effects were occasionally observed. The 'negative' result shown in Supplementary Figure 2g was replicated once.
Randomization	Animals in GBM xenograft experiments were allocated to experimental groups based on pre-therapy tumor burden determined by bioluminescence imaging, ensuring that groups were balanced with respect to tumor size prior to treatment. Animals in lymphoma xenograft experiments were randomly allocated to experimental groups because in that experimental setup, tumor size differences were not measurable prior to treatment. Allocation does not apply to other types of experiments in the study.
Blinding	Blinding was not possible in most experiments because the majority of experiments were performed by a single researcher. Exceptions were: (1) the pharmacoscopy experiment of Figure 7, where the experimenter was blinded to GBM patient EGFRvIII status and treatment during the co-culture and image analysis; and (2) the repetitions of in vivo experiments of Figures 3 and 8, where treatments were administered by researchers blinded to the group allocations. Outcome measurements, including humane endpoint determination and tumor size monitoring were performed by blinded assessors in these repetition experiments. Additionally, in all in vivo experiments, animals of different treatment groups were co-housed in the same cage to blind assessors in determining the humane endpoint.<br>Except for point (1) above, there was no blinding during data analysis.

Reporting for specific materials, systems and methods

We require information from authors about some types of materials, experimental systems and methods used in many studies. Here, indicate whether each material, system or method listed is relevant to your study. If you are not sure if a list item applies to your research, read the appropriate section before selecting a response.

Materials & experimental systems

n/a	Involved in the study
☐	☒ Antibodies
☐	☒ Eukaryotic cell lines
☒	☐ Palaeontology and archaeology
☐	☒ Animals and other organisms
☐	☒ Clinical data
☒	☐ Dual use research of concern
☒	☐ Plants

Methods

n/a	Involved in the study
☒	☐ ChIP-seq
☐	☒ Flow cytometry
☒	☐ MRI-based neuroimaging

Antibodies

Antibodies used

Conventional FC antibodies:
CD4 BV711: supplier BioLegend, catalog number 317440, clone OK14, lot unknown
CD8a FITC: supplier BioLegend, catalog number 300906, clone HIT8a, lot unknown
CD19 BV510: supplier BioLegend, catalog number 302241, clone HIB19, lot unknown
CD47 FITC: supplier BioLegend, catalog number 323106, clone CC2C6, lot unknown
EGFRvIII FITC: supplier Novus Biologicals, catalog number NBP2-50599F, clone DH8.3, lot unknown
LAMP1 BB700: supplier BD Biosciences, catalog number 566558, clone H4A3, lot unknown
Streptavidin APC: supplier BioLegend, catalog number 405207, lot unknown
Streptavidin FITC: supplier BioLegend, catalog number 405202, lot unknown

Phagocytosis spectral FC antibodies:
CD3 BV605: supplier BioLegend, catalog number 344835, clone SK7, lot B376001
CD11b BV395: supplier BD Biosciences, catalog number 563553, clone M1/70, lot 2245854
CD86 BV785: supplier BioLegend, catalog number 305441, clone IT2.2, lot B376334
CD163 APC: supplier BioLegend, catalog number 333609, clone GHI/61, lot B352117
CD206 PE/F700: supplier BioLegend, catalog number 321153, clone 15-2, lot B414740
CD209 PE: supplier BioLegend, catalog number 343004, clone DCS-8C1, lot B377998
HLA-DR APC/F810: supplier BioLegend, catalog number 307673, clone L243, lot B370992
SIGLEC-1 PE/Cy7: supplier BioLegend, catalog number 346013, clone 7-239, lot B354805
Human TruStain FcX: supplier BioLegend, catalog number 422302, clone unknown, lot B417359

In vivo antibody treatments:
aCCL3: supplier R&D Systems, catalog number AF-270-SP, clone polyclonal, lot unknown

aCD47: supplier Bio X Cell, catalog number BE0019-1, clone B6.H12, lot unknown
Isotype (Goat IgG): supplier R&D Systems, catalog number AB-108-C, clone polyclonal, lot unknown
Isotype (Mouse IgG1): supplier Bio X Cell, catalog number BE0083, clone MOPC-21, lot unknown

On-off target CAR spectral FC antibodies:
CD3 BV605: supplier BioLegend, catalog number 344835, clone SK7, lot B376001
CD4 BV711: supplier BioLegend, catalog number 317440, clone OKT4, lot B261488
CD8a SB574: supplier BioLegend, catalog number 344783, clone SK1, lot B413964
CD11b BUV395: supplier BD Biosciences, catalog number 563553, clone M1/70, lot 3346840
CD11c BUV496: supplier BD Biosciences, catalog number 750450, clone N418, lot 4079322
CD25 PerCP/F806: supplier BioLegend, catalog number 356163, clone M-A251, lot B416234
CD45 BUV737: supplier BD Biosciences, catalog number 748371, clone 30-F11, lot 2322187
CD64 PE/Cy5: supplier BioLegend, catalog number 139331, clone X54-5/7.1, lot B403177
CD69 APC/Cy7: supplier BioLegend, catalog number 310913, clone FN50, lot B284261
CD206 AF700: supplier BioLegend, catalog number 141734, clone C068C2, lot B384250
EGFRvIII FITC: supplier Novus Biologicals, catalog number NBP2-50599F, clone DH8.3, lot D156917
F4/80 BUV805: supplier BD Biosciences, catalog number 749282, clone T45-2342, lot 3200633
FAP AF647: supplier R&D Systems, catalog number FAB3715R, clone 427819, lot AFEM0321011
CD49d BUV563: supplier BD Biosciences, catalog number 741243, clone 9C10(MFR4.B), lot 3200542
LAMP1 BB700: supplier BD Biosciences, catalog number 566558, clone H4A3, lot 9080923
Ly6C BV785: supplier BioLegend, catalog number 128041, clone HK1.4, lot B386418
Ly6G BUV661: supplier BD Biosciences, catalog number 741587, clone 1A8, lot 4012615
MHC-II NF610-70S: supplier ThermoFisher Scientific, catalog number M024T02B06, clone M5/114.152, lot 2925490
P2RY12 APC/F810: supplier BioLegend, catalog number 848013, clone S16007D, lot B39443
GZMB PE: supplier BioLegend, catalog number 372207, clone QA16A02, lot B402217
IFNG PE/Cy7: supplier BioLegend, catalog number 502527, clone 4S.B3, lot B374513
Human TruStain FcX: supplier BioLegend, catalog number 422302, clone unknown, lot B369982
TruStain FcX: supplier BioLegend, catalog number 101320, clone 93, lot B380119

IHC antibodies:
CD3: supplier Abcam, catalog number ab5690, clone polyclonal, lot unknown
CD68: supplier Cell Signaling Technology, catalog number 97778S, clone E3O7V, lot unknown

IF antibodies:
CD3: supplier Bio-Rad, catalog number MCA1477, clone CD3-12, lot unknown
CD206: supplier Cell Signaling Technology, catalog number 24595S, clone E6T5J, lot unknown
EGFRvIII: supplier ThermoFisher Scientific, catalog number MA5-36216, clone RM419, lot unknown
GFAP: supplier Cell Signaling Technology, catalog number 12389S, clone D1F4Q, lot unknown
IBA1: supplier Novus Biologicals, catalog number NB100-1028, clone polyclonal, lot unknown
Ki67: supplier ThermoFisher Scientific, catalog number 14-5698-82, clone SolA15, lot unknown
TMEM119: supplier Abcam, catalog number ab209064, clone 28-3, lot unknown
Donkey anti-Rabbit AF647: supplier Jackson ImmunoResearch, catalog number 711-605-152, clone polyclonal, lot unknown
Donkey anti-Rat AF555: supplier Invitrogen, catalog number A78945, clone polyclonal, lot unknown
Donkey anti-Goat AF488: supplier Jackson ImmunoResearch, catalog number 705-545-147, clone polyclonal, lot unknown

GAM modulation spectral FC antibodies:
AXL AF488: supplier R&D Systems, catalog number FAB8541G, clone 175128, lot AEWU0422121
CD4 BUV496: supplier BD Biosciences, catalog number 612952, clone GK1.5, lot 2272360
CD8a BV650: supplier BioLegend, catalog number 100742, clone 53-6.7, lot B368585
CD11b BV510: supplier BioLegend, catalog number 101245, clone M1/70, lot B376886
CD11c PE/Cy7: supplier BioLegend, catalog number 117317, clone N418, lot B346714
CD45 BUV737: supplier BD Biosciences, catalog number 748371, clone 30-F11, lot 23222187
CD86 PE/Cy5: supplier BioLegend, catalog number 105016, clone GL-1, lot B240898
CD206 AF700: supplier BioLegend, catalog number 141734, clone C068C2, lot B384250
EGFRvIII PerCP: supplier Novus Biologicals, catalog number NBP2-50599PCP, clone DH8.3, lot D139305
F4/80 BV605: supplier BioLegend, catalog number 123133, clone BM8, lot B386425
Ly6C BV785: supplier BioLegend, catalog number 128041, clone HK1.4, lot B371649
Ly6G BUV563: supplier BD Biosciences, catalog number 612921, clone 1A8, lot 2343097
MERTK BV711: supplier BioLegend, catalog number 151515, clone 2B10C42, lot B354635
MHC-II NF610-70S: supplier ThermoFisher Scientific, catalog number M024T02B06, clone M5/114.152, lot 2511116
P2RY12 APC/F810: supplier BioLegend, catalog number 848013, clone S16007D, lot B343968
Siglec-H APC: supplier BioLegend, catalog number 129612, clone 551, lot B286621
XCR1 BV421: supplier BioLegend, catalog number 148216, clone ZET, lot B377550
TNF PE: supplier BioLegend, catalog number 506305, clone MP6-XT22, lot B336284
Human TruStain FcX: supplier BioLegend, catalog number 422302, clone unknown, lot B369982
TruStain FcX: supplier BioLegend, catalog number 101320, clone 93, lot B380119

IF pharmacoscopy antibodies:

	CD3 AF488: supplier BioLegend, catalog number 300415, clone UCHT1, lot unknown<br>CD14 AF647: supplier BioLegend, catalog number 325612, clone HCD14, lot unknown<br>EGFRvIII: supplier ThermoFisher Scientific, catalog number MA5-36216, clone RM419, lot unknown<br>NESTIN: supplier BioLegend, catalog number 656802, clone 10C2, lot unknown<br>Goat anti-Mouse AF488: supplier Invitrogen, catalog number A32723TR, clone polyclonal, lot unknown<br>Goat anti-Rabbit AF647: supplier Invitrogen, catalog number A32733, clone polyclonal, lot unknown<br><br>CAR T cell and cetuximab combination spectral FC antibodies:<br>CD11b BUV395: supplier BD Biosciences, catalog number 563553, clone M1/70, lot 3346840<br>CD11c BUV496: supplier BD Biosciences, catalog number 750450, clone N418, lot 4079322<br>CD45 BUV737: supplier BD Biosciences, catalog number 748371, clone 30-F11, lot 2322187<br>CD80 BV605: supplier BioLegend, catalog number 104729, clone 16-10A1, lot B357122<br>CD86 PE/Cy5: supplier BioLegend, catalog number 105016, clone GL-1, lot B376879<br>CD163 APC/Cy7: supplier BioLegend, catalog number 155323, clone S15049I, lot B383053<br>CD206 AF700: supplier BioLegend, catalog number 141734, clone C068C2, lot B384250<br>CD274 PE/Cy7: supplier BioLegend, catalog number 153613, clone MIH6, lot unknown<br>CD49d BUV563: supplier BD Biosciences, catalog number 741243, clone 9C10(MFR4.B), lot 3200542<br>Ly6C BV785: supplier BioLegend, catalog number 128041, clone HK1.4, lot B386418<br>Ly6G BUV661: supplier BD Biosciences, catalog number 741587, clone 1A8, lot 4012615<br>MHC-II NF610-70S: supplier ThermoFisher Scientific, catalog number M024T02B06, clone M5/114.152, lot 2925490<br>P2RY12 APC/F810: supplier BioLegend, catalog number 848013, clone S16007D, lot B39443<br>Siglec-1 FITC: supplier BioLegend, catalog number 142405, clone 3D6.112, lot B388729<br>XCR1 APC: supplier BioLegend, catalog number 148205, clone ZET, lot B349249<br>Human TruStain FcX: supplier BioLegend, catalog number 422302, clone unknown, lot B369982<br>TruStain FcX: supplier BioLegend, catalog number 101320, clone 93, lot B380119
Validation	All antibodies used in this study were obtained from reputable commercial suppliers and were selected based on their documented validation by the vendors. The validation process for these antibodies typically included rigorous testing for specificity, sensitivity, and reproducibility. The antibodies were also cross-referenced with literature to confirm their suitability for our experimental applications. A full list of antibodies and dyes used in the study is provided in Supplementary Table 4.

Eukaryotic cell lines

Policy information about cell lines and Sex and Gender in Research

Cell line source(s)	BS153 tumor cell line: GBM cells derived from a patient of unknown sex and age. Source: University of Basel. Gifted by Prof. Alfred Zippelius, University of Basel.<br>HEK293T embryonic cell line: Kidney cells derived from a female fetus. Source: ATCC. Gifted by Prof. Mohamed Bentires-Alj, University of Basel.<br>NSC197 stem cell line: Neural stem cells derived from a subject of unknown sex and age. Source: McMaster University. Gifted by Prof. Sheila Singh, McMaster University.<br>Raji tumor cell line: Burkitt's lymphoma cells derived from an 11-year-old male patient. Source: ATCC. Provided by Prof. Heinz Läubli, University of Basel.<br>U87 tumor cell line: GBM cells derived from a 44-year-old female patient. Source: ATCC. Provided by Prof. Luigi Mariani, University Hospital Basel.<br>U87vIII tumor cell line: U87 cell line specifically engineered to express the EGFRvIII mutation found in GBM. Gifted by Prof. Sebastian Kobold, Ludwig Maximilian University of Munich.<br>U251 tumor cell line: Astrocytoma cells derived from a 75-year-old male patient. Source: ATCC. Gifted by Prof. Sebastian Kobold, Ludwig Maximilian University of Munich.<br>U251vIII tumor cell line: U251 cell line specifically engineered to express the EGFRvIII mutation found in GBM. Gifted by Prof. Sebastian Kobold, Ludwig Maximilian University of Munich.
Authentication	Cell lines were not genetically profiled for this study, and were already in stock at our institution or gifted by collaborators. Cell surface expression of CD19, CD47, EGFR, and EGFRvIII was verified by FC. Transcriptomic EGFR and EGFRvIII expression levels were determined by RT-qPCR. Growth rates and morphology were assessed directly in vitro. In vivo engraftment rates were tested prior to large-scale experiments.
Mycoplasma contamination	Cell cultures were routinely tested for mycoplasma contamination using a MycoAlert PLUS Mycoplasma Detection kit (#LT07-710, Lonza, Switzerland).
Commonly misidentified lines<br>(See ICLAC register)	No misidentified cell lines were used in the study.

Animals and other research organisms

Policy information about studies involving animals; ARRIVE guidelines recommended for reporting animal research, and Sex and Gender in Research

Laboratory animals	NOD.Cg-Prkdcscid Il2rg/SzJ (NSG) mice with identifier RRID:IMSR_JAX:005557 were obtained from in-house breedings or externally from Janvier Labs, France, under protocols approved by the Swiss Federal Veterinary Office (SFVO) and Cantonal Veterinary Office

	(CVO) of Basel-Stadt. All experiments involving GBM models were performed on NSG mice of the male sex, aged 7-12 weeks at the time of tumor implantation and all experiments involving lymphoma models were performed on NSG mice of the female sex, aged 8-12 weeks
Wild animals	N/A.
Reporting on sex	For logistic reasons, only male NSG mice were used in the GBM experiments and only female mice were used in the lymphoma experiments. CAR T cells were sourced from anonymous healthy donors of unknown sex. We did not investigate sex-specific differences in outcomes of the CAR T cell treatment and tumor engraftment studies.
Field-collected samples	N/A.
Ethics oversight	Animal handling, surveillance, and experimentation were performed according to the SFVO guidelines and the CVO of Basel-Stadt. GBM model experiments were executed under licenses #2929_31795 and #3176_35274, and lymphoma model experiments under license #3036_34231.

Note that full information on the approval of the study protocol must also be provided in the manuscript.

Clinical data

Policy information about clinical studies
All manuscripts should comply with the ICMJEguidelines for publication of clinical research and a completedCONSORT checklist must be included with all submissions.

Clinical trial registration	N/A.
Study protocol	N/A.
Data collection	N/A.
Outcomes	N/A.

Plants

Seed stocks	N/A.
Novel plant genotypes	N/A.
Authentication	N/A.

Flow Cytometry

Plots

Confirm that:

- [x] The axis labels state the marker and fluorochrome used (e.g. CD4-FITC).
- [x] The axis scales are clearly visible. Include numbers along axes only for bottom left plot of group (a 'group' is an analysis of identical markers).
- [ ] All plots are contour plots with outliers or pseudocolor plots.
- [x] A numerical value for number of cells or percentage (with statistics) is provided.

Methodology

Sample preparation	Preparation of CAR T cells for sorting:<br>Peripheral blood mononuclear cells (PBMCs) were isolated with Ficoll Paque-PLUS (#GE17-1440-02, Cytiva, Germany) and density centrifugation. After up to 2 rounds of ACK-lysis (#A10492-01, Gibco, USA) to remove erythrocytes, PBMCs were washed with PBS. CD3+ T cells were magnetically separated by negative selection with a Human Pan T cell isolation kit (#130-096-535, Miltenyi Biotec, Germany) and stored long-term in Bambanker serum-free cell freezing medium (#BB01, GC Lymphotec, Japan) in liquid nitrogen (LN2). CD14+ cells used in phagocytosis assays were magnetically separated by positive selection using Human CD14 MicroBeads (#130-050-201, Miltenyi Biotec, Germany) and stored in Bambanker serum-free cell freezing medium in LN2. CAR T cells were produced from HD PBMCs, depleted for non-T cells, and frozen in batches. CAR T cells were freshly made from frozen HD T cell batches for every experiment. Upon thawing, T cells were washed with PBS and rested in X-VIVO 15 (#BE02-060F, Lonza, Switzerland) at a density of 1×10^6cells per mL at 37°C in a 5% CO2 atmosphere. After 24 h, the T cells were activated in X-VIVO 15 containing 150 U/mL of human IL-2 (#Ro 23-6019, Roche, Switzerland), 10

ng/mL of recombinant IL-7 (#200-07, PeproTech, USA), 10 ng/mL of recombinant IL-15 (#200-15, PeproTech, USA), 20 ng/mL of recombinant IL-21 (#200-21, PeproTech, USA) and Dynabeads human T-activator CD3/CD28 (aCD3/CD28; #11131D, Gibco, USA) in a 1:1 cell-bead ratio3. After 48h, aCD3/CD28 beads were magnetically removed, and T cells were resuspended in X-VIVO 15 with 5 µg/mL polybrene (#TR-1003-G, Sigma-Aldrich, USA) at a density of 3×10^6cells per mL. Lentiviral suspensions were added to the T cells at different multiplicity of infection (MOI) ratios and spinfected at 1200 × g, at 30°C for 90 min. After spinfection, the T cells were washed and maintained at a density of 1×10^6cells per mL in X-VIVO 15 containing 500 U/mL IL-2 for 5-7 days. After this post-transduction cell expansion, T cells were sorted for mCherry expression using a BD FACSMelody Cell Sorter (BD Biosciences, USA).

Preparation of in vitro CAR-biotinylated target protein samples:
CAR T cell viability and count were assessed by Trypan blue exclusion. Cells were washed with PBS and seeded into a 96-well plate at a density of 2 ×10^5 live cells per well. Cells were immediately stained with a Zombie NIR Viability kit (#423106, BioLegend, USA) diluted 1:5000 in PBS for 20 min in the dark at RT. After viability staining, the cells were washed with autoMACS Running Buffer (#130-091-221, Miltenyi Biotec, Germany) and then resuspended in 100 µL per well of 10 µg/mL dilutions of biotinylated CD19, EGFR, or EGFRvIII proteins (#CD9-H82E9, #EGR-H82E3, and #EGR-H82E0, ACROBiosystems, USA) for 1 h in the dark at 4°C. After CAR-target exposure, the cells were washed with autoMACS Running Buffer and stained with 100 µL per well of FITC Streptavidin (SA; #405202, BioLegend, USA) diluted 1:50 in autoMACS Running Buffer for 1 h in the dark at 4°C. Afterward, the cells were washed three times with autoMACS Running Buffer and resuspended in 100 µL of autoMACS Running Buffer. Samples were acquired with a CytoFLEX Flow Cytometer (Beckman Coulter, USA), and data were analyzed using FlowJo v10 Software (BD Biosciences, USA).

Preparation of samples for in vitro cell surface marker expression analysis:
The expression of cell surface markers was determined by FC. Briefly, single-cell suspensions (SCS) were counted by Trypan blue exclusion and seeded in 96-well plates at a density of 2 × 10^5 cells per well. Cells were washed with PBS and resuspended in 100 µL of antibody staining solution. Depending on the experiment, a viability staining step was performed either before antibody staining, using Zombie NIR Viability kit (#423105, BioLegend, USA) or after, using BD Pharmingen DAPI Solution (#564907, BD Biosciences, USA) or DRAQ7 (#424001, BioLegend, USA).

Preparation of samples for in vitro CAR T cell degranulation assay:
T cell degranulation in co-cultures with GBM cells was assessed by FC. Briefly, tumor cells were washed with PBS, dissociated, and counted by Trypan blue exclusion. GBM cells were seeded in a flat-bottom 96-well plate at a density of 1 × 10^4 cells per well in 100 µL of growth medium and incubated for 24 h to form a cell monolayer. Afterward, media in the wells was discarded, replaced by 100 µL of CAR T cell or mock-transduced T cell suspensions in GBM growth medium in 1:1 E:T, and incubated for 24 h. At 24 h of co-culture, suspension cells were gently mixed in the supernatant and collected into a round-bottom 96-well plate. Cells were washed with PBS and stained with a BB700-conjugated anti-LAMP1 (clone H4A3; #566558, BD Biosciences, USA) for 20 min in the dark at 4°C. After surface staining, the cells were washed 3 times with autoMACS Running Buffer and resuspended in 100 µL per well of 0.5X DAPI diluted in autoMACS Running Buffer. Samples were acquired in a CytoFLEX Flow Cytometer.

Preparation of samples for in vitro on-cell blocking assay:
BS153 viability and count were assessed by Trypan blue exclusion, after which cells were seeded in flat-bottom 96-well plates at a density of 3 × 10^5 cells per well. Cells were then treated for 30 min at 4°C with 50 µL per well of 10 µg/mL of InVivoMAb anti-human CD47 (clone B6.H12; #BE0019-1, Bio X Cell, USA) or InVivoMAb mouse IgG1 isotype control (clone MOPC-21; #BE0083, Bio X Cell, USA) or conditioned-media from 24 h-rested, antigen-naïve aEGFRvIII or aEGFRvIII-SGRP CAR T cells seeded at a density of 1 × 10^6 cells per mL of unsupplemented RPMI. Pre-treated tumor cells were washed with PBS and incubated for 30 min at 4°C with biotinylated SIRPα (bt-SIRPα; #CDA-H82F2, ACROBiosystems, USA), which bound to the unblocked CD47 on BS153 cells. Finally, APC Streptavidin (SA; #405207, BioLegend, USA) staining was performed, followed by three washes with autoMACS Running Buffer and resuspension in 100 µL per well of 0.5X DAPI in autoMACS Running Buffer. Samples were acquired in a CytoFLEX Flow Cytometer.

Preparation of samples for in vitro phagocytosis assay:
Primary monocytes were thawed, washed in PBS and plated at 0.8 x 10^6 cells per mL in RPMI 1640 with 1X GlutaMAX (#61870-036, Gibco, USA), 10% FBS (#P30-3302, PAN-Biotech, Germany), 5% human serum (#H4522, Sigma-Aldrich, USA) and 25 ng/mL M-CSF (#300-25, PeproTech, USA), and incubated at 37°C in a 5% CO2 atmosphere. After 48 h, the medium was replaced with human serum-free growth media and refreshed every 2-3 days for a maximum of 14 days until phagocytosis assays. Macrophages were detached from culture dishes using cell scrapers after a 15-minute incubation at 37°C in TrypLE Express (#12604-021, Gibco, USA). Cells were washed in PBS, counted, seeded at 5 x 10^4 cells per well in a 96-well flat-bottom plate (#353072, Corning, USA), and incubated for 48 h to allow attachment. CAR T cells were counted and diluted to 1 x 10^6 cells per mL in X VIVO 15. Tumor cells were washed in PBS, dissociated, and counted. All cell counts were performed by Trypan Blue exclusion. U251vIII cells were diluted to 1 x 10^6 cells per mL in IMDM. U87 cells were stained with 62.5 x 10 nM CellTracker Green (#C2925, Thermo Scientific, USA) diluted in IMDM (#12440-053, Gibco, USA) for 30 min at 37°C at a density of 1 x 10^6 cells per mL. After two washes with PBS, stained cells were counted and adjusted to 1 x 10^6 cells per mL in IMDM. U87 and U251vIII cells were then mixed in a 1:1 ratio. The tumor cell mixture (1 x 10^5 cells) and CAR T cells (5 x 10^4 cells) were added to each well containing macrophages. The contents were resuspended in IMDM and incubated at 37 °C for 3 h. After incubation, cells were detached using TrypLE, transferred to a 96-well U-bottom plate, and washed twice in PBS. Viability staining was carried out by incubating cells with a Zombie UV Viability kit (#423107, BioLegend, USA) for 20 min at 4°C in the dark. Fc-block was performed by incubating cells in a dilution of Human TruStain FcX (#101320, BioLegend, USA) for 10 min at 4°C. Antibody mastermixes (full-stains and FMOs) were prepared freshly in autoMACS Running Buffer. Cells were stained with surface marker antibody mastermixes for 25 min at 4°C in the dark and washed twice in autoMACS Running Buffer. Cells were fixed by incubation for 20 min at RT using a Cyto-Fast Fix/Perm Buffer set (#426803, BioLegend, USA). After antibody staining, samples were washed twice with autoMACS Running Buffer, resuspended in 200 µL MACS, and acquired on a Cytek Aurora 5-Laser Spectral Analyzer (Cytek Biosciences, USA), using standard, daily quality-controlled, Cytek-Assay-Settings.

Preparation of mouse brain tissue samples:
Animals were euthanized by CO2 suffocation, and brain regions were immediately harvested into ice-cold HBSS. Depending on the experiment, the tumor-injected hemisphere, contralateral hemisphere, or brain meninges were carefully dissected and manually minced using razor blades and enzymatically dissociated at 37°C for 30 min with 1 mg/mL collagenase type IV (#LS004188, Worthington Biochemical Corporation, USA) and 250 U/mL DNase 1 (#10104159001, Roche, Switzerland) in a buffer containing HBSS with Ca2+/Mg2+ (#14205-050, Gibco, USA), 1% MEM NEAA, 1 mM sodium pyruvate, 44 mM sodium bicarbonate (#25080-060, Gibco, USA), 25 mM HEPES (#H0887, Gibco, USA), 1% GlutaMAX-I and 1% antibiotic-antimycotic (#15240062, Gibco, USA). The resulting cell suspensions were filtered through a 70 µm strainer and centrifuged in a density gradient using debris removal solution (#130-109-398, Miltenyi Biotec, Germany) according to the manufacturer's protocol to remove myelin and cell debris. Erythrocytes were removed using ACK lysing solution (#A1049201, ThermoFisher Scientific, USA), and cell suspensions were washed with PBS and kept on ice until spectral flow staining. Freshly dissociated cells were washed with PBS and resuspended in 400 µL of PBS. A 200 µL fraction of one sample per treatment group was used as an unstained control to detect and correct for condition-specific autofluorescence. In all cases, the input volume for full-stained samples was kept at 200 µL to ensure equivalent staining conditions across all samples. The remaining 200 µL of SCS volume was mixed and used to generate fluorescence minus one (FMO) stainings, allowing for condition-specific FMO gating. All centrifugation and incubation steps were performed at 300 × g at 4°C, protected from light, unless otherwise stated. Viability staining was performed by incubating cells with a Zombie Aqua or NIR Viability kit for 20 min. Subsequently, Fc-block was performed by incubating cells in a dilution of human TruStain FcX (#422302, BioLegend, USA) and mouse TruStain FcX (#101320, BioLegend, USA) for 10 min. Antibody mastermixes (full-stains and FMOs) were freshly prepared on the day of staining in Brilliant Stain Buffer (#00-4409-75, ThermoFisher Scientific, USA). Cell surface markers were stained with surface antibody mastermixes for 25 min and then washed twice in autoMACS Running Buffer. To allow the subsequent staining of intracellular antigens, a fixation/permeabilization step was performed by incubating the cells for 20 min at RT using a Cyto-Fast Fix/Perm Buffer set (#426803, BioLegend, USA). Intracellular antibody staining was then performed for 25 min at RT. Finally, the samples were washed twice, resuspended in a final volume of 200 µL of PBS, and acquired on a Cytek Aurora 5-Laser Spectral Analyzer using standard daily quality-controlled Cytek-Assay-Settings.

Instrument

Conventional FC: CytoFLEX Flow Cytometer (Beckman Coulter, USA)
FC sorting: BD FACSMelody Cell Sorter (BD Biosciences, USA), BD FACSAria SORP Cell Sorter (BD Biosciences, USA)
Spectral FC: Cytek Aurora 5-Laser Spectral Analyzer (Cytek Biosciences, USA)

Software

Conventional FC: Cytexpert v2.6 (Beckman Coulter, USA), FlowJo v10 (BD Biosciences, USA), GraphPad Prism v10 (GraphPad, USA)
FC sorting: BD FACSChorus v3.0 (BD Biosciences, USA), BD FACSDiva v6.0 (BD Biosciences, USA)
Spectral FC: Spectroflo v3.3 (Cytek Biosciences, USA), FlowJo v10 (BD Biosciences, USA), GraphPad Prism v10 (GraphPad, USA), R v4.3.1 and packages FlowVS, FlowSOM, PeacoQC, ConsensusClusterPlus, CATALYS, diffcyt

Cell population abundance

The purity of mCherry+ CAR T cells and subset proportions after transduction and expansion is shown in Supplementary Figure 1d. The purity of mCherry+ CAR T cells and subset proportions after sorting is shown in Supplementary Figure 1e.

Gating strategy

The gating strategy of the FC analysis of Figure 6 is shown in Supplementary Figure 8. The gating strategy of the FC analyses of Supplementary Figure 10 are shown within that Supplementary Figure. All other FC analyses in our study followed a simple gating strategy: debris removal, single cells, and live cells.

☒ Tick this box to confirm that a figure exemplifying the gating strategy is provided in the Supplementary Information.
