# Supplementary material for: Enhancing anti-EGFRvIII CAR T cell therapy against glioblastoma with a paracrine SIRPγ-derived CD47 blocker
Source: Nat Commun. 2024 Nov 9;15:9718. doi: 10.1038/s41467-024-54129-w (PMC11550474; doi:10.1038/s41467-024-54129-w)
Supplement: Supplementary file 16 — Transparent Peer Review file [file 41467_2024_54129_MOESM16_ESM.pdf]

Peer Review File

Enhancing anti-EGFRvIII CAR T cell therapy against glioblastoma with a paracrine SIRPγ-derived CD47 blocker

Corresponding Author: Dr Tomás Martins

Parts of this Peer Review File have been redacted as indicated to remove third-party material.

This file contains all reviewer reports in order by version, followed by all author rebuttals in order by version.

Version 0:

Reviewer comments:

Reviewer #1

(Remarks to the Author)
Glioblastoma (GBM) is an extremely aggressive brain tumour with a dire prognosis, as current treatments like surgery and chemotherapy offer only a temporary reprieve, leading to a median survival time of just 15 months. Although Chimeric Antigen Receptor (CAR) T cell therapies have been effective in blood cancers, their efficacy against solid tumours like GBM is limited due to issues like antigen variability and immune suppression within the tumour environment. Recent trials targeting the GBM-specific antigen EGFRvIII with CAR T cells demonstrated safety but not lasting effectiveness. Furthermore, while immune checkpoint therapies show promise, the immunosuppressive nature of GBM's microenvironment hinders their success. A novel approach combining intratumoural CAR T cell therapy with modulation of the tumour's immune environment—specifically targeting the myeloid cells that often support tumour growth—has been proposed. This strategy, leveraging both direct CAR T cell targeting and manipulation of the tumour's own defenses, could potentially lead to complete clearance of GBM and represents a significant step forward in the treatment of this and potentially other solid tumours.

Authors conducted large scale animal models of a heterogeneous mosaic model of GBM, implanted orthotopically, and show superior efficacy of the EGFRvIII-SGRP secreting CART cells. The authors demonstrate their SGRP secreted vIII-specific CAR leads to increased myeloid cell mediated phagocytosis of GBM. The authors also demonstrate utility of the SGRP CART cells in a mouse model of peripheral lymphoma, highlighting the broader application beyond GBM.

Overall the paper presented is very comprehensive, well written and the figures are presented nicely. The results are noteworthy and significant. I list three small suggestions below that would enhance it further.

1) Authors demonstrate CAR specificity and CAR T cell in vitro cytotoxicity, 24 hour degranulation (LAMP1 exposure) and IFNg release against GBM cell lines. Interestingly, they generate CD4+ CART cells almost exclusively. It would be beneficial for the authors to more specifically address their proposed mechanism of action given CD8+ T cells are not present, and they have demonstrated CD4 CART cells are degranulating (ie. Discussion is worded such that the may be ruling out perforin/gnz mediated death in favour of indirect killing via IFNg/TNF. In any case it isn't clear).

2) Figure 2. The Y-Axis in figure2 is slightly problematic (ie. Fig 2a-c starts at 5e4, then goes to 2.5e5, then to 4.5e5 etc). This axis does not follow either a linear OR logarithmic progression. This is not standard practice as it can lead to misinterpretation of the information presented. I strongly suggest the authors regraph using either a linear or Log scale, to present the range of values. Similarly, the graph in Fig2g, jumps from0, to 5e3, 1e4, then to 1.5e4. This is misleading.

3) T cells also express CD47, and in fact CD47 expression is critical for CART cell survival in vivo (PMID: 36918226 & should be cited). In the 'on-cell CD47 blocking assay' how did the authors control for CD47-mediated fratricide between the T cells? It would be beneficial to show the level of CD47 expression on the T cells. The authors correctly hypothesise that SGRP may be partially captured by CAR T cells. Given CD47 expression on T cells, it is intriguing that the delivery of anti-CD47 antibody in animal models (Fig 3) in combination with CART cells, does not deplete the human CAR T cells from the animal, and therefore render the EGFRvIII therapy ineffective. The authors should show the levels of CART cells in the blood to demonstrate engraftment of the therapy, and to show that the added survival benefit of antiCD47 blockade is CART

cell mediated.

Reviewer #2

(Remarks to the Author)
This work addresses a clinical important issue such as the immunosuppressive tumor microenvironment impeding immunotherapy, including CAR-t approaches. The authors propose the way to overcome those obstacles with a novel CAR-T cell approach by combining targeting tumor associated myeloid cells with CAR-T. They engineered a CAR T cell against epidermal growth factor receptor variant III (EGFRvIII) expressing cells that constitutively secrete a signal regulatory protein gamma (SIRPγ)-related protein (SGRP) with high affinity to CD47. Blockade of CD47 augmented phagocytic function of glioma associated microglia and macrophages in GBM-bearing mice and elicited antitumoral responses in vivo, but due to the ubiquitous expression of CD47, the therapy had side effects. Anti-EGFRvIII-SGRP CAR T cells eradicated orthotopic EGFRvIII-mosaic glioblastoma (GBM) xenografts in vivo and promoted GAM-mediated tumor cell phagocytosis. EGFRvIII is expressed in approximately 40% of GBMs. In another tumor model mouse CD19+ lymphoma, anti-CD19-SGRP CAR T cell therapy was superior to conventional anti-CD19 CAR T. Tumor rechallenge of aEGFRvIII-SGRP CAR-cured mice resulted in prolonged survival. Altogether, data convincingly show that combining a specific modulation of GAMs with anti-EGFRvIII targeted CAR T cells produces the sustainable anti-glioma effects.
The results are noteworthy and of significance to the field of neurooncology and in general, cancer immunotherapy.

The methodology sound and the work meets the expected standards.
Technically, this is an elegant and comprehensive work carefully presenting activity and specificity of the created anti-EGFRvIII-SGRP CAR T cells. The results were collected with the impressive scope of methods. Important aspects of characterization of EGFRvIII-SGRP CAR T cells were considered and necessary controls were applied, including testing of targets of interest on multiple cell lines: 4 GBM cell lines (U251, U87, U251vIII with transgenic overexpression EGFRvIII, BS153 with endogenous EGFRvIII expression), Raji Burkitt's lymphoma cell line and neural stem cell (NSC) line (NSC197). aCD19 CAR and aCD19-SGRP CAR were used as controls in GBM experiments and Raji as CD19+ 112 target cells. It should be appreciated that the authors performed experiments with numerous and appropriate controls: the production of SGRP was confirmed by proteomics; in vitro efficacy of EGFRvIII-SGRP CAR was tested against 2 glioma cell lines; activity of SGRP from CAR T was compared to anti-CD47 antibody, etc. Some minor drawbacks were identified but those can be remedied by a proper revision and the better explanation of the author's viewpoint.
Comments:
1. The combination of aEGFRvIII CAR + aCD47 failed to improve survival compared to aEGFRvIII CAR monotherapy (20%), while aEGFRvIII-SGRP CAR was extraordinarily potent , with 94.7% overall and 63.2% tumor-free survival. This result should be better explained.
2. In the description of the Fig.3 they write :"that a majority of EGFRvIII- tumors were eradicated after aEGFRvIII-SGRP CAR treatment". Why it so? This was EGFRvIII-mosaic GBM mouse model and bystander effects on aEGFRvIII- cells can hardly explain such results. Please comment on this issue.
3. The statement " a potential persistence of CAR T cells and/or antitumor GAMs in the brains of these animals" is not supported by any evidence.
4. Targeted proteomic analysis of plasma samples was performed to identify proteins differentially expressed between groups and revealed some hints. The analysis was done on 2 samples and such important finding should be verified with quantitative method (ELISA) on more samples.
5. I am not fully convinced about the use of the EGFRvIII-mosaic GBM mouse model obtained by mixing two glioma cell lines with EGFRvIII positive and negative cells, respectively. They implanted U251 and U87 cells which means they implanted two completely different tumors into one host. I understand that the cells should be distinct but these tumors are entirely different so their responses to therapy could be related to their genetic background not to a lack of EGFRvIII. Relevance this model to human pathology is unclear.
6. The authors state that animals were treated with therapy twice – at 7 and 14 days – after intracranial tumor implantation using the same stereotactic coordinates. It is not simple to deliver intratumoral injection into the same place. I wonder if this was monitored somehow.
7. They found only tumor remnants in aEGFRvIII-SGRP CAR treated group and did not the immune TME in this condition. Actually, despite small tumor sizes that adjacent area could contain many immune cells with interesting phenotypes and the analysis could reveal interesting observation. From my expertise even is tumor is residual the immune infiltrate could be the same as in large tumor, only cells have different phenotypes. Those samples should be analyzed as well and in fact, they are the most interesting group.
8. TSNE plots for aEGFRvIII-CAR and aEGFRvIII-SGRP CAR in the Fig.6f look the same (correct TNSE on Y axis). One would expect differences in 8 presented subpopulations between groups considering data presented on reprograming of GAMs.
9. I do appreciate showing the higher efficacy of aCD19-SGRP CAR in another tumor model (Raji lymphoma cells). But the number of animals are small n=5-6 for most interesting groups and n=4 for other groups is not sufficient. Extended fig.4 b shows that BLI measurements of tumors and in the control group 2 out of 10 mice do not show any BLI signal.
10. Extended fig.4 b shows bioluminescence (BLi) data indicating that EGFRvIII+ and EGFRvIII- tumor cells were equally eradicated with aEGFRvIII-SGRP CAR (only 1 out of 10 EGFRvIII- showed slight BLi). The authors attribute high effectiveness of EGFRvIII+ and EGFRvIII- tumor cells were equally eradicated with aEGFRvIII-SGRP CAR against EGFRvIII-tumors to the bystander effect but than it would mean the lack of selectivity in the treatment. In aEGFRvIII CAR + aCD47 treated group the BLi signal was visible in 9 out of 10 10 EGFRvIII-tumors indicting a high selectivity of the treatment and the bystander effects on viability was not evident.

11. Extended fig. 6 shows CD3 staining in brains with tumors: the IHC images show differences between treatment groups but there is no statistical differences in the CD3 abundance. The choice of IHC staining images was not fortunate as those data do not correspond (more CD3 staining in EGFRvIII-CAR and EGFRvIII-CAR+aCD47 groups. Why in aEGFRvIII-SGRP CAR group the staining is shown at day 13 while in other groups at day 21?
12. The CD47 treatment leads to systemic toxicity. The authors state "off-tumor toxicity …was clinically absent in our mouse model" as they delivered the treatment intratumorally. However, some observed effects on myeloid populations could be explained SGRP inflammation and there is no data on local toxicity.

Minor comments:
Abstract: Correct the sentence: "CAR T cell against epidermal growth factor receptor variant III (EGFRvIII) that constitutively secretes a signal regulatory protein gamma (SIRPγ)-related protein (SGRP) with high affinity to 39 CD47". The authors meant EGFRvIII cells expressing….Correct: "GAM-mediated tumor cell uptake". In case of cells it is phagocytosis. In biology "an uptake" has very a specific meaning and refers to transport of ions or compounds.
This sentence is not clear: "Conversely, aEGFRvIII CAR treatment (median survival: 65.5 d) led to 40% survival 90 d after tumor implantation, starkly contrasting with aCD19 CAR control (median survival: 29 d)". Why 90 days if mice do not survive that long?

Reviewer #3

(Remarks to the Author)
This manuscript describes the construction of CAR-T cells with secretive CD47 blockers to achieve inhibition of brain tumors. I am concerned with the overall novelty of the study, which is modest at best and the selection of animal models used in the study. In addition, there are many major experiments missing that need to be done to support the conclusions of the paper. Please see my specific comments as follows:

1. The novelty of the study is a particular concern. It has been reported that CAR-T with secretive SIRPa can block the CD47 pathway and enhance the therapy efficacy (PMID: 35110357). Although the current work targets a different kind of tumor, the concept is not novel and is merely an extension of prior work.
2. This work used SIRPg to bind with CD47, instead of the commonly used SIRPa. Is there any rationale to choose SIRPg? Does SIPRa and SIRPg blockades have similar effects? Or SIRPg works better?
3. Experimental data is missing to characterize the secreted SIRPg. For example, the amount of protein secreted, especially the concentration of the protein in vitro and in vivo, to determine if this concentration is enough to make any therapeutic difference.
4. In the GBM CAR-T trial (NCT01454596), the CAR-T were infused to patient. However, in the present study, CAR-T were intratumorally injected, which is expected to have better efficacy. Can the SGRP CAR achieve any therapeutic effect against brain tumor if injected intravenously? Also, intravenous injection is expected to have more toxicity since SIRPg will be secreted to the circulation and block the CD47 on RBC. Therefore, toxicity study should also be carried out in the more clinical-relevant intravenous injection model.
5. The brain tumor model used tumor: T cell ratio of 1:10, which is very high. How does this ratio compare to other brain CAR-T models and those trials used clinically?
6. Figure 2 showed no SGRP-mediated benefit in vitro. How about adding macrophages to the in vitro co-culture model to test if there's any benefit?
7. Some work is needed to justify the model selection. This work uses the mixture of two different cell lines to establish the model. One cell line is EGFRvIII + and one is -. It is possible that the two cell lines respond differently to CAR-T therapy or their growth characteristics are different. Therefore the observed phenotype may due to their innate nature, not EGFRvIII status. I recommend use the same cell line with or without EGFRvIII expression and label them with different luciferase to monitor the treatment outcome.
8. Figure 3f showed that majority of EGFRvIII- tumors were eradicated after aEGFRvIII-SGRP CAR treatment. But what's the mechanism? When treating EGFRvIII- tumors, the aEGFRvIII-SGRP CAR is expected to work similar to aCD19-SGRP CAR, since in both cases, there's no target for the CAR. Then why aEGFRvIII-SGRP CAR can inhibit EGFRvIII- but not aCD19-SGRP CAR? I recommend just inject the EGFRvIII- tumors (don't use the mixture) and test if these two CARs can inhibit the tumor growth.

Reviewer #4

(Remarks to the Author)
I would like to commend the authors on these well designed experiments exploring the potential benefit of blocking the CD47/SIRPy pathway as a way to mitigate against GBM antigen heterogeneity. The authors did a good job at dissecting the TME and effects of blocking CD47 mediated inhibition of phagocytosis on GAMs.

However, the paper does not address the true benefit of engaging the GAM compartment of GBMs in vivo by blocking CD47 using secreted SIRPy. What is missing is experiments both in vivo and in vitro addressing the benefit of having CAR T cells secreting SIRPy in trans in bolstering GAM phagocytic activity.

The paper would be improved by evaluating if the secreted SIRPy enhances phagocytosis in vitro using conditioned media from the CAR T cells secreting SIRPy and the addition of a opsonizing antibody to a GBM associated antigen. Moreover, in

vivo this could be addressed with a smaller experiment similar to the one outlined in Figure 3 where a mosaic GBM is dual targeted with CAR T cells secreting SIRPγ but complemented by the addition of opsonizing antibody that targets a tumor associated antigen found on antigen negative cells. These experiments will strengthen the paper as it goes gets to the heart of the project, can CAR T cells secreting SIRPγ lower the threshold for GAM mediated phagocytosis and engage them in mediating anti-tumor activity. Perhaps CD19 CAR P2A SIRPγ T cells complimented in trans with an EGFRvIII opsonizing antibody delivered IP at regular intervals. This would underscore the contribution of the GAM compartment being engaged to phagocytose tumor cells.

Minor issues:
Figure 3 results and figure legends: include the CAR T cell dose in the texts for readers to follow along.

Figure 4: CCL3 was identified to be enriched in plasma of mice treated with EGFRvIII CAR + SIRPγ T cells. Would blocking this GAM chemoattractant with CCL3 antibodies reduce the anti-tumor effect of SIRPγ secreting CAR T cells? This paper is filled with hypothesis generating data but this would test some of your observations.

Figure 5: Given the interspecies nature of the immune cells involved in these experiments, it would help to designate the species of the marker being examined. For example murine CD206, human CD45, etc. Is it not clear to the reader if the markers outlined are human or mouse.

Version 1:

Reviewer comments:

Reviewer #1

(Remarks to the Author)
The authors have performed substantial new experiments and I appreciate the thoughtful responses to the reviews.

Authors have adequately addressed all of my concerns and I am very comfortable recommending publication.

Reviewer #2

(Remarks to the Author)
The revised version is considerably improved with all additional experiments (including cytotoxicity experiments) and the detailed explanation of the author’s interpretation. Overall the study presented is very comprehensive, original and well written. It is not extension of previous studies but a novel approach to improve CAT cells efficacy in GBM, where up to now most immunotherapies failed. The authors addressed thoroughly all comments of this reviewer (and others as well) and provided additional data supporting their interpretation. Altogether, the findings presented in the revised are convincing and dissolve some of the uncertainties regarding a design and execution some of the experiments. The authors were no evasive and justified their conclusions with proper experiments.

Minor comments:
I do agree with the authors comments regarding the use of a mosaic tumor model, and I accept the model usefulness for some mechanistic studies. However, some of the explanation about a tumor heterogeneity is not correct. While it is true that the presence of different clones with distinct genomic/transcriptomic profiles in GBM results in high genomic and phenotypic intratumoral heterogeneity, these are clones from the same tumor lineage with changes arising during tumor clonal evolution. In the model applied in the study tumor cells are entirely different and their intrinsic setting might contribute to various responses to therapy. I do agree that the model, albeit reductionistic, to some extent is useful to resolve the questions about therapy efficacy of new CART cells. It would be worthy to mention the model limitations.
In the Introduction they used an abbreviation MoMacs for monocyte-derived macrophages. In the field MDM is frequently used for monocyte-derived macrophages, so it would better to sue the same abbreviation to describe the subpopulation.

Reviewer #3

(Remarks to the Author)
The authors have spent a significant amount of effort addressing many of the suggestions from the reviewers. Overall, the revised manuscript demonstrates technical innovation with a potential impact on glioblastoma, which may compensate for the modest conceptual novelty.

Reviewer #4

(Remarks to the Author)
I think the authors have done a very nice job responding to the queries and the paper looks great.

Open Access This Peer Review File is licensed under a Creative Commons Attribution 4.0 International License, which permits use, sharing, adaptation, distribution and reproduction in any medium or format, as long as you give appropriate credit to the original author(s) and the source, provide a link to the Creative Commons license, and indicate if changes were made.
In cases where reviewers are anonymous, credit should be given to 'Anonymous Referee' and the source.
The images or other third party material in this Peer Review File are included in the article's Creative Commons license, unless indicated otherwise in a credit line to the material. If material is not included in the article's Creative Commons license and your intended use is not permitted by statutory regulation or exceeds the permitted use, you will need to obtain permission directly from the copyright holder.
To view a copy of this license, visit https://creativecommons.org/licenses/by/4.0/

Point-by-point Response to the Reviewers

Reviewer #1 (Remarks to the Author): with expertise in CAR-T, glioblastoma

Glioblastoma (GBM) is an extremely aggressive brain tumour with a dire prognosis, as current treatments like surgery and chemotherapy offer only a temporary reprieve, leading to a median survival time of just 15 months. Although Chimeric Antigen Receptor (CAR) T cell therapies have been effective in blood cancers, their efficacy against solid tumours like GBM is limited due to issues like antigen variability and immune suppression within the tumour environment. Recent trials targeting the GBM-specific antigen EGFRvIII with CAR T cells demonstrated safety but not lasting effectiveness. Furthermore, while immune checkpoint therapies show promise, the immunosuppressive nature of GBM's microenvironment hinders their success. A novel approach combining intratumoural CAR T cell therapy with modulation of the tumour's immune environment—specifically targeting the myeloid cells that often support tumour growth—has been proposed. This strategy, leveraging both direct CAR T cell targeting and manipulation of the tumour's own defenses, could potentially lead to complete clearance of GBM and represents a significant step forward in the treatment of this and potentially other solid tumours.

Authors conducted large scale animal models of a heterogeneous mosaic model of GBM, implanted orthotopically, and show superior efficacy of the EGFRvIII-SGRP secreting CART cells. The authors demonstrate their SGRP secreted vIII-specific CAR leads to increased myeloid cell mediated phagocytosis of GBM. The authors also demonstrate utility of the SGRP CART cells in a mouse model of peripheral lymphoma, highlighting the broader application beyond GBM.

Overall the paper presented is very comprehensive, well written and the figures are presented nicely. The results are noteworthy and significant.

Response

Dear Reviewer,

Thank you for your encouraging feedback on our manuscript. We're grateful for the positive comments on the comprehensiveness of our study and the significance of our proposed approach for treating GBM and, potentially, other solid cancers. We welcome your suggestions for further enhancing the manuscript and provide a point-by-point response to your comments below. We

revised the manuscript accordingly, with all changes highlighted in yellow. We look forward to your comments on the revised version. Thank you for your consideration.

I list three small suggestions below that would enhance it further.

1) Authors demonstrate CAR specificity and CAR T cell in vitro cytotoxicity, 24 hour degranulation (LAMP1 exposure) and IFNg release against GBM cell lines. Interestingly, they generate CD4+ CART cells almost exclusively. It would be beneficial for the authors to more specifically address their proposed mechanism of action given CD8+ T cells are not present, and they have demonstrated CD4 CART cells are degranulating (ie. Discussion is worded such that the may be ruling out perforin/gnz mediated death in favour of indirect killing via IFNg/TNF. In any case it isn't clear).

1.1. We added PMIDs 37248395, 37316667, 29769444, 25378643, 36593069, and 35110735 as references 54-59 and extended the discussion to provide a better overview of CD4$^+$ CAR T cells and their purported mode of action in our study (manuscript lines 425-431 and 433-436). We also updated Fig. 8 and its legend to reflect these changes. Furthermore, we replaced the gene/protein "CD107a" with its HGNC-approved symbol "LAMP1" throughout the manuscript.

2) Figure 2. The Y-Axis in figure2 is slightly problematic (ie. Fig 2a-c starts at 5e4, then goes to 2.5e5, then to 4.5e5 etc). This axis does not follow either a linear OR logarithmic progression. This is not standard practice as it can lead to misinterpretation of the information presented. I strongly suggest the authors regraph using either a linear or Log scale, to present the range of values.

1.2. We corrected the mistake in the Y-axes of Fig. 2a-c. We also noticed a similar issue in Supplementary Fig. 2c,d, and corrected those axes as well.

Similarly, the graph in Fig2g, jumps from0, to 5e3, 1e4, then to 1.5e4. This is misleading.

1.3. The Y-axes of Fig. 2g follow a linear progression (from 0 to 5e3, to 1e4, to 1.5e4, and so on). The relevant ranges are shown for antibody-mediated blockade (range: 0 to 2.5e4 with major ticks every 5e3) or CAR supernatant-mediated blockade (range: 0 to 1e4 with major ticks every 2e3). The data are plotted separately since antibody-mediated and CAR supernatant-mediated

blockade are not directly comparable in dose or kinetics of blocking agent (aCD47 vs SGRP). Thus, each graph compares one blocking agent to its relevant control.

3) T cells also express CD47, and in fact CD47 expression is critical for CART cell survival in vivo (PMID: 36918226 & should be cited).

1.4. Thank you for this important comment. We added PMIDs 36918226 and 37549290 as references 29 and 30 (manuscript lines 153-154). Please refer also to responses 1.5 and 1.6, where we discuss the recent publication by Yamada-Hunter et al.

In the 'on-cell CD47 blocking assay' how did the authors control for CD47-mediated fratricide between the T cells? It would be beneficial to show the level of CD47 expression on the T cells.

1.5. As stated in the study by Beckett et al. (PMID 36918226), "T-cell fratricide can occur when CAR-targeted antigens are expressed on the T-cell surface." However, unlike the CV1-CAR T cells in that study, the CAR T cells in our study do not target CD47 via a CAR. Moreover, neither CAR target employed in our study (CD19 or EGFRvIII) is expressed on the T cell surface. aEGFRvIII-SGRP CAR T cells may block CD47 in an autocrine or paracrine manner, but to our knowledge, SIRPα-mediated T cell fratricide has not been described. Alternatively, to inquire about a potential downregulation of CD47 on T cells exposed to autocrine SGRP, we stained aEGFRvIII CAR and aEGFRvIII-SGRP CAR T cells from 3 healthy donors but found no differences in CD47 surface expression between them, as depicted in the revised Supplementary Fig. 2f and described in the corresponding results section (manuscript lines 155 and 156).

In the study by Dacek et al. (PMID 36696633) that uses a similar strategy in a non-solid tumor setting, the paracrine delivery of a high-affinity SIRPα analogon (CV1) secreted by a CD19-targeting CAR T cell equally resulted in superior efficacy and TME reprogramming, especially in combination with monoclonal antibodies. CV1 was difficult to detect in the blood and most probably preferentially bound to the highly CD47-expressing tumor cells at the site of CAR T cell targeting. The authors did not observe T cell apoptosis both in xenograft and syngeneic models, in contrast to the recent paper by Yamada-Hunter et al. (PMID 38750365), which employed anti-CD47 antibodies as a CAR T cell 'safety switch'.

The authors correctly hypothesise that SGRP may be partially captured by CAR T cells. Given CD47 expression on T cells, it is intriguing that the delivery of anti-CD47 antibody in animal

models (Fig 3) in combination with CART cells, does not deplete the human CAR T cells from the animal, and therefore render the EGFRvIII therapy ineffective. The authors should show the levels of CART cells in the blood to demonstrate engraftment of the therapy, and to show that the added survival benefit of antiCD47 blockade is CART cell mediated.

1.6. Thank you for this comment. The combination of CD47 antibodies and CAR T cells was not synergistic, contrary to what we had expected. We saw a similar performance of 20% overall survival in the aEGFRvIII CAR monotreatment and aEGFRvIII CAR + aCD47 combination treatment groups (Fig. 3c). Likely, the main reason for this observation is that antibody-based CD47 blockade may induce GAM-mediated phagocytosis of tumor cells but also CAR T cells, due to the generalized distribution of aCD47 when infused intracranially and systemically. Concomitant infusion of T cells and anti-CD47 antibodies may serve as a ‘safety switch’ in CAR T cell treatments, as mentioned in response 1.5. The recent paper by Yamada-Hunter et al. (PMID 38750365) showed that adoptively transferred T cells are phagocytosed by macrophages if concomitant anti-CD47 treatment is employed. The authors elegantly replaced the native CD47 on T cells with an engineered form not bound by anti-CD47 antibodies. This approach enhanced CAR T cell persistence and performance and promoted the recruitment of macrophages into solid tumors in any local CAR T cell treatment setting (Fig. 5). In our brain FC experiments, we equally observed an enhanced GAM response (Fig. 6).

Hence, in our study, after the disappointing performance of CAR T cell and anti-CD47 antibody combination, we were impressed by the high efficacy of aEGFRvIII-SGRP CAR T cells. aEGFRvIII CAR and aEGFRvIII-SGRP CAR T cells displayed similar levels of CD47 surface expression in cell cultures (Supplementary Fig. 2f), which suggests that SGRP might preferentially bind to tumor cells overexpressing CD47, making them more prone to phagocytosis, while CAR T cells remain active. Furthermore, the mode of infusion of SGRP may be a crucial factor for its efficacy compared to the antibody-based blockade. SGRP is constitutively secreted by CAR T cells as they engage EGFRvIII$^+$ tumor cells in niches shared with bystander EGFRvIII$^-$ tumor cells. CAR T cell activation within these niches may result in higher levels of locally secreted SGRP and other inflammatory cytokines. Collectively, these GAM stimulatory molecules result in increased GAM phagocytic function, leading to tumor clearance.

Since we also observed an increased efficacy of systemically infused aCD19-SGRP CAR T cells in a CD19$^+$ lymphoma flank model (Fig. 7c), a significant phagocytic clearance of SGRP-secreting CAR T cells by host phagocytes is unlikely. Similar observations were made by Dacek et al. (PMID 36696633), who used CAR T cells that secreted a high-affinity SIRPα analogon

(CV1). Compared to our optimized SIRPγ (SGRP), CV1 has a 9-fold lower affinity to human CD47, while SGRP and CV1 have similar half-lives (PMID 23722425, manuscript reference 26). Phagocytosis is a multistep process involving numerous carefully balanced ‘eat me’ and ‘don’t eat me’ signals. We thus hypothesize that tumor cell lysis and cellular stress can be additional stimuli for preferential phagocytosis of tumor cells compared to intact CAR T cells in case a CD47 blocker is constitutively released into the TME.

We performed adequately controlled survival experiments and validated the results of our GBM and lymphoma models at least twice. We also provide evidence of the presence of CAR T cells in the brains of our GBM mouse models at different time points soon after intracerebral treatments (Fig. 5, Fig. 6, Supplementary Fig. 4d-f, and Supplementary Fig. 6), demonstrating engraftment of the therapy in this model.

As requested by the Reviewer, we conducted a FC analysis of peripheral blood from both EGFRvIII-mosaic GBM and CD19^{+} lymphoma xenografts. For the GBM model, we collected blood 24 h after CAR treatment to monitor early toxicity (Supplementary Fig. 7e) and did not detect CAR T cells at this early stage post-treatment in the blood of either aEGFRvIII CAR- or aEGFRvIII-SGRP CAR-treated mice (gating strategy of GBM blood FC is provided in Fig. R1a; mCherry gate in Fig. R1b), indicating that the CAR T cells remained engaged in intracerebral tumor targeting 24 hours after intracerebral injection. For the lymphoma model, blood was collected 11 days after CAR treatment (corresponding to day 14 post tumor implantation). At this time point, aCD19 CAR- and aCD19-SGRP CAR-treated animals displayed clear differences in tumor burden compared to control conditions (Fig. 7d). However, no CAR T cells were detected in the blood, regardless of treatment condition (gating strategy of lymphoma blood FC is provided in Fig. R1c; mCherry gate in Fig. R1d). The outcome of the experiment illustrated in Fig. 7 suggests that systemically infused CD19-targeting CAR T cells effectively home to the tumor site, significantly controlling tumor growth and improving survival. In contrast, EGFRvIII-targeting CAR T cells may be quickly eliminated due to the lack of a target in these animals.

Reviewer #2 (Remarks to the Author): with expertise in glioblastoma, cancer immunology

This work addresses a clinical important issue such as the immunosuppressive tumor microenvironment impeding immunotherapy, including CAR-t approaches. The authors propose the way to overcome those obstacles with a novel CAR-T cell approach by combining targeting tumor associated myeloid cells with CAR-T. They engineered a CAR T cell against epidermal growth factor receptor variant III (EGFRvIII) expressing cells that constitutively secrete a signal regulatory protein gamma (SIRPγ)-related protein (SGRP) with high affinity to CD47. Blockade of CD47 augmented phagocytic function of glioma associated microglia and macrophages in GBM-bearing mice and elicited antitumoral responses in vivo, but due to the ubiquitous expression of CD47, the therapy had side effects. Anti-EGFRvIII-SGRP CAR T cells eradicated orthotopic EGFRvIII-mosaic glioblastoma (GBM) xenografts in vivo and promoted GAM-mediated tumor cell phagocytosis. EGFRvIII is expressed in approximately 40% of GBMs. In another tumor model mouse CD19+ lymphoma, anti-CD19-SGRP CAR T cell therapy was superior to conventional anti-CD19 CAR T. Tumor rechallenge of aEGFRvIII-SGRP CAR-cured mice resulted in prolonged survival. Altogether, data convincingly show that combining a specific modulation of GAMs with anti-EGFRvIII targeted CAR T cells produces the sustainable anti-glioma effects.

The results are noteworthy and of significance to the field of neurooncology and in general, cancer immunotherapy.

The methodology sound and the work meets the expected standards.

Technically, this is an elegant and comprehensive work carefully presenting activity and specificity of the created anti-EGFRvIII-SGRP CAR T cells. The results were collected with the impressive scope of methods. Important aspects of characterization of EGFRvIII-SGRP CAR T cells were considered and necessary controls were applied, including testing of targets of interest on multiple cell lines: 4 GBM cell lines (U251, U87, U251vIII with transgenic overexpression EGFRvIII, BS153 with endogenous EGFRvIII expression), Raji Burkitt's lymphoma cell line and neural stem cell (NSC) line (NSC197). aCD19 CAR and aCD19-SGRP CAR were used as controls in GBM experiments and Raji as CD19+ 112 target cells. It should be appreciated that the authors performed experiments with numerous and appropriate controls: the production of SGRP was confirmed by proteomics; in vitro efficacy of EGFRvIII-SGRP CAR was tested against 2 glioma cell lines; activity of SGRP from CAR T was compared to anti-CD47 antibody, etc. Some minor

drawbacks were identified but those can be remedied by a proper revision and the better explanation of the author's viewpoint.

Response

Dear Reviewer,

Thank you for your kind words regarding the technical elegance and comprehensive experimental design of our study. We are pleased that you found the work to have been meticulously conducted and our experiments adequately controlled. We addressed the identified drawbacks, providing a clearer explanation of our viewpoint in our point-by-point response to the comments below. We revised the manuscript accordingly, with all changes highlighted in yellow. We look forward to your thoughts on the revised version. Thank you for your consideration.

Comments:

1. The combination of aEGFRvIII CAR + aCD47 failed to improve survival compared to aEGFRvIII CAR monotherapy (20%), while aEGFRvIII-SGRP CAR was extraordinarily potent , with 94.7% overall and 63.2% tumor-free survival. This result should be better explained.

2.1. We provide potential explanations for this effect at the end of the relevant results section of the manuscript (manuscript lines 234-242). The striking difference in the outcome of the two CAR + CD47 blocking combinations (aEGFRvIII CAR + aCD47 and aEGFRvIII-SGRP CAR) can be explained mainly by differences in kinetics, mode, and frequency of infusion. Specifically, SGRP is constitutionally secreted by effector cells that come in direct contact with target cells and GAMs, releasing SGRP in the vicinity of "bystander" antigen-negative tumor cells. In contrast, aCD47 was given intratumorally twice and then continued as an i.p. regimen for two additional weeks. The dosage, kinetics, and intermittent nature of aCD47 infusions were crucial factors for the disappointing effect observed for aCD47 monotherapy and aCD47 combination with CAR T cells. Moreover, we would like to refer to response 1.6 to Reviewer 1 regarding this important comment and highlight the recent publication by Yamada-Hunter et al. (PMID 38750365) that provides an important explanation for this observation.

2. In the description of the Fig.3 they write :"that a majority of EGFRvIII- tumors were eradicated after aEGFRvIII-SGRP CAR treatment". Why it so? This was EGFRvIII-mosaic GBM mouse

model and bystander effects on aEGFRvIII- cells can hardly explain such results. Please comment on this issue.

2.2. We would like to clarify that, for lack of a better term, this so-called ‘bystander effect’ on $EGFRvIII^-$ tumor cells is, in fact, a targeted local secretion of SGRP in the vicinity of $EGFRvIII^-$ ‘bystander’ cells during the CAR-mediated killing of $EGFRvIII^+$ tumor cells. We attempted to reproduce this effect in phagocytosis assays *in vitro* by combining EGFRvIII-mosaic tumor cells with donor-matched macrophages and CAR T cells (Supplementary Fig. 2g). In these short-term assays, we found no significant benefit of the presence of SGRP in terms of macrophage polarization and effector function, determined by spectral FC. We specifically assessed the expression of typical macrophage polarization markers (CD163, CD206, HLA-DR, and CD86), phagocytic marker CD209, and antigen presentation marker SIGLEC-1, as well as the fractions of phagocytosed $EGFRvIII^-$ U87 and $EGFRvIII^+$ U251vIII tumor cells. We described the results and offered some hypotheses in the manuscript (manuscript lines 157-164, 166 and 167). It’s important to note several aspects in which these *in vivo* and *in vitro* setups differ. First, the effector cells of SGRP-mediated CD47 blockade (GAMs) are far more numerous, lineage-diverse, and naturally polarized in our orthotopic GBM models than *in vitro*, where we applied naïve M0-like monocyte-derived macrophages. In fact, microglia subtypes made up between 70-80% of the total number of GAMs in our GBM models (Fig. 6c) and were also responsible for the majority of tumor cell phagocytosis (Fig. 6h,i). Second, although CAR T cells release SGRP constitutively, continued and prolonged exposure to SGRP may be required for effective CD47 blockade, leading to GAM phagocytic modulation. Our GBM mouse models are better suited to assess the effect of sustained SGRP-induced GAM modulation. Lastly, the dilution of SGRP in relatively large cell culture volumes (100 μL per well) may preclude it from achieving the required concentration for effective blockade. In contrast, in the *in vivo* setting, higher concentrations of SGRP can be delivered by CAR T cells locally to tumor cells in niches highly infiltrated by GAMs.

3. The statement “ a potential persistence of CAR T cells and/or antitumor GAMs in the brains of these animals” is not supported by any evidence.

2.3. To test our hypothesis that residual CAR T cells persist in the brain of aEGFRvIII-SGRP CAR-treated animals at tumor-rechallenge (day 90 after the original tumor implantation), we extended our CD3 staining IHC analysis to the brains of animals treated with aEGFRvIII-SGRP CAR who reached day 90 post tumor implantation. In CD3-stained IHC sections, we did not detect

significant numbers of CAR T cells in the brain (n = 10 aEGFRvIII-SGRP CAR-treated d90 brains). Supplementary Fig. 5b shows an exemplary brain micrograph with only a few $CD3^{+}$ cells localizing in the lateral ventricle. To better visualize CAR persistence in day 90 brain regions, we injected a small cohort of mice (n = 6) with EGFRvIII-mosaic GBM and treated them with aEGFRvIII-SGRP CAR. Upon reaching day 90 post tumor implantation, the brains were collected for FC analysis. Each brain was carefully dissected into three regions: tumor-implanted hemisphere, contralateral hemisphere, and meninges. We found low frequencies of CAR T cells ($mCherry^{+}$ cells) in all three brain regions but significantly higher in the meninges (Supplementary Fig. 5c). These results were described in the revised manuscript (manuscript lines 229-233). Thus, the observed effect on survival upon tumor rechallenge (Fig. 3g) is unlikely to be attributable to the small number of brain-residual CAR T cells localizing at the brain periphery. Consequently, we propose that aEGFRvIII-SGRP CAR treatment may induce long-term changes in innate immune cell populations or within the brain microenvironment upon clearance of EGFRvIII-mosaic tumors, rendering the brain less conducive to repeated tumor cell engraftment and proliferation.

4. Targeted proteomic analysis of plasma samples was performed to identify proteins differentially expressed between groups and revealed some hints. The analysis was done on 2 samples and such important finding should be verified with quantitative method (ELISA) on more samples.

2.4. We want to clarify that the targeted proteomic analysis was performed using plasma samples from 6 animals per treatment group, not 2 (datapoints in Fig. 4a,c). All 6 biological replicates in each treatment group were pooled for the differential expression analysis of aEGFRvIII CAR and aEGFRvIII-SGRP CAR shown in Fig. 4b. The method we used, a proximity extension assay provided by Olink proteomics (www.olink.com), is a validated high throughput method for protein detection and quantification. Compared to ELISA, this method is highly sensitive and allows multiplexed analysis of large panels of proteins using extremely low sample volumes (1-5 µL).

5. I am not fully convinced about the use of the EGFRvIII-mosaic GBM mouse model obtained by mixing two glioma cell lines with EGFRvIII positive and negative cells, respectively. They implanted U251 and U87 cells which means they implanted two completely different tumors into one host. I understand that the cells should be distinct but these tumors are entirely different so their responses to therapy could be related to their genetic background not to a lack of EGFRvIII. Relevance this model to human pathology is unclear.

2.5. We understand the criticism of the model. Initially, we intended to use the same parental and EGFRvIII-overexpressed cell line to generate the EGFRvIII-mosaic brain tumor model. We carefully validated the engraftment of various cell lines before performing the large-scale *in vivo* experiments. We also considered a cell line with endogenous EGFRvIII expression, BS153, at first, which did not form *in vivo* GBM-like tumors at sufficiently high penetrance. In our preliminary experiments, we identified engraftment discrepancies between U87 and U251 parental and EGFRvIII-overexpressing cells. U87vIII and U251 cells did not consistently form tumors and displayed a longer-term survival, while U251vIII and U87 cells consistently led to rapid-onsetting *in vivo* tumors. We employed a mixed model using U87 and U251vIII, which resulted in complete morbidity of mice in their respective cohorts (Fig. R1e; U87 median survival: 42 d; U251vIII median survival: 29 d; U87 vs U251vIII $p = 0.0006$).

On the other hand, GBM is an explicit example of tumor heterogeneity, which makes treatment of this tumor so difficult. Different clones with completely different profiles co-exist in GBM, resulting in high genomic and phenotypic intratumoral heterogeneity as stated, e.g., by Snuderl et al. (PMID 22137795), in the commentary by Ene and Fine (PMID 22172718), and many others. Therefore, we would also like to highlight that our model is, albeit reductionist, reflecting a certain degree of the natural tumor's heterogeneity. Although we agree that the model is not ideal, we confirmed that EGFRvIII-negative U87 cells elicited a similar IFNγ response compared to EGFRvIII-negative U251 cells in co-cultures with CAR T cells *in vitro* (Fig. 2e).

6. The authors state that animals were treated with therapy twice – at 7 and 14 days – after intracranial tumor implantation using the same stereotactic coordinates. It is not simple to deliver intratumoral injection into the same place. I wonder if this was monitored somehow.

2.6. We used the same stereotactic frame and parameters for digitally controlled intracranial injections of tumor cells and treatments. The micrometer screw for positioning the syringe is controlled computationally by the Neurostar robotic stereotaxic system we have installed at our facility (https://neurostar.de/). The injection speed and removal of the syringe are equally controlled computationally. There may have been minimal variations in the exact coordinates of each intracranial injection due to the fixation of the animal on the stereotactic frame, which is done manually. However, we use the same cranial burr hole for all injections, and the coordinates are set digitally. We would also like to refer to the histological stainings of Fig. 5e and Supplementary Fig. 6b, which show the presence of CAR T cells within and around the tumor in all conditions. In

some instances, we identified CAR T cells migrating to distant sites, even the contralateral side of the brain, highlighting the migratory potential of CAR T cells.

7. They found only tumor remnants in aEGFRvIII-SGRP CAR treated group and did not the immune TME in this condition. Actually, despite small tumor sizes that adjacent area could contain many immune cells with interesting phenotypes and the analysis could reveal interesting observation. From my expertise even is tumor is residual the immune infiltrate could be the same as in large tumor, only cells have different phenotypes. Those samples should be analyzed as well and in fact, they are the most interesting group.

2.7. We assume this comment refers to the histology shown in Fig. 5. We would like to point out that although we do not show multiplex IF stainings on d21 aEGFRvIII-SGRP CAR-treated brains due to a lack of tumors in this condition at this time point, we stained and quantified CD3 and CD68 in d13 aEGFRvIII-SGRP CAR-treated brains (these stainings were combined in the revised Supplementary Fig. 6). This superficial assessment showed CAR T cell persistence and GAM recruitment. We agree that the immune cells in the TME of these tumors, even if the tumors are small/residual, may have interesting phenotypes. For that reason, we performed in-depth FC analyses of aEGFRvIII CAR- and aEGFRvIII-SGRP CAR-treated brains (Fig. 6, Supplementary Fig. 8, and Supplementary Fig. 9) with the goal of defining the abundance, phenotype, and function of these immune cells.

8. TSNE plots for aEGFRvIII-CAR and aEGFRvIII-SGRP CAR in the Fig.6f look the same (correct TNSE on Y axis). One would expect differences in 8 presented subpopulations between groups considering data presented on reprograming of GAMs.

2.8. Thank you for the comment. We corrected the typo on the Y-axis. Although dimensionality reduction can sometimes mask differences between conditions, we can still appreciate small differences between aEGFRvIII CAR and aEGFRvIII-SGRP CAR treatments in the TSNEs of Fig. 6f, namely in the density of homeostatic MG and CD11c^{hi} MG subpopulations. Using both conventional FC gating and FlowSOM analyses, we observed clear differences between the two treatments in terms of their induced myeloid cell fractions (Fig. 6c), GAM phenotypes (Fig. 6d,e), and aggregated marker expression (Fig. 6g), which identified a significant difference in the phagocytic potential of GAMs (homeostatic MG, activated MG, CD11c^{hi} MG, and moMacs) in the aEGFRvIII-SGRP CAR condition. These analyses show differences in function and relative

abundance of GAM subpopulations, which we believe are a direct consequence of enhanced GAM reprogramming by aEGFRvIII-SGRP CAR T cells.

9. I do appreciate showing the higher efficacy of aCD19-SGRP CAR in another tumor model (Raji lymphoma cells). But the number of animals are small n=5-6 for most interesting groups and n=4 for other groups is not sufficient. Extended fig.4 b shows that BLI measurements of tumors and in the control group 2 out of 10 mice do not show any BLI signal.

2.9. We repeated the lymphoma experiment with a similarly sized cohort, which showed consistent results with the first trial. Data from both trials were pooled for a total of n = 10 animals per group (n = 9 in the 'vehicle' control; Fig. 7b). The log-rank test comparing the overall survival of aCD19-SGRP CAR and aCD19 CAR groups now shows statistical significance (p = 0.012; Fig. 7c). The short comment about Fig. 4b is addressed together with comment 10, below.

10. Extended fig.4 b shows bioluminescence (BLi) data indicating that EGFRvIII+ and EGFRvIII- tumor cells were equally eradicated with aEGFRvIII-SGRP CAR (only 1 out of 10 EGFRvIII- showed slight BLi). The authors attribute high effectiveness of EGFRvIII+ and EGFRvIII- tumor cells were equally eradicated with aEGFRvIII-SGRP CAR against EGFRvIII-tumors to the bystander effect but than it would mean the lack of selectivity in the treatment. In aEGFRvIII CAR + aCD47 treated group the BLi signal was visible in 9 out of 10 10 EGFRvIII-tumors indicting a high selectivity of the treatment and the bystander effects on viability was not evident.

2.10. The time point of the comparison shown in Fig. 4b was chosen to highlight the stark differences in tumor burden between aEGFRvIII-SGRP CAR-treated animals and those in other relevant groups (aEGFRvIII CAR and aEGFRvIII CAR + aCD47). In these experiments, animals were randomized across all treatment/control groups, which could have led to differences in the proliferation of EGFRvIII^{+} and EGFRvIII^{-} tumor subsets in a mosaic tumor model. We hypothesize that the effect of aEGFRvIII CAR activation/cytotoxicity in the context of mosaic tumors like the one employed in our study may lead to the indirect killing of a fraction of target-negative/bystander tumor cells. Combined with slight variations in the relative engraftment of U87 and U251vIII cells in some animals, the absence of any tumor in 2 out of 10 mice in the aEGFRvIII CAR group at this time point could be due to a lack of selectivity.

However, we would like to distinguish two types of "bystander effect": (1) the indirect killing of EGFRvIII^{-} tumor cells by anti-EGFRvIII CAR T cells, and (2) the increased targeting of tumor

cells by GAMs using CD47-blocking agents (anti-CD47 antibodies or SGRP). EGFRvIII$^-$ bystander tumor cells were only consistently eliminated in the aEGFRvIII-SGRP CAR condition. Although both types of “bystander effect” could be responsible for this effect, there is a clear benefit of SGRP-secreting CAR T cells in this model, suggesting that SGRP is required for the complete eradication of EGFRvIII-mosaic tumors. Moreover, paracrine and continuous intratumoral secretion of SGRP seems effective and required for this bystander-killing effect since aCD47 treatment in combination with conventional aEGFRvIII CARs failed to show efficacy.

The combination of CD47 antibodies and CAR T cells did not yield the expected synergistic effect, with both aEGFRvIII CAR and aEGFRvIII CAR + aCD47 groups showing similar 20% overall survival. This may be due to CD47 blockade making T cells more prone to phagocytosis by host GAMs, acting as a potential 'safety switch'. A study by Yamada-Hunter et al. (PMID 38750365) demonstrated that engineered CD47 on T cells avoids this issue, enhancing CAR T cell performance and macrophage recruitment. In our study, aEGFRvIII-SGRP CAR T cells showed high efficacy despite normal CD47 expression, likely due to SGRP’s preferential binding to tumor cells overexpressing CD47. This leads to increased local inflammatory cytokine levels and enhanced phagocytic function of GAMs, promoting tumor clearance. This hypothesis is supported by the efficacy of aCD19-SGRP CAR T cells in a CD19$^+$ lymphoma model (Fig. 7) and similar findings by Dacek et al. (PMID 36696633), where the authors employed a similar strategy against CD19 tumors with a different CD47 blocker. We hypothesize that tumor cell lysis and cell stress further stimulate the preferential phagocytosis of tumor cells over CAR T cells when CD47 blockers are present, potentially explaining the differences observed in the BLi of Supplementary Fig. 4b. In support of this hypothesis, we show data from a new *in vivo* experiment (Supplementary Fig. 4d-f) where we investigated the effect of CAR target priming with target-positive tumor cells on CAR T cell persistence and myeloid immune cell infiltration into the brain. This experiment was described in detail in the revised manuscript (manuscript lines 213-225). We also refer to response 3.8 to Reviewer 3, who suggested this experiment.

11. Extended fig. 6 shows CD3 staining in brains with tumors: the IHC images show differences between treatment groups but there is no statistical differences in the CD3 abundance. The choice of IHC staining images was not fortunate as those data do not correspond (more CD3 staining in EGFRvIII-CAR and EGFRvIII-CAR+aCD47 groups. Why in aEGFRvIII-SGRP CAR group the staining is shown at day 13 while in other groups at day 21?

2.11. Thank you for this comment. We would like to point out that the quantification plots in the revised Supplementary Fig. 6 show the percentage of stained cells (CD3 or CD68) among all cells in the relevant tumor region (tumor center or tumor rim). The definition of these tumor regions is detailed in the methods section. We found significantly smaller tumors but also fewer CAR T cells present in aEGFRvIII-SGRP CAR T-treated brains compared to those of other treatments. This explains the similar ratios of CAR T cells to tumor cells among all CAR treatments and the lack of statistical differences in CD3 abundance.

As we mentioned in response 2.7., aEGFRvIII-SGRP CAR T-treated brains at d21 had only tumor remnants, which did not allow for defining tumor center and tumor rim regions to quantify CD3 and CD68 IHC stainings. For this reason, we chose to collect aEGFRvIII-SGRP CAR T-treated brains at an earlier time point (d13), where tumors were small but still present in most animals. This was described in the manuscript (manuscript lines 278-285).

12. The CD47 treatment leads to systemic toxicity. The authors state “off-tumor toxicity …was clinically absent in our mouse model” as they delivered the treatment intratumorally. However, some observed effects on myeloid populations could be explained SGRP inflammation and there is no data on local toxicity.

2.12. Thank you for the comment regarding toxicity in our tumor models. We have revised the manuscript (manuscript lines 303-315) and added a new Supplementary Fig. 7 in response to this important comment. Although aEGFRvIII-SGRP CAR treatment was extremely efficacious in clearing EGFRvIII-mosaic GBM, we found no evidence of long-term xenogeneic graft versus host disease, as assessed by treatment-induced changes in spleen size, weight, plasma IL6 levels, plasma CRP levels, onset of anemia, thrombocytopenia and neutropenia (PMID 23772685 and 30214443). While we observed transient changes in posture and locomotion of the animals after intracranial tumor implantation and treatment administration, these clinical signs were treatment-unspecific and rather a result of the invasive intracranial interventions.

We observed no significant splenomegaly (Supplementary Fig. 7a) and only a transient weight loss in a few animals following the first treatment with aEGFRvIII-SGRP CAR (Supplementary Fig. 7b). This suggests that any toxicity was short-lived, as indicated by the rapid return of the animals' weight to normal levels by the time the second treatment dose was administered. Interestingly, the second aEGFRvIII-SGRP CAR treatment did not elicit weight loss in any animal, suggesting a batch effect or a cytokine storm event potentially correlated with higher tumor burden at treatment initiation. We also found no signs of hair loss or epidermal

hyperplasia in our cohorts (PMID 29662203). Furthermore, IL6 and CRP, markers typically assessed in CAR T cell toxicity studies (PMID 34166502), were not significantly elevated in the plasma of animals treated with aEGFRvIII-SGRP CAR (Supplementary Fig. 7c,d). Moreover, we conducted a hematological investigation and found no evidence of anemia, thrombocytopenia, or neutropenia elicited by CAR therapy (Supplementary Fig. 7e).

Myelin stainings of aEGFRvIII CAR and aEGFRvIII-SGRP CAR-treated brains revealed normal myelination of the corpus callosum despite intracranial CAR T cell infusion in our model (Supplementary Fig. 7f-g). Thus, we conclude that aEGFRvIII-SGRP CAR treatment may elicit acute toxicity in some animals, as evidenced by weight changes after the first aEGFRvIII-SGRP CAR treatment. However, these events were rare and short-lived, resolving autonomously within hours to a few days.

Minor comments:

Abstract: Correct the sentence: “CAR T cell against epidermal growth factor receptor variant III (EGFRvIII) that constitutively secretes a signal regulatory protein gamma (SIRPγ)-related protein (SGRP) with high affinity to 39 CD47”. The authors meant EGFRvIII cells expressing….Correct: “GAM-mediated tumor cell uptake”. In case of cells it is phagocytosis. In biology “an uptake” has very a specific meaning and refers to transport of ions or compounds.

2.13. Thank you for pointing out these inaccuracies. We corrected the sentence in the abstract (manuscript lines 33 and 34) and replaced all instances of “cell uptake” with “phagocytosis” throughout the manuscript.

This sentence is not clear: “Conversely, aEGFRvIII CAR treatment (median survival: 65.5 d) led to 40% survival 90 d after tumor implantation, starkly contrasting with aCD19 CAR control (median survival: 29 d)”. Why 90 days if mice do not survive that long?

2.14. Although mice in control groups (vehicle, isotype, and aCD19 CAR) do not survive past 55 days, differences between the main treatment groups (aEGFRvIII CAR, aEGFRvIII CAR + aCD47, and aEGFRvIII-SGRP CAR) can only be fully appreciated between days 60 and 90 (Fig. 3c). Due to a possible delayed outgrowth of residual tumor cells, a major mechanism of GBM resistance to targeted therapies, we monitored animals for up to 90 days in the GBM survival

cohorts. We think this allows for greater resolution when comparing the effect of SGRP-secreting CARs to other treatments regarding overall and tumor-free survival (Fig. 3c,d).

Reviewer #3 (Remarks to the Author): with expertise in glioblastoma, cancer immunology

This manuscript describes the construction of CAR-T cells with secretive CD47 blockers to achieve inhibition of brain tumors. I am concerned with the overall novelty of the study, which is modest at best and the selection of animal models used in the study. In addition, there are many major experiments missing that need to be done to support the conclusions of the paper.

Response

Dear Reviewer,

Thank you for your feedback. We welcome the opportunity to further clarify the novelty of our approach and experimentally address many of your concerns to support our conclusions. We carried out a comprehensive revision of our manuscript, taking into account each of the specific questions raised and provide a point-by-point response to the comments below. All changes to the manuscript are highlighted in yellow. We look forward to your thoughts on the revised version. Thank you for your consideration.

Please see my specific comments as follows:

1. The novelty of the study is a particular concern. It has been reported that CAR-T with secretive SIRPa can block the CD47 pathway and enhance the therapy efficacy (PMID: 35110357). Although the current work targets a different kind of tumor, the concept is not novel and is merely an extension of prior work.

3.1. We thank the Reviewer for this comment and for bringing up this important study. We cited their work along with two other studies that target CD47 in combination with a CAR using SIRPα analogons (PMID 36696633 and 38266806; references 25, 48 and 49) in the discussion (manuscript lines 397-401). These works were published recently and are important and novel contributions to this competitive field. In the scientific community, it is of utmost importance to reproduce findings from colleagues with other means, and we are certainly strengthening and reinforcing the findings from Chen et al., Dacek et al., and Lu et al. with our strategy, albeit in a more challenging brain tumor setting. The tumors in their study were flank tumor models or leukemia models that represent tumor entities with diverse microenvironmental conditions than the brain tumor models we generated in our study. Preclinical validation of an armored CAR in complex, orthotopic brain tumor models is, therefore, novel and of high translational importance.

Furthermore, instead of using a SIRPα analogon, we armored our CAR with SGRP, which has a significantly higher affinity to CD47 than conventional SIRPα or CV1 (reference 26). SGRP has a K_D of 92 pM and a half-life of 44 min (reference 26), which renders the affinity of SGRP 9-fold higher than CV1 and more than 450'000x higher than that of endogenous phagocyte-expressed SIRPα (reference 26). This is another novel component that will yield a higher therapeutic benefit and potentially less toxic side effects since most of the SGRP is captured in the proximity of the tumor-targeting site, while a SIRPα analogon might be less stringently bound to CD47-expressing cells and more easily displaced by SIRPα expressed on the surface of immune cells. Furthermore, we present a more in-depth characterization of the immune tumor microenvironment and have included more controls than seen in the previous studies.

2. This work used SIRPg to bind with CD47, instead of the commonly used SIRPa. Is there any rationale to choose SIRPg? Does SIPRa and SIRPg blockades have similar effects? Or SIRPg works better?

3.2. As mentioned above, we use SGRP, a high-affinity CD47 blocker based on the V1 domain of human SIRPγ. The original amino acid sequence of SGRP was taken from the patent cited in the manuscript (reference 26). SGRP results from 9 amino acid modifications to the SIRPγ-V1 sequence, which enhance the affinity of SGRP to CD47, outcompeting SIRPα in the binding to CD47. We further modified SGRP to be secreted by CAR T cells with the addition of an IL-2 signal sequence and optimized its codon sequence for improved expression in human T cells (all sequences provided in Supplementary Table 2).

3. Experimental data is missing to characterize the secreted SIRPg. For example, the amount of protein secreted, especially the concentration of the protein in vitro and in vivo, to determine if this concentration is enough to make any therapeutic difference.

3.3. We provide evidence of SGRP cDNA in aEGFRvIII-SGRP CAR T cells from four healthy donors (Fig. 1h) and specific SGRP peptide sequences in supernatants of aEGFRvIII-SGRP CAR T cell cultures compared to conventional aEGFRvIII CAR T cells from two healthy donors (Fig. 1i and Supplementary Fig. 1g). While revising our manuscript, we undertook several efforts to provide an alternative visualization of SGRP recovered from cell culture and mouse samples. However, alternative modes of detection are limited due to a lack of specific SGRP antibodies. The following paragraphs summarize our efforts.

SGRP is derived from the N-terminus extracellular domain of SIRPγ. SIRPγ has a 10-fold lower affinity for CD47 compared to SIRPα. This is due to structural differences in the N-terminus domains of the two receptors, e.g., (1) Q37L reduces the binding affinity, though there are no structural explanations to support this; (2) D100N prevents salt bridge formation with K96 of SIRPα or K39 of CD47; (3) D100 in SIRPα forms salt bridges with both (PMID 18657508). These key amino acids that interact with CD47 have been introduced in the SGRP sequence and display homology with SIRPα. Therefore, we hypothesized that a SIRPα ELISA assay could detect SGRP from the supernatant or lysates of transduced T cells (Fig. R2a). We used donor-matched aEGFRvIII CAR T cell supernatants and lysates as a negative control for this assay. With this method, we could only detect non-specific background derived from both supernatants and lysates (Fig. R2b,c). Importantly, SGRP is derived from the binding domain of SIRPγ (SIRPγ-V1). Despite having a high sequence homology with SIRPα, it is unclear if this ELISA assay is targeted to the binding domain of SIRPα. Furthermore, it is also unknown if SIRPα and SGRP are sufficiently homologous for anti-SIRPα antibodies to cross-react with SGRP.

We then moved to a competition ELISA assay that uses immobilized CD47 as a capture agent (Fig. R2d). We incubated the ELISA plate with supernatants containing a fixed concentration of biotinylated (bt)-SIRPα. When SGRP is present in high concentrations, it would compete with SIRPα, leading to a decrease in absorbance as displayed in Fig. R2e. A commercially available anti-CD47 antibody was used for the standard curve. However, we did not appreciate significant differences between our samples regarding CD47 inhibition potential (Fig. R2f).

Given the background interference seen in the ELISA assays, we decided to move on to a more specific detection method using a new lentiviral vector encoding a 6xHis-tagged SGRP (aEGFRvIII-His.SGRP CAR; Fig. R2g). We hypothesized that the secreted His.SGRP could be detected without interference in the supernatant by Western Blot (WB) using an anti-His antibody. In accordance with published protocols (PMID 38513100), we performed WB analysis of mCherry-sorted CAR T cells (Fig. R2h, left image) but found no His-tag protein in CAR T cell supernatants or lysates (SGRP expected molecular weight: ca. 15 kDa; Fig. R2i). Due to the low mCherry intensity of this batch of transduced T cells, we also performed a WB analysis of transfected HEK293T cells, which had a strong mCherry signal (Fig. R2h, right image). Even then, we could not detect His-tagged protein in supernatant or cell lysates (Fig. R2j), although we could detect faint bands relative to mCherry in the lysates of transfected T cells in the blot imaged with a f595 Y3 filter (Fig. R2k). Given the short half-life of SGRP (ca. 44 min) and its relatively low expected concentrations in cell culture media and cell lysates, standard cell culture systems

coupled with methods such as ELISA and WB were not sensitive enough to provide an alternative visualization of the protein. In repeated efforts to measure SGRP in brain homogenates after local CAR T cell injection via LC-MS proteomics, we failed to detect the protein, probably again because of the low abundance, high capture rate by target cells and short protein half-life. However, in a commercial mammalian (HEK293T) expression system, we could show that His.SGRP is produced and secreted onto culture supernatants (Fig. R2I).

We hypothesize that in CAR T cell cultures, SGRP may be captured in autocrine loops by CD47 on CAR T cells due to its high affinity and the lack of other CD47 'consumer' cells in this setting. Furthermore, CAR T cells are maintained in culture and kept at a relatively low cell density (1×10^6 CAR T cells per mL), which might preclude us from recovering significant amounts of SGRP. The high affinity of SGRP for CD47 (92 pM) would suggest that even very small amounts of SGRP (<1 ng/mL) secreted and locally delivered by CAR T cells to tumor cells, could induce a biological effect on neighboring GAMs, as seen in our preclinical models.

In view of the urgent clinical translation of these findings that GBM patients could benefit from, we are focusing on two key areas: (1) large-scale production of recombinant SGRP using a mammalian expression system and (2) generating an SGRP-specific antibody to enable direct SGRP detection. To preserve SGRP function, we initially chose not to add a tag that may have interfered with protein folding or binding to CD47. Since we expect SGRP to be captured by CD47 in the tumor vicinity, its detection in peripheral blood might be difficult if the therapy is locally applied, as in our study. However, local therapy application will lead to reduced systemic toxicity. To facilitate SGRP detection without autocrine consumption in CAR T cell cultures, we are developing a CRISPR-Cas9 CD47 knockout Jurkat cell line. In the context of this revision, we do not have a highly sensitive assay for detecting SGRP in body fluids or tissues and must rely on LC-MS, large-scale HEK293T-based protein expression and transcriptional data.

4. In the GBM CAR-T trial (NCT01454596), the CAR-T were infused to patient. However, in the present study, CAR-T were intratumorally injected, which is expected to have better efficacy. Can the SGRP CAR achieve any therapeutic effect against brain tumor if injected intravenously? Also, intravenous injection is expected to have more toxicity since SIRPg will be secreted to the circulation and block the CD47 on RBC. Therefore, toxicity study should also be carried out in the more clinical-relevant intravenous injection model.

3.4. Recent clinical CAR T cell trials in GBM have used direct intratumoral or intrathecal injection as a preferred CAR T cell application route (NCT05660369, NCT02208362, and

NCT05168423). Tumor targeting is greatly improved, and hematological toxicity can be largely circumvented. Furthermore, conditional lymphodepletion, which is needed in peripherally infused CAR T cell therapy, can be avoided. In fact, the intratumoral and/or intrathecal application route is the most clinically relevant setting for brain tumors as it mitigates the risks of systemic CAR T cell-related toxicity to a great extent. Based on currently published clinical data, we note that systemic application of CAR T cells in brain tumors would be the second choice unless the homing to the tumor site could be greatly improved by yet-to-be-defined strategies.

Regarding the comment about toxicity, we performed a toxicity study, illustrated in the new Supplementary Fig. 7, encompassing analyses of spleen size, weight monitoring, plasma IL6 and CRP, hematological parameters, and myelin staining of brain sections of our intratumorally treated GBM model. Our findings were described in the manuscript (manuscript lines 303-315) and summarized in response 2.12 to Reviewer 2.

5. The brain tumor model used tumor: T cell ratio of 1:10, which is very high. How does this ratio compare to other brain CAR-T models and those trials used clinically?

3.5. It's important to note that the exact E:T ratio in our brain tumor models was not 10:1, as mentioned. We implanted 5 x 10^4 tumor cells intracranially and allowed tumors to develop over a 7-day period. Given the expected doubling time of approximately 48 h for U87 and U251vIII, we estimated that tumors would contain approximately 5 x 10^5 cells by d7, assuming a near-100% engraftment. This estimate served as a reference for determining the treatment dose and time points. Hence, at the first treatment time point (day 7), the approximate E:T ratio would have been roughly 1:1.

6. Figure 2 showed no SGRP-mediated benefit in vitro. How about adding macrophages to the in vitro co-culture model to test if there's any benefit?

3.6. We performed the suggested phagocytosis assay in an attempt to mimic the *in vivo* effect of aEGFRvIII-SGRP CAR T cells, albeit with disappointing results (Supplementary Fig. 2g), as described in the revised manuscript (manuscript lines 157-164). In response 2.2 to Reviewer 2, we offer potential explanations for this result, highlighting key differences between the *in vitro* and *in vivo* models and technical challenges of measuring the effect of SGRP in short-term, single-time point assays.

7. Some work is needed to justify the model selection. This work uses the mixture of two different cell lines to establish the model. One cell line is EGFRvIII + and one is -. It is possible that the two cell lines respond differently to CAR-T therapy or their growth characteristics are different. Therefore the observed phenotype may due to their innate nature, not EGFRvIII status. I recommend use the same cell line with or without EGFRvIII expression and label them with different luciferase to monitor the treatment outcome.

3.7. Regarding this important comment about the EGFRvIII mosaic tumor model, we would like to refer to response 2.5 to Reviewer 2.

8. Figure 3f showed that majority of EGFRvIII- tumors were eradicated after aEGFRvIII-SGRP CAR treatment. But what's the mechanism? When treating EGFRvIII- tumors, the aEGFRvIII-SGRP CAR is expected to work similar to aCD19-SGRP CAR, since in both cases, there's no target for the CAR. Then why aEGFRvIII-SGRP CAR can inhibit EGFRvIII- but not aCD19-SGRP CAR? I recommend just inject the EGFRvIII- tumors (don't use the mixture) and test if these two CARs can inhibit the tumor growth.

3.8. Thank you for this observation and the comment about the mechanism of aEGFRvIII-SGRP CAR-mediated tumor clearance. Following the Reviewer's suggestion, we performed an *in vivo* experiment employing orthotopic U87 or U87+U251vIII tumors and treated these animals with one dose of aCD19-SGRP CAR or aEGFRvIII-SGRP CAR (n = 5 per group; Supplementary Fig. 4d). Three days after the CAR infusion, we collected the tumor-injected brain hemispheres and dissociated them for a spectral FC analysis of CAR T cell activation and to examine the effect of CAR target priming in shaping the tumor immune landscape. The expression of lineage markers was used to define 10 immune cell populations that were manually annotated (Supplementary Fig. 4e). We could observe differences in cell abundance between aCD19-SGRP CAR- and aEGFRvIII-SGRP CAR-treated brains (Supplementary Fig. 4f) regardless of the tumor type employed. The aEGFRvIII-SGRP CAR treatment resulted in an increased frequency of T cells in the brain, indicating higher baseline proliferation and activation of these CARs compared to aCD19-SGRP CAR. We previously had some indication of the higher baseline signaling of EGFRvIII-targeted CARs compared to CD19-targeted CARs from our *in vitro* experiments where EGFRvIII-targeted CARs showed a slightly higher baseline IFNγ release than CD19-targeted CARs in co-cultures with target-negative cell lines (Fig. 2e). Nonetheless, in our killing assays this higher baseline T cell activation did not result in off-target killing, as shown by the similar tumor

viability curves in the CAR T cell co-culture with target-negative tumor cells (Fig. 2b). Most significantly, in EGFRvIII-mosaic tumor-burdened brains, aEGFRvIII-SGRP CARs but not aCD19-SGRP CARs induced a significant influx of neutrophils, moMacs, and moDCs/cDC1s (Supplementary Fig. 4f). Concomitantly, we detected higher frequencies of $CD25^{hi}CD4^{+}$ CAR T cells in the EGFRvIII-mosaic, aEGFRvIII-SGRP CAR-treated condition (Supplementary Fig. 4f).

These data suggest that the GAM-modulating effect of SGRP relies on a broader influx of myeloid cell populations which is only triggered as a result of CAR T cell activation. In turn, the presence of a CAR target in the brain is crucial for the proliferation and persistence of activated CAR T cells. Consequently, an increased number of CAR T cells in the brain raises the amount of available SGRP within the tumor. Moreover, CAR, mCherry, and SGRP transgenes are expressed under the same promoter within one lentiviral construct. Therefore, a potential upregulation of CAR expression due to increased CAR-mediated tumor killing should equimolarly upregulate mCherry and SGRP expression (Fig. 1h). Sustained SGRP-mediated CD47 blockade on bystander tumor cells, combined with increased myeloid cell infiltration into the brain results in the clearance of EGFRvIII-mosaic GBM by aEGFRvIII-SGRP CARs but not by aCD19-SGRP CARs, as shown in Fig. 3c,d. Similarly, we showed that aEGFRvIII-SGRP CARs do not significantly improve survival compared to aEGFRvIII CARs in the $CD19^{+}$ lymphoma model (Fig. 7c). These results were described in the relevant results section of the revised manuscript (manuscript lines 213-225).

Reviewer #4 (Remarks to the Author): with expertise in CAR-T, glioblastoma

I would like to commend the authors on these well designed experiments exploring the potential benefit of blocking the CD47/SIRPγ pathway as a way to mitigate against GBM antigen heterogeneity. The authors did a good job at dissecting the TME and effects of blocking CD47 mediated inhibition of phagocytosis on GAMs.

However, the paper does not address the true benefit of engaging the GAM compartment of GBMs in vivo by blocking CD47 using secreted SIRPγ. What is missing is experiments both in vivo and in vitro addressing the benefit of having CAR T cells secreting SIRPγ in trans in bolstering GAM phagocytic activity.

Response

Dear Reviewer,

Thank you for your constructive feedback. We appreciate your acknowledgment of our study's design and objectives. In this revised version, we hope to fully address your comments, specifically on the benefits of further engaging the GAM compartment using opsonizing antibodies and the importance of CCL3 for SGRP-mediated tumor clearance. We are grateful for your valuable input and are convinced that it enhanced our manuscript significantly. All changes to the manuscript are highlighted in yellow. We look forward to your comments on the revised version. Thank you for your consideration.

The paper would be improved by evaluating if the secreted SIRPγ enhances phagocytosis in vitro using conditioned media from the CAR T cells secreting SIRPγ and the addition of a opsonizing antibody to a GBM associated antigen. Moreover, in vivo this could be addressed with a smaller experiment similar to the one outlined in Figure 3 where a mosaic GBM is dual targeted with CAR T cells secreting SIRPγ but complemented by the addition of opsonizing antibody that targets a tumor associated antigen found on antigen negative cells. These experiments will strengthen the paper as it goes gets to the heart of the project, can CAR T cells secreting SIRPγ lower the threshold for GAM mediated phagocytosis and engage them in mediating anti-tumor activity. Perhaps CD19 CAR P2A SIRPγ T cells complimented in trans with an EGFRvIII opsonizing antibody delivered IP at regular intervals. This would underscore the contribution of the GAM compartment being engaged to phagocytose tumor cells.

4.1. Thank you for this comment. We attempted to replicate the effect of aEGFRvIII-SGRP CAR in phagocytosis assays *in vitro* by combining EGFRvIII-mosaic tumor cells with donor-matched macrophages and CAR T cells. However, in these short-term assays, the presence of SGRP-secreting CAR T cells did not significantly influence macrophage polarization or phagocytic function, as determined by spectral FC (Supplementary Fig. 2g). These findings were described in the manuscript (manuscript lines 157-164) and discussed in detail in response 2.2 to Reviewer 2.

Following the Reviewer's suggestion, we performed a new *in vivo* experiment employing orthotopically U87-engrafted mice. U87 cells express EGFRwt (Fig. R3a) but low levels of EGFRvIII (Supplementary Fig. 2a, center histogram plot). Prior to this experiment, U87 cells were sorted for their $EGFRwt^{+}EGFRvIII^{-}$ fraction (Supplementary Fig. 2b, bottom contour plot). Tumor-burdened mice were primed with a high dose of EGFR opsonizing antibody (cetuximab; Erbitux, #L01FE01, Merck, Switzerland) and treated with one dose of off-target CARs (aCD19 CAR or aCD19-SGRP CAR), followed by two maintenance doses of cetuximab (Fig. R3b,c). Four days after the CAR infusion, we collected the tumor- and treatment-injected brain hemispheres of the animals and dissociated them for a spectral FC investigation of a potential synergistic effect of SGRP and cetuximab. We found no significant differences in tumor burden between the two groups (Fig. R3d). Using a myeloid-focused FC panel, we investigated potential differences in abundance and phenotypic modulation of myeloid cell populations in the brain. Ten clusters defined by specific cell lineage marker expression were manually annotated (Fig. R3e). Ultimately, the combination of cetuximab and off-target SGRP-secreting CAR T cells did not elicit a significant modulation of GAM subpopulations (Fig. R3f,g).

Importantly, aCD19-SGRP CAR T cells were infused into the brain but lacked a target therein. Without a target, CAR T cells do not become activated or come into close contact with tumor cells. This leads to SGRP not being substantially targeted to tumor cells, precluding GAM-mediated tumor cell phagocytosis. Without a target, CAR T cells quickly leak out of the brain, as shown also in the case of aEGFRvIII-SGRP CAR in our EGFRvIII-mosaic GBM model following tumor clearance (Fig. 5e and Supplementary Fig. 6b). Without sustained CAR targeting and SGRP secretion into tumor niches, we do not expect effective GAM modulation and tumor clearance, even with the addition of an opsonizing antibody.

We are aware that opsonizing antibodies were successfully used in other experimental paradigms in conjunction with SIRPα analogon-assisted CD47 blocking strategies, such as in the papers by Ring et al. (PMID 23722425) and, more recently, using a similar setup as ours, by Dacek et al. (PMID 36696633). In these paradigms, CD47 blockade and orthogonal opsonizing

antibodies were indeed synergistic and resulted in improved overall survival. Hence, as stated above, several factors may be responsible for the lack of synergy in the case of aCD19-SGRP CARs and opsonizing antibodies: (1) SGRP continued paracrine expression might be too low in the absence of a target to elicit an effect or to overcome the threshold for phagocytosis induction, (2) the above-mentioned studies investigated peripheral tumors, whereas the concept of opsonizing antibodies in conjunction with CD47 blockade was not tested in brain tumors so far, leading to (3) potential underdosing of cetuximab behind the blood-brain-barrier; on the other hand, in the presence of a target such as EGFRvIII, the strong immune-mediated cell death overcomes the threshold for additional phagocyte activation by local CD47 blockade, which results in the observed tumor control in our GBM and lymphoma xenografts.

Minor issues:

Figure 3 results and figure legends: include the CAR T cell dose in the texts for readers to follow along.

4.2. As suggested, we added mentions of CAR T cell and antibody doses for Fig. 3 in the results section and figure legend for better readability. We also applied the same to Fig. 7.

Figure 4: CCL3 was identified to be enriched in plasma of mice treated with EGFRvIII CAR + SIRPy T cells. Would blocking this GAM chemoattractant with CCL3 antibodies reduce the anti-tumor effect of SIRPy secreting CAR T cells? This paper is filled with hypothesis generating data but this would test some of your observations.

4.3. In response to this important comment, we tested our hypothesis of CCL3 acting as a crucial GAM chemoattractant for the efficacy of aEGFRvIII-SGRP CAR T cell therapy. For this, we applied a modified therapeutic scheme from Fig. 3a, assessing the efficacy of intratumoral aEGFRvIII-SGRP CAR with continued systemic infusion of an anti-human CCL3 antibody. Animals injected intratumorally with aEGFRvIII-SGRP CAR and systemically with an isotype IgG or PBS were used as controls (Supplementary Fig. 5e,f). Despite CCL3 blockade, the observed antitumor efficacy of aEGFRvIII-SGRP CAR T cells remained unchanged compared to isotype and PBS-injected controls (Supplementary Fig. 5f), suggesting that CCL3 is not essential in this context. This could be due to the upregulation of compensatory molecules, such as other chemokines or cytokines, which can maintain immune cell recruitment and activation. Additionally,

the redundancy within chemokine networks or CAR T cell-intrinsic properties might sustain their therapeutic effects independent of CCL3. This experiment was described in the relevant results section and in the discussion section of the revised manuscript (manuscript lines 263-273 and 438-443).

Figure 5: Given the interspecies nature of the immune cells involved in these experiments, it would help to designate the species of the marker being examined. For example murine CD206, human CD45, etc. Is it not clear to the reader if the markers outlined are human or mouse.

4.4. Thank you for this comment. We included descriptions of the markers' species reactivity in the manuscript text. Moreover, Supplementary Table 5 provides detailed lists of all antibody panels.

Point-by-point Response Figures

Figure R1

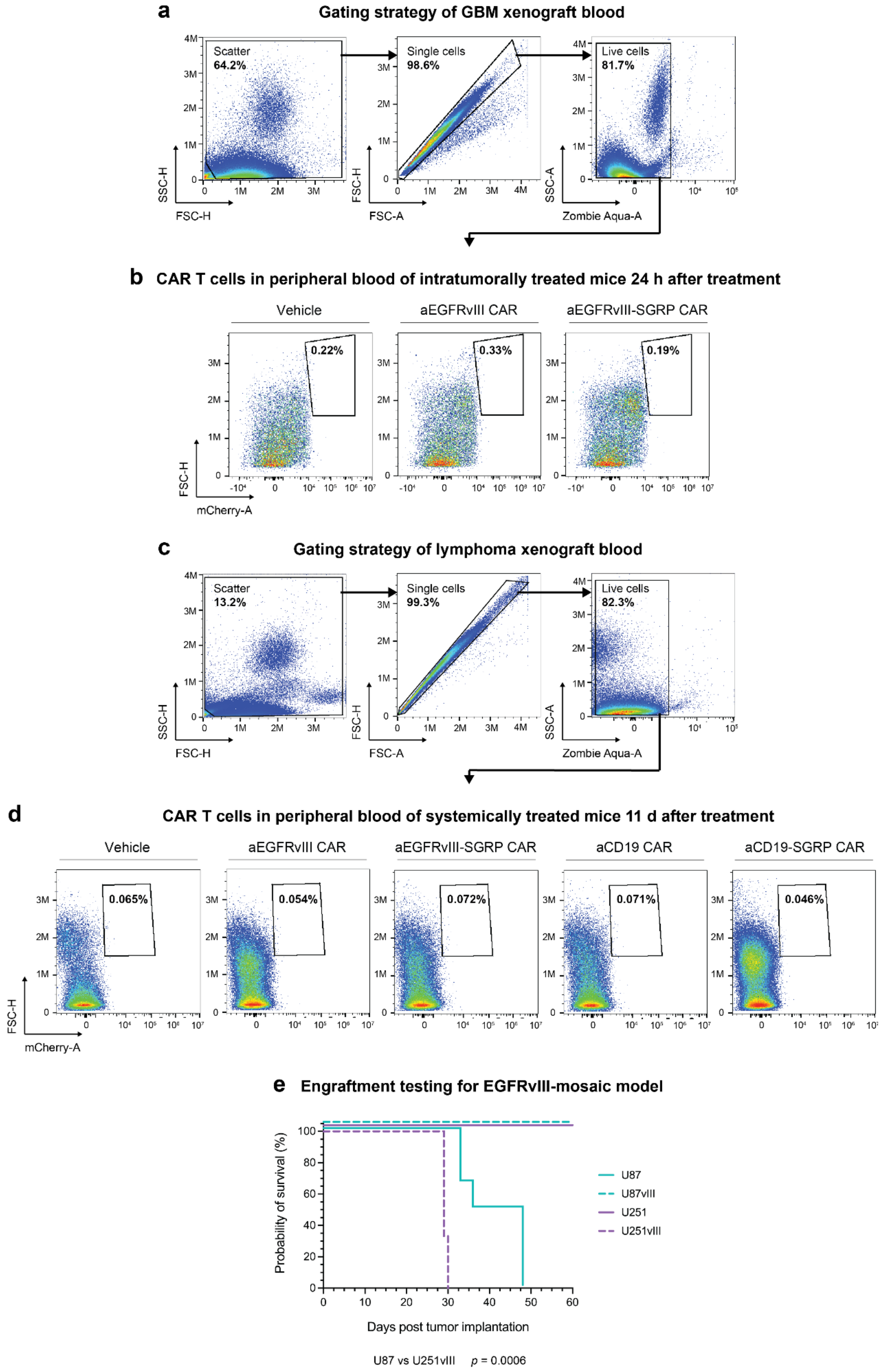

Figure R1. CAR T cell detection in peripheral blood and engraftment testing for GBM cell lines.

a, Representative pseudocolor plots of the gating strategy applied to isolate single, live (Zombie Aqua$^-$) cells within erythrocyte-depleted peripheral blood from EGFRvIII-mosaic GBM-engrafted mice. **b,** Representative pseudocolor plots derived from individual mouse blood samples per condition collected 24 h after i.t. CAR therapy, displaying the frequency of mCherry$^+$ CAR T cells. **c,** Representative pseudocolor plots of the gating strategy applied to isolate single, live (Zombie Aqua$^-$) cells within erythrocyte-rich peripheral blood from CD19$^+$ lymphoma-engrafted mice. **d,** Representative pseudocolor plots derived from individual mouse blood samples per condition collected 11 d after i.v. CAR therapy, displaying the frequency of mCherry$^+$ CAR T cells. **e,** Kaplan–Meier plot of overall survival (in days). Tumor cells were implanted intracranially (2.5×10^4 per animal; n = 6 per cell line). Log-rank test was used to compare U87 and U251vIII groups: $p = 0.0006$.

Figure R2

[figure redacted]

[figure redacted]

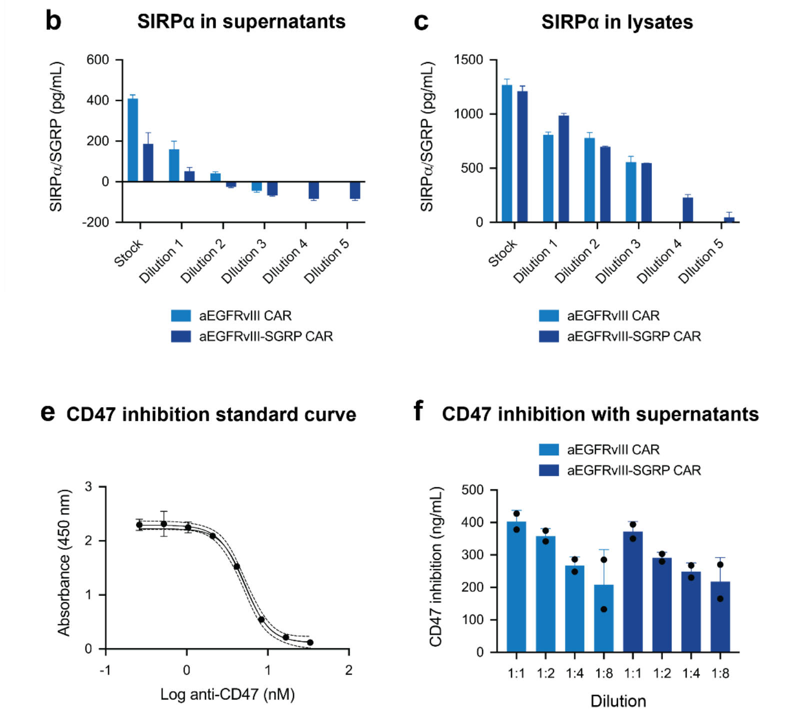

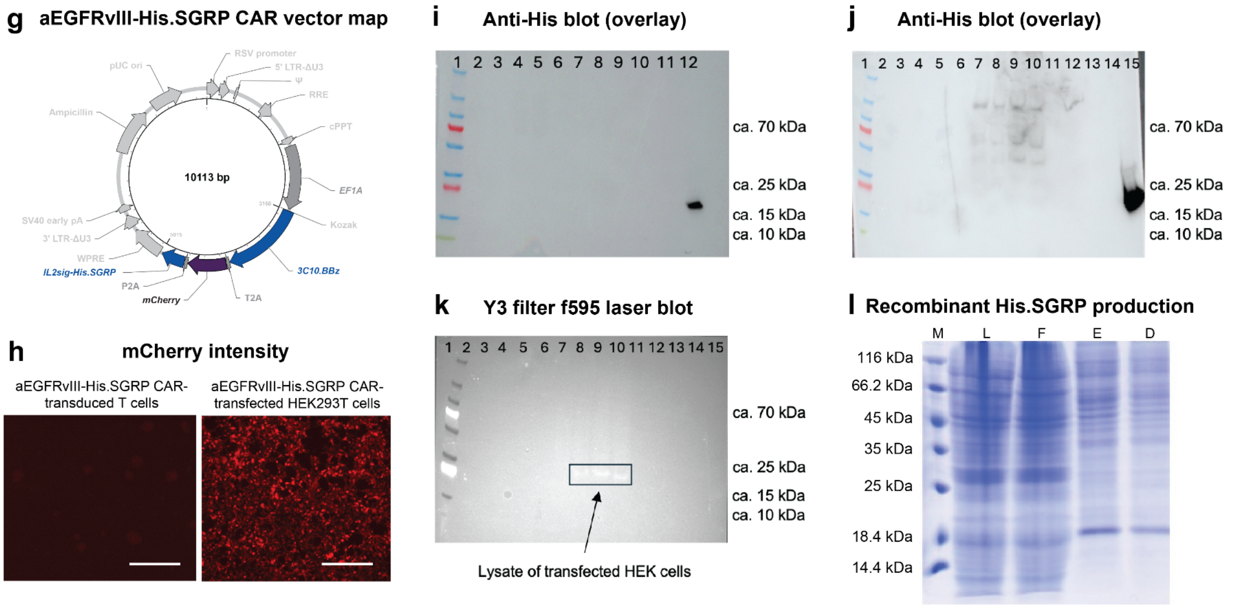

Figure R2. Attempts of SGRP detection via LC–MS-alternative methods.

a, Schematic representation of a SIRPα ELISA assay. **b,c,** Analysis of supernatants (**b**) and lysates (**c**) of CAR T cells secreting SGRP (aEGFRvIII-SGRP CAR) and negative control (aEGFRvIII CAR) at different dilution factors. **d,** Schematic representation of the CD47 inhibition ELISA assay. **e,** Standard curve with varying concentration of anti-CD47 antibody and fixed concentration of biotinylated (bt)-SIRPα. **f,** analysis of CAR T cell culture supernatants of aEGFRvIII-SGRP CAR and aEGFRvIII CAR at different dilution factors. **g,** Lentiviral vector map of aEGFRvIII-SGRP CAR with His-tagged SGRP (aEGFRvIII-His.SGRP CAR). **h,** Fluorescence microscopy images (RFP channel) of mCherry$^+$-sorted T cells 22 days after transduction or HEK293T cells 48 h after transfection; Scale bars: 500 µm. **i,** Anti-His blot of transduced T cell lysates and supernatants; SGRP expected molecular weight: ca. 15 kDa; Lanes: (1) Ladder; (2) aEGFRvIII-SGRP CAR supernatant; (3) aEGFRvIII-His.SGRP CAR supernatant; (4) aEGFRvIII-SGRP CAR lysate; (5) aEGFRvIII-His.SGRP CAR lysate; (6) His-purified aEGFRvIII-SGRP CAR eluate #1; (7) His-purified aEGFRvIII-His.SGRP CAR eluate #1; (8) His-purified aEGFRvIII-SGRP CAR eluate #2; (9) His-purified aEGFRvIII-His.SGRP CAR eluate #2; (10) His-purified aEGFRvIII-SGRP CAR eluate #3; (11) His-purified aEGFRvIII-His.SGRP CAR eluate #3; (12) dnPD-L1 6xHis positive control. **j,k,** Anti-His and f595 Y3 filter blots; SGRP expected molecular weight: ca. 15 kDa; Lanes: (1) Ladder; (2-6) His-purified supernatants - Control; His.SGRP #1; His.SGRP #2; His.SGRP #3; His.SGRP #4; (7-10) Cell lysates - SGRP control; His.SGRP #1; His.SGRP #2; His.SGRP #3; (11-14) Non-purified supernatants - SGRP control; His.SGRP #1; His.SGRP #2; His.SGRP #3; (15) aPD-L1 His-tag positive control. **l,** Recombinant SGRP production and recovery using a up-scaled mammalian (HEK293T) expression system; SGRP expected molecular weight: ca. 15 kDa; Lanes: (M) marker; (L) loaded raw supernatant; (F) flow-through sample; (E) eluted sample; (D) desalted sample.

Figure R3

[figure redacted]

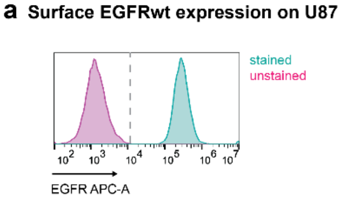

[figure redacted]

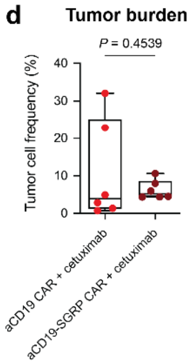

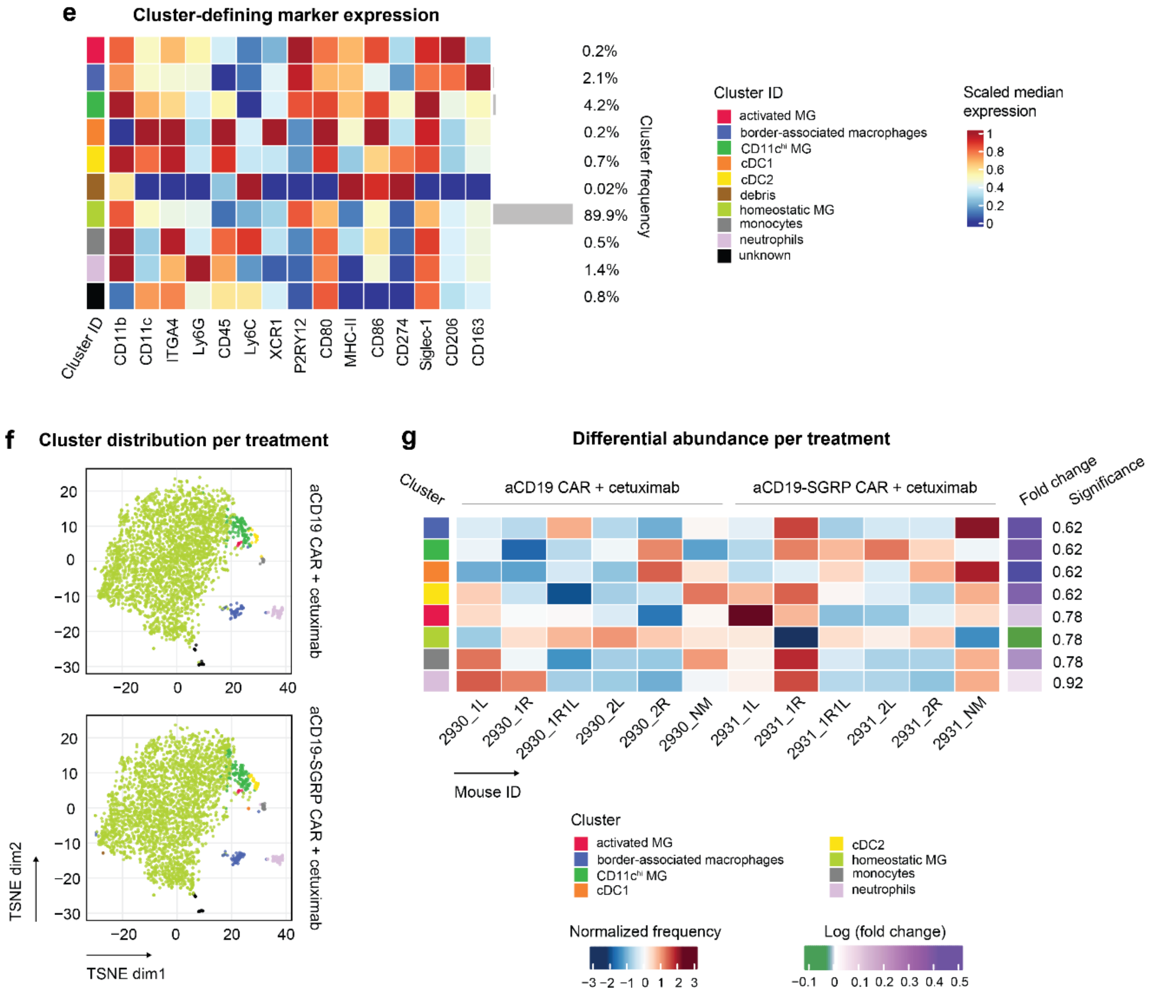

Figure R3. Combination of cetuximab and SGRP does not warrant synergistic tumor targeting by GAMs.

a, FC representation of EGFRwt surface expression on U87 tumor cells. **b,** Experimental treatment and monitoring schedule. Animals were treated once with CAR T cells – at 7 days – after intracranial (i.c.) tumor implantation using the same stereotactic coordinates and monitored for clinical signs and bioluminescence imaging (BLi) every 2 days. Systemic aEGFR opsonizing antibody doses were administered on days 6, 8, and 10. Animals were euthanized on day 11 post tumor implantation (n = 6 per group). **c,** Experimental setup of orthotopic xenograft experiments in NSG mice implanted with $EGFRvIII^-$ $EGFRwt^+$ U87 GBM cells and therapeutic cohorts, including local CAR T cell combination with aEGFR (cetuximab). CAR T cell dose: 5 x 10^5 cells delivered i.t.; Antibody dose (priming): 1 mg delivered i.p.; Antibody dose (maintenance): 0.5 mg delivered i.p.. **b,c,** Created with BioRender.com. **d,** Frequency of mTagBFP2-labeled U87 tumor cells in the brains of animals treated with aCD19 CAR + cetuximab or aCD19-SGRP CAR + cetuximab combinations; Each dot represents one animal; An unpaired t test with Welch's correction was used to compare the two conditions: p = 0.4539. **e,** Heatmap of scaled median cluster-defining cell lineage marker expression on 10 immune cell populations indicated on the left y-axis. Cluster frequency is indicated on the right y-axis. **f,** TSNE plots depicting 10 populations manually annotated based on the heatmap from (**e**). **g,** Heatmap of the differential abundance of immune cell populations per sample depicting no significant changes between aCD19 CAR + cetuximab and aCD19-SGRP CAR + cetuximab treatments. Significance cutoffs: false discovery rate = 0.05, log (fold change) = 0.5.

Point-by-point Response Tables

Table R1. Spectral FC panel used to characterize the brains of the off-target CAR and cetuximab combination cohort.

Fluorescently-tagged cell	Fluorescent protein
CAR T	mCherry
U87	mTagBFP2

Surface marker	Fluorophore	Clone	Host/Isotype	Reported reactivity	Catalog reference	Lot	Company	Stock concentration	Final dilution
CD11b	BUV395	M1/70	Rat DA	Human/Mouse	563553	3346840	BD Biosciences	0.2 mg/mL	1:1000
CD11c	BUV496	N418	Armenian Hamster IgG2	Mouse	750450	4079322	BD Biosciences	0.2 mg/mL	1:200
CD45	BUV737	30-F11	Rat LOU	Mouse	748371	2322187	BD Biosciences	0.2 mg/mL	1:4000
CD80	BV605	16-10A1	Armenian Hamster IgG	Mouse	104729	B357122	BioLegend	50 µg/mL	1:200
CD86	PE/Cy5	GL-1	Rat IgG2a, κ	Mouse	105016	B376879	BioLegend	0.2 mg/mL	1:1000
CD163	APC/Cy7	S15049I	Rat IgG2a, κ	Mouse	155323	B383053	BioLegend	0.2 mg/mL	1:100
CD206	AF700	C068C2	Rat IgG2a, κ	Mouse	141734	B384250	BioLegend	0.5 mg/mL	1:100
CD274	PE/Cy7	MIH6	Rat IgG2a, λ	Mouse	153613	-	BioLegend	0.2 mg/mL	1:800
ITGA4	BUV563	9C10(MFR4.B)	Rat LEW	Mouse	741243	3200542	BD Biosciences	0.2 mg/mL	1:800
Ly6C	BV785	HK1.4	Rat IgG2c, κ	Mouse	128041	B386418	BioLegend	0.2 mg/mL	1:10000
Ly6G	BUV661	1A8	Rat LEW	Mouse	741587	4012615	BD Biosciences	0.2 mg/mL	1:500
MHC-II	NF610-70S	M5/114.152	Rat IgG2b, κ	Mouse	M024T02B06	2925490	ThermoFisher Scientific	0.1 mg/mL	1:1000
P2RY12	APC/F810	S16007D	Rat IgG2b, κ	Mouse	848013	B39443	BioLegend	0.2 mg/mL	1:50
Siglec-1	FITC	3D6.112	Rat IgG2a, κ	Mouse	142405	B388729	BioLegend	0.5 mg/mL	1:100
XCR1	APC	ZET	Mouse IgG2b, κ	Mouse/Rat	148205	B349249	BioLegend	0.2 mg/mL	1:100

Viability dye	Catalog reference	Company	Final dilution
Zombie Aqua	423101	BioLegend	1:1000

Fc-blocking antibody	Target	Clone	Host/Isotype	Reported reactivity	Catalog reference	Lot	Company	Stock concentration	Final dilution
Human TruStain FcX	CD16/CD32/CD64	Unknown	Unknown	Human	422302	B369982	BioLegend	Unknown	1:50
TruStain FcX	CD16/CD32	93	Rat IgG2a, λ	Mouse	101320	B380119	BioLegend	0.5 mg/mL	1:50

Reviewer #1 (Remarks to the Author):

The authors have performed substantial new experiments and I appreciate the thoughtful responses to the reviews. Authors have adequately addressed all of my concerns and I am very comfortable recommending publication.

Response: We are pleased that the additional experiments and our point-by-point response have addressed your concerns. We appreciate the positive feedback and the recommendation for publication.

Reviewer #2 (Remarks to the Author):

The revised version is considerably improved with all additional experiments (including cytotoxicity experiments) and the detailed explanation of the author's interpretation. Overall the study presented is very comprehensive, original and well written. It is not extension of previous studies but a novel approach to improve CAT cells efficacy in GBM, where up to now most immunotherapies failed. The authors addressed thoroughly all comments of this reviewer (and others as well) and provided additional data supporting their interpretation. Altogether, the findings presented in the revised are convincing and dissolve some of the uncertainties regarding a design and execution some of the experiments. The authors were no evasive and justified their conclusions with proper experiments.

Response: We appreciate the comments on the study's design, comprehensiveness and originality. Thank you for the thorough review of both the original and revised versions of our manuscript. We are glad to have been able to provide additional data to support our interpretation and experimentally address the legitimate concerns that were raised.

Minor comments:

-I do agree with the authors comments regarding the use of a mosaic tumor model, and I accept the model usefulness for some mechanistic studies. However, some of the explanation about a tumor heterogeneity is not correct. While it is true that the presence of different clones with distinct genomic/transcriptomic profiles in GBM results in high genomic and phenotypic intratumoral heterogeneity, these are clones from the same tumor lineage with changes arising during tumor clonal evolution. In the model applied in the study tumor cells are entirely different and their intrinsic setting might contribute to various responses to therapy. I do agree that the model, albeit reductionistic, to some extent is useful to resolve the questions about therapy efficacy of new CART cells. It would be worthy to mention the model limitations.

-In the Introduction they used an abbreviation MoMacs for monocyte-derived macrophages. In the field MDM is frequently used for monocyte-derived macrophages, so it would better to sue the same abbreviation to describe the subpopulation.

Response: Thank you for the valuable feedback on the topic of glioblastoma heterogeneity. We agree that our EGFRvIII-mosaic GBM model does not capture the clonal evolution of GBM seen in the clinical setting. The mosaic tumor model we employed indeed consists of two tumor cell populations with distinct intrinsic characteristics, which can result in varied responses to therapy. While we acknowledge that this model is reductionistic, it nonetheless offers insights into the efficacy of anti-EGFRvIII CAR and anti-EGFRvIII-SGRP CAR T cells in targeting antigen-mosaic tumor populations. In response to this comment, we have added a section in the discussion that addresses the limitations of this model in reflecting the clonal heterogeneity of real GBM tumors and to emphasize the caution required in future clinical translation of these findings. Regarding the use of the term 'MoMacs' for monocyte-derived macrophages, we accept the suggestion and have replaced all instances of 'MoMacs' with the more commonly used abbreviation 'MDM' throughout the manuscript.

Reviewer #3 (Remarks to the Author):

The authors have spent a significant amount of effort addressing many of the suggestions from the reviewers. Overall, the revised manuscript demonstrates technical innovation with a potential impact on glioblastoma, which may compensate for the modest conceptual novelty.

Response: Thank you for acknowledging our efforts in addressing the comments you and the other Reviewers made that undoubtedly improved the quality of our manuscript. We also appreciate the positive comment regarding the technical innovation and potential impact of our study on glioblastoma. However, we respectfully disagree with the notion of modest conceptual novelty, as our work presents several aspects specifically tailored to the challenges of targeting GBM using anti-EGFRvIII CAR T cells, which have not been addressed in prior studies. Unlike previously published work combining CD47 blockade and CAR T cell therapy in subcutaneous leukemia models (Chen et al., Dacek et al., and Lu et al.), our study applies this approach to GBM using orthotopic brain tumor models, which present a more complex myeloid immune microenvironment. Additionally, we combined our CAR T cells with SGRP, a SIRPγ-derived protein with significantly higher affinity for CD47 than conventional SIRPα analogs used in previous studies. Furthermore, we provide a deeper characterization of the immune tumor microenvironment in GBM, adding translational relevance and novelty to our study. We believe this combination of factors significantly advances the field.

Reviewer #4 (Remarks to the Author):

I think the authors have done a very nice job responding to the queries and the paper looks great.

Response: Thank you very much for your feedback. We are pleased to have fully addressed your concerns and suggestions.
